# Supplementary material for: Electrophotocatalytic hydrogenation of imines and reductive functionalization of aryl halides
Source: Nat Commun. 2024 Jan 22;15:655. doi: 10.1038/s41467-024-45015-6 (PMC10803379; doi:10.1038/s41467-024-45015-6)
Supplement: Supplementary file 1 — Supplementary Information [file 41467_2024_45015_MOESM1_ESM.pdf]

Supplementary Information for

Electrophotocatalytic hydrogenation of imines and reductive functionalization of aryl halides

Wen-Jie Kang[1], Yanbin Zhang[1]*, Bo Li[2]*, and Hao Guo[1]*

[1]Department of Chemistry, Fudan University, 2005 Songhu Road, Shanghai 200438, PR China, E-mail: ybzhang@nus.edu.sg; Hao_Guo@fudan.edu.cn.

[2]Division of Chemistry and Chemical Engineering, California Institute of Technology, Pasadena, CA 91106, United States, E-mail: bli3@caltech.edu.

Table of Contents

General information S3

General setup for the electrophotochemical reaction S4

Graphic guide for setting up the electrophotocatalytic reaction S5

Optimization of the reaction conditions S6

Mechanistic studies S10

- Cyclic voltammetry S10
- Fluorescence spectra S13
- Fluorescence lifetime S14
- Electron paramagnetic resonance S15
- Stern-Volmer luminescence quenching experiments S19
- Controlled potential electrolysis S21
- Light on-off experiments S22
- Deuterium labeling studies S23
- Faradaic Efficiency S24
- Computational method S26

Experimental procedures S28

- Typical procedure I for the synthesis of substrates S28
- Typical procedure II for the synthesis of substrates S31
- Typical procedure III for the electrophotocatalytic imine hydrogenation S33
- Typical procedure IV for the electrophotocatalytic reductive functionalization S43

Gram-scale synthesis and synthetic derivatization S50

Compared with the previous strategies for electrophotocatalytic coupling reactions S55

NMR spectra of substrates and products S57

Supplementary references S100

General information

All electrophotochemical reactions were carried out using 415 nm LED strip (height: 10 cm, diameter: 15 cm, 60 W) at a distance of 3-5 cm at 30 °C unless stated otherwise. ^{1}H (400 MHz), ^{13}C (101 MHz), and ^{11}B (128 MHz) NMR spectra of samples in $CDCl_3$ or DMSO-d_6 were recorded on an AVANCE III 400 spectrometer. IR spectra were recorded on an Avatar 360 FT-IR spectrometer. HRMS (ESI) determinations of **23S**, **24S**, **10**, **14**, **16**, **23**, **24**, **38**, **52** and **53** were carried out on an Aglient 7250 & JEOL-JMS-T100LP AccuTOF spectrometer. HRMS (EI) determinations of **8**, **17**, **26**, **37**, **44** and **51** were carried out on an Agilent Technologies 7250 GCQTOF spectrometer. Melting points were determined on a WRS-2 apparatus. Cyclic voltammograms were recorded using a CHI760E instrument. Electron paramagnetic resonance (EPR) spectra was obtained using an EMX-8/2.7100G-18KG instrument. UV/Vis absorption spectra were recorded on a Thermo Evolution 220 UV/Vis spectrometer. Fluorescence spectra were collected by Hitachi FL-4700 fluorescence spectrometer equipped with chopping systems. The fluorescence decay profiles in nanosecond region were recorded by using time-correlated single photon counting technique (TCSPC) on an Edinburgh FLS1000 fluorescence spectrometer equipped with a picosecond pulsed diode laser. Anhydrous DCE, MeCN, toluene, and *p*-xylene were distilled with CaH_2. Anhydrous THF and 1,4-dioxane was distilled with Na using benzophenone as monitor. Substrates (**5**, **7S**, **9S**, **11S**, **12S**, **15S**, **25S**, **26S**, **27S**, **28S**, **29S**, **30S**, **31S**, **32S**, **33S**, **34S**, **35S**, **36S**, **37S**, **38S**, **40S**, **41S**, **42S**, and **44S)** were commercially available and used as purchased without further purification.

General setup for the electrophotochemical reaction

Supplementary Figure 1. Spectral distribution of irradiance density for the 415 nm LED strip

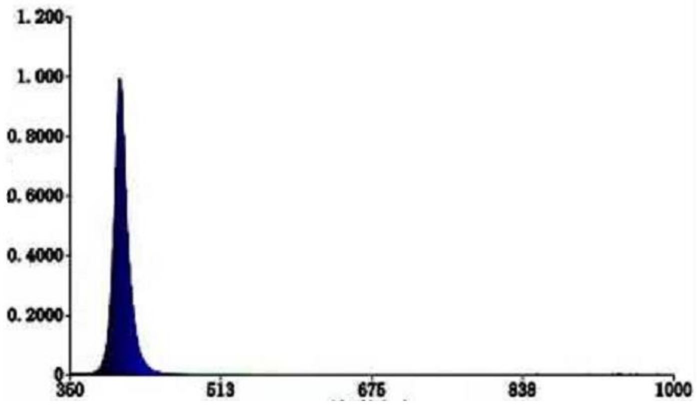

Supplementary Figure 2. General setup and gram-scale setup maintained with four cooling fans.

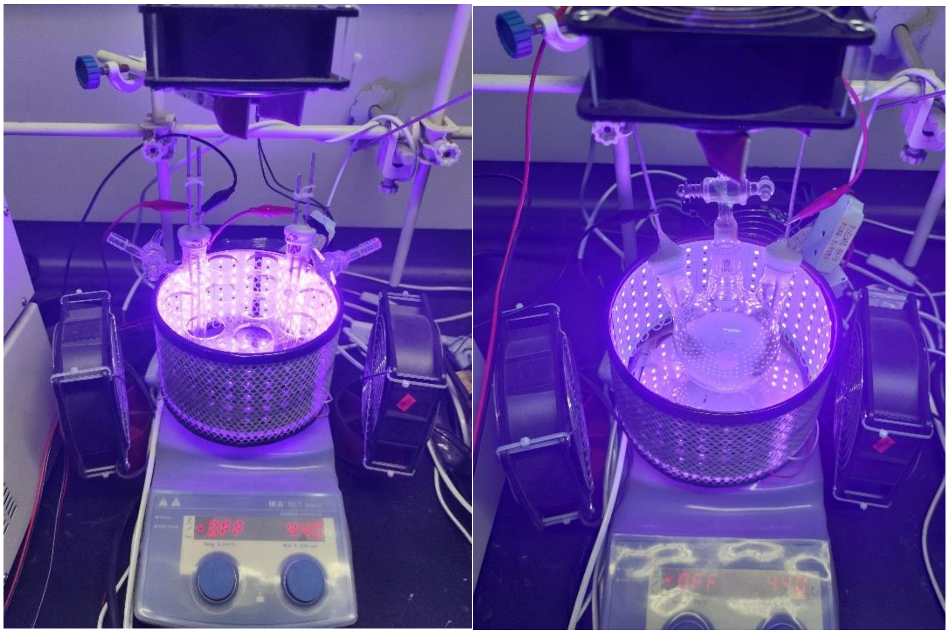

Graphic guide for setting up the electrophotocatalytic reaction

Supplementary Figure 3. Preparation of electrodes

Imine hydrogenation

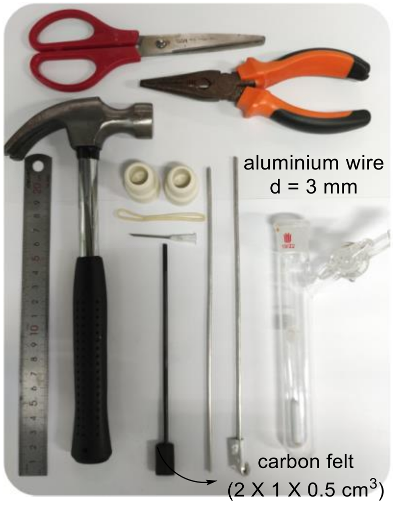

Reductive functionalization

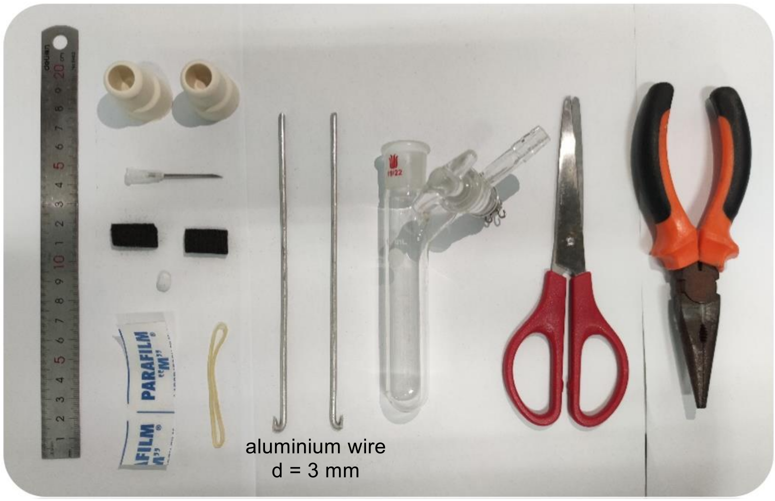

Supplementary Figure 4. Setting up the reaction

Standard Condition I

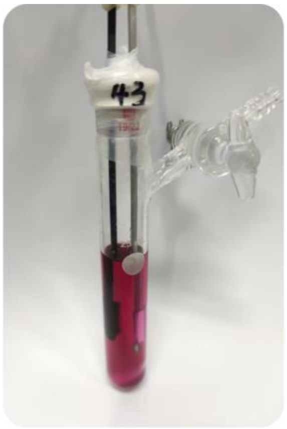

Standard Condition II

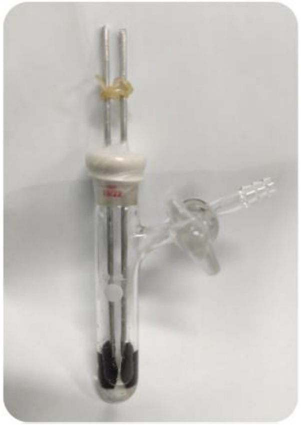

Optimization of the reaction conditions

Supplementary Table 1. Electrophotocatalytic imine hydrogenation[a]

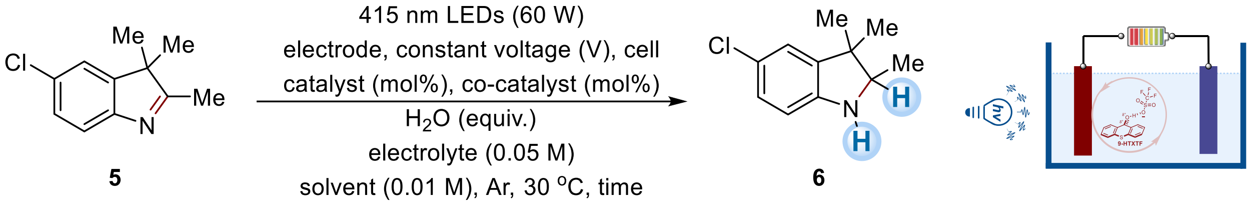

entry	solvent	electrode	electrolyte	catalyst (mol%)	TfOH (mol%)	H_2O (equiv.)	voltage (V)	time (h)	^{1}H NMR yield[b] (%) 5	^{1}H NMR yield[b] (%) 6
1	DMF	Al(+)/C(–)	$^{n}Bu_4NPF_6$	TX (10)	10	3	4.0	24	0	0
2	$MeNO_2$	Al(+)/C(–)	$^{n}Bu_4NPF_6$	TX (10)	10	3	4.0	24	0	0
3	THF	Al(+)/C(–)	$^{n}Bu_4NPF_6$	TX (10)	10	3	4.0	24	0	0
4	MeOH	Al(+)/C(–)	$^{n}Bu_4NPF_6$	TX (10)	10	3	4.0	24	7	0
5	Acetone	Al(+)/C(–)	$^{n}Bu_4NPF_6$	TX (10)	10	3	4.0	24	61	0
6	EA	Al(+)/C(–)	$^{n}Bu_4NPF_6$	TX (10)	10	3	4.0	24	93	0
7	MeCN	Al(+)/C(–)	$^{n}Bu_4NPF_6$	TX (10)	10	3	4.0	24	50	16
8	DCE	Al(+)/C(–)	$^{n}Bu_4NPF_6$	TX (10)	10	3	4.0	24	10	52
9	DCE	Al(+)/Pt(–)	$^{n}Bu_4NPF_6$	TX (10)	10	3	4.0	24	50	0
10	DCE	Al(+)/Al(–)	$^{n}Bu_4NPF_6$	TX (10)	10	3	4.0	24	82	0
11	DCE	Al(+)/Zn(–)	$^{n}Bu_4NPF_6$	TX (10)	10	3	4.0	24	89	4
12	DCE	Al(+)/Ag(–)	$^{n}Bu_4NPF_6$	TX (10)	10	3	4.0	24	61	9
13	DCE	Al(+)/Cu(–)	$^{n}Bu_4NPF_6$	TX (10)	10	3	4.0	24	41	18
14	DCE	Al(+)/Fe(–)	$^{n}Bu_4NPF_6$	TX (10)	10	3	4.0	24	35	29
15	DCE	Fe(+)/C(–)	$^{n}Bu_4NPF_6$	TX (10)	10	3	4.0	24	71	19

16	DCE	Cu(+)/C(−)	$^{n}Bu_4NPF_6$	TX (10)	10	3	4.0	24	54	20
17	DCE	Pt(+)/C(−)	$^{n}Bu_4NPF_6$	TX (10)	10	3	4.0	24	16	33
18	DCE	Al(+)/C(−)	$^{n}Bu_4NI$	TX (10)	10	3	4.0	24	75	0
19	DCE	Al(+)/C(−)	$^{n}Bu_4NOTf$	TX (10)	10	3	4.0	24	87	0
20	DCE	Al(+)/C(−)	$^{n}Bu_4NClO_4$	TX (10)	10	3	4.0	24	46	23
21	DCE	Al(+)/C(−)	$^{n}Bu_4NPF_6$	TX-4 (10)	10	3	4.0	24	12	20
22	DCE	Al(+)/C(−)	$^{n}Bu_4NPF_6$	TX-5 (10)	10	3	4.0	24	36	24
23	DCE	Al(+)/C(−)	$^{n}Bu_4NPF_6$	TX-3 (10)	10	3	4.0	24	14	29
24	DCE	Al(+)/C(−)	$^{n}Bu_4NPF_6$	TX-6 (10)	10	3	4.0	24	0	43
25	DCE	Al(+)/C(−)	$^{n}Bu_4NPF_6$	TX-1 (10)	10	3	4.0	24	43	48
26	DCE	Al(+)/C(−)	$^{n}Bu_4NPF_6$	TX-2 (10)	10	3	4.0	24	0	51
27	DCE	Al(+)/C(−)	$^{n}Bu_4NPF_6$	TX (10)	10	1	4.0	24	0	41
28	DCE	Al(+)/C(−)	$^{n}Bu_4NPF_6$	TX (5)	10	3	4.0	24	0	49
29	DCE	Al(+)/C(−)	$^{n}Bu_4NPF_6$	TX (2.5)	10	3	4.0	42	0	25
30	DCE	Al(+)/C(−)	$^{n}Bu_4NPF_6$	TX (5)	5	3	4.0	36	0	41
31	DCE	Al(+)/C(−)	$^{n}Bu_4NPF_6$	TX (5)	10	3	4.5	10	0	45
32	DCE	Al(+)/C(−)	$^{n}Bu_4NPF_6$	TX (5)	10	3	3.0	13	0	54
33	DCE	Al(+)/C(−)	$^{n}Bu_4NPF_6$	TX (5)	10	3	2.0	16	0	76
34	DCE	Al(+)/C(−)	$^{n}Bu_4NPF_6$	TX (5)	10	3	1.8	22	0	87 (85)[c]
35	DCE	Al(+)/C(−)	$^{n}Bu_4NPF_6$	TX (5)	10	3	1.5	22	17	74
36[d]	DCE	Al(+)/C(−)	$^{n}Bu_4NPF_6$	TX (5)	10	3	1.8	22	46	46
37[e]	DCE	Al(+)/C(−)	$^{n}Bu_4NPF_6$	TX (5)	10	3	1.8	22	99	0

38[f]	DCE	Al(+)/C(−)	^{n}Bu$_4$NPF$_6$	TX (5)	10	3	1.8	22	99	0
39[g]	DCE	Al(+)/C(−)	^{n}Bu$_4$NPF$_6$	TX (5)	10	3	1.8	22	84	0
40	DCE	Al(+)/C(−)	--	TX (5)	10	3	1.8	22	73	15
41	DCE	Al(+)/C(−)	^{n}Bu$_4$NPF$_6$	--	10	3	1.8	22	77	9
42	DCE	Al(+)/C(−)	^{n}Bu$_4$NPF$_6$	TX (5)	--	3	1.8	22	75	7
43	DCE	Al(+)/C(−)	^{n}Bu$_4$NPF$_6$	TX (5)	10	--	1.8	22	77	13
44	DCE	Al(+)/C(−)	^{n}Bu$_4$NPF$_6$	TX (5)	10	3	--	22	85	9

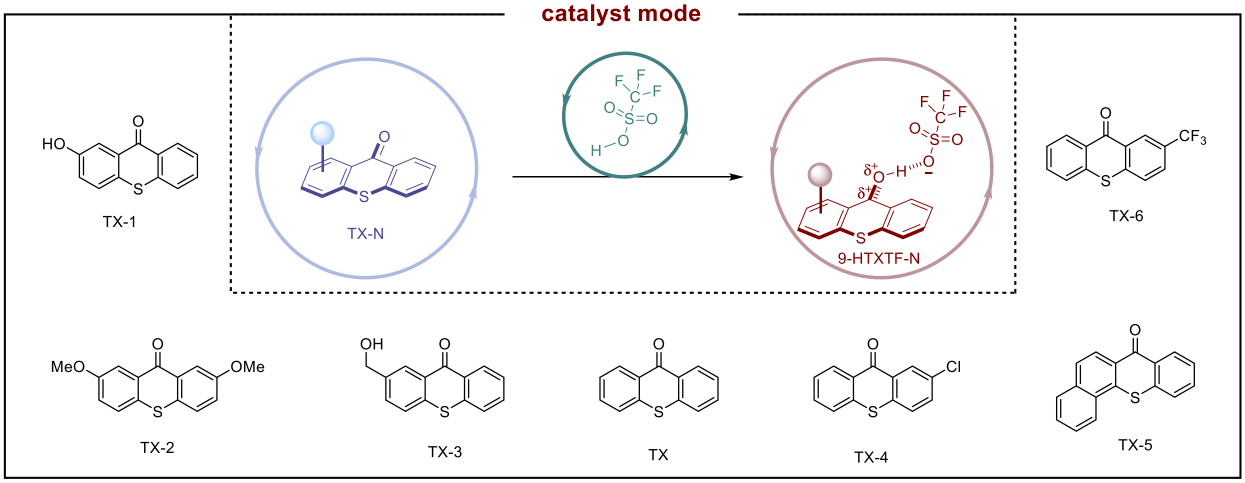

[a]Reaction conditions: **5** (0.2 mmol), electrode, constant voltage (U), catalyst, TfOH, H_2O, electrolyte (0.05 M), solvent (20 mL), cell, 415 nm LEDs (60 W), 30 °C, argon atmosphere, 10-36 h. [b]Yield and recovery were determined by ^{1}H NMR analysis (400 MHz) of the crude reaction mixture using CH_2Br_2 (0.2 mmol) as the internal standard. [c]Isolated yield of **6**. [d]Divided cell. [e]The reaction was carried out without light. [f]The reaction was carried out at 60 °C without light. [g]The reaction was carried out at 80 °C without light. Note：Due to different electrode batches, the content of TfOH (10-20 mol%) will fluctuate.

Supplementary Table 2. Electrophotocatalytic reductive dehalogenation[a]

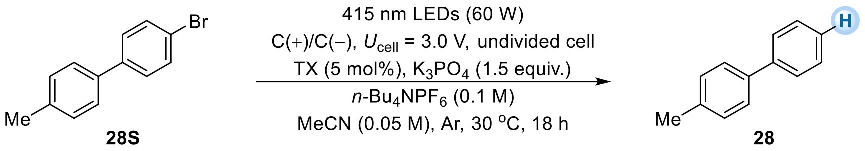

Entry	Variation from standard conditions	Yield[b] (%)
1	none	92 (86)[c]
2	no photo irradiation	0 (100)
3	no applied voltage	0 (100)
4	no TX	5 (93)
5	no K_3PO_4	8 (92)
6	50 mol% K_3PO_4	57 (42)
7	no n-Bu_4NPF_6	0 (100)

[a]Reaction conditions: **28S** (0.2 mmol), electrode, constant voltage (U), TX, K_3PO_4, electrolyte, MeCN (4 mL), undivided cell, 415 nm LEDs (60 W), 30 °C, argon atmosphere, 18 h. [b]Yield and recovery were determined by ^1H NMR analysis (400 MHz) of the crude reaction mixture using CH_2Br_2 (0.2 mmol) as the internal standard. Unreacted **28S** in parenthesis. [c]Isolated yield of **28**.

Mechanistic studies

Cyclic voltammetry

Cyclic voltammograms were recorded using a CHI760E instrument model and a glassy carbon working electrode, an Ag/AgCl reference electrode and a Pt wire counter electrode.

Supplementary Figure 5. The cyclic voltammogram of TX

The voltammogram of TX was recorded at room temperature in anhydrous degassed tetrabutylammonium perchlorate (0.1 M) in MeCN (8 mL) containing TX (4 mM). The scan rate was 100 mV/s.

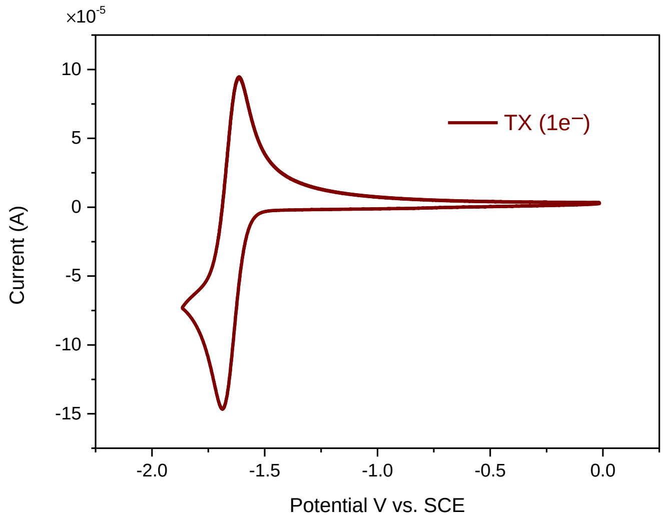

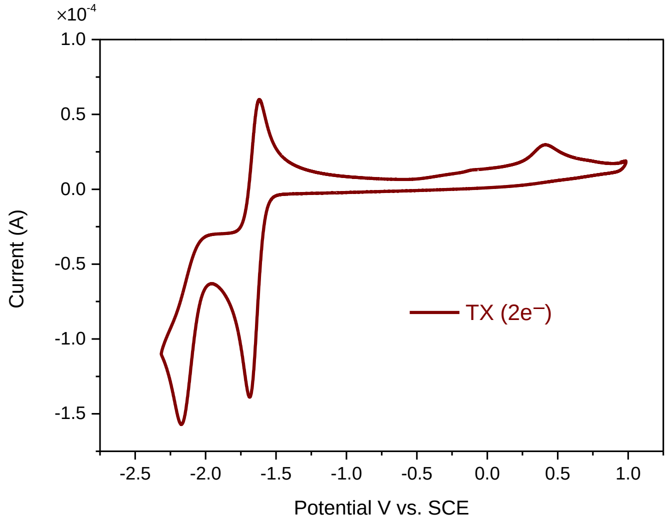

Supplementary Figure 6. The cyclic voltammogram of 1

The voltammogram of 9-HTXTF (**1**) was recorded at room temperature in anhydrous degassed tetrabutylammonium perchlorate (0.1 M) in MeCN (8 mL) containing TX (4 mM) and TfOH (4 mM). The scan rate was 100 mV/s. The excited-state oxidation potential of **4** was estimated according to literature methods[1-3].

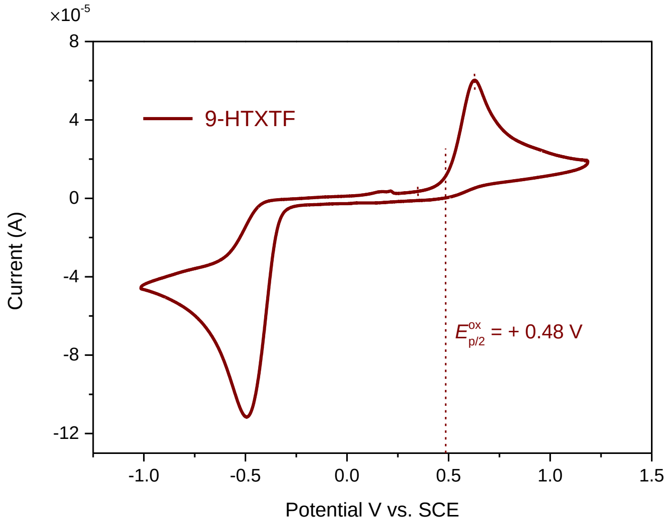

$$E^{ox}_{1/2,} = +\ 0.48\ \text{V vs. SCE}$$

$$E_{0,0,\ 9\text{-HTX}^-} = \frac{3.54\ \text{ev}\ (350\ \text{nm}) + 2.85\ \text{ev}\ (435\ \text{nm})}{2} = 3.20\ \text{ev}$$

$$E^{ox}_{1/2,\ 9\text{-HTX}^{-*}} = +\ 0.48\ \text{V vs. SCE}\ -3.20\ \text{eV} = -\ 2.72\ \text{V vs. SCE} \approx -2.7\ \text{V vs. SCE}$$

Supplementary Figure 7. The cyclic voltammogram of substrate

The voltammogram of substrate was recorded at room temperature in anhydrous degassed tetrabutylammonium perchlorate (0.1 M) in MeCN (8 mL) containing substrate (4 mM). The scan rate was 100 mV/s.

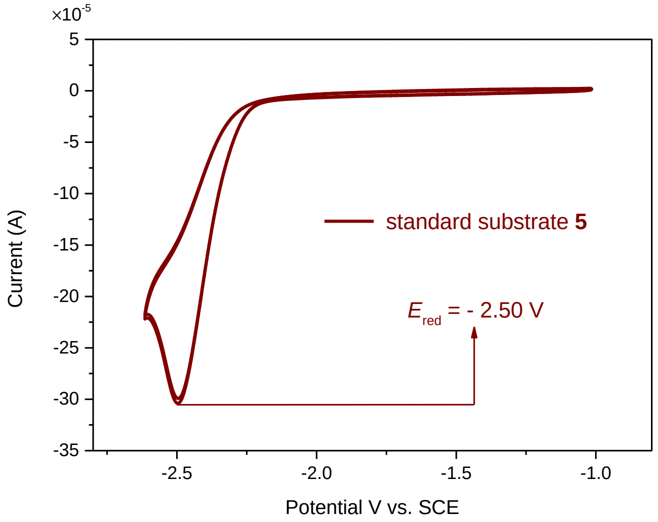

$$E_{\text{red, substrate } \mathbf{5}} = -\ 2.5\ \text{V vs. SCE}$$

$$E^{\text{ox}}_{1/2,\ 9\text{-HTX}^{-*}} < E_{\text{red, substrate } \mathbf{5}}$$

Supplementary Figure 8. Known reduction potentials for reductive functionalization

The reduction potentials of **29S**, **42S** and **43S** were cited from the literature[4].

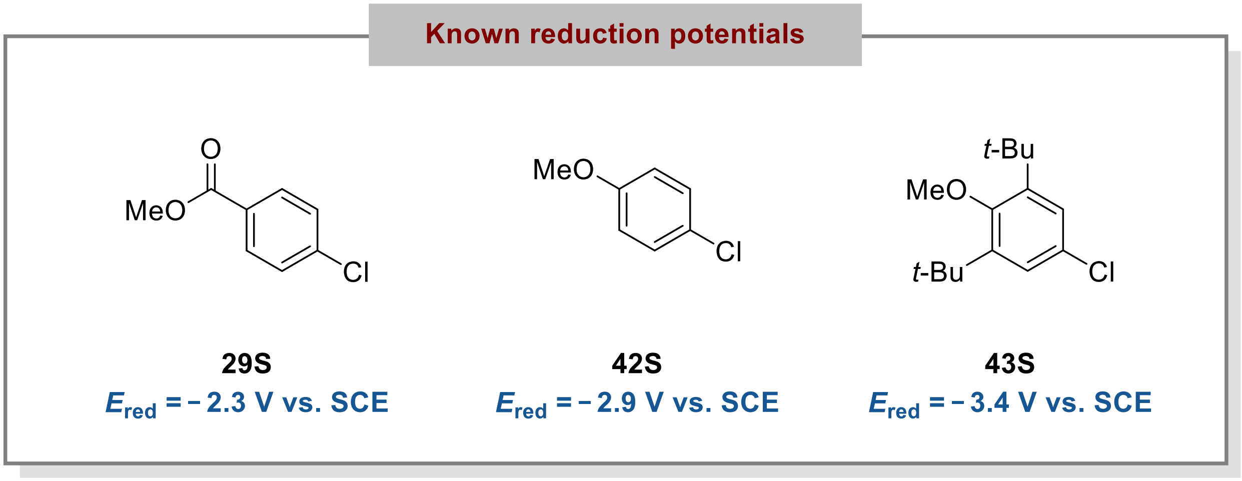

Supplementary Figure 9. Electrolysis of 9-HTXTF

To a flame dried 10 mL of Schlenk tube were added thioxanthone (5 mM), TfOH (10 mM), *n*-Bu_4NPF_6 (0.2 M), and anhydrous DCE (10 mL) under argon atmosphere. The cell was equipped with a carbon cathode and an aluminum anode, and was sealed using a rubber septum and parafilm. The reaction mixture was electrolyzed at a constant cell potential of 1.8 V at 30 °C (maintained with four cooling fans) for 5 d to gain the reduced species of 9-HTXTF (note: In order to carefully study the reduced species of 9-HTXTF, it was synthesized in large quantities, which required longer reaction times compared to the catalytic imine hydrogenation reaction.).

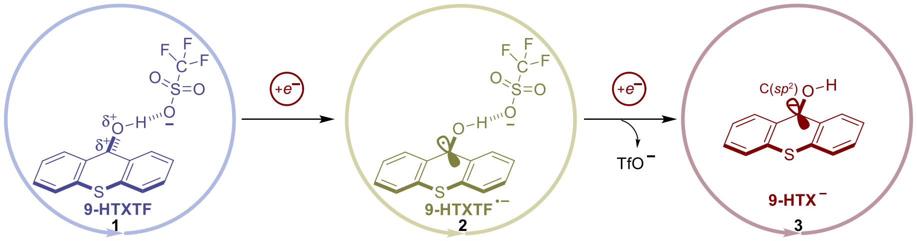

Fluorescence spectra

Supplementary Figure 10. Fluorescence spectra of the reduced species of 1

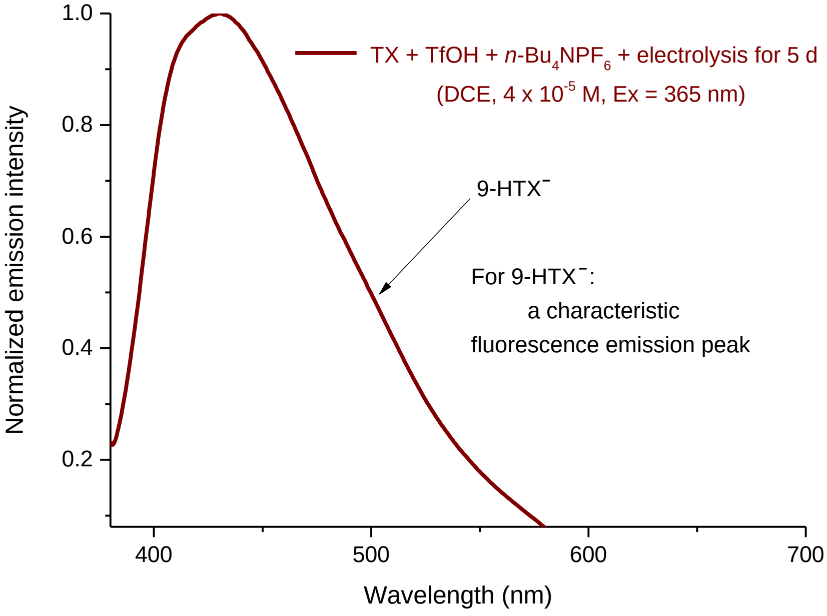

Fluorescence lifetime

Supplementary Figure 11. Fluorescence lifetime of the reduced species of 1

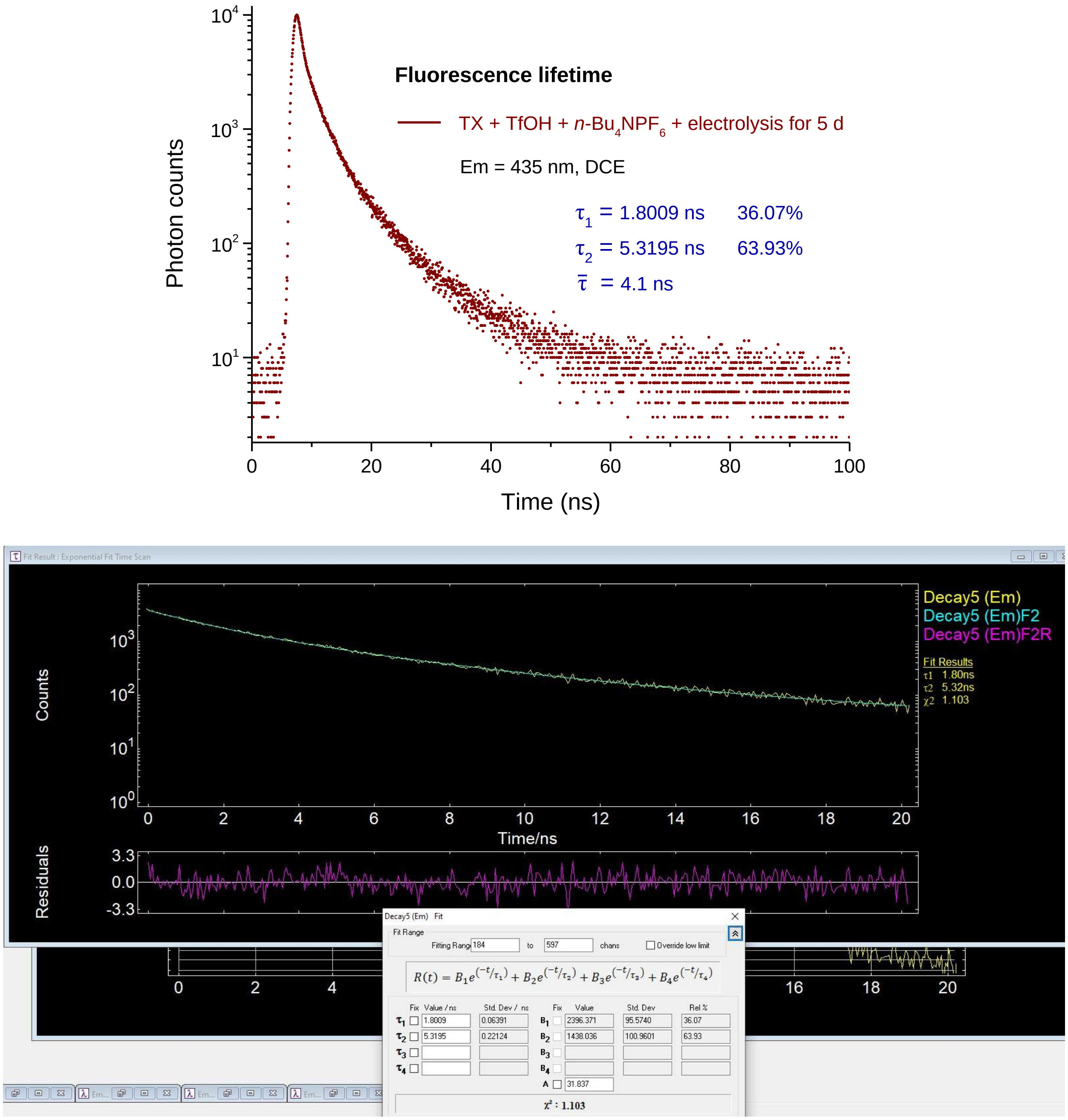

Electron paramagnetic resonance

Supplementary Figure 12. The simulation of the EPR spectrum of 2

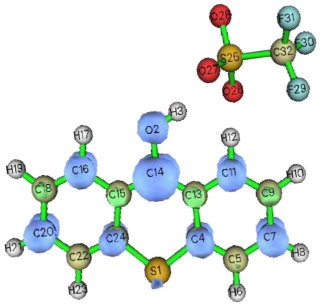

B3LYP-D3(BJ)/def2-SVP

Spin population based on Becke method

Atomic space	Value	% of sum	% of sum abs
1(S)	0.06679737	6.679738	4.840129
2(O)	0.12295132	12.295134	8.909036
3(H)	0.00027921	0.027921	0.020231
4(C)	0.09144513	9.144515	6.626102
5(C)	-0.02897965	-2.897965	-2.099862
6(H)	-0.00008680	-0.008680	-0.006290
7(C)	0.12585307	12.585309	9.119297
8(H)	-0.00007514	-0.007514	-0.005445
9(C)	-0.03843259	-3.843259	-2.784820
10(H)	0.00011738	0.011738	0.008506
11(C)	0.11783158	11.783160	8.538060
12(H)	-0.00028986	-0.028986	-0.021003
13(C)	-0.03485511	-3.485512	-2.525597
14(C)	0.32951077	32.951083	23.876307
15(C)	-0.02973124	-2.973124	-2.154322
16(C)	0.12225720	12.225723	8.858741
17(H)	-0.00012957	-0.012957	-0.009389
18(C)	-0.03647953	-3.647953	-2.643302
19(H)	0.00008410	0.008410	0.006094
20(C)	0.12816328	12.816330	9.286694

21(H)	-0.00000586	-0.000586	-0.000425
22(C)	-0.02062560	-2.062560	-1.494528
23(H)	-0.00008926	-0.008926	-0.006468
24(C)	0.08343536	8.343537	6.045715
25(S)	0.00004508	0.004508	0.003267
26(O)	0.00118933	0.118933	0.086179
27(O)	-0.00020564	-0.020564	-0.014901
28(O)	-0.00000432	-0.000432	-0.000313
29(F)	0.00007687	0.007687	0.005570
30(F)	-0.00000956	-0.000956	-0.000693
31(F)	-0.00000059	-0.000059	-0.000042
32(C)	-0.00003691	-0.003691	-0.002675

Summing up above values: 0.99999982

Summing up absolute value of above values: 1.38007428

Supplementary Figure 13. The simulated EPR signal of 2

The simplest way to include line broadening is to convolute a stick spectrum with a (Gaussian or Lorentzian) lineshape after the end of the simulation. Such a convolution broadening is specified in the spin system field lwpp.

B3LYP-D3(BJ)/def2-TZVP.

Sys.lwpp = 0.15.

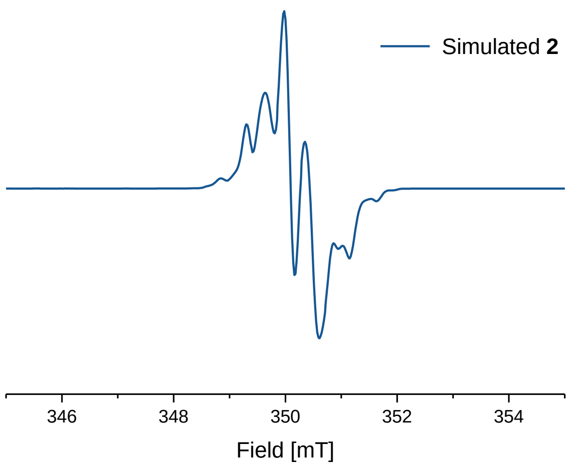

Supplementary Figure 14. Electron paramagnetic resonance characterization of the reduced species

A solution of TX (0.02 mmol, 4.2 mg), TfOH (0.04 mmol, 3.5 μL), and *n*-Bu_4NPF_6 (0.14 mmol, 54.1 mg) in $CDCl_3$ (3 mL) was electrolyzed at a constant cell potential of 1.8 V (C(+)/C(-)) at rt under argon atmosphere for 5 d. EPR spectrum was obtained using an EMX-8/2.7100G-18KG instrument, as shown below:

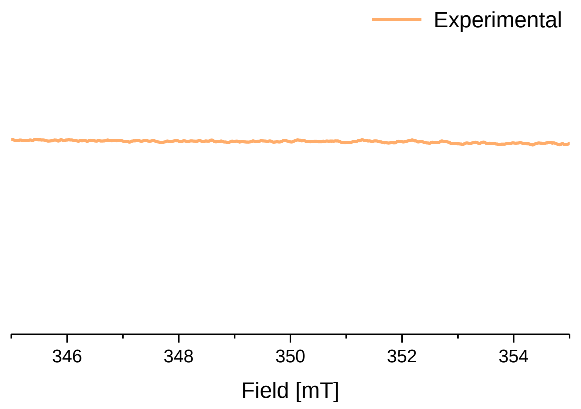

Supplementary Figure 15. ^{1}H NMR analysis of the reduced species of 1

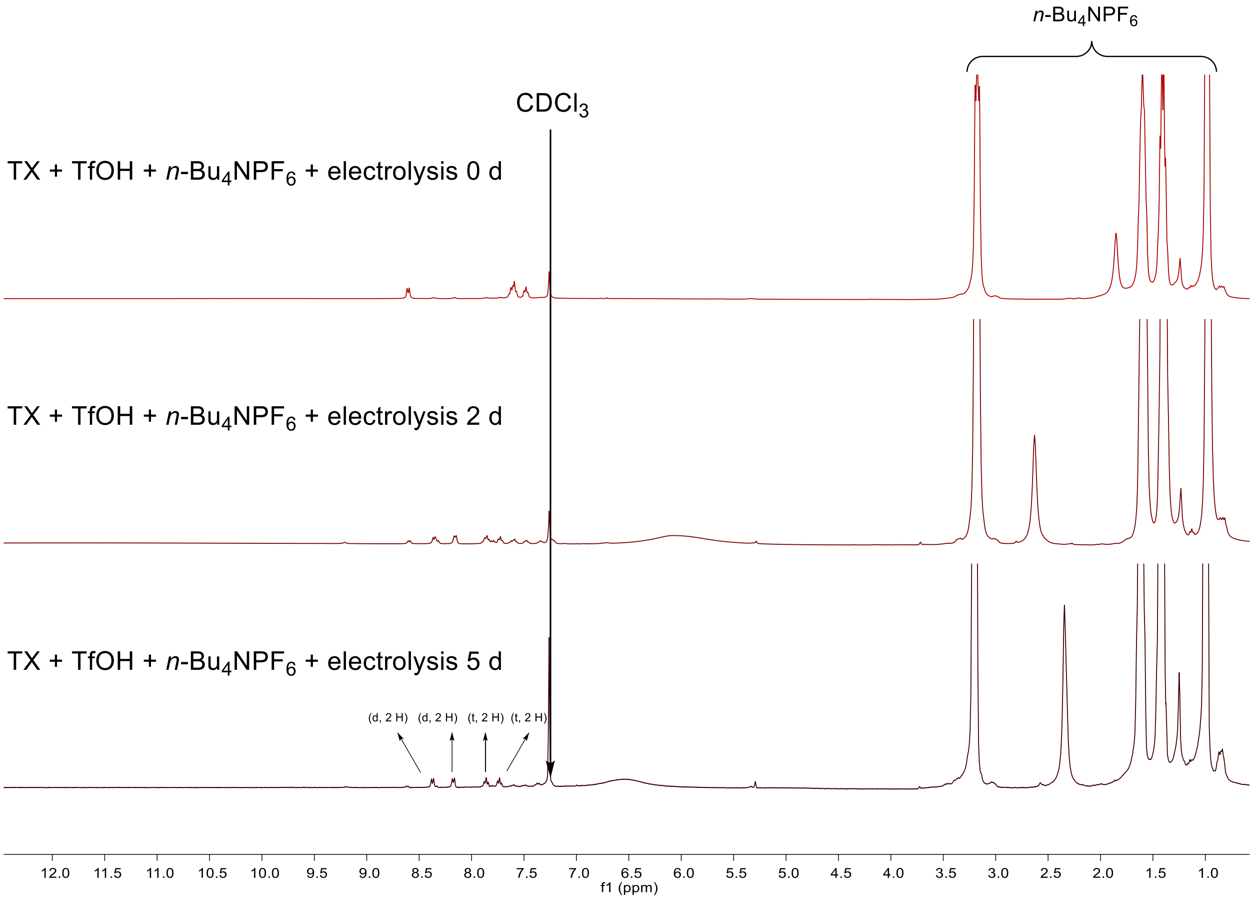

A solution of TX (0.02 mmol, 4.2 mg), TfOH (0.04 mmol, 3.5 μL), and *n*-Bu_4NPF_6 (0.14 mmol, 54.1 mg) in $CDCl_3$ (3 mL) was electrolyzed at a constant cell potential of 1.8 V (C(+)/C(-))at rt under argon atmosphere for 0-5 d.

Supplementary Figure 16. Experiment equipment for ^{1}H NMR and EPR tests

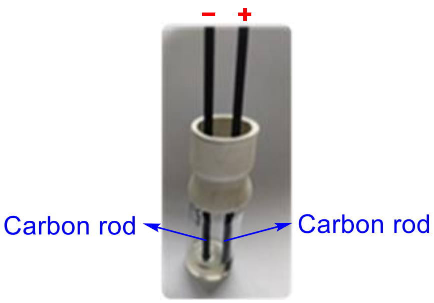

Stern-Volmer luminescence quenching experiments

The fluorescence decay profiles in nanosecond region were recorded by using time-correlated single photon counting technique (TCSPC) on an Edinburgh FLS1000 fluorescence spectrometer equipped with a picosecond pulsed diode laser. To a flame dried 10 mL of Schlenk tube were added thioxanthone (5 mM), TfOH (10 mM), *n*-Bu_4NPF_6 (0.2 M), and anhydrous DCE (10 mL) under argon atmosphere. The cell was equipped with a carbon cathode and an aluminum anode, and was sealed using a rubber septum and parafilm. The reaction mixture was electrolyzed at a constant cell potential of 1.8 V at 30 °C (maintained with three cooling fans) for 5 d. A set of the luminescence decays for the mixture solutions of 9-HTX^- with different concentrations of substrate **5** were monitored at 435 nm. Stern–Volmer plot resulting from the luminescence lifetime data[5].

Supplementary Figure 17. Stern-Volmer luminescence quenching experiments

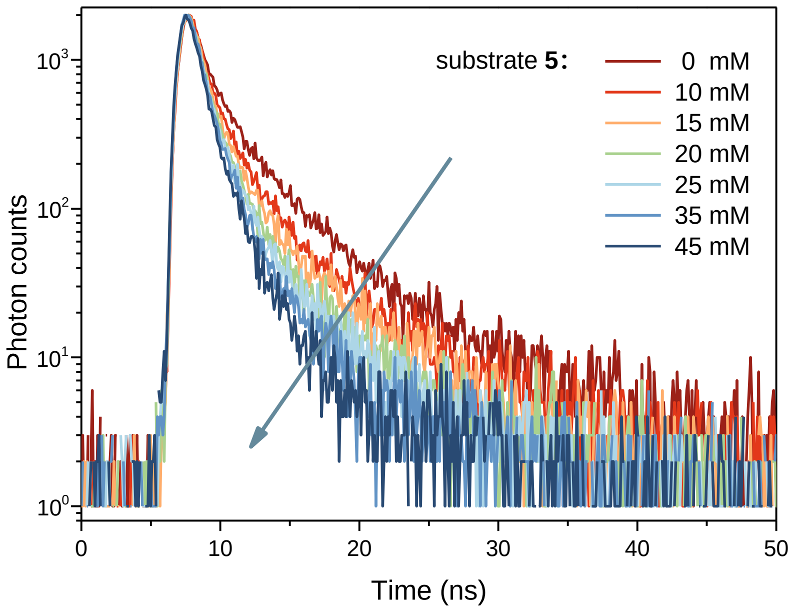

Supplementary Table 3. Stern-Volmer luminescence quenching experiments (quencher: 5)

quencher: substrate **5** (mM)	photocatalyst： 9-HTX$^-$ (0.5 mM) τ_0/τ
0	1
10	1.39241
15	1.48649
20	1.89655
25	2.07547
35	2.30769
45	2.53846

Supplementary Figure 18. Stern–Volmer plot

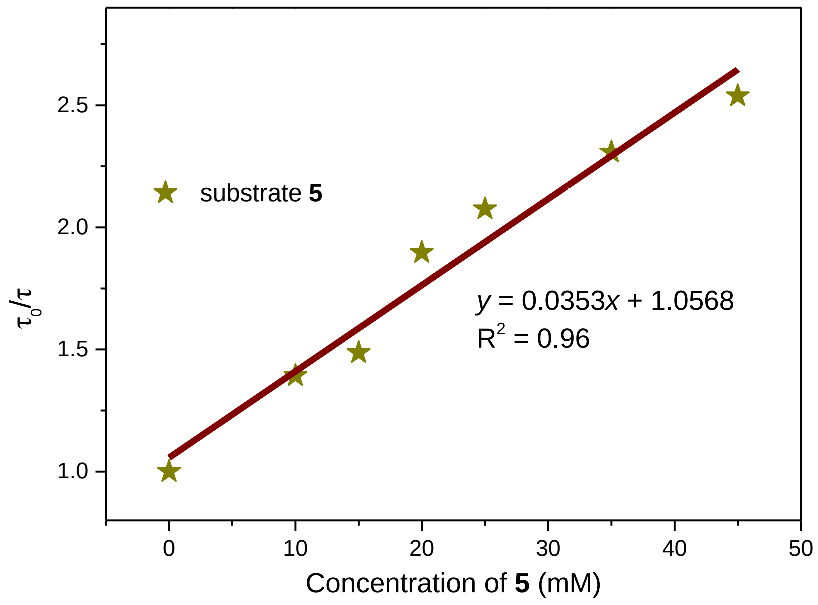

Controlled potential electrolysis

Supplementary Figure 19. Reactivity initiation via catalyst reduction[a,b]

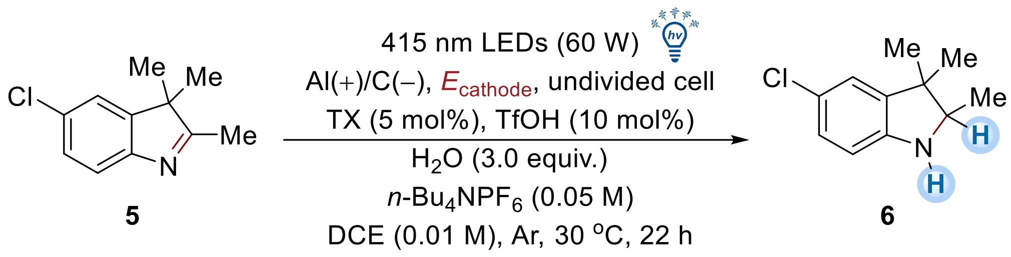

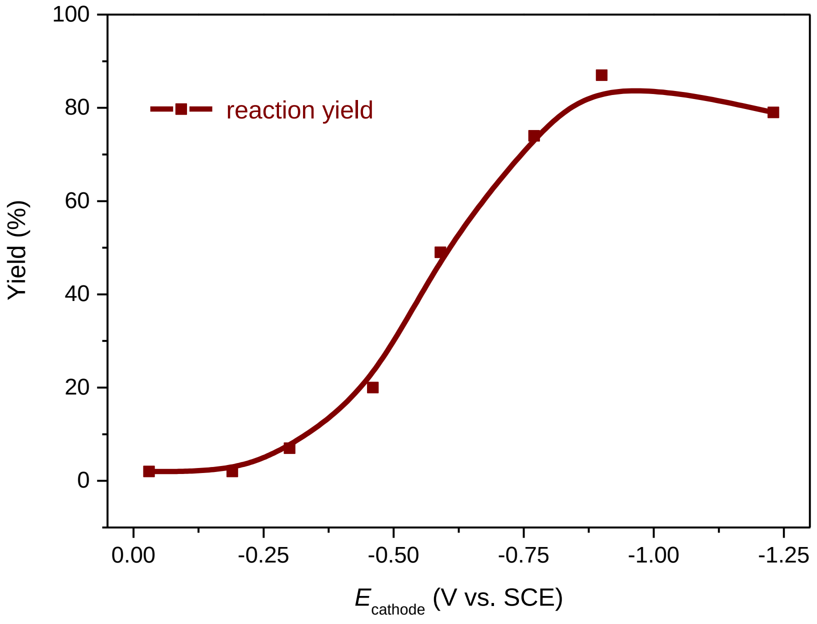

[a]In addition to operating under the controlled cathodic potential (as shown above) by using a reference electrode, the electrolysis experiments were conducted according to the typical procedure III. Yield was determined by ^{1}H NMR analysis (400 MHz) of the crude reaction mixture using CH_2Br_2 (0.2 mmol) as the internal standard. [b]Inspired by references[6,7], we carried out the electrolysis experiments.

Light on-off experiments

Supplementary Figure 20. Photo irradiation on-off experiments[a]

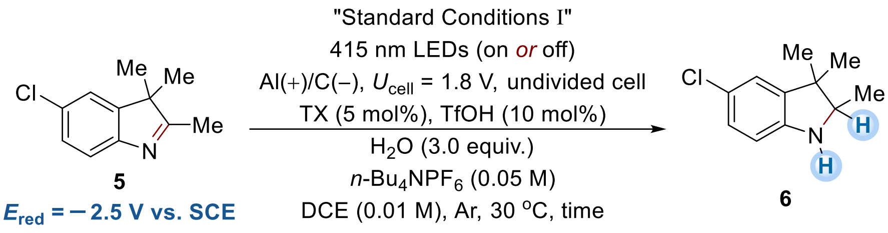

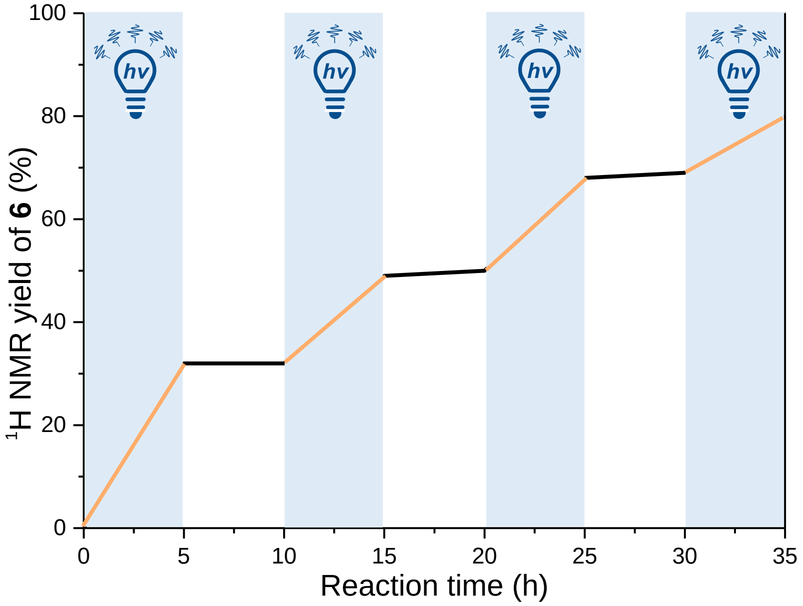

[a]The electrolysis experiments were conducted according to the typical procedure III. Light was switched off during the "OFF" periods. Yield was determined by ^{1}H NMR analysis (400 MHz) of the crude reaction mixture using CH_2Br_2 (0.2 mmol) as the internal standard.

Deuterium labeling studies

Supplementary Table 4. Deuterium labeling experiments[a]

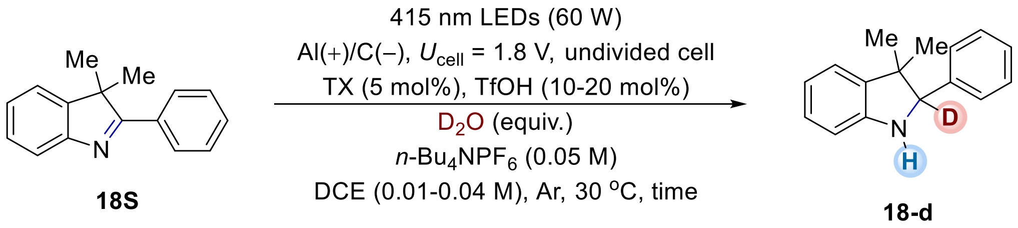

Entry	TfOH (mol%)	D_2O (equiv.)	DCE （mL）	Time (h)	Isolated yield (%)	Deuterium ratio (%)[b]
1	10	3	20	33	85	22
2	20	10	10	33	86	45
3	20	30	5	22	89	81
4	20	50	5	19	90	92

[a]Reaction conditions: **18S** (0.2 mmol), a carbon cathode and an aluminium anode, constant voltage (U = 1.8 V), thioxanthone (5 mol%), TfOH, D_2O, *n*-Bu_4NPF, anhydrous DCE, 415 nm LEDs (60 W), 30 °C, argon atmosphere, 19-33 h. [b]Deuterium ratio was determined by ^1H NMR analysis.

Faradaic Efficiency

Supplementary Figure 21. Electrophotocatalytic imine hydrogenation[a]

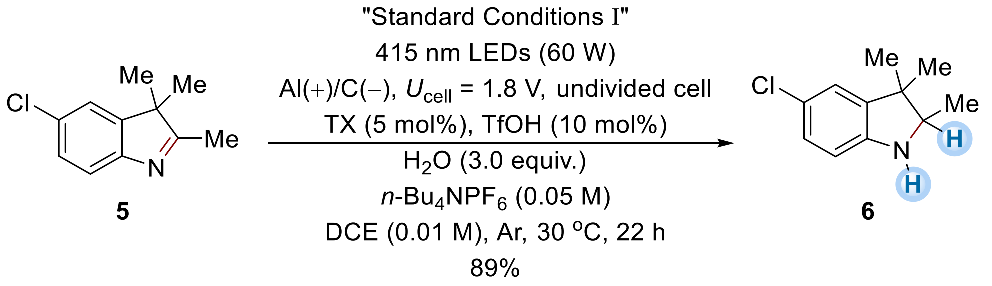

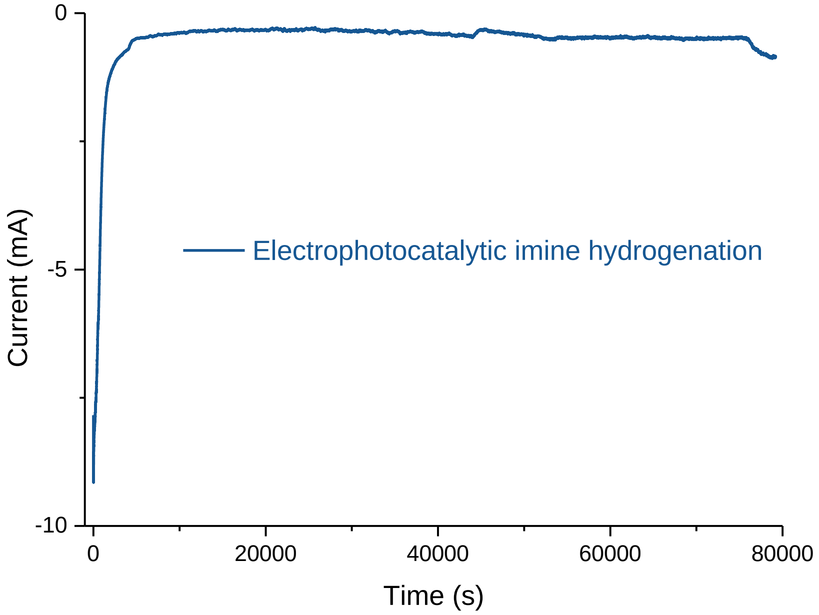

[a]The electrolysis experiments (**5**, 0.2 mmol) were conducted according to Standard Conditions I. Yield was determined by ^{1}H NMR analysis (400 MHz) of the crude reaction mixture using CH_2Br_2 (0.2 mmol) as the internal standard.

$$Q_1 = \frac{41.6\ \mathrm{C}}{0.2\ \mathrm{mmol}} = \frac{41.6}{0.2 \times 0.001 \times 96485} = 2.2\ \mathrm{F/mol}$$

$$FE_1 = \frac{0.2 \times 0.001 \times 89\% \times 2 \times 96485}{41.6} = 83\%$$

Supplementary Figure 22. Electrophotocatalytic reductive functionalization of aryl halide[a]

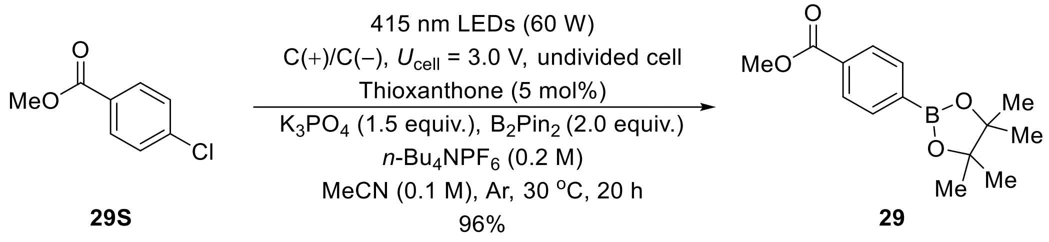

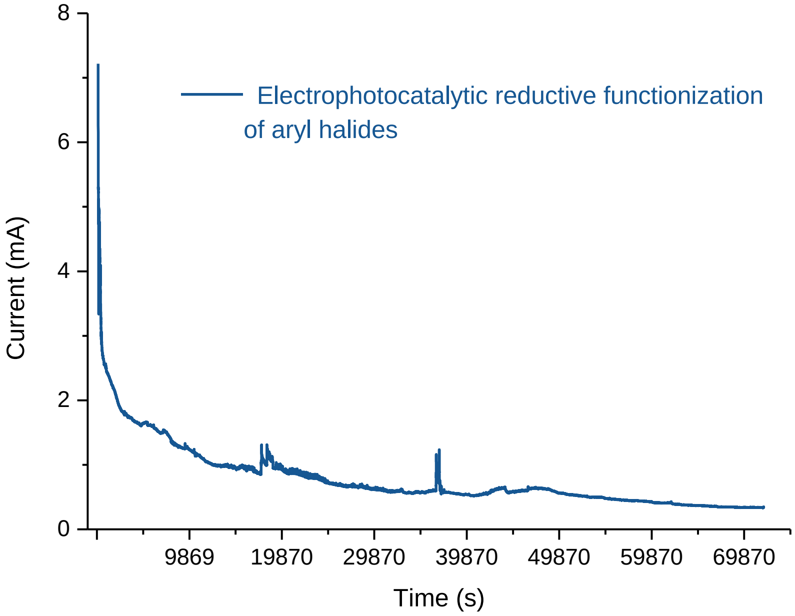

[a] The electrolysis experiments (**29S**, 0.4 mmol) were conducted according to Standard Conditions II. Yield was determined by ^{1}H NMR analysis (400 MHz) of the crude reaction mixture using CH_2Br_2 (0.2 mmol) as the internal standard.

$$Q_2 = \frac{56.4\ \text{C}}{0.4\ \text{mmol}} = \frac{56.4}{0.4 \times 0.001 \times 96485} = 1.5\ \text{F/mol}$$

$$FE_2 = \frac{0.4 \times 0.001 \times 96\% \times 96485}{56.4} = 66\%$$

Computational method

Using the Gaussian 16 software program[8], density functional theory (DFT) and time-dependent density functional theory (TD-DFT) calculations were performed with the CAM-B3LYP range-separated functional[9], the DFT-D3(BJ) dispersion correction[10], the def2-TZVP basis set[11], and the SMD implicit solvation model for dichloroethane or acetonitrile[12]. All geometries were optimized and were verified as local minimum using harmonic vibrational frequency calculations. Visualizations were carried out in PyMOL[13].

Reduction potential of 5B

We evaluated the reduction potential of **5B** by computational means using the same level of theory as described above. To eliminate computational errors, we estimated the relative potentials between **5B** and **5**. The reduction potential of **5** had been experimentally determined to be −2.5 V vs. SCE. The calculations suggested that the reduction potential of **5B** was higher than that of **5** by 0.2 V, so we estimated that $E_{\mathrm{red,}\,\mathbf{5B}} = -2.3$ V vs. SCE.

Quantum mechanical computations were carried out to probe the one-electron reduction of **5**. The calculations suggest that, in agreement with the abovementioned redox potentials (**4** and **5**), **5** undergoes an exergonic single-electron reduction by the photoexcited 9-HTX^{-}*, showing a considerable ΔG of -17.8 kcal/mol. The ground-state 9-HTX^{-}, however, requires a prohibitively high ΔG of 27.5 kcal/mol, which explains the lack of reaction in the absence of visible light, underscoring the critical role of 9-HTX^{-}* in initiating this energy-demanding reaction.

Supplementary Figure 23. One-electron reduction of 43S

The free energy for **43S** to be reduced by photoexcited catalyst TX^{2-}* is -19.3 kcal/mol:

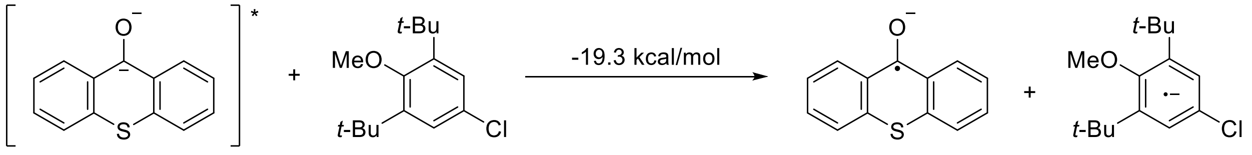

TX^{2-*} **43S** TX$^{\bullet-}$ **43S**$^{\bullet-}$

Supplementary Table 5. Absolute free energies (arb. units) of calculated structures

Species	Free Energies	Species	Free Energies
**1**	-1935.68921	TfOH	-962.21272
**2**	-1935.82526	TfO^{-}	-961.80160
**3**	-974.12421	**43S** (MeCN)	-1120.49969
**3'**	-1935.90830	**43S**$^{\bullet-}$ (MeCN)	-1120.54227
**4**	-974.05195	TX^{2-}* (MeCN)	-973.57155
**5**	-941.15789	$TX^{\bullet-}$ (MeCN)	-973.55970
**5A**	-1903.40389		
**5B**	-941.71123		
**5C**	-941.77453		
**6**	-942.34324		
**TS-1**	-1915.71694		

Experimental procedures

Typical procedure I for the synthesis of substrates

Synthesis of 5-(*tert*-butyl)-2,3,3-trimethyl-3*H*-indole (8S)

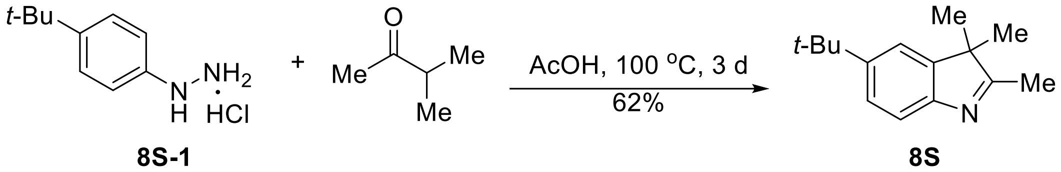

To a 120 mL of Pyrex sealed tube were added **8S-1** (2.000 g, 10.0 mmol), AcOH (10 mL), and 3-methylbutan-2-one (1.3 mL, 12.2 mmol). The resulting mixture was stirred at 100 °C with an oil bath for 3 d as monitored by TLC (petroleum ether/ethyl acetate = 10:1). After concentration, the reaction was diluted with saturated $NaHCO_3$ solution (50 mL). The aqueous layer was extracted with DCM (50 mL × 3). The combined organic layer was washed by brine and then dried over $MgSO_4$, filtered and concentrated under reduced pressure. The crude product was purified by flash chromatography on silica gel (eluent: petroleum ether/ethyl acetate = 20:1) to afford **8S**[14] as a red solid (1.3351 g, 62%). ^{1}H NMR (400 MHz, $CDCl_3$) δ 7.44 (d, J = 8.0 Hz, 1 H), 7.33 (dd, J = 8.0, 1.6 Hz, 1 H), 7.29 (d, J = 2.0 Hz, 1 H), 2.26 (s, 3 H), 1.35 (s, 9 H), 1.30 (s, 6 H).

The following compounds were synthesized according to typical procedure I.

1) 5-Fluoro-2,3,3-trimethyl-3*H*-indole (13S)

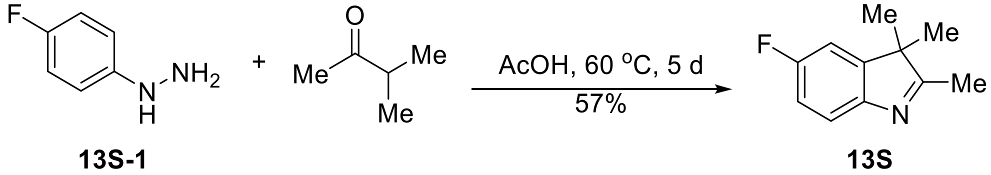

The reaction of **13S-1** (2.0 mL, 20.0 mmol), 3-methylbutan-2-one (2.1 mL, 20.0 mmol), and AcOH (50 mL) afforded **13S**[15] as a rufous solid (2.020 g, 57%). ^{1}H NMR (400 MHz, $CDCl_3$) δ 7.45 (dd, J = 8.8, 4.4 Hz, 1 H), 7.02-6.94 (m, 2 H), 2.26 (s, 3 H), 1.30 (s, 6 H).

2) 5-Cyano-2,3,3-trimethyl-3*H*-indole (14S)

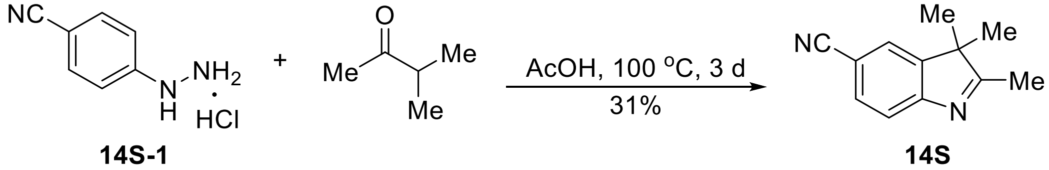

The reaction of **14S-1** (849 mg, 5.0 mmol), 3-methylbutan-2-one (652 μL, 6.0 mmol), and AcOH (4 mL) afforded **14S**[16] as a red solid (286 mg, 31%). ^{1}H NMR (400 MHz, $CDCl_3$) *δ* 7.67-7.57 (m, 2 H), 7.55 (s, 1 H), 2.33 (s, 3 H), 1.33 (s, 6 H).

3) 2,3,3,4,6-Pentamethyl-3*H*-indole (16S)

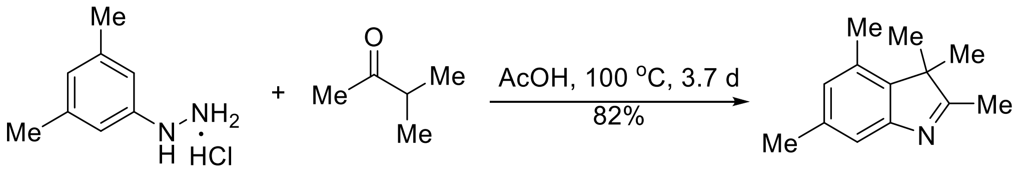

16S-1 **16S**

The reaction of **16S-1** (3.470 g, 20.0 mmol), 3-methylbutan-2-one (2.6 mL, 24.0 mmol), and AcOH (20 mL) afforded **16S**[17] as an orange oil (3.090 g, 82%). ^{1}H NMR (400 MHz, $CDCl_3$) *δ* 7.18 (s, 1 H), 6.78 (s, 1 H), 2.40 (s, 3 H), 2.35 (s, 3 H), 2.24 (s, 3 H), 1.36 (s, 6 H).

4) 4,6-Dichloro-2,3,3-trimethyl-3*H*-indole (17S)

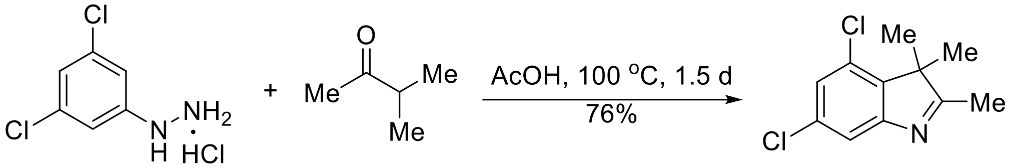

17S-1 **17S**

The reaction of **17S-1** (4.270 g, 20.0 mmol), 3-methylbutan-2-one (2.1 mL, 20.0 mmol), and AcOH (50 mL) afforded **17S**[18] as a yellow solid (3.466 g, 76%). ^{1}H NMR (400 MHz, $CDCl_3$) *δ* 7.41 (d, *J* = 1.6 Hz, 1 H), 7.14 (d, *J* = 2.0 Hz, 1 H), 2.27 (s, 3 H), 1.43 (s, 6 H).

5) 2-Phenyl-3,3-dimethyl-3*H*-indole (18S)

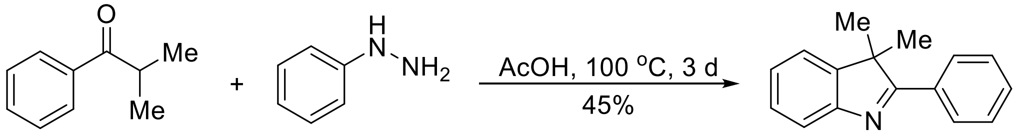

18S-1 **18S**

The reaction of **18S-1** (1.8 mL, 12.0 mmol), phenylhydrazine (1.0 mL, 10.0 mmol), and AcOH (10 mL) afforded **18S**[15] as a white solid (996 mg, 45%). ^{1}H NMR (400 MHz, $CDCl_3$) *δ* 8.18-8.11 (m, 2 H), 7.72-7.67 (m, 1 H), 7.51-7.45 (m, 3 H), 7.40-7.31 (m, 2 H), 7.31-7.23 (m, 1 H), 1.59 (s, 6 H).

6) 2'-Phenylspiro[cyclohexane-1,3'-indole] (19S)

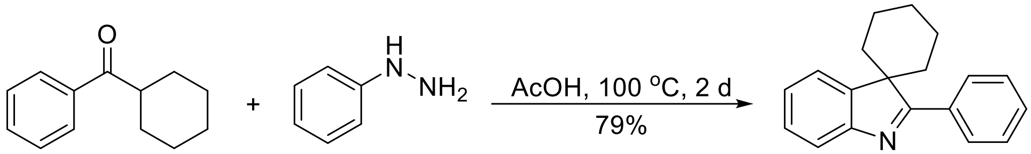

19S-1 **19S**

The reaction of **19S-1** (3.766 g, 20.0 mmol), phenylhydrazine (2.0 mL, 20.0 mmol), and AcOH (50 mL) afforded **19S**[15] as a yellow oil (4.126 g, 79%). ^{1}H NMR (400 MHz, $CDCl_3$) δ 8.07-8.01 (m, 2 H), 7.82 (d, J = 7.2 Hz, 1 H), 7.72 (d, J = 7.6 Hz, 1 H), 7.49-7.43 (m, 3 H), 7.39 (t, J = 7.6 Hz, 1 H), 7.22 (t, J = 7.2 Hz, 1 H), 2.28 (td, J = 13.2, 4.4 Hz, 2 H), 2.05-1.97 (m, 3 H), 1.86-1.78 (m, 2 H), 1.55-1.39 (m, 3 H).

7) 2'-Methylspiro[cyclopentane-1,3'-indole] (20S)

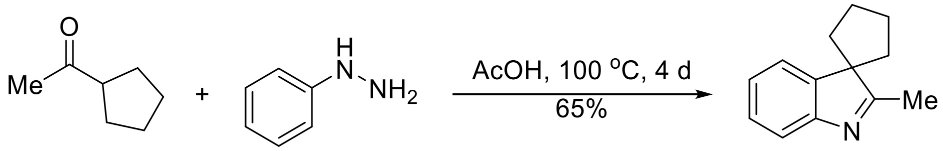

20S-1 **20S**

The reaction of **20S-1** (2.5 mL, 20.0 mmol), phenylhydrazine (2.0 mL, 20.0 mmol), and AcOH (50 mL) afforded **20S**[15] as a red liquid (2.409 g, 65%). ^{1}H NMR (400 MHz, $CDCl_3$) δ 7.51 (d, J = 7.6 Hz, 1 H), 7.30 (t, J = 6.8 Hz, 2 H), 7.16 (t, J = 7.2 Hz, 1 H), 2.29 (s, 3 H), 2.12-1.96 (m, 6 H), 1.83-1.75 (m, 2 H).

8) 2'-Methylspiro[cyclohexane-1,3'-indole] (21S)

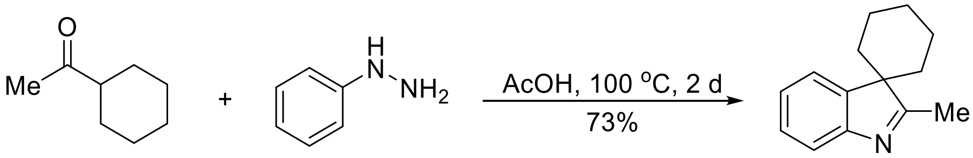

21S-1 **21S**

The reaction of **21S-1** (2.8 mL, 20.0 mmol), phenylhydrazine (2.0 mL, 20.0 mmol), and AcOH (50 mL) afforded **21S**[15] as a red solid (2.903 g, 73%). ^{1}H NMR (400 MHz, $CDCl_3$) δ 7.71 (d, J = 7.6 Hz, 1 H), 7.56 (d, J = 7.6 Hz, 1 H), 7.33 (t, J = 7.6 Hz, 1 H), 7.16 (t, J = 6.8 Hz, 1 H), 2.28 (s, 3 H), 2.01-1.87 (m, 3 H), 1.84-1.74 (m, 4 H), 1.54-1.42 (m, 1 H), 1.33-1.26 (m, 2 H).

9) 2'-Methylspiro[2,3,5,6-tetrahydropyran-4,3'-indole] (22)

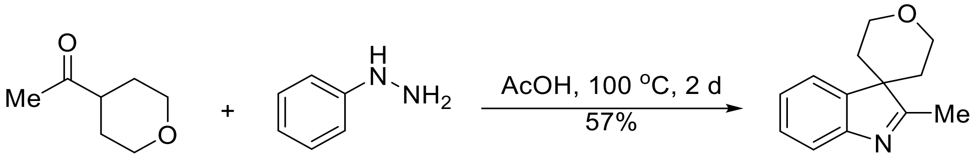

The reaction of **22S-1** (2.5 mL, 20.0 mmol), phenylhydrazine (2.0 mL, 20.0 mmol), and AcOH (50 mL) afforded **22S**[15] as a yellow solid (2.295 g, 57%). ^{1}H NMR (400 MHz, $CDCl_3$) δ 7.83 (d, J = 7.2 Hz, 1 H), 7.58 (d, J = 7.6 Hz, 1 H), 7.37 (t, J = 7.6 Hz, 1 H), 7.20 (t, J = 7.6 Hz, 1 H), 4.17-4.01 (m, 4 H), 2.33 (s, 3 H), 2.25-2.13 (m, 2 H), 1.30-1.22 (m, 2 H).

Typical procedure II for the synthesis of substrates

Synthesis of 5-(naphthalen-2-yl)-2,3,3-trimethyl-3*H*-indole (24S)

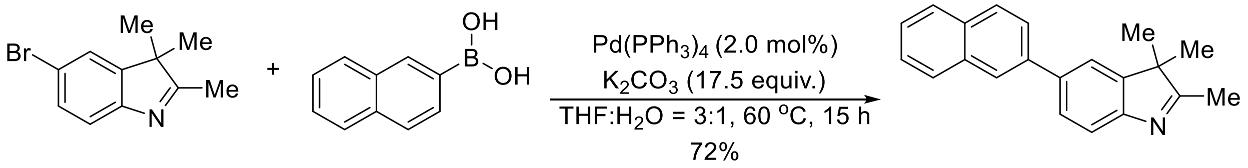

To a flame dried 250 mL of Schlenk flask were added **24S-1** (2.382 g, 10.0 mmol), naphthalen-2-ylboronic acid (2.581 g, 15.0 mmol), $Pd(PPh_3)_4$ (234.0 mg, 0.2 mmol), K_2CO_3 (24.167 g, 175.0 mmol), anhydrous THF (105 mL) and H_2O (35 mL) under argon atmosphere. The resulting mixture was stirred at 60 °C with an oil bath for 15 h as monitored by TLC (petroleum ether/ethyl acetate = 4:1). After concentration, the reaction was diluted with saturated NH_4Cl solution (50 mL). The aqueous layer was extracted with ethyl acetate (50 mL × 3). The combined organic layer was washed by brine and then dried over $MgSO_4$, filtered and concentrated under reduced pressure. The crude product was purified by flash chromatography on silica gel (eluent: petroleum ether/ethyl acetate = 5:1) to afford **24S** as a yellow solid (2.052 g, 72%). Mp: 153.4-154.4 °C (petroleum ether/ethyl acetate). ^{1}H NMR (400 MHz, $CDCl_3$) δ 8.05 (s, 1 H), 7.95-7.84 (m, 3 H), 7.77 (dd, J = 8.4, 2.0 Hz, 1 H), 7.70-7.60 (m, 3 H), 7.55-7.44 (m, 2 H), 2.32 (s, 3 H), 1.39 (s, 6 H); ^{13}C NMR (101 MHz, $CDCl_3$) δ 188.3, 153.3, 146.4, 138.7, 138.3, 133.7, 132.5, 128.4, 128.1, 127.6, 127.0, 126.3, 125.8, 125.71, 125.68, 120.4, 120.1, 53.8, 23.2, 15.5; IR (neat) 1598, 1578, 1481, 1458, 1427 cm^{-1}; HRMS (ESI): calcd for $C_{21}H_{20}N^+$ [M+H]$^+$: 286.1590, found: 286.1588.

The following compounds were synthesized according to typical procedure II.

1) 5-Phenyl-2,3,3-trimethyl-3*H*-indole (10S)

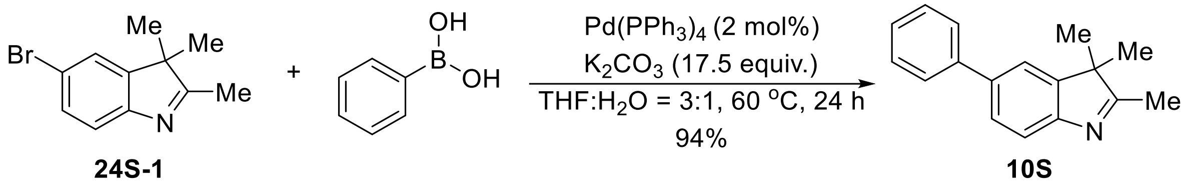

The reaction of **24S-1** (4.286 g, 18.0 mmol), phenylboronic acid (3.293 g, 27.0 mmol), $Pd(PPh_3)_4$ (416.0 mg, 0.36 mmol), K_2CO_3 (40.132 g, 315.0 mmol), anhydrous THF (225 mL) and H_2O (75 mL) afforded **10S**[19] as a yellow solid (3.968 g, 94%). ^{1}H NMR (400 MHz, $CDCl_3$) δ 7.64-7.56 (m, 3 H), 7.53 (dd, *J* = 7.6, 1.6 Hz, 1 H), 7.48 (d, *J* = 1.6 Hz, 1 H), 7.44 (t, *J* = 7.6 Hz, 2 H), 7.33 (t, *J* = 7.2 Hz, 1 H), 2.30 (s, 3 H), 1.35 (s, 6 H).

2) 5-(Thiophen-3-yl)-2,3,3-trimethyl-3*H*-indole (23S)

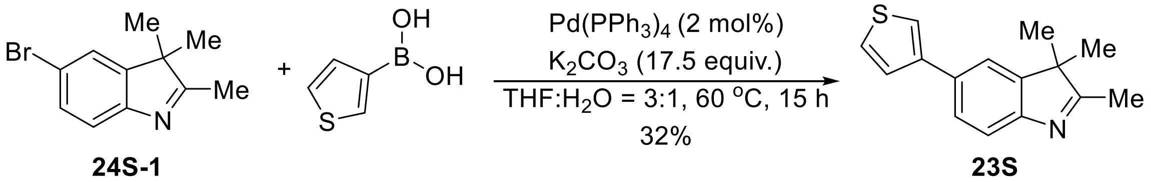

The reaction of **24S-1** (2.382 g, 10.0 mmol), thiophen-3-ylboronic acid (1.919 g, 15.0 mmol), $Pd(PPh_3)_4$ (234.1 mg, 0.2 mmol), K_2CO_3 (24.165 g, 175.0 mmol), anhydrous THF (105 mL) and H_2O (35 mL) afforded **23S** as a black solid (773.8 mg, 32%). Mp: 101.8-102.6 °C (petroleum ether/ethyl acetate). ^{1}H NMR (400 MHz, $CDCl_3$) δ 7.56-7.51 (m, 2 H), 7.48 (s, 1 H), 7.43-7.40 (m, 1 H), 7.39-7.32 (m, 2 H), 2.28 (s, 3 H), 1.32 (s, 6 H); ^{13}C NMR (101 MHz, $CDCl_3$) δ 188.1, 153.0, 146.4, 142.5, 133.1, 126.5, 126.2, 126.0, 120.1, 119.9, 119.6, 53.7, 23.2, 15.5; IR (neat) 1615, 1572, 1461, 1429 cm^{-1}; HRMS (ESI): calcd for $C_{15}H_{16}NS^+$ $[M+H]^+$: 242.0998, found: 242.0990.

Synthesis of 1,3-di-*tert*-butyl-5-chloro-2-methoxybenzene (43S)[20]

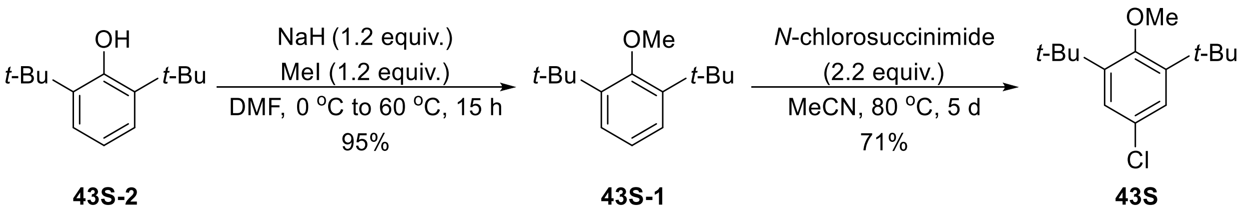

To a flame dried 25 mL of Pyrex sealed tube were added **43S-2** (5.132 g, 24.9 mmol) and anhydrous DMF (25 mL) under argon atmosphere. The mixture was cooled to 0 °C and NaH (60% wt. in mineral oil, 1.202 g, 30.0 mmol) was added. The mixture was cooled to 0 °C for 3 h and

MeI (1.9 mL, 30.0 mmol) was added subsequently. The mixture was stirred at 60 °C for 12 h, and then treated with H_2O (50 mL) and Et_3N (2 mL). The aqueous layer was extracted with diethyl ether (40 mL × 3). The combined organic layer was washed with water and brine, and then dried over $MgSO_4$, filtered and concentrated under reduced pressure to afford **43S-1** as a colorless oil (5.248 g, 95%). The crude product was directly subjected to subsequent transformation without further purification.

To a flame dried 100 mL of Schlenk flask were added *N*-chlorosuccinimide (2.497 g, 18.7 mmol), anhydrous MeCN (40 mL), and crude **43S-1** (1.873 g, 8.5 mmol) under argon atmosphere. The resulting mixture was stirred at 80 °C for 5 d. After concentration, hexane (40 mL) was added and the precipitated succinimide was filtered. The crude product was purified by flash chromatography on silica gel (eluent: petroleum ether) to afford **43S**[20] as a colorless oil (1.538 g, 71%). ^1H NMR (400 MHz, $CDCl_3$) δ 7.21 (s, 2 H), 3.69 (s, 3 H), 1.42 (s, 18 H).

Typical procedure III for the electrophotocatalytic imine hydrogenation

Synthesis of 5-chloro-2,3,3-trimethylindoline (6)

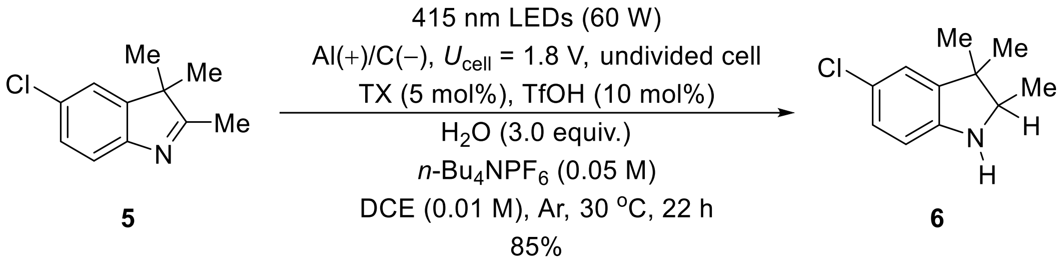

An undivided cell was prepared and equipped with a stir bar. To a flame dried 25 mL of Schlenk tube were added thioxanthone (2.1 mg, 0.01 mmol), *n*-Bu_4NPF_6 (387.4 mg, 1.0 mmol), anhydrous DCE (20 mL), **5** (38.7 mg, 0.2 mmol), H_2O (11.0 μL, 0.6 mmol), and TfOH (2.0 μL, 0.02 mmol) under argon atmosphere. The cell was equipped with a carbon cathode and an aluminium anode (note: aluminium needs to be activated with HCl (3 M in H_2O) on site), and was sealed using a rubber septum and parafilm. The reaction mixture was electrolyzed at a constant cell potential of 1.8 V under irradiation of 415 nm LEDs (60 W) at 30 °C (maintained with four cooling fans). The reaction was completed after 22 h as monitored by TLC (petroleum ether/ethyl acetate = 20:1). The crude product was collected by washing chamber and electrodes with EtOAc (10 mL × 3) in an ultrasonic bath. The solvent was then removed and the residue was purified by flash chromatography on silica gel (eluent: petroleum ether/ethyl acetate = 20:1) to afford **6**[21] as a yellow

oil (33.3 mg, 85%). ^{1}H NMR (400 MHz, $CDCl_3$) δ 6.98-6.92 (m, 2 H), 6.51 (d, J = 8.8 Hz, 1 H), 3.75 (brs, 1 H), 3.52 (q, J = 6.4 Hz, 1 H), 1.26 (s, 3 H), 1.16 (d, J = 6.4 Hz, 3 H), 1.03 (s, 3 H).

The following compounds were synthesized according to typical procedure III.

1) 5-Methoxy-2,3,3-trimethylindoline (7)

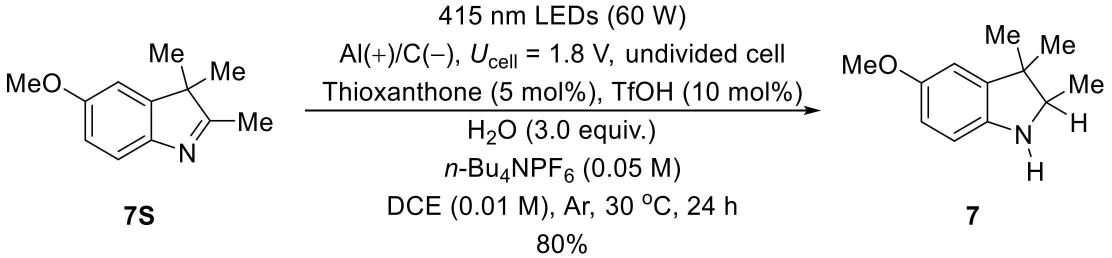

The reaction of **7S** (37.9 mg, 0.2 mmol), thioxanthone (2.1 mg, 0.01 mmol), TfOH (1.8 μL, 0.02 mmol), H_2O (10.8 μL, 0.6 mmol), *n*-Bu_4NPF_6 (387.4 mg, 1.0 mmol), and anhydrous DCE (20 mL) afforded **7**[21] as a yellow oil (30.6 mg, 80%). ^{1}H NMR (400 MHz, $CDCl_3$) δ 6.65 (s, 1 H), 6.62-6.53 (m, 2 H), 3.75 (s, 3 H), 3.49 (q, J = 6.4 Hz, 1 H), 1.27 (s, 3 H), 1.18 (d, J = 6.4 Hz, 3 H), 1.03 (s, 3 H).

2) 5-(*Tert*-butyl)-2,3,3-trimethylindoline (8)

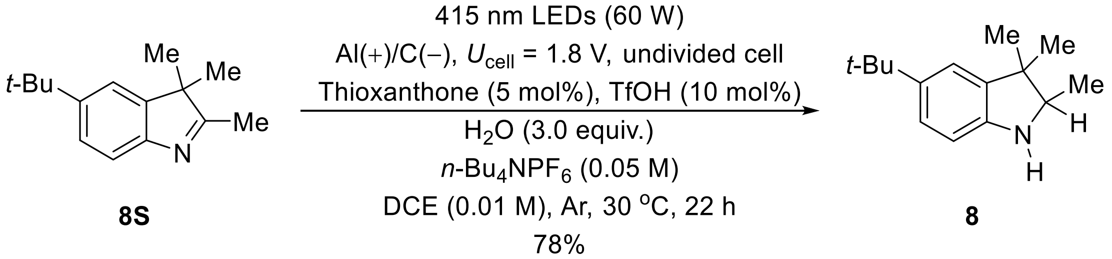

The reaction of **8S** (43.2 mg, 0.2 mmol), thioxanthone (2.1 mg, 0.01 mmol), TfOH (1.8 μL, 0.02 mmol), H_2O (10.8 μL, 0.6 mmol), *n*-Bu_4NPF_6 (387.4 mg, 1.0 mmol), and anhydrous DCE (20 mL) afforded **8** as a yellow oil (33.7 mg, 78%). ^{1}H NMR (400 MHz, $CDCl_3$) δ 7.07-7.01 (m, 2 H), 6.56 (d, J = 8.0 Hz, 1 H), 3.51 (q, J = 6.4 Hz, 1 H), 1.31-1.27 (m, 12 H), 1.18 (d, J = 6.4 Hz, 3 H), 1.03 (s, 3 H); ^{13}C NMR (101 MHz, $CDCl_3$) δ 146.9, 142.0, 139.0, 123.7, 119.2, 108.9, 65.4, 43.5, 34.3, 31.8, 26.0, 22.4, 15.0; IR (neat) 3360, 1618, 1493, 1462 cm^{-1}; HRMS (EI): calcd for $C_{15}H_{23}N^+$ $[M]^+$: 217.1825, found: 217.1825.

3) 2,3,3,5-Tetramethylindoline (9)

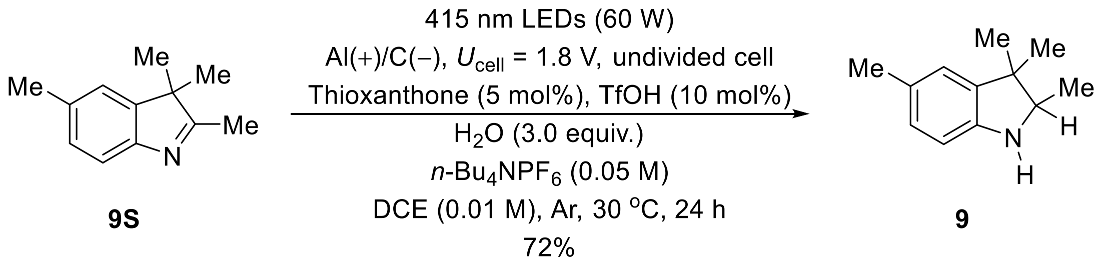

The reaction of **9S** (35.4 μL, 0.2 mmol), thioxanthone (2.1 mg, 0.01 mmol), TfOH (1.8 μL, 0.02 mmol), H_2O (10.8 μL, 0.6 mmol), n-Bu_4NPF_6 (387.3 mg, 1.0 mmol), and anhydrous DCE (20 mL) afforded **9**[21] as a yellow oil (25.2 mg, 72%). ^{1}H NMR (400 MHz, $CDCl_3$) δ 6.87-6.80 (m, 2 H), 6.54 (d, J = 7.6 Hz, 1 H), 3.49 (q, J = 6.4 Hz, 1 H), 2.27 (s, 3 H), 1.27 (s, 3 H), 1.17 (d, J = 6.4 Hz, 3 H), 1.03 (s, 3 H).

4) 5-Phenyl-2,3,3-trimethylindoline (10)

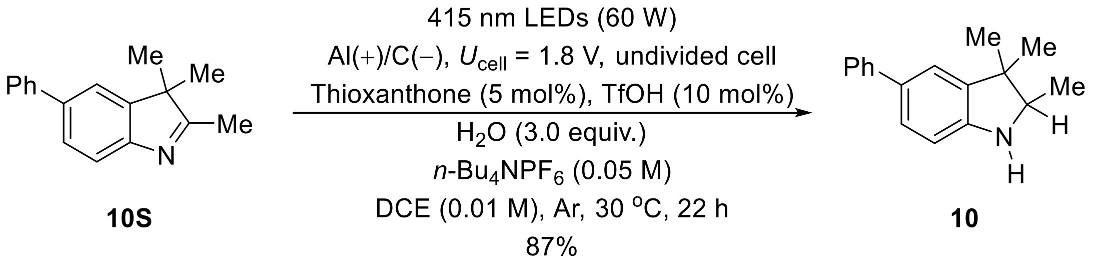

The reaction of **10S** (47.0 mg, 0.2 mmol), thioxanthone (2.1 mg, 0.01 mmol), TfOH (1.8 μL, 0.02 mmol), H_2O (10.8 μL, 0.6 mmol), n-Bu_4NPF_6 (387.3 mg, 1.0 mmol), and anhydrous DCE (20 mL) afforded **10** as a yellow oil (41.3 mg, 87%). ^{1}H NMR (400 MHz, $CDCl_3$) δ 7.54 (d, J = 7.2 Hz, 2 H), 7.39 (t, J = 7.2 Hz, 2 H), 7.30-7.21 (m, 3 H), 6.68 (d, J = 8.4 Hz, 1 H), 3.79 (brs, 1 H), 3.57 (q, J = 6.4 Hz, 1 H), 1.33 (s, 3 H), 1.20 (d, J = 6.8 Hz, 3 H), 1.09 (s, 3 H); ^{13}C NMR (101 MHz, $CDCl_3$) δ 148.8, 141.9, 139.7, 132.1, 128.5, 126.5, 126.2, 126.0, 121.1, 109.4, 65.4, 43.4, 26.2, 22.4, 15.1; IR (neat) 3371, 1615, 1541, 1507, 1481, 1456 cm^{-1}; HRMS (ESI): calcd for $C_{17}H_{20}N^+$ [M+H]$^+$: 238.1590, found: 238.1589.

5) 2,3,3-Trimethylindoline (11)

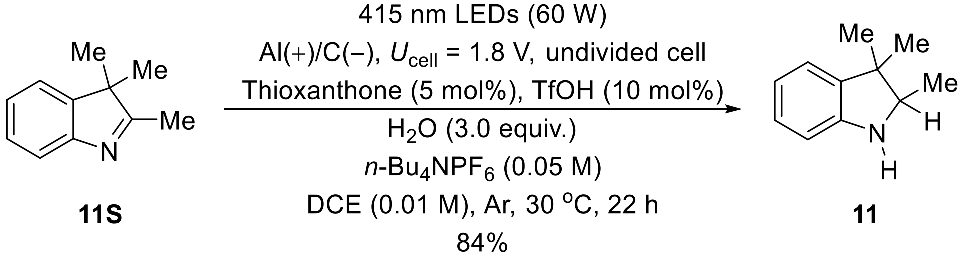

The reaction of **11S** (31.8 mg, 0.2 mmol), thioxanthone (2.1 mg, 0.01 mmol), TfOH (1.8 μL, 0.02 mmol), H_2O (10.8 μL, 0.6 mmol), *n*-Bu_4NPF_6 (387.3 mg, 1.0 mmol), and anhydrous DCE (20 mL) afforded **11**[22] as a colorless oil (27.1 mg, 84%). ^1H NMR (400 MHz, $CDCl_3$) δ 7.02 (t, *J* = 8.0 Hz, 2 H), 6.74 (t, *J* = 8.0 Hz, 1 H), 6.62 (d, *J* = 7.6 Hz, 1 H), 3.72 (brs, 1 H), 3.52 (q, *J* = 6.4 Hz, 1 H), 1.28 (s, 3 H), 1.18 (d, *J* = 6.4 Hz, 3 H), 1.04 (s, 3 H).

6) 5-Bromo-2,3,3-trimethylindoline (12)

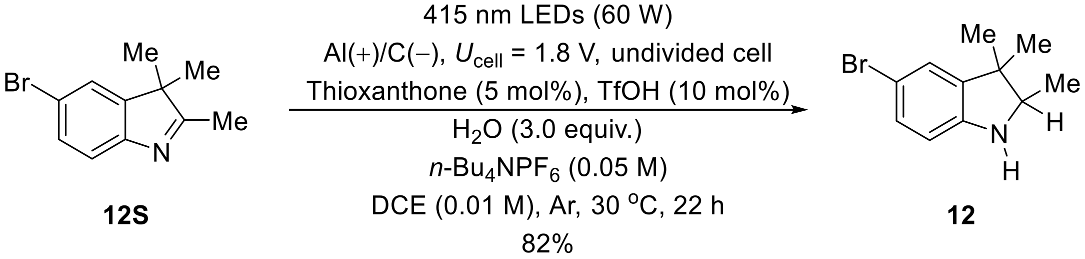

The reaction of **12S** (47.6 mg, 0.2 mmol), thioxanthone (2.1 mg, 0.01 mmol), TfOH (1.8 μL, 0.02 mmol), H_2O (10.8 μL, 0.6 mmol), *n*-Bu_4NPF_6 (387.3 mg, 1.0 mmol), and anhydrous DCE (20 mL) afforded **12**[21] as a yellow oil (39.4 mg, 82%). ^1H NMR (400 MHz, $CDCl_3$) δ 7.12-7.06 (m, 2 H), 6.47 (d, *J* = 8.8 Hz, 1 H), 3.74 (brs, 1 H), 3.52 (q, *J* = 6.4 Hz, 1 H), 1.26 (s, 3 H), 1.16 (d, *J* = 6.8 Hz, 3 H), 1.03 (s, 3 H).

7) 5-Fluoro-2,3,3-trimethylindoline (13)

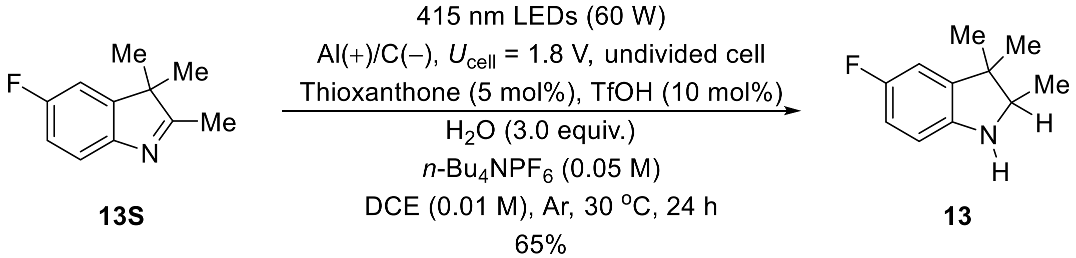

The reaction of **13S** (35.4 mg, 0.2 mmol), thioxanthone (2.1 mg, 0.01 mmol), TfOH (1.8 μL, 0.02 mmol), H_2O (11.0 μL, 0.6 mmol), *n*-Bu_4NPF_6 (387.3 mg, 1.0 mmol), and anhydrous DCE (20 mL)

afforded **13**[21] as a yellow oil (23.3 mg, 65%). ^{1}H NMR (400 MHz, $CDCl_3$) δ 6.77-6.65 (m, 2 H), 6.52 (dd, *J* = 8.4, 4.4 Hz, 1 H), 3.52 (q, *J* = 6.4 Hz, 1 H), 1.26 (s, 3 H), 1.17 (d, *J* = 6.8 Hz, 3 H), 1.03 (s, 3 H).

8) 5-Cyano-2,3,3-trimethylindoline (14)

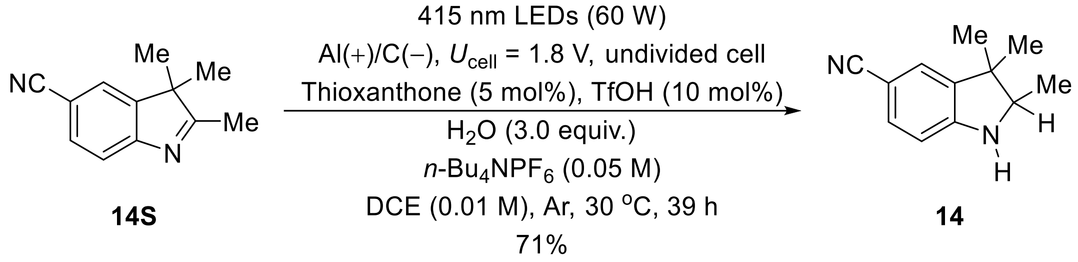

The reaction of **14S** (36.8 mg, 0.2 mmol), thioxanthone (2.1 mg, 0.01 mmol), TfOH (1.8 μL, 0.02 mmol), H_2O (10.8 μL, 0.6 mmol), *n*-Bu_4NPF_6 (387.5 mg, 1.0 mmol), and anhydrous DCE (20 mL) afforded **14** as a yellow oil (26.4 mg, 71%). ^{1}H NMR (400 MHz, $CDCl_3$) δ 7.29 (d, *J* = 8.0 Hz, 1 H), 7.20 (s, 1 H), 6.53 (d, *J* = 8.4 Hz, 1 H), 4.27 (brs, 1 H), 3.61 (q, *J* = 6.4 Hz, 1 H), 1.28 (s, 3 H), 1.18 (d, *J* = 6.0 Hz, 3 H), 1.07 (s, 3 H); ^{13}C NMR (101 MHz, $CDCl_3$) δ 152.9, 139.2, 132.8, 125.9, 120.8, 108.2, 99.7, 65.1, 43.1, 26.4, 22.3, 15.1; IR (neat) 3357, 2213, 1615, 1490 cm^{-1}; HRMS (ESI): calcd for $C_{12}H_{15}N_2^+$ $[M+H]^+$: 187.1230, found: 187.1228.

9) 5-Trifluoromethyl-2,3,3-trimethylindoline (15)

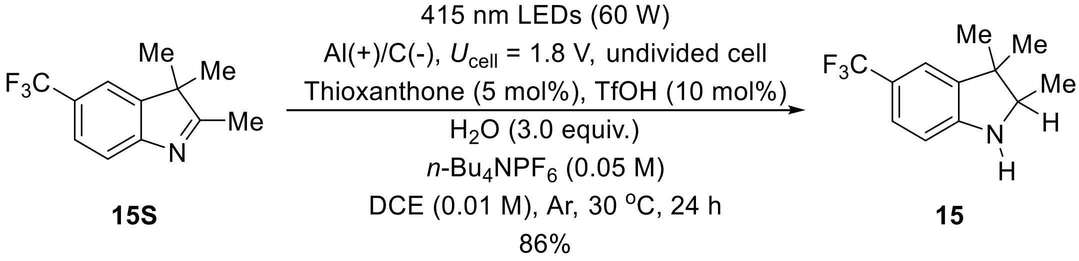

The reaction of **15S** (45.4 mg, 0.2 mmol), thioxanthone (2.1 mg, 0.01 mmol), TfOH (1.8 μL, 0.02 mmol), H_2O (10.8 μL, 0.6 mmol), *n*-Bu_4NPF_6 (387.6 mg, 1.0 mmol), and anhydrous DCE (20 mL) afforded **15**[21] as a yellow oil (39.4 mg, 86%). ^{1}H NMR (400 MHz, $CDCl_3$) δ 7.26 (d, *J* = 8.0 Hz, 1 H), 7.21 (s, 1 H), 6.58 (d, *J* = 8.0 Hz, 1 H), 4.01 (brs, 1 H), 3.58 (q, *J* = 6.4 Hz, 1 H), 1.29 (s, 3 H), 1.18 (d, *J* = 6.4 Hz, 3 H), 1.07 (s, 3 H).

10) 2,3,3,4,6-Pentamethylindoline (16)

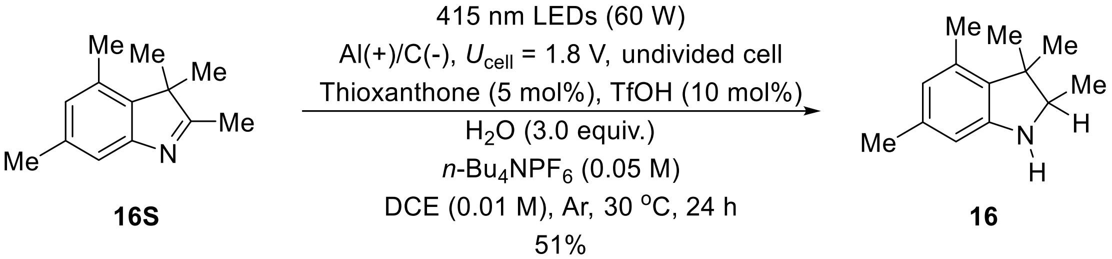

The reaction of **16S** (37.6 mg, 0.2 mmol), thioxanthone (2.1 mg, 0.01 mmol), TfOH (1.8 μL, 0.02 mmol), H_2O (10.8 μL, 0.6 mmol), *n*-Bu_4NPF_6 (387.6 mg, 1.0 mmol), and anhydrous DCE (20 mL) afforded **16** as a yellow oil (19.3 mg, 51%). ^{1}H NMR (400 MHz, $CDCl_3$) δ 6.35-6.30 (m, 2 H), 3.63 (brs, 1 H), 3.43 (q, J = 6.4 Hz, 1 H), 2.29 (s, 3 H), 2.21 (s, 3 H), 1.34 (s, 3 H), 1.16 (d, J = 6.4 Hz, 3 H), 1.06 (s, 3 H); ^{13}C NMR (101 MHz, $CDCl_3$) δ 149.9, 137.0, 133.8, 132.6, 122.6, 108.3, 65.0, 44.0, 25.8, 21.2, 19.9, 18.5, 14.5; IR (neat) 3363, 1615, 1590, 1462 cm^{-1}; HRMS (ESI): calcd for $C_{13}H_{20}N^+$ $[M+H]^+$: 190.1590, found: 190.1591.

11) 4,6-Dichloro-2,3,3-trimethylindoline (17)

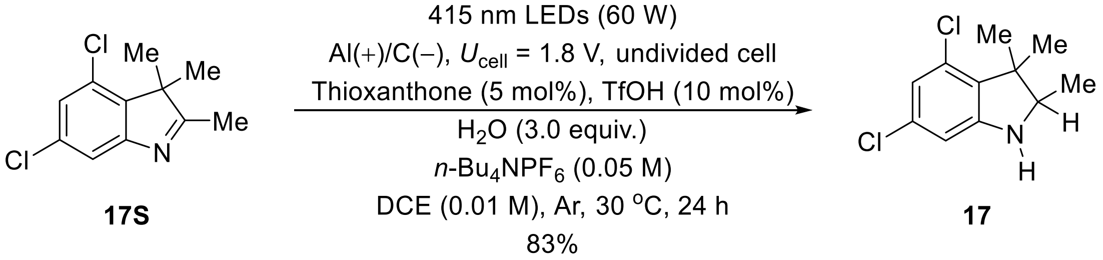

The reaction of **17S** (45.6 mg, 0.2 mmol), thioxanthone (2.1 mg, 0.01 mmol), TfOH (1.8 μL, 0.02 mmol), H_2O (10.8 μL, 0.6 mmol), *n*-Bu_4NPF_6 (387.5 mg, 1.0 mmol), and anhydrous DCE (20 mL) afforded **17** as a yellow oil (38.2 mg, 83%). ^{1}H NMR (400 MHz, $CDCl_3$) δ 6.63 (d, J = 1.6 Hz, 1 H), 6.44 (d, J = 1.6 Hz, 1 H), 3.93 (brs, 1 H), 3.52 (q, J = 6.4 Hz, 1 H), 1.42 (s, 3 H), 1.17 (d, J = 6.4 Hz, 3 H), 1.13 (s, 3 H); ^{13}C NMR (101 MHz, $CDCl_3$) δ 152.0, 133.3, 132.5, 130.9, 119.3, 107.7, 65.2, 44.9, 25.2, 19.4, 14.6; IR (neat) 3397, 1598, 1575, 1470, 1430 cm^{-1}; HRMS (EI): calcd for $C_{11}H_{13}Cl_2N^+$ $[M]^+$: 229.0420, found: 229.0420.

12) 2-Phenyl-3,3-dimethylindoline (18)

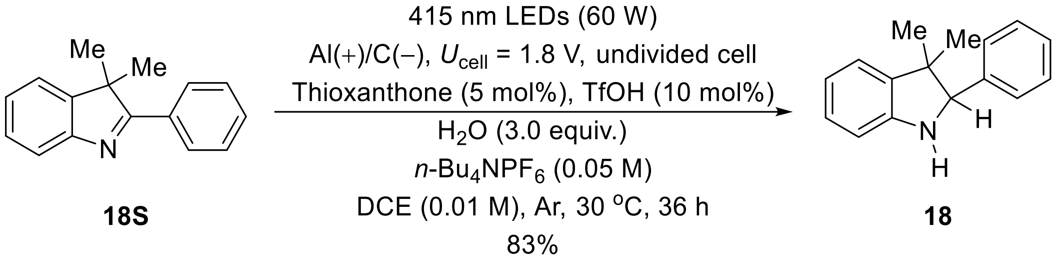

The reaction of **18S** (44.3 mg, 0.2 mmol), thioxanthone (2.1 mg, 0.01 mmol), TfOH (1.8 μL, 0.02 mmol), H_2O (11.0 μL, 0.6 mmol), n-Bu_4NPF_6 (387.3 mg, 1.0 mmol), and anhydrous DCE (20 mL) afforded **18**[23] as a white solid (36.9 mg, 83%). ^1H NMR (400 MHz, $CDCl_3$) δ 7.45 (d, J = 6.8 Hz, 2 H), 7.39-7.28 (m, 3 H), 7.12-7.02 (m, 2 H), 6.78 (t, J = 7.6 Hz, 1 H), 6.72 (d, J = 8.0 Hz, 1 H), 4.60 (s, 1 H), 4.07 (brs, 1 H), 1.43 (s, 3 H), 0.73 (s, 3 H).

13) 2'-Phenylspiro[cyclohexane-1,3'-indoline] (19)

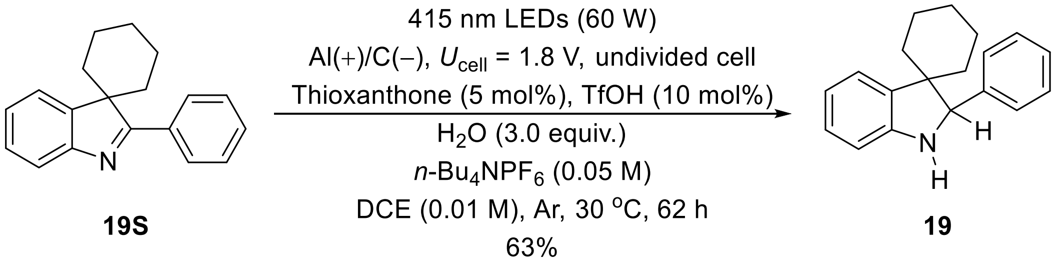

The reaction of **19S** (52.3 mg, 0.2 mmol), thioxanthone (2.1 mg, 0.01 mmol), TfOH (1.8 μL, 0.02 mmol), H_2O (10.8 μL, 0.6 mmol), n-Bu_4NPF_6 (387.4 mg, 1.0 mmol), and anhydrous DCE (20 mL) afforded **19**[23] as a thick liquid (33.2 mg, 63%). ^1H NMR (400 MHz, $CDCl_3$) δ 7.33-7.21 (m, 6 H), 7.08 (t, J = 7.6 Hz, 1 H), 6.75 (t, J = 8.4 Hz, 1 H), 6.66 (d, J = 7.6 Hz, 1 H), 4.55 (s, 1 H), 4.03 (brs, 1 H), 1.87-1.79 (m, 2 H), 1.73-1.66 (m, 2 H), 1.61-1.36 (m, 4 H), 1.24-1.08 (m, 2 H).

14) 2'-Methylspiro[cyclopentane-1,3'-indoline] (20)

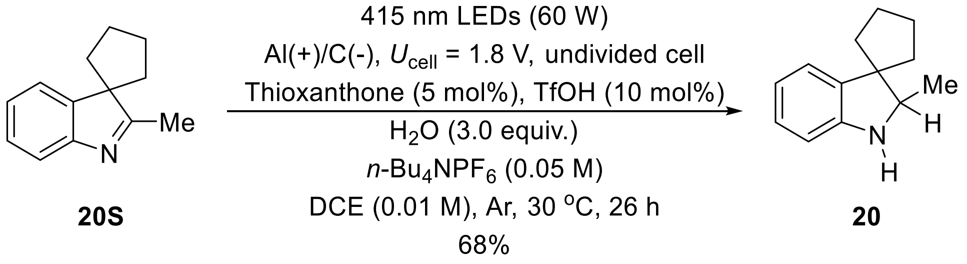

The reaction of **20S** (37.1 mg, 0.2 mmol), thioxanthone (2.1 mg, 0.01 mmol), TfOH (1.8 μL, 0.02 mmol), H_2O (10.8 μL, 0.6 mmol), n-Bu_4NPF_6 (387.5 mg, 1.0 mmol), and anhydrous DCE (20 mL)

afforded **20**[24] as a yellow oil (25.5 mg, 68%). ^{1}H NMR (400 MHz, $CDCl_3$) δ 7.07-6.97 (m, 2 H), 6.73 (t, *J* = 7.2 Hz, 1 H), 6.62 (d, *J* = 7.6 Hz, 1 H), 3.69-3.57 (m, 2 H), 2.03-1.46 (m, 8 H), 1.18 (d, *J* = 6.4 Hz, 3 H).

15) 2'-Methylspiro[cyclohexane-1,3'-indoline] (21)

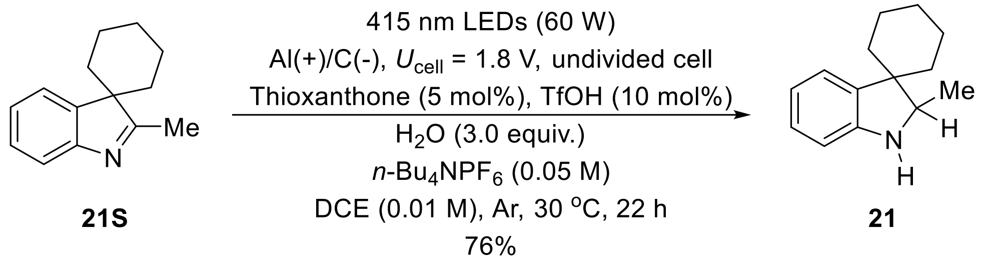

The reaction of **21S** (39.9 mg, 0.2 mmol), thioxanthone (2.1 mg, 0.01 mmol), TfOH (1.8 µL, 0.02 mmol), H_2O (10.8 µL, 0.6 mmol), *n*-Bu_4NPF_6 (387.4 mg, 1.0 mmol), and anhydrous DCE (20 mL) afforded **21**[24] as a solid (30.6 mg, 76%). ^{1}H NMR (400 MHz, $CDCl_3$) δ 7.14 (d, *J* = 7.2 Hz, 1 H), 7.02 (t, *J* = 7.6 Hz, 1 H), 6.72 (t, *J* = 7.6 Hz, 1 H), 6.61 (d, *J* = 7.6 Hz, 1 H), 3.69 (q, *J* = 6.4 Hz, 1 H), 1.76-1.35 (m, 10 H), 1.11 (d, *J* = 6.4 Hz, 3 H).

16) 2'-Methylspiro[2,3,5,6-tetrahydropyran-4,3'-indoline] (22)

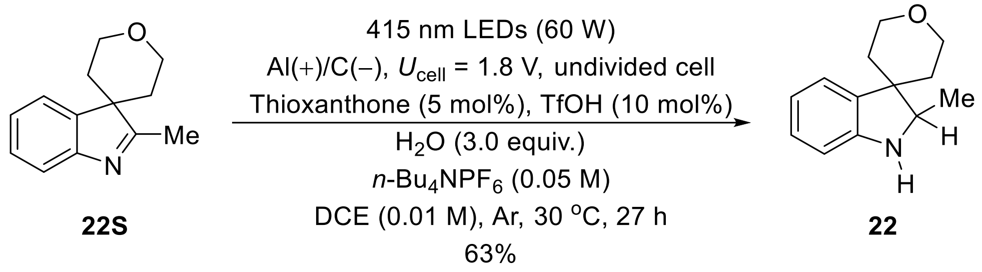

The reaction of **22S** (40.3 mg, 0.2 mmol), thioxanthone (2.1 mg, 0.01 mmol), TfOH (1.8 µL, 0.02 mmol), H_2O (10.8 µL, 0.6 mmol), *n*-Bu_4NPF_6 (387.4 mg, 1.0 mmol), and anhydrous DCE (20 mL) afforded **22**[24] as a white solid (25.6 mg, 63%). ^{1}H NMR (400 MHz, $CDCl_3$) δ 7.22 (d, *J* = 7.2 Hz, 1 H), 7.05 (t, *J* = 7.6 Hz, 1 H), 6.75 (t, *J* = 7.2 Hz, 1 H), 6.63 (d, *J* = 7.6 Hz, 1 H), 4.01-3.91 (m, 2 H), 3.84-3.73 (m, 2 H), 3.66-3.46 (m, 2 H), 1.99-1.89 (m, 1 H), 1.86-1.75 (m, 2 H), 1.73-1.62 (m, 1 H), 1.15 (d, *J* = 6.4 Hz, 3 H).

17) 5-(Thiophen-3-yl)-2,3,3-trimethylindoline (23)

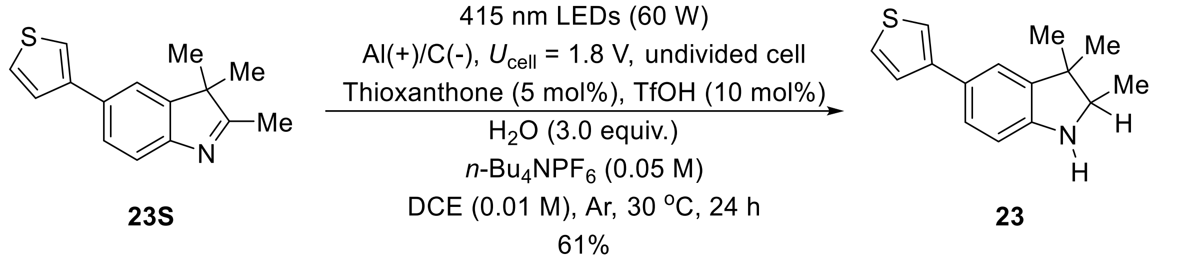

The reaction of **23S** (48.3 mg, 0.2 mmol), thioxanthone (2.1 mg, 0.01 mmol), TfOH (1.8 μL, 0.02 mmol), H_2O (10.8 μL, 0.6 mmol), n-Bu_4NPF_6 (387.5 mg, 1.0 mmol), and anhydrous DCE (20 mL) afforded **23** as a yellow oil (29.7 mg, 61%). ^{1}H NMR (400 MHz, $CDCl_3$) δ 7.32 (d, J = 2.4 Hz, 2 H), 7.29-7.22 (m, 3 H), 6.61 (d, J = 8.0 Hz, 1 H), 3.79 (brs, 1 H), 3.53 (q, J = 6.4 Hz, 1 H), 1.31 (s, 3 H), 1.18 (d, J = 6.4 Hz, 3 H), 1.07 (s, 3 H); ^{13}C NMR (101 MHz, $CDCl_3$) δ 148.6, 143.1, 139.6, 127.1, 126.3, 125.6, 120.6, 117.8, 109.3, 65.4, 43.4, 26.2, 22.4, 15.1; IR (neat) 3368, 1615, 1487, 1456, 1439 cm^{-1}; HRMS (ESI): calcd for $C_{15}H_{18}NS^+$ [M+H]$^+$: 244.1154, found: 244.1160.

18) 5-(Naphthalen-2-yl) -2,3,3-trimethylindoline (24)

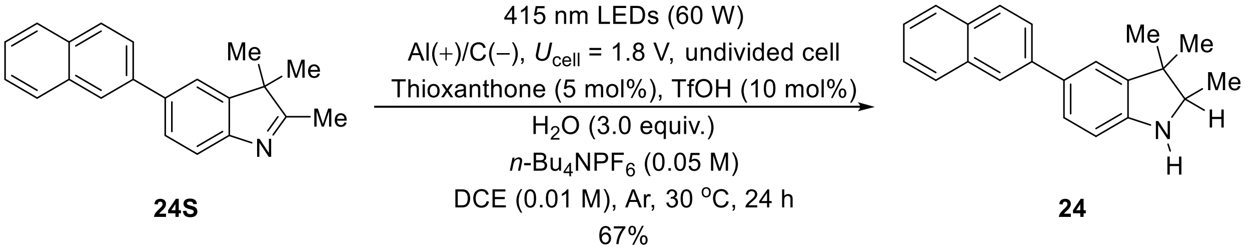

The reaction of **24S** (57.2 mg, 0.2 mmol), thioxanthone (2.1 mg, 0.01 mmol), TfOH (1.8 μL, 0.02 mmol), H_2O (10.8 μL, 0.6 mmol), n-Bu_4NPF_6 (387.5 mg, 1.0 mmol), and anhydrous DCE (20 mL) afforded **24** as a red solid (38.5 mg, 67%). Mp: 132.9-133.7 °C (n-hexane). ^{1}H NMR (400 MHz, $CDCl_3$) δ 7.96 (s, 1 H), 7.89-7.80 (m, 3 H), 7.72 (d, J = 10.0 Hz, 1 H), 7.50-7.37 (m, 4 H), 6.72 (d, J = 8.4 Hz, 1 H), 3.85 (brs, 1 H), 3.59 (q, J = 6.4 Hz, 1 H), 1.36 (s, 3 H), 1.22 (d, J = 6.8 Hz, 3 H), 1.13 (s, 3 H); ^{13}C NMR (101 MHz, $CDCl_3$) δ 149.0, 139.9, 139.3, 133.9, 132.1, 132.0, 128.1, 127.9, 127.6, 126.6, 126.0, 125.6, 125.2, 124.5, 121.4, 109.5, 65.5, 43.5, 26.3, 22.5, 15.2; IR (neat) 3377, 1612, 1496, 1476, 1442 cm^{-1}; HRMS (ESI): calcd for $C_{21}H_{22}N^+$ [M+H]$^+$: 288.1747, found: 288.1753.

19) 1,1,2-Trimethyl-2,3-dihydro-1*H*-benzo[*e*]indole (25)

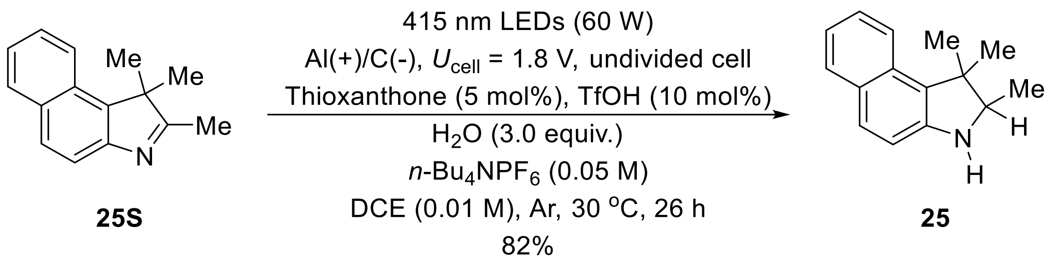

The reaction of **25S** (41.9 mg, 0.2 mmol), thioxanthone (2.1 mg, 0.01 mmol), TfOH (1.8 μL, 0.02 mmol), H_2O (10.8 μL, 0.6 mmol), *n*-Bu_4NPF_6 (387.6 mg, 1.0 mmol), and anhydrous DCE (20 mL) afforded **25**[25] as a colorless oil (34.7 mg, 82%). ^1H NMR (400 MHz, $CDCl_3$) δ 7.95 (d, *J* = 8.4 Hz, 1 H), 7.73 (d, *J* = 8.4 Hz, 1 H), 7.56 (d, *J* = 8.4 Hz, 1 H), 7.37 (t, *J* = 8.4 Hz, 1 H), 7.24-7.14 (m, 1 H), 6.94 (d, *J* = 8.4 Hz, 1 H), 3.84 (brs, 1 H), 3.61 (q, *J* = 6.4 Hz, 1 H), 1.59 (s, 3 H), 1.28-1.22 (m, 6 H).

20) 7-Bromo-1,1,2-trimethyl-2,3-dihydro-1*H*-benzo[*e*]indole (26)

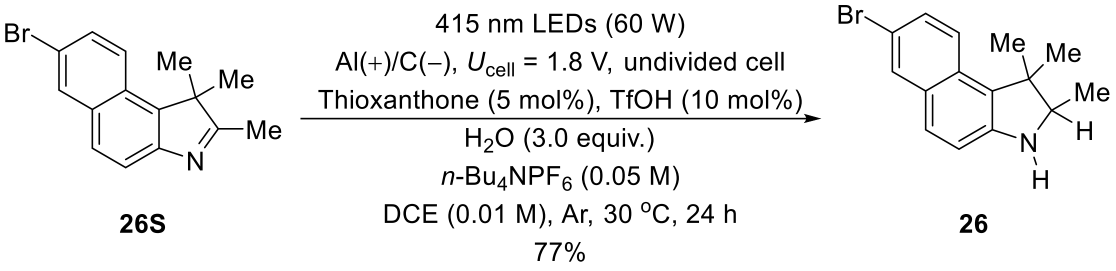

The reaction of **26S** (57.6 mg, 0.2 mmol), thioxanthone (2.1 mg, 0.01 mmol), TfOH (2.0 μL, 0.02 mmol), H_2O (11.0 μL, 0.6 mmol), *n*-Bu_4NPF_6 (387.3 mg, 1.0 mmol), and anhydrous DCE (20 mL) afforded **26** as a thick liquid (44.7 mg, 77%). ^1H NMR (400 MHz, $CDCl_3$) δ 7.86 (d, *J* = 2.0 Hz, 1 H), 7.80 (d, *J* = 9.2 Hz, 1 H), 7.48-7.39 (m, 2 H), 6.94 (d, *J* = 8.4 Hz, 1 H), 3.92 (brs, 1 H), 3.61 (q, *J* = 6.4 Hz, 1 H), 1.56 (s, 3 H), 1.27-1.20 (m, 6 H); ^{13}C NMR (101 MHz, $CDCl_3$) δ 147.5, 131.1, 130.7, 129.3, 129.1, 128.2, 127.7, 123.2, 114.7, 113.8, 65.6, 45.0, 27.0, 20.6, 14.4; IR (neat) 3374, 1621, 1590, 1510, 1456 cm^{-1}; HRMS (ESI): calcd for $C_{15}H_{16}BrN^+$ $[M]^+$: 289.0461, found: 289.0461.

21) *N*-benzylaniline (27)

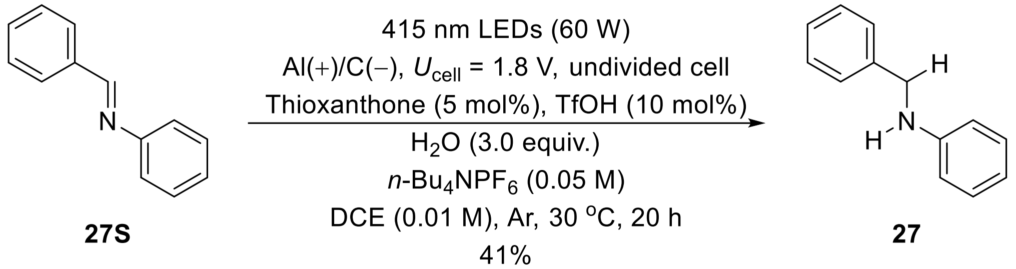

The reaction of **27S** (36.3 mg, 0.2 mmol), thioxanthone (2.1 mg, 0.01 mmol), TfOH (2.0 μL, 0.02 mmol), H_2O (11.0 μL, 0.6 mmol), *n*-Bu_4NPF_6 (387.6 mg, 1.0 mmol), and anhydrous DCE (20 mL) afforded **27**[26] as a white solid (15.0 mg, 41%). ^1H NMR (400 MHz, $CDCl_3$) δ 7.40-7.30 (m, 4 H), 7.29-7.25 (m, 1 H), 7.21-7.13 (m, 2 H), 6.71 (t, *J* = 7.2 Hz, 1 H), 6.64 (d, *J* = 7.6 Hz, 2 H), 4.33 (s, 2 H), 4.02 (brs, 1 H).

Typical procedure IV for the electrophotocatalytic reductive functionalization

Synthesis of methyl 4-(4,4,5,5-tetramethyl-1,3,2-dioxaborolan-2-yl)benzoate (29)

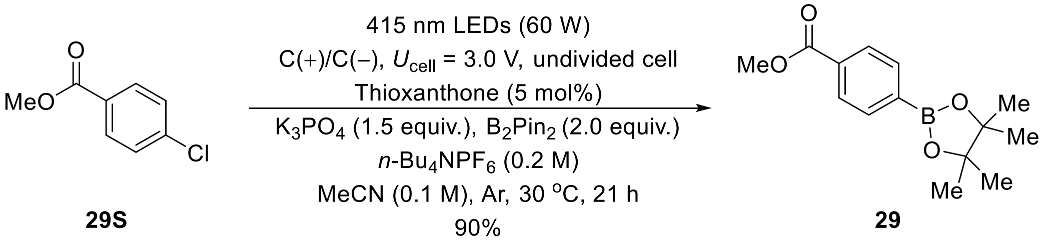

An undivided cell was prepared and equipped with a stir bar. To a flame dried 10 mL of Schlenk tube were added **29S** (68.3 mg, 0.4 mmol), thioxanthone (4.2 mg, 0.02 mmol), K_3PO_4 (127.5 mg, 0.6 mmol), B_2Pin_2 (203.7 mg, 0.8 mmol), *n*-Bu_4NPF_6 (310.1 mg, 0.8 mmol), and anhydrous MeCN (4 mL) under argon atmosphere. The cell was equipped with a carbon felt cathode (2 × 1 × 0.5 cm^3) and a carbon felt anode (2 × 1 × 0.5 cm^3), and was sealed using a rubber septum and parafilm. The reaction mixture was electrolyzed at a constant cell potential of 3.0 V under irradiation of 415 nm LEDs (60 W) at 30 °C (maintained with four cooling fans). The reaction was completed after 21 h as monitored by TLC (petroleum ether/ethyl acetate = 20:1). The crude product was collected by washing chamber and carbon felts with EtOAc (10 mL × 3) in an ultrasonic bath. The solvent was then removed and the residue was purified by flash chromatography on silica gel (eluent: petroleum ether/ethyl acetate = 40:1) to afford **29**[27] as a white solid (94.3 mg, 90%). ^1H NMR (400 MHz, $CDCl_3$) δ 8.02 (d, *J* = 8.4 Hz, 2 H), 7.87 (d, *J* = 8.4 Hz, 2 H), 3.92 (s, 3 H), 1.36 (s, 12 H).

The following compounds were synthesized according to typical procedure IV.

1) 4-Methyl-1,1'-biphenyl (28)

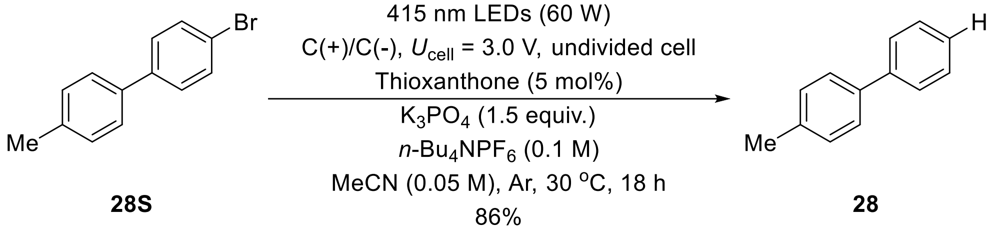

The reaction of **28S** (49.5 mg, 0.2 mmol), thioxanthone (2.1 mg, 0.01 mmol), K_3PO_4 (63.6 mg, 0.3 mmol), n-Bu_4NPF_6 (155.1 mg, 0.4 mmol), and anhydrous MeCN (4 mL) afforded **28**[28] as a white solid (28.9 mg, 86%). ^{1}H NMR (400 MHz, $CDCl_3$) δ 7.58 (d, J = 7.2 Hz, 2 H), 7.49 (d, J = 8.0 Hz, 2 H), 7.42 (t, J = 7.6 Hz, 2 H), 7.32 (t, J = 7.2 Hz, 1 H), 7.25 (d, J = 7.6 Hz, 2 H), 2.39 (s, 3 H).

2) 4-(4,4,5,5-Tetramethyl-1,3,2-dioxaborolan-2-yl) 1,1'-biphenyl (30)

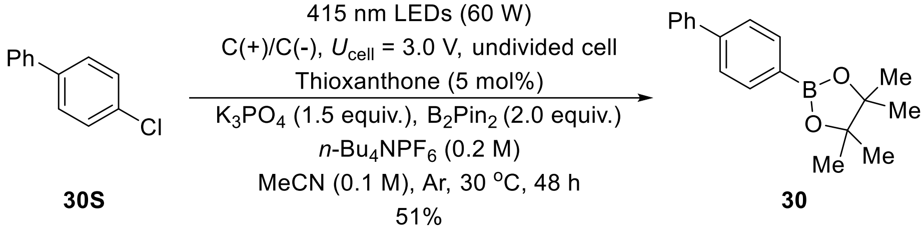

The reaction of **30S** (75.6 mg, 0.4 mmol), thioxanthone (4.2 mg, 0.02 mmol), K_3PO_4 (127.6 mg, 0.6 mmol), B_2Pin_2 (203.3 mg, 0.8 mmol), n-Bu_4NPF_6 (310.1 mg, 0.8 mmol), and anhydrous MeCN (4 mL) afforded **30**[6] as a colorless oil (57.1 mg, 51%). ^{1}H NMR (400 MHz, $CDCl_3$) δ 7.89 (d, J = 8.0 Hz, 2 H), 7.64-7.58 (m, 4 H), 7.44 (t, J = 7.6 Hz, 2 H), 7.39-7.31 (m, 1 H), 1.36 (s, 12 H).

3) Methyl 4-(4,4,5,5-tetramethyl-1,3,2-dioxaborolan-2-yl)benzoate (31)

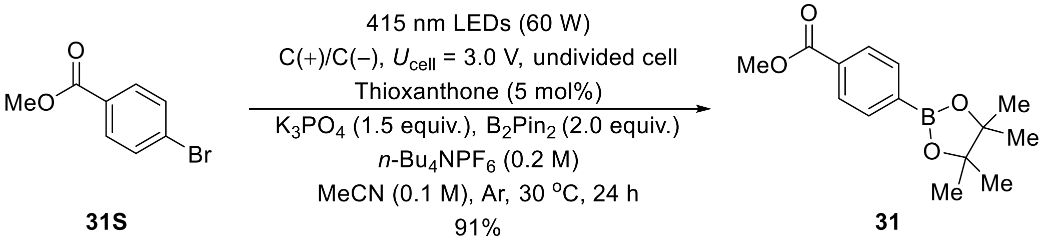

The reaction of **31S** (85.7 mg, 0.4 mmol), thioxanthone (4.2 mg, 0.02 mmol), K_3PO_4 (127.6 mg, 0.6 mmol), B_2Pin_2 (203.5 mg, 0.8 mmol), n-Bu_4NPF_6 (310.5 mg, 0.8 mmol), and anhydrous MeCN (4 mL) afforded **31**[27] as a white solid (95.4 mg, 91%). ^{1}H NMR (400 MHz, $CDCl_3$) δ 8.02 (d, J = 8.4 Hz, 2 H), 7.87 (d, J = 8.0 Hz, 2 H), 3.92 (s, 3 H), 1.36 (s, 12 H).

4) 4-(4,4,5,5-Tetramethyl-1,3,2-dioxaborolan-2-yl)benzonitrile (32)

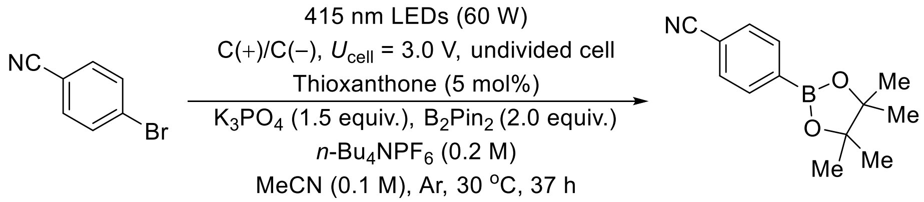

32S **32**

The reaction of **32S** (72.8 mg, 0.4 mmol), thioxanthone (4.2 mg, 0.02 mmol), K_3PO_4 (127.5 mg, 0.6 mmol), B_2Pin_2 (203.3 mg, 0.8 mmol), *n*-Bu_4NPF_6 (310.2 mg, 0.8 mmol), and anhydrous MeCN (4 mL) afforded **32**[29] as a white solid (57.7 mg, 63%). ^{1}H NMR (400 MHz, $CDCl_3$) δ 7.88 (d, J = 8.4 Hz, 2 H), 7.64 (d, J = 8.0 Hz, 2 H), 1.35 (s, 12 H).

5) 1-(4-(4,4,5,5-Tetramethyl-1,3,2-dioxaborolan-2-yl)phenyl)ethan-1-one (33)

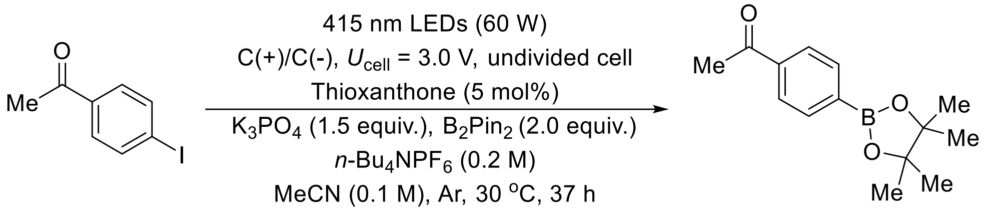

33S **33**

The reaction of **33S** (98.4 mg, 0.4 mmol), thioxanthone (4.2 mg, 0.02 mmol), K_3PO_4 (127.3 mg, 0.6 mmol), B_2Pin_2 (203.4 mg, 0.8 mmol), *n*-Bu_4NPF_6 (310.2 mg, 0.8 mmol), and anhydrous MeCN (4 mL) afforded **33**[30] as a colorless oil (63.9 mg, 65%). ^{1}H NMR (400 MHz, $CDCl_3$) δ 7.96-7.86 (m, 4 H), 2.62 (s, 3 H), 1.36 (s, 12 H).

6) 4-(4,4,5,5-Tetramethyl-1,3,2-dioxaborolan-2-yl)phenol (34)

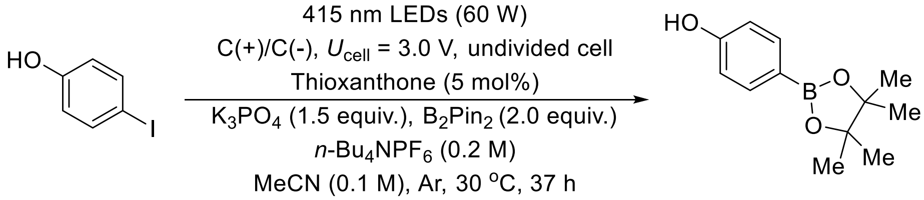

34S **34**

The reaction of **34S** (88.1 mg, 0.4 mmol), thioxanthone (4.2 mg, 0.02 mmol), K_3PO_4 (127.6 mg, 0.6 mmol), B_2Pin_2 (203.4 mg, 0.8 mmol), *n*-Bu_4NPF_6 (310.2 mg, 0.8 mmol), and anhydrous MeCN (4 mL) afforded **34**[31] as a white solid (81.8 mg, 93%). ^{1}H NMR (400 MHz, $CDCl_3$) δ 7.71 (d, J = 8.4 Hz, 2 H), 6.82 (d, J = 8.4 Hz, 2 H), 5.21 (brs, 1 H), 1.33 (s, 12 H).

7) Methyl 4-(5,5-dimethyl-1,3,2-dioxaborinan-2-yl)benzoate (35)

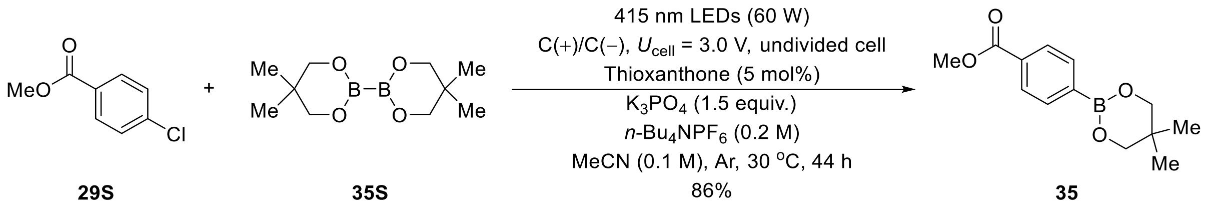

The reaction of **29S** (68.3 mg, 0.4 mmol), thioxanthone (4.2 mg, 0.02 mmol), K_3PO_4 (127.5 mg, 0.6 mmol), **35S** (180.9 mg, 0.8 mmol), n-Bu_4NPF_6 (311.0 mg, 0.8 mmol), and anhydrous MeCN (4 mL) afforded **35**[32] as a white solid (85.3 mg, 86%). ^{1}H NMR (400 MHz, $CDCl_3$) δ 8.00 (d, J = 8.4 Hz, 2 H), 7.86 (d, J = 8.4 Hz, 2 H), 3.91 (s, 3 H), 3.78 (s, 4 H), 1.03 (s, 6 H).

8) Methyl 4-(4,4,6-trimethyl-1,3,2-dioxaborinan-2-yl)benzoate (36)

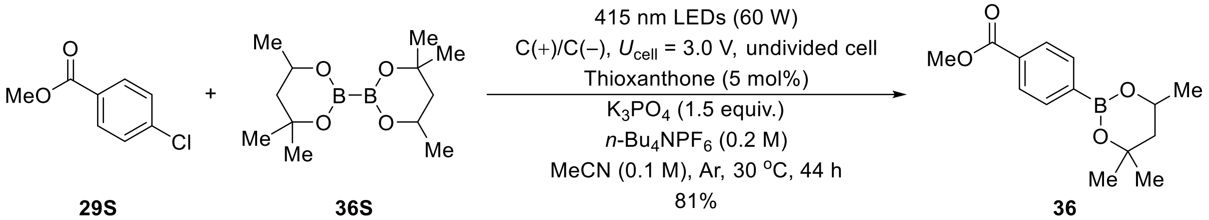

The reaction of **29S** (68.2 mg, 0.4 mmol), thioxanthone (4.2 mg, 0.02 mmol), K_3PO_4 (127.5 mg, 0.6 mmol), **36S** (203.4 mg, 0.8 mmol), n-Bu_4NPF_6 (310.2 mg, 0.8 mmol), and anhydrous MeCN (4 mL) afforded **36**[33] as a colorless thick oil (84.9 mg, 81%). ^{1}H NMR (400 MHz, $CDCl_3$) δ 7.98 (d, J = 8.4 Hz, 2 H), 7.86 (d, J = 8.0 Hz, 2 H), 4.40-4.30 (m, 1 H), 3.91 (s, 3 H), 1.87 (dd, J = 14.0, 2.8 Hz, 1 H), 1.60 (t, J = 12.0 Hz, 1 H), 1.40-1.32 (m, 9 H).

9) Methyl 4-(4,4,6,6-tetramethyl-1,3,2-dioxaborinan-2-yl)benzoate (37)

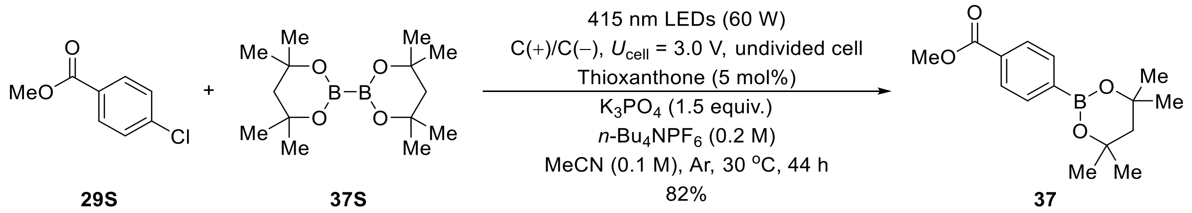

The reaction of **29S** (68.2 mg, 0.4 mmol), thioxanthone (4.2 mg, 0.02 mmol), K_3PO_4 (127.7 mg, 0.6 mmol), **37S** (225.7 mg, 0.8 mmol), n-Bu_4NPF_6 (310.3 mg, 0.8 mmol), and anhydrous MeCN (4 mL) afforded **37** as a white solid (90.6 mg, 82%). Mp: 71.4-72.3 °C (n-hexane). ^{1}H NMR (400 MHz, $CDCl_3$) δ 7.98 (d, J = 8.0 Hz, 2 H), 7.88 (d, J = 8.0 Hz, 2 H), 3.91 (s, 3 H), 1.93 (s, 2 H), 1.43 (s, 12 H); ^{13}C NMR (101 MHz, $CDCl_3$) δ 167.3, 133.7, 131.3, 128.3, 71.1, 51.9, 48.8, 31.7;

^{11}B NMR (128 MHz, $CDCl_3$) δ 26.13; IR (neat) 1726, 1437, 1407 cm^{-1}; HRMS (EI): calcd for $C_{15}H_{21}BO_4^+$ [M]$^+$: 276.1527, found: 276.1535.

10) Methyl 4-((3a*R*,4*R*,6*R*,7a*S*)-3a,5,5-trimethylhexahydro-4,6-methanobenzo[*d*][1,3,2]dioxaborol-2-yl)benzoate (38)

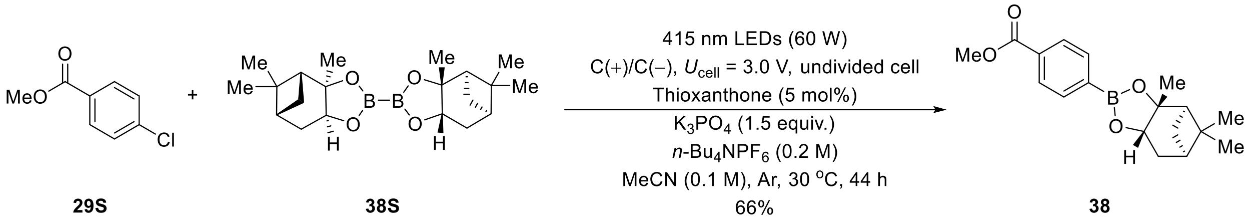

The reaction of **29S** (68.4 mg, 0.4 mmol), thioxanthone (4.2 mg, 0.02 mmol), K_3PO_4 (127.5 mg, 0.6 mmol), **38S** (287.1 mg, 0.8 mmol), *n*-Bu_4NPF_6 (310.1 mg, 0.8 mmol), and anhydrous MeCN (4 mL) afforded **38** as a white solid (82.9 mg, 66%). Mp: 100.2-101.0 °C (*n*-hexane). ^{1}H NMR (400 MHz, $CDCl_3$) δ 8.03 (d, J = 8.0 Hz, 2 H), 7.88 (d, J = 8.0 Hz, 2 H), 4.48 (dd, J = 8.8, 1.6 Hz, 1 H), 3.93 (s, 3 H), 2.48-2.37 (m, 1 H), 2.29-2.20 (m, 1 H), 2.16 (t, J = 5.6 Hz, 1 H), 2.03-1.93 (m, 2 H), 1.50 (s, 3 H), 1.32 (s, 3 H), 1.20 (d, J = 11.2 Hz, 1 H), 0.90 (s, 3 H); ^{13}C NMR (101 MHz, $CDCl_3$) δ 167.1, 134.7, 132.3, 128.6, 86.6, 78.5, 52.1, 51.4, 39.5, 38.2, 35.5, 28.7, 27.1, 26.5, 24.0; ^{11}B NMR (128 MHz, $CDCl_3$) δ 30.11; IR (neat) 1729, 1561, 1513, 1439, 1402 cm^{-1}; HRMS (ESI): calcd for $C_{18}H_{24}BO_4^+$ [M+H]$^+$: 315.1762, found: 315.1768.

11) Methyl 4-(trimethylstannyl)benzoate (39)

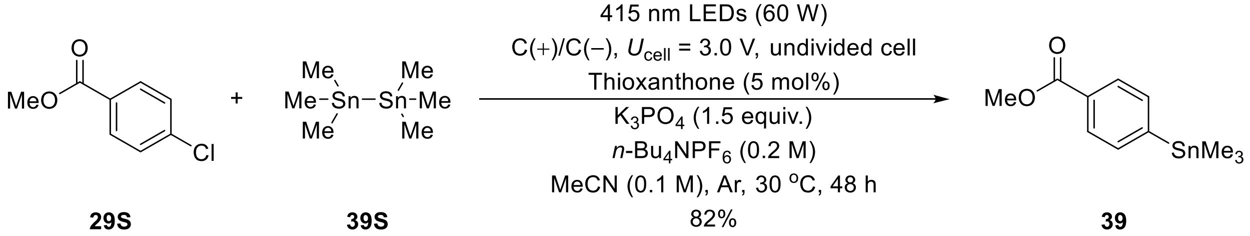

The reaction of **29S** (68.2 mg, 0.4 mmol), thioxanthone (4.2 mg, 0.02 mmol), K_3PO_4 (127.6 mg, 0.6 mmol), **39S** (166.0 μL, 0.8 mmol), *n*-Bu_4NPF_6 (310.4 mg, 0.8 mmol), and anhydrous MeCN (4 mL) afforded **39**[34] as a colorless oil (97.9 mg, 82%). ^{1}H NMR (400 MHz, $CDCl_3$) δ 7.97 (d, J = 7.6 Hz, 2 H), 7.58 (d, J = 8.0 Hz, 2 H), 3.91 (s, 3 H), 0.32 (s, 9 H).

12) Methyl 2,6-difluoro-2',4',6'-trimethoxy-[1,1'-biphenyl]-4-carboxylate (40)

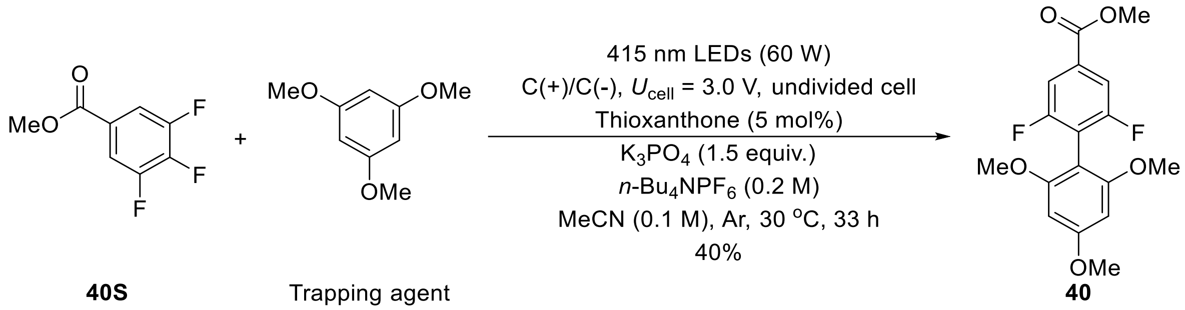

The reaction of **40S** (76.0 mg, 0.4 mmol), thioxanthone (4.2 mg, 0.02 mmol), K_3PO_4 (127.5 mg, 0.6 mmol), 1,3,5-trimethoxybenzene (336.5 mg, 2.0 mmol), n-Bu_4NPF_6 (310.1 mg, 0.8 mmol), and anhydrous MeCN (4 mL) afforded **40**[35] as a white solid (54.0 mg, 40%). ^{1}H NMR (400 MHz, $CDCl_3$) δ 7.59 (d, J = 7.6 Hz, 2 H), 6.22 (s, 2 H), 3.93 (s, 3 H), 3.87 (s, 3 H), 3.75 (s, 6 H).

13) 2,6-Difluoro-2',4',6'-trimethoxy-[1,1'-biphenyl]-4-carbonitrile (41)

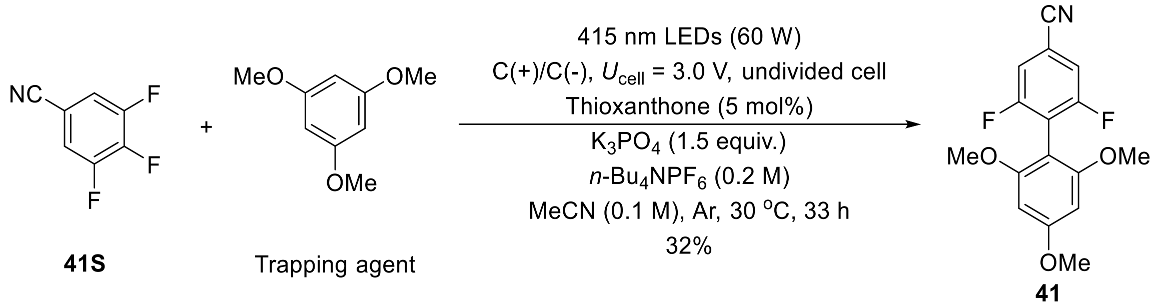

The reaction of **41S** (62.8 mg, 0.4 mmol), thioxanthone (4.2 mg, 0.02 mmol), K_3PO_4 (127.6 mg, 0.6 mmol), 1,3,5-trimethoxybenzene (336.5 mg, 2.0 mmol), n-Bu_4NPF_6 (310.0 mg, 0.8 mmol), and anhydrous MeCN (4 mL) afforded **41**[35] as a white solid (39.0 mg, 32%). ^{1}H NMR (400 MHz, $CDCl_3$) δ 7.22 (d, J = 6.4 Hz, 2 H), 6.22 (s, 2 H), 3.87 (s, 3 H), 3.75 (s, 6 H).

14) 1-Methoxy-4-(1-phenyl-1*H*-pyrrole-2-yl)benzene (42)

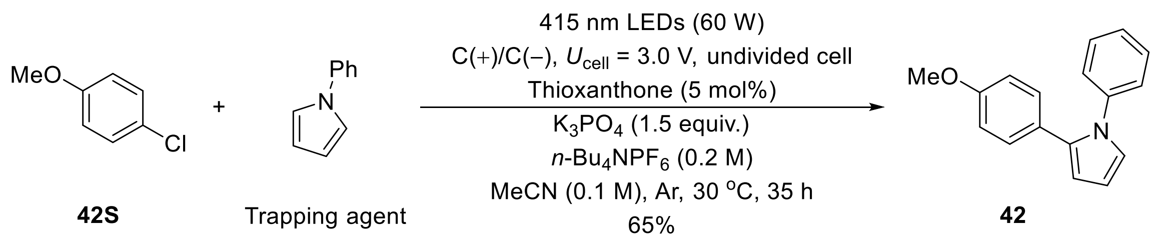

The reaction of **42S** (49.0 μL, 0.4 mmol), thioxanthone (4.2 mg, 0.02 mmol), K_3PO_4 (127.6 mg, 0.6 mmol), 1-phenyl-1*H*-pyrrole (1.1457 g, 8.0 mmol), n-Bu_4NPF_6 (310.2 mg, 0.8 mmol), and anhydrous MeCN (4 mL) afforded **42**[36] as a white solid (64.8 mg, 65%). ^{1}H NMR (400 MHz,

$CDCl_3$) δ 7.31 (t, J = 7.2 Hz, 2 H), 7.25 (t, J = 6.8 Hz, 1 H), 7.16 (d, J = 7.6 Hz, 2 H), 7.05 (d, J = 8.8 Hz, 2 H), 6.94-6.89 (m, 1 H), 6.75 (d, J = 8.8 Hz, 2 H), 6.37-6.32 (m, 2 H), 3.76 (s, 3 H).

15) 2,6-Di-*tert*-butyl-1-methoxy-4-(1-methyl-1*H*-pyrrole-2-yl)benzene (43)

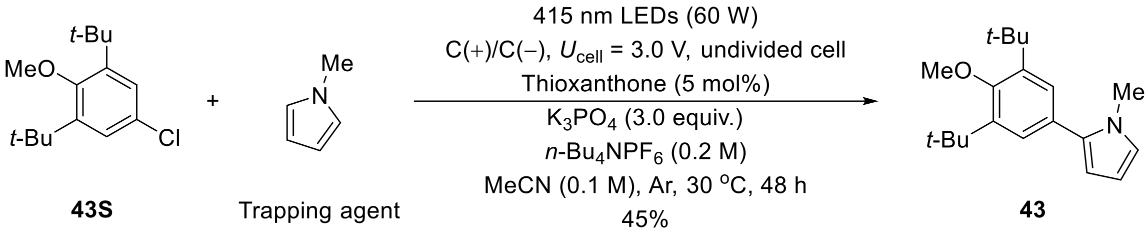

The reaction of **43S** (101.9 mg, 0.4 mmol), thioxanthone (4.2 mg, 0.02 mmol), K_3PO_4 (256.3 mg, 1.2 mmol), 1-methyl-1*H*-pyrrole (710.0 μL, 8.0 mmol), *n*-Bu_4NPF_6 (311.3 mg, 0.8 mmol), and anhydrous MeCN (4 mL) afforded **43**[4] as a white solid (53.9 mg, 45%). ^1H NMR (400 MHz, $CDCl_3$) δ 7.27 (s, 2 H), 6.73-6.68 (m, 1 H), 6.23-6.17 (m, 2 H), 3.74 (s, 3 H), 3.66 (s, 3 H), 1.46 (s, 18 H).

16) 5-(1*H*-pyrrole-2-yl)benzo[*b*]thiophene (44)

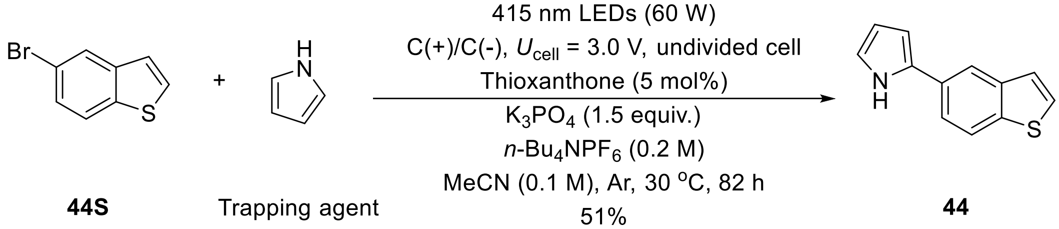

The reaction of **44S** (85.3 mg, 0.4 mmol), thioxanthone (4.2 mg, 0.02 mmol), K_3PO_4 (127.5 mg, 0.6 mmol), 1*H*-pyrrole (555.0 μL, 8.0 mmol), *n*-Bu_4NPF_6 (310.2 mg, 0.8 mmol), and anhydrous MeCN (4 mL) afforded **44** as a solid (40.6 mg, 51%). Mp: 132.5-133.3 °C (petroleum ether/ethyl acetate). ^1H NMR (400 MHz, $CDCl_3$) δ 8.48 (brs, 1 H), 7.91-7.83 (m, 2 H), 7.53-7.43 (m, 2 H), 7.33 (d, J = 5.2 Hz, 1 H), 6.89 (s, 1 H), 6.57 (s, 1 H), 6.36-6.30 (m, 1 H); ^{13}C NMR (101 MHz, $CDCl_3$) δ 140.2, 137.6, 132.3, 129.4, 127.2, 123.8, 122.9, 121.3, 118.7, 118.3, 110.2, 106.0; IR (neat) 3437, 1655, 1595, 1499, 1462 cm^{-1}; HRMS (EI): calcd for $C_{12}H_9NS^+$ $[M]^+$: 199.0450, found: 199.0451.

17) Methyl 4-(1-methyl-1*H*-pyrrol-2-yl)benzoate (45)

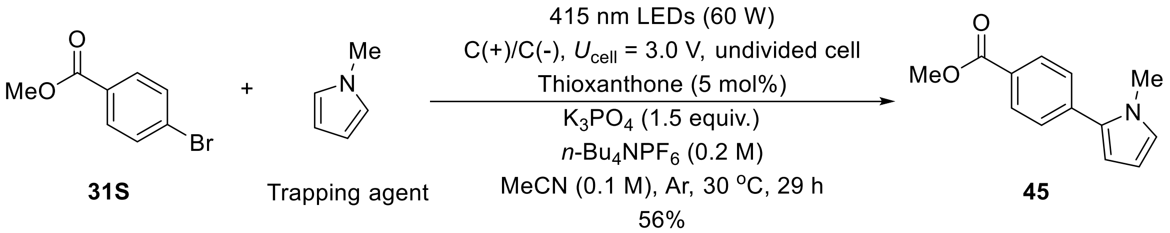

The reaction of **31S** (85.7 mg, 0.4 mmol), thioxanthone (4.2 mg, 0.02 mmol), K_3PO_4 (127.8 mg, 0.6 mmol), 1-methyl-1*H*-pyrrole (710.0 μL, 8.0 mmol), n-Bu_4NPF_6 (310.2 mg, 0.8 mmol), and anhydrous MeCN (4 mL) afforded **45**[37] as a white solid (47.8 mg, 56%). ^{1}H NMR (400 MHz, $CDCl_3$) δ 8.05 (d, J = 8.4 Hz, 2 H), 7.47 (d, J = 8.4 Hz, 2 H), 6.76 (t, J = 2.0 Hz, 1 H), 6.34 (dd, J = 3.6, 1.6 Hz, 1 H), 6.22 (t, J = 3.6 Hz, 1 H), 3.93 (s, 3 H), 3.71 (s, 3 H).

Gram-scale synthesis and synthetic derivatization

Gram-scale synthesis of methyl 4-(4,4,5,5-tetramethyl-1,3,2-dioxaborolan-2-yl)benzoate (29)

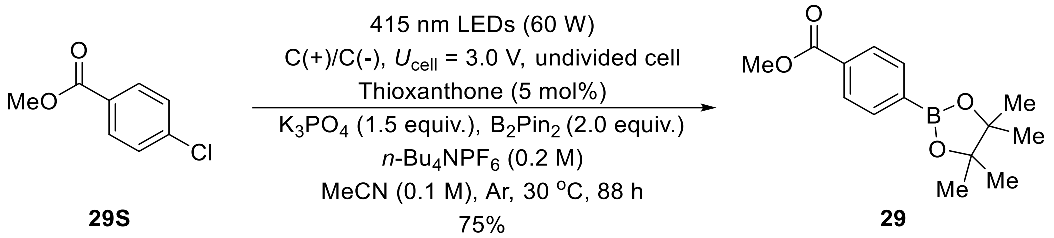

An undivided cell was prepared and equipped with a stir bar. To a flame dried 250 mL of three necked flask were added **29S** (1.024 g, 6.0 mmol), thioxanthone (63.6 mg, 0.3 mmol), K_3PO_4 (1.911 g, 9.0 mmol), B_2Pin_2 (3.048 g, 12.0 mmol), n-Bu_4NPF_6 (4.648 g, 12.0 mmol), and anhydrous MeCN (60 mL) under argon atmosphere. The cell was equipped with a carbon felt cathode (3 × 2 × 0.5 cm^3) and a carbon felt anode (3 × 2 × 0.5 cm^3), and was sealed using a rubber septum and parafilm. The reaction mixture was electrolyzed at a constant cell potential of 3.0 V under irradiation of 415 nm LEDs (60 W) at 30 °C (maintained with four cooling fans). The reaction was completed after 88 h as monitored by TLC (petroleum ether/ethyl acetate = 20:1). The crude product was collected by washing chamber and carbon felts with EtOAc (10 mL × 3) in an ultrasonic bath. The solvent was removed and the residue was then diluted with H_2O (60 mL). The aqueous layer was extracted with ethyl acetate (60 mL × 3). The combined organic layer was dried over $MgSO_4$, filtered and concentrated under reduced pressure. The crude product was purified by flash chromatography on silica gel (eluent: petroleum ether/ethyl acetate = 40:1) to afford **29**[27] as

a white solid (1.180 g, 75%). ^1H NMR (400 MHz, $CDCl_3$) δ 8.02 (d, J = 8.0 Hz, 2 H), 7.87 (d, J = 8.4 Hz, 2 H), 3.92 (s, 3 H), 1.36 (s, 12 H).

Synthesis of 4-hydroxybenzoic acid (46)[38]

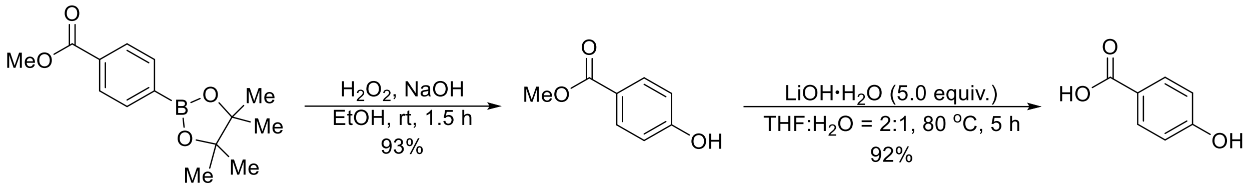

29 **47** **46**
Methylparaben PHBA

To a 10 mL of Pyrex sealed tube were added **29** (52.4 mg, 0.2 mmol), EtOH (3 mL), H_2O_2 (30%, 1.2 mL) and an aqueous solution of NaOH (1M, 1.2 mL). The resulting mixture was stirred at room temperature. The reaction was completed after 1.5 h as monitored by TLC (petroleum ether/ethyl acetate = 4:1). The mixture was concentrated in vacuo, and the residue was partitioned between EtOAc (10 mL × 3) and an aqueous solution of HCl (1 M). The combined organic layer was dried over $MgSO_4$, filtered and concentrated under reduced pressure. The crude product was purified by flash chromatography on silica gel (eluent: petroleum ether/ethyl acetate = 4:1) to afford **47**[39] as a white solid (28.3 mg, 93%). ^1H NMR (400 MHz, $CDCl_3$) δ 7.96 (d, J = 8.8 Hz, 2 H), 6.87 (d, J = 8.4 Hz, 2 H), 5.81 (brs, 1 H), 3.89 (s, 3 H).

To a 25 mL of vial were added **47** (30.5 mg, 0.2 mmol), THF (4 mL), H_2O (2 mL), and lithium hydroxide monohydrate (42.5 mg, 1.0 mmol). The resulting mixture was stirred at 80 °C with an oil bath for 5 h as monitored by TLC (petroleum ether/ethyl acetate = 4:1). The solvent was evaporated and the residue was diluted with water (20 mL). The water layer was acidified using conc. HCl to pH = 4. Then, the aqueous layer was extracted with EtOAc (20 mL × 6). The combined organic layer was dried over $MgSO_4$, filtered and concentrated under reduced pressure to afford **46**[40] as a white solid (25.3 mg, 92%). ^1H NMR (400 MHz, DMSO-d_6) δ 12.39 (brs, 1 H), 10.28 (brs, 1 H), 7.78 (d, J = 8.4 Hz, 2 H), 6.83 (d, J = 8.4 Hz, 2 H).

Synthesis of 4-aminobenzoic acid (49)[38]

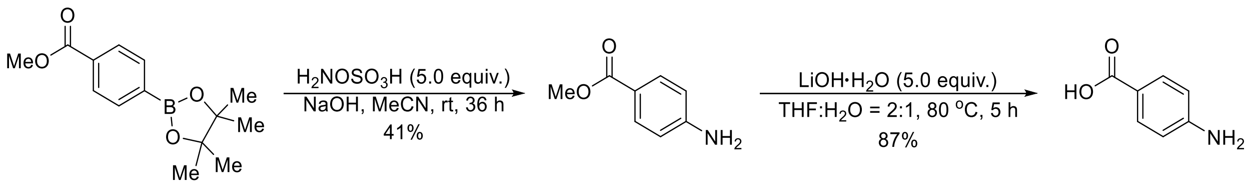

29 **48** **49**
Benzocaine analogue PABA

To a flame dried 10 mL of Pyrex sealed tube were added **29** (52.5 mg, 0.2 mmol), H_2NOSO_3H (113.6 mg, 1.0 mmol), anhydrous MeCN (1 mL), and an aqueous solution of NaOH (1 M, 1 mL) under argon atmosphere. The resulting mixture was stirred at room temperature. The reaction was completed after 36 h as monitored by TLC (petroleum ether/ethyl acetate = 4:1). After completion, the reaction was quenched by using an aqueous solution of HCl (1 M) to pH = 7. The aqueous layer was extracted with EtOAc (10 mL × 3). The combined organic layer was washed by brine and then dried over $MgSO_4$, filtered and concentrated under reduced pressure. The crude product was purified by flash chromatography on silica gel (eluent: petroleum ether/ethyl acetate = 4:1) to afford **48**[41] as a white solid (12.4 mg, 41%). ^{1}H NMR (400 MHz, $CDCl_3$) δ 7.84 (d, J = 8.4 Hz, 2 H), 6.63 (d, J = 8.4 Hz, 2 H), 4.12 (brs, 2 H), 3.85 (s, 3 H).

To a 25 mL of vial were added **48** (30.4 mg, 0.2 mmol), THF (4 mL), H_2O (2 mL), and lithium hydroxide monohydrate (42.3 mg, 1.0 mmol). The resulting mixture was stirred at 80 ^oC with an oil bath for 5 h as monitored by TLC (dichloromethane/methanol = 15:1). The solvent was evaporated and the residue was diluted with water (40 mL). The water layer was acidified using an aqueous solution of HCl (1 M) to pH = 7. Then, the aqueous layer was extracted with EtOAc (40 mL × 3). The combined organic layer was dried over $MgSO_4$, filtered and concentrated under reduced pressure to afford **49**[42] as a white solid (23.9 mg, 87%). ^{1}H NMR (400 MHz, DMSO-d_6) δ 11.93 (brs, 1 H), 7.67 (d, J = 8.4 Hz, 2 H), 6.59 (d, J = 8.4 Hz, 2 H), 5.84 (brs, 2 H).

Synthesis of 4'-octyl-[1,1'-biphenyl]-4-carboxylic acid (50)

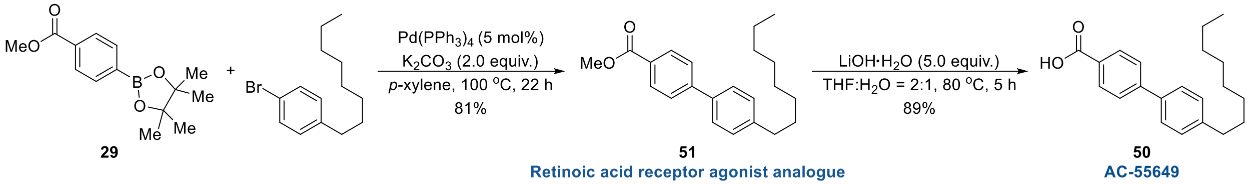

To a flame dried 10 mL of Pyrex sealed tube were added **29** (131.1 mg, 0.5 mmol), $Pd(PPh_3)_4$ (29.1 mg, 0.025 mmol), K_2CO_3 (138.0 mg, 1.0 mmol), anhydrous *p*-xylene (1 mL), and 1-bromo-4-octylbenzene (143.0 μL, 0.6 mmol) under argon atmosphere. The resulting mixture was stirred at 100 ^oC with an oil bath for 22 h as monitored by TLC (petroleum ether/ethyl acetate = 40:1). The solvent was removed and the residue was purified by flash chromatography on silica gel (eluent: petroleum ether/ethyl acetate = 80:1) to afford **51** as a white solid (132.0 mg, 81%). Mp: 78.5-79.3 ^oC (*n*-hexane). ^{1}H NMR (400 MHz, $CDCl_3$) δ 8.08 (d, J = 8.4 Hz, 2 H), 7.65 (d, J = 8.0 Hz, 2 H), 7.54 (d, J = 8.0 Hz, 2 H), 7.27 (d, J = 8.0 Hz, 2 H), 3.93 (s, 3 H), 2.65 (t, J = 7.6 Hz, 2

H), 1.68-1.61 (m, 2 H), 1.35-1.26 (m, 10 H), 0.88 (t, *J* = 6.4 Hz, 3 H); ^{13}C NMR (101 MHz, $CDCl_3$) δ 167.0, 145.6, 143.2, 137.2, 130.0, 129.0, 128.6, 127.1, 126.8, 52.0, 35.6, 31.9, 31.4, 29.5, 29.3, 29.2, 22.7, 14.1; IR (neat) 1726, 1609, 1470, 1436, 1402 cm^{-1}; HRMS (EI): calcd for $C_{22}H_{28}O_2^+$ $[M]^+$: 324.2084, found: 324.2084.

To a 25 mL of vial were added **51** (67.6 mg, 0.2 mmol), THF (4 mL), H_2O (2 mL), and lithium hydroxide monohydrate (42.3 mg, 1.0 mmol). The resulting mixture was stirred at 80 °C with an oil bath for 5 h as monitored by TLC (dichloromethane/methanol = 15:2). The solvent was evaporated and the residue was diluted with water (1 mL). The water layer was acidified using conc. HCl to pH = 1. The resulting mixture was filtered, washed with H_2O (2 mL), and then dried in the oven to afford **50**[43] as a white solid (57.8 mg, 89%). ^{1}H NMR (400 MHz, $CDCl_3$) δ 8.18 (d, *J* = 8.0 Hz, 2 H), 7.70 (d, *J* = 8.0 Hz, 2 H), 7.56 (d, *J* = 8.0 Hz, 2 H), 7.29 (d, *J* = 7.6 Hz, 2 H), 2.66 (t, *J* = 7.2 Hz, 2 H), 1.69-1.62 (m, 2 H), 1.35-1.26 (m, 10 H), 0.88 (t, *J* = 6.4 Hz, 3 H).

Synthesis of 4-(isoquinolin-1-yl)-*N*-(2-morpholinoethyl)benzamide (53)[44]

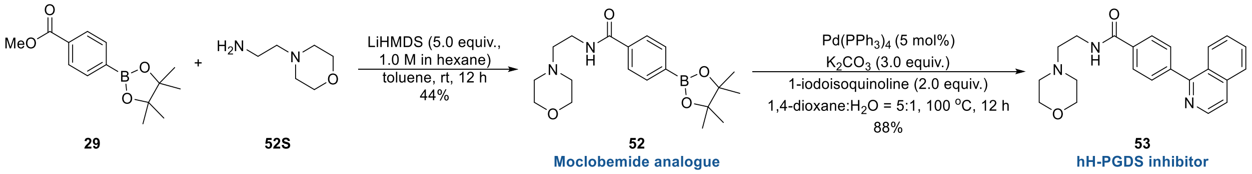

To a flame dried 10 mL of Pyrex sealed tube were added **29** (52.4 mg, 0.2 mmol), **52S** (52.5 μL, 0.4 mmol), anhydrous toluene (0.8 mL), and LiHMDS (1 mL, 1.0 mmol, 1.0 M in hexane) under argon atmosphere. The resulting mixture was stirred at room temperature. The reaction was completed after 12 h as monitored by TLC (dichloromethane/methanol = 10:1). After completion, the reaction was quenched with saturated NH_4Cl solution. The aqueous layer was extracted with EtOAc (10 mL × 3). The combined organic layer was washed by brine and then dried over $MgSO_4$, filtered and concentrated under reduced pressure. The crude product was purified by flash chromatography on silica gel (eluent: dichloromethane/methanol = 30:1) to afford **52** as a white solid (31.7 mg, 44%). Mp: 179.7-180.3 °C (*n*-hexane/dichloromethane). ^{1}H NMR (400 MHz, $CDCl_3$) δ 7.88 (d, *J* = 8.0 Hz, 2 H), 7.75 (d, *J* = 8.0 Hz, 2 H), 6.79 (brs, 1 H), 3.72 (t, *J* = 4.4 Hz, 4 H), 3.56 (q, *J* = 5.6 Hz, 2 H), 2.61 (t, *J* = 6.0 Hz, 2 H), 2.51 (t, *J* = 4.0 Hz, 4 H), 1.36 (s, 12 H); ^{13}C NMR (101 MHz, $CDCl_3$) δ 167.3, 136.8, 134.9, 126.0, 84.1, 67.0, 56.8, 53.3, 36.0, 24.8; ^{11}B NMR (128 MHz, $CDCl_3$) δ 30.59; IR (neat) 3331, 1644, 1544, 1456 cm^{-1}; HRMS (ESI): calcd for $C_{19}H_{30}BN_2O_4^+$ $[M+H]^+$: 361.2293, found: 361.2291.

To a flame dried 10 mL of Pyrex sealed tube were added **52** (72.2 mg, 0.2 mmol), 1-iodoisoquinoline (102.1 mg, 0.4 mmol), $Pd(PPh_3)_4$ (11.8 mg, 0.01 mmol), K_2CO_3 (83.4 mg, 0.6 mmol), anhydrous 1,4-dioxane (2 mL), and H_2O (0.4 mL) under argon atmosphere. The resulting mixture was stirred at 100 ^oC with an oil bath for 12 h as monitored by TLC (dichloromethane/methanol = 10:1). The reaction was diluted with water (40 mL). The aqueous layer was extracted with EtOAc (40 mL × 3). The combined organic layer was dried over $MgSO_4$, filtered and concentrated under reduced pressure. The crude product was purified by flash chromatography on silica gel (eluent: dichloromethane/methanol = 20:1) to afford **53** as a white solid (63.7 mg, 88%). Mp: 190.7-191.5 ^oC (*n*-hexane/dichloromethane). ^1H NMR (400 MHz, $CDCl_3$) δ 8.63 (d, J = 5.6 Hz, 1 H), 8.05 (d, J = 8.4 Hz, 1 H), 7.95 (d, J = 8.0 Hz, 2 H), 7.91 (d, J = 8.4 Hz, 1 H), 7.79 (d, J = 8.0 Hz, 2 H), 7.75-7.66 (m, 2 H), 7.56 (t, J = 8.0 Hz, 1 H), 6.92 (brs, 1 H), 3.75 (t, J = 4.4 Hz, 4 H), 3.61 (q, J = 5.6 Hz, 2 H), 2.65 (t, J = 5.6 Hz, 2 H), 2.54 (t, J = 4.0 Hz, 4 H); ^{13}C NMR (101 MHz, $CDCl_3$) δ 167.1, 159.5, 142.6, 142.2, 136.8, 134.6, 130.2, 127.4, 127.1, 127.0, 126.9, 126.5, 120.3, 67.0, 56.8, 53.3, 36.1; IR (neat) 3328, 1644, 1584, 1549, 1498, 1455 cm^{-1}; HRMS (ESI): calcd for $C_{22}H_{24}N_3O_2^+$ $[M+H]^+$: 362.1863, found: 362.1858.

Compared with the previous strategies for electrophotocatalytic coupling reactions

Supplementary Figure 24. Previous strategies for electrophotocatalytic coupling reactions[4,6]

Using dicyanoanthracene or anthraquinone as electrophotocatalyst, Mg^0 was sacrificed. Using naphthalene imides as electrophotocatalyst, Et_3N (2 equiv) was added to the anodic chamber as a terminal reductant. Using dicyanoanthracene as electrophotocatalyst, the faradaic efficiency was 35%.

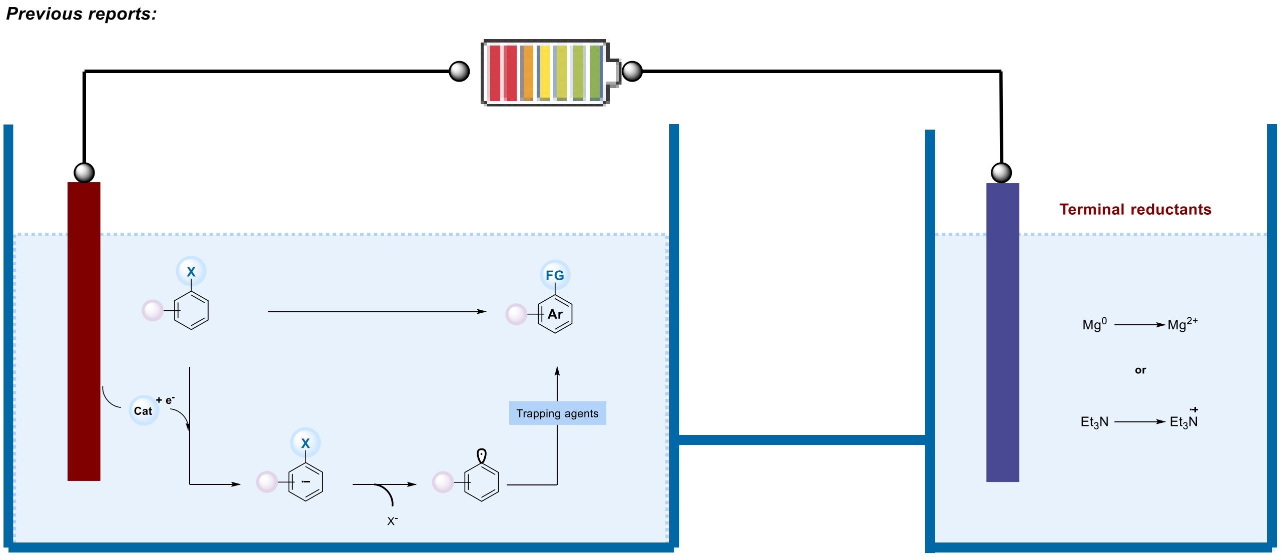

Supplementary Figure 25. Our strategy for electrophotocatalytic coupling reactions

In our work, radical intermediates were used as sacrificial agents. The faradaic efficiency was 66%.

This work:

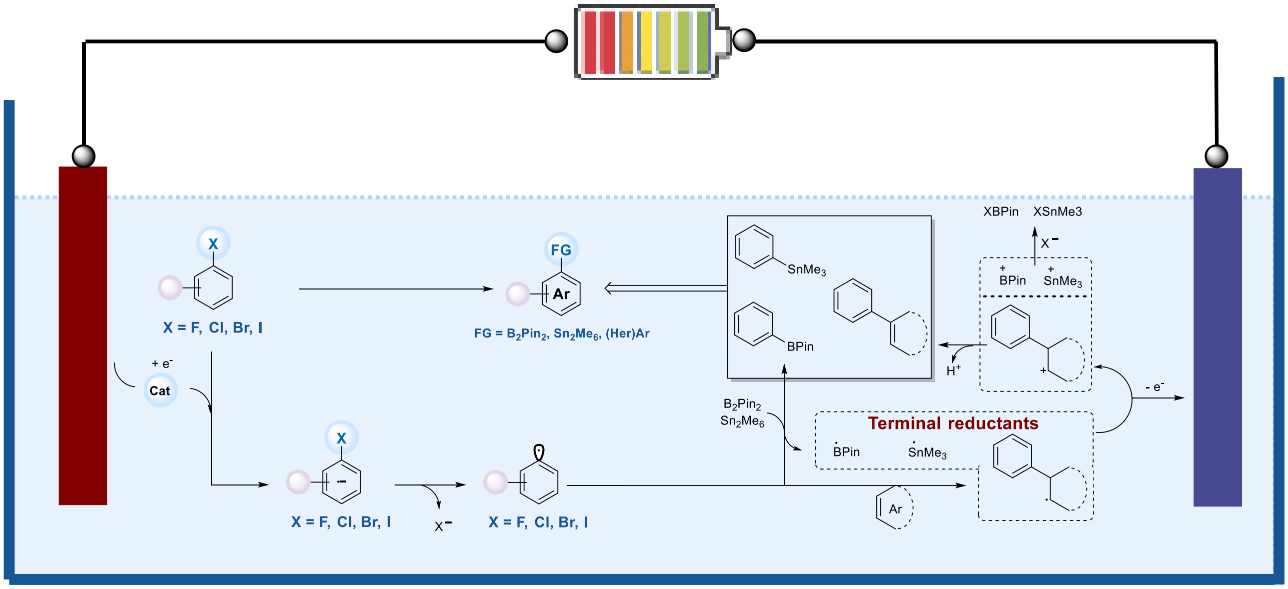

NMR spectra of substrates and products

Supplementary Figure 26. ^{1}H NMR spectra of compound **18-d**~22% D (400 MHz, $CDCl_3$)

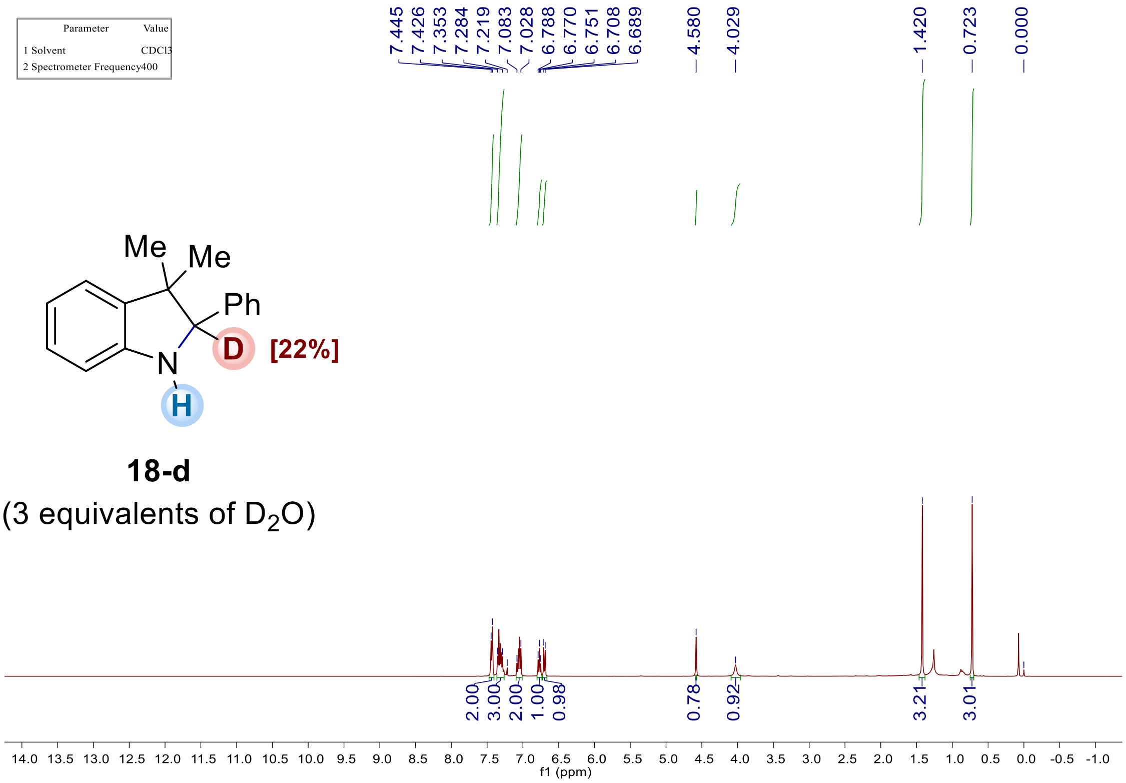

Supplementary Figure 27. ^{1}H NMR spectra of compound **18-d**~45% D (400 MHz, $CDCl_3$)

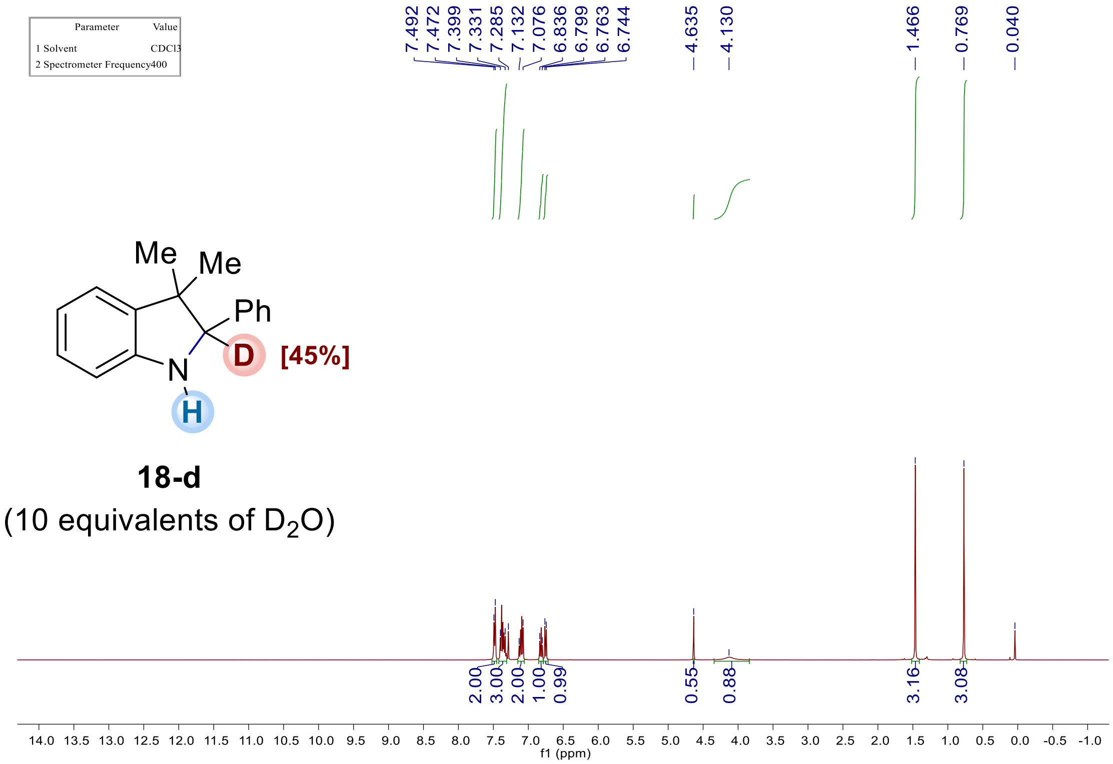

Supplementary Figure 28. ^{1}H NMR spectra of compound **18-d**~81% D (400 MHz, $CDCl_3$)

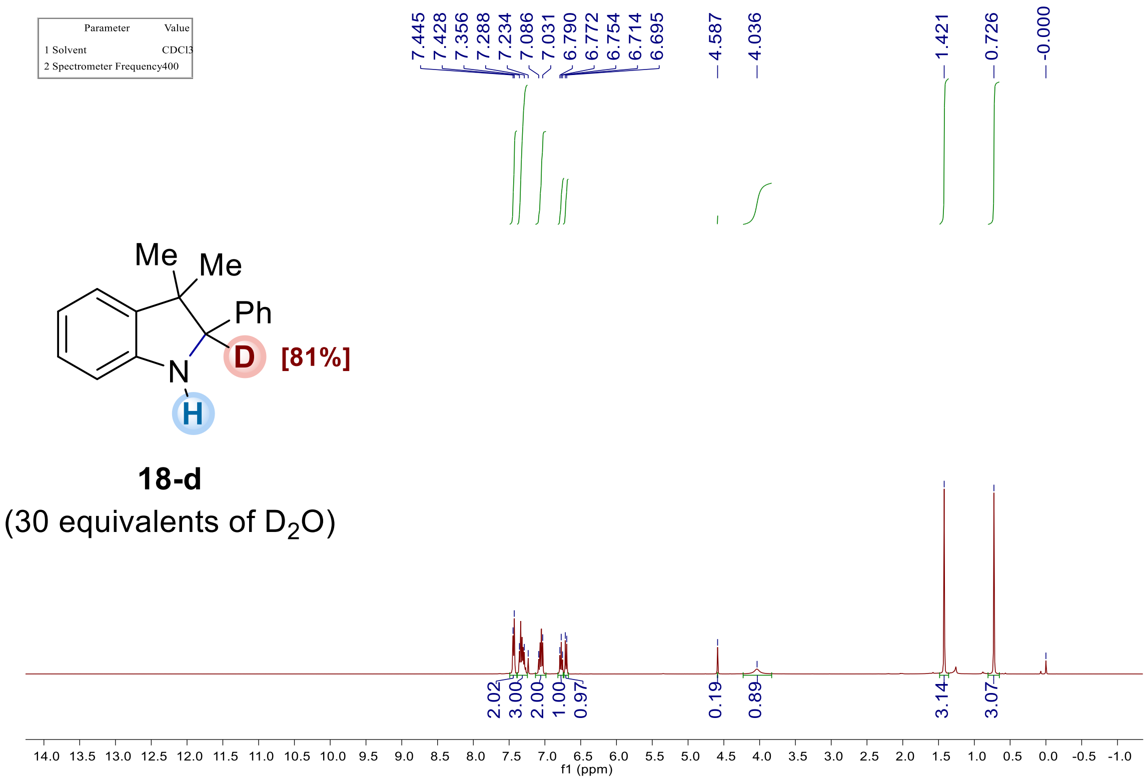

Supplementary Figure 29. ^{1}H NMR spectra of compound **18-d**~92% D (400 MHz, $CDCl_3$)

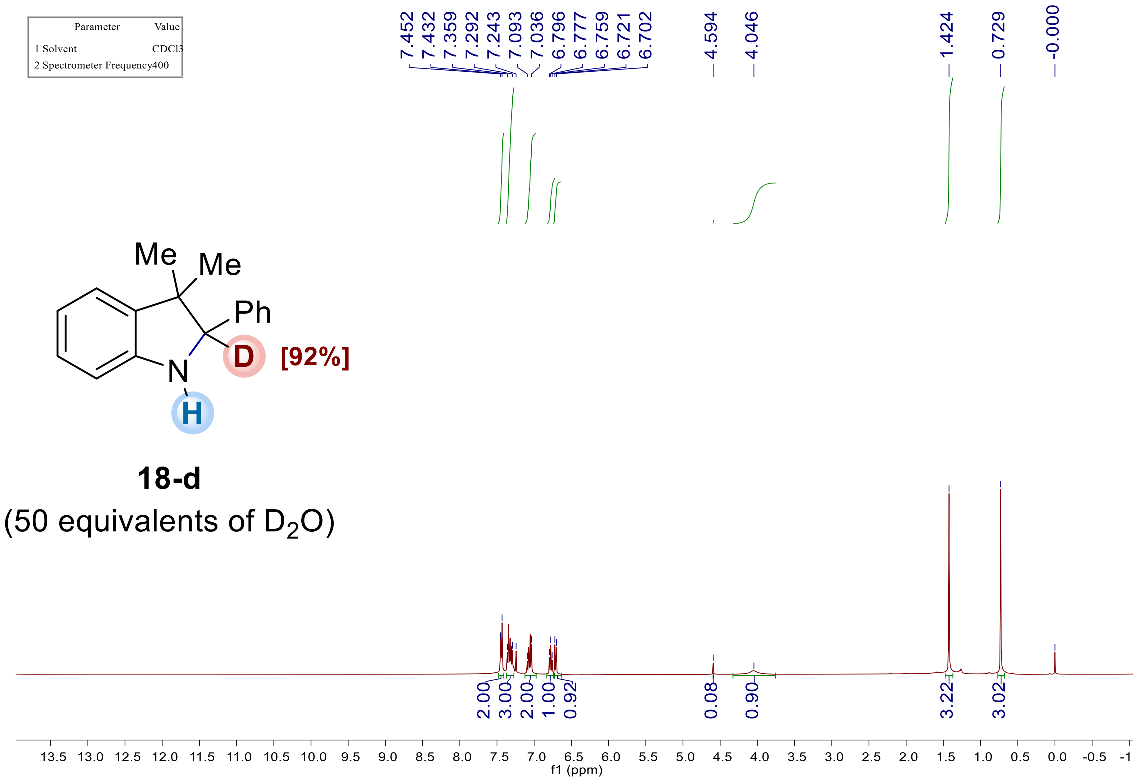

Supplementary Figure 30. ^{1}H NMR spectra of compound **8S** (400 MHz, $CDCl_3$)

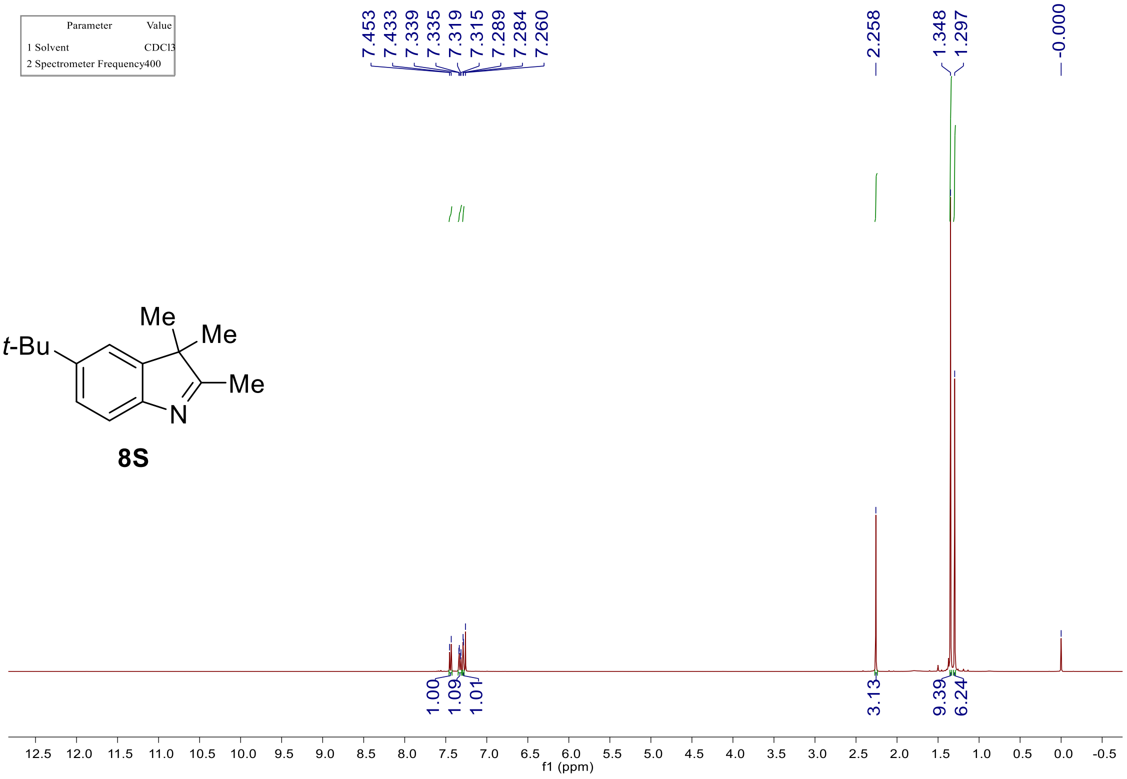

Supplementary Figure 31. ^{1}H NMR spectra of compound **10S** (400 MHz, $CDCl_3$)

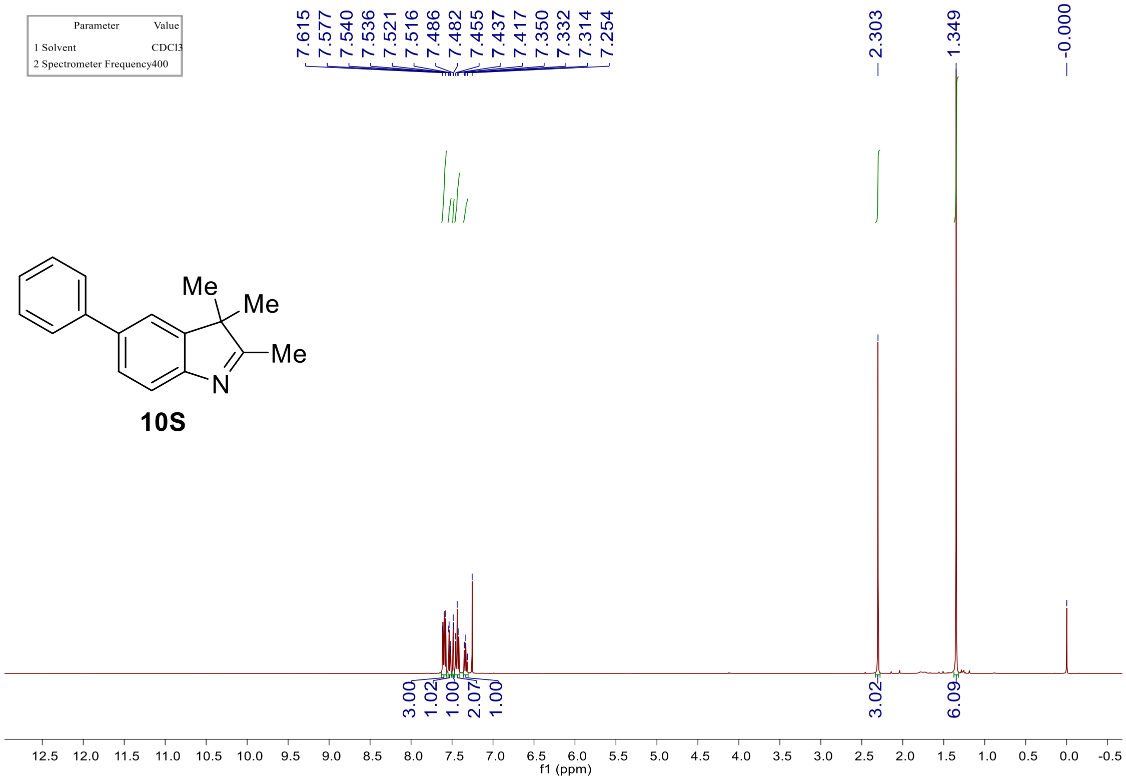

Supplementary Figure 32. ^{1}H NMR spectra of compound **13S** (400 MHz, $CDCl_3$)

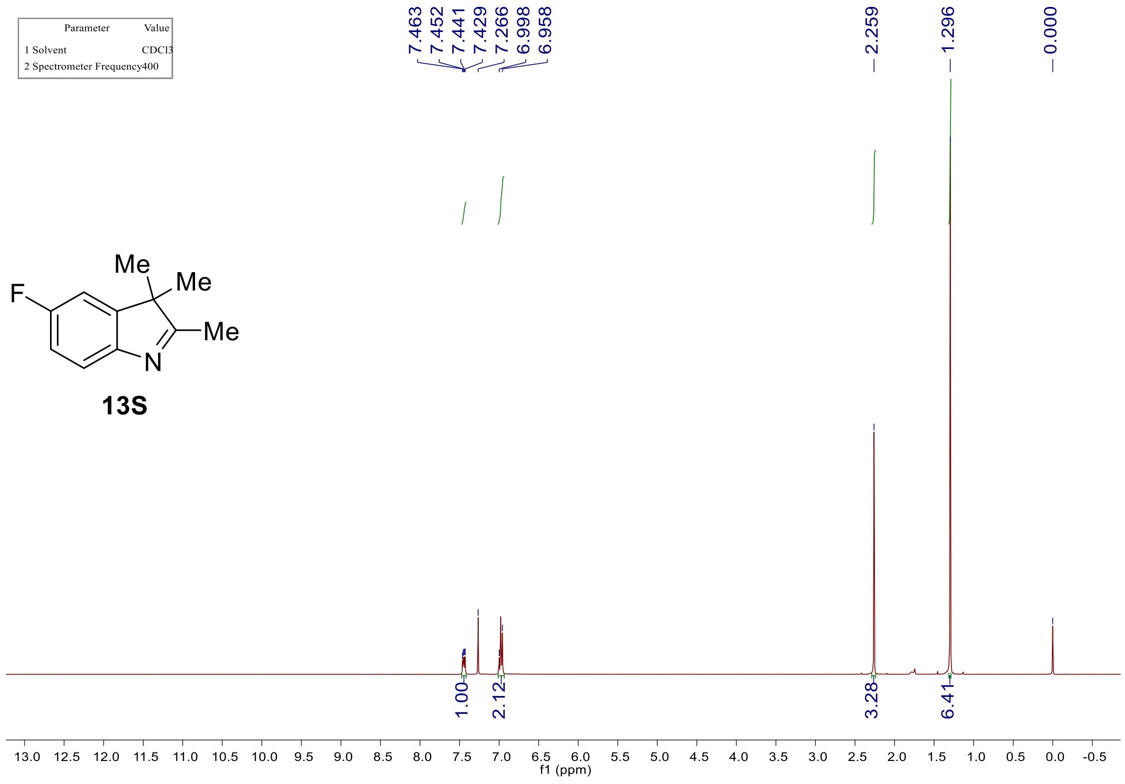

Supplementary Figure 33. ^{1}H NMR spectra of compound **14S** (400 MHz, $CDCl_3$)

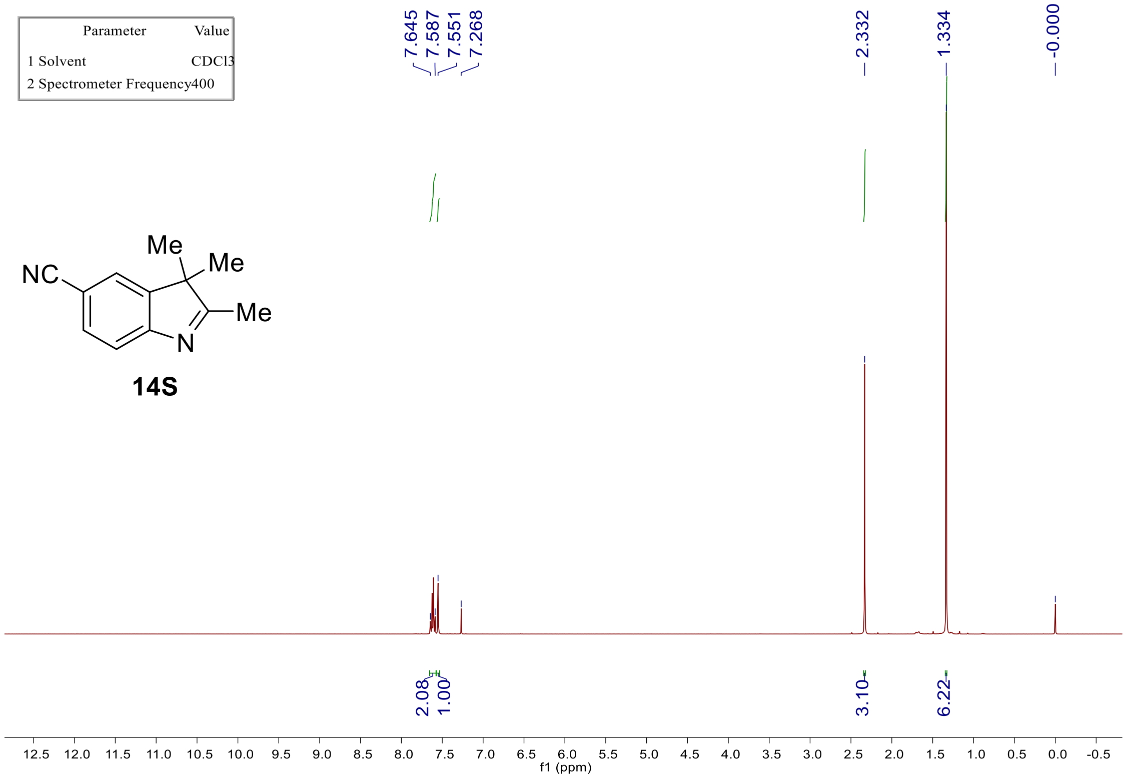

Supplementary Figure 34. ^{1}H NMR spectra of compound **16S** (400 MHz, $CDCl_3$)

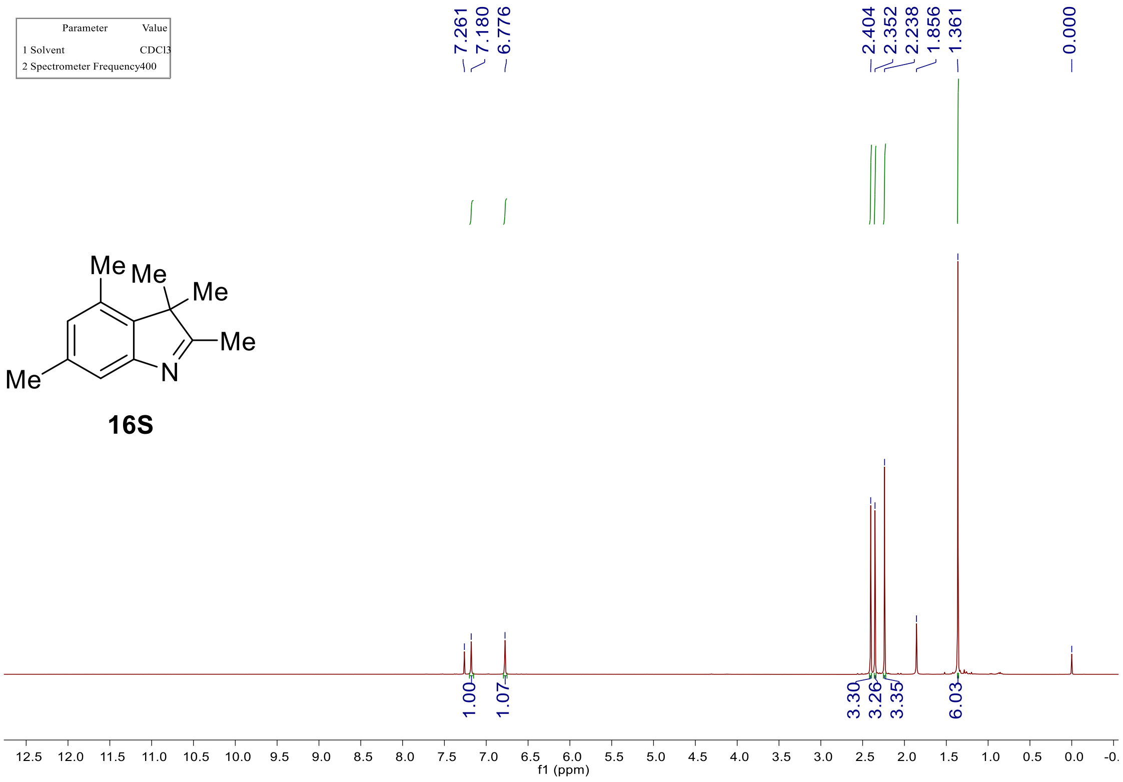

Supplementary Figure 35. ^{1}H NMR spectra of compound **17S** (400 MHz, $CDCl_3$)

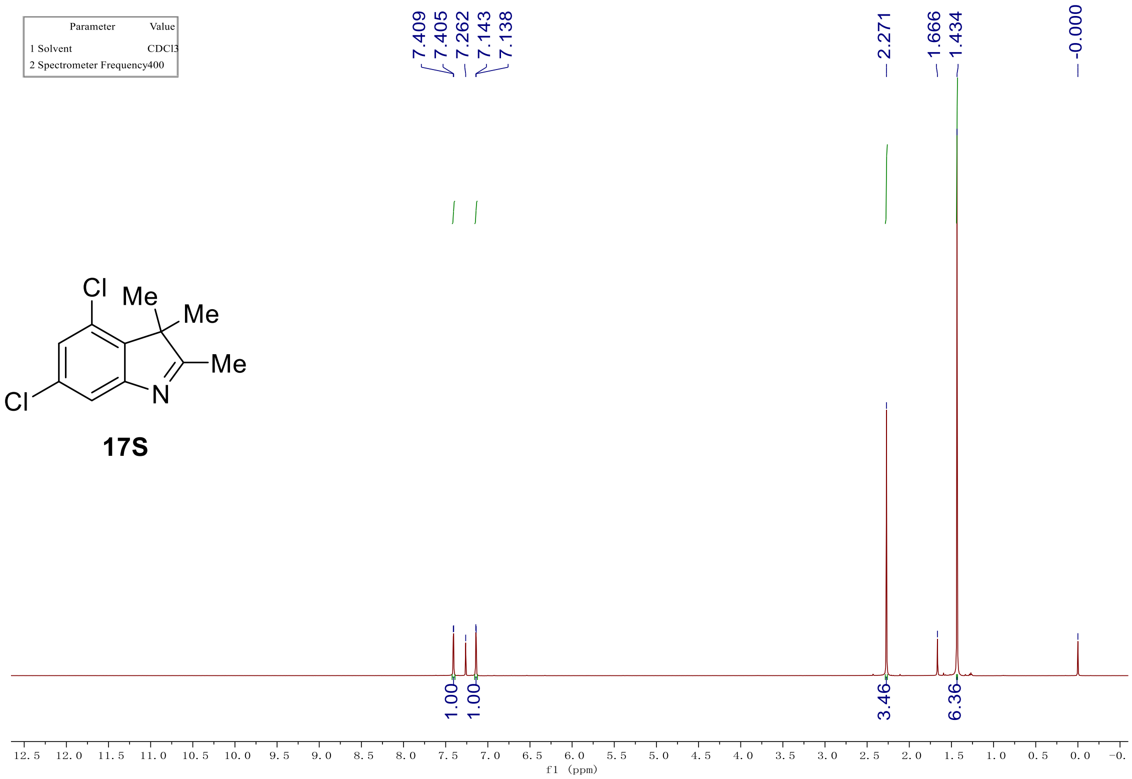

Supplementary Figure 36. ^{1}H NMR spectra of compound **18S** (400 MHz, $CDCl_3$)

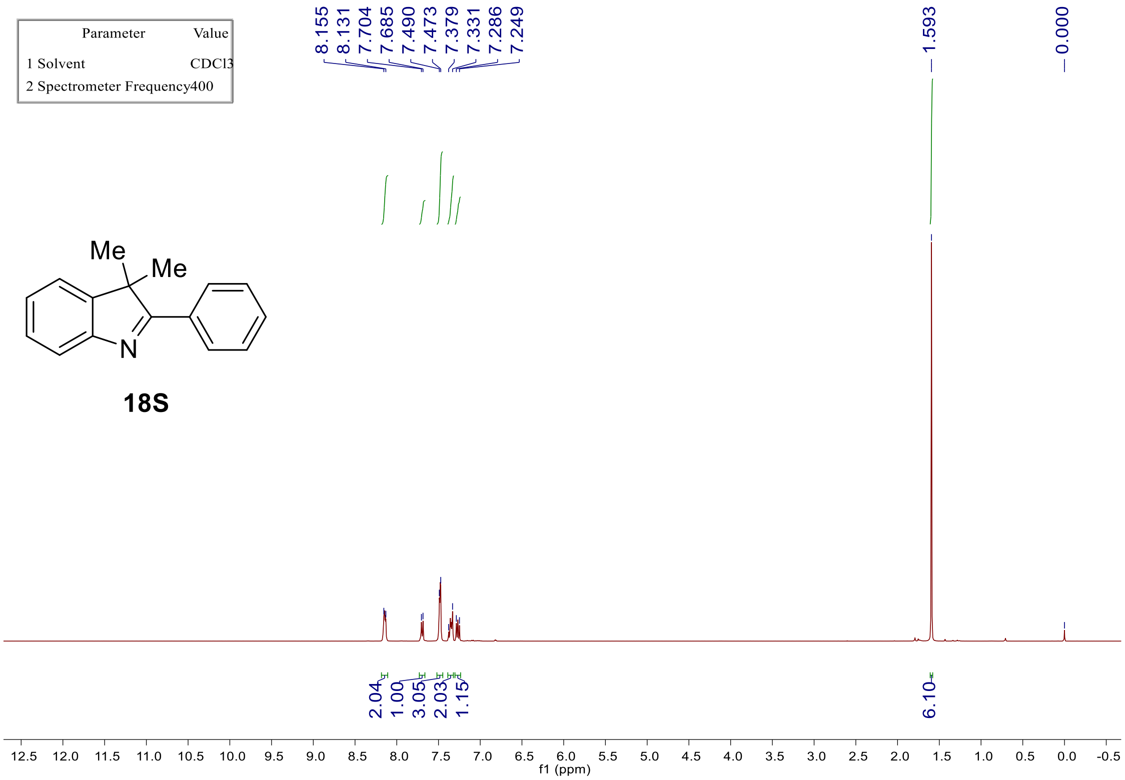

Supplementary Figure 37. ^{1}H NMR spectra of compound **19S** (400 MHz, $CDCl_3$)

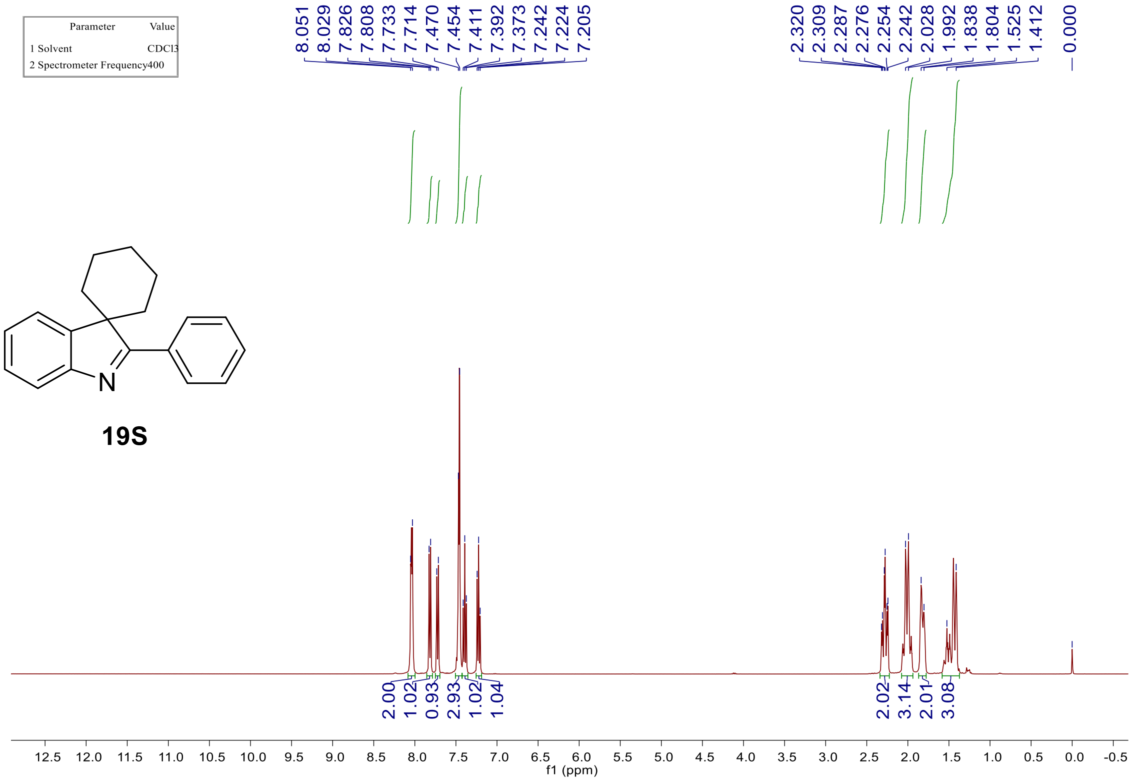

Supplementary Figure 38. ^{1}H NMR spectra of compound **20S** (400 MHz, $CDCl_3$)

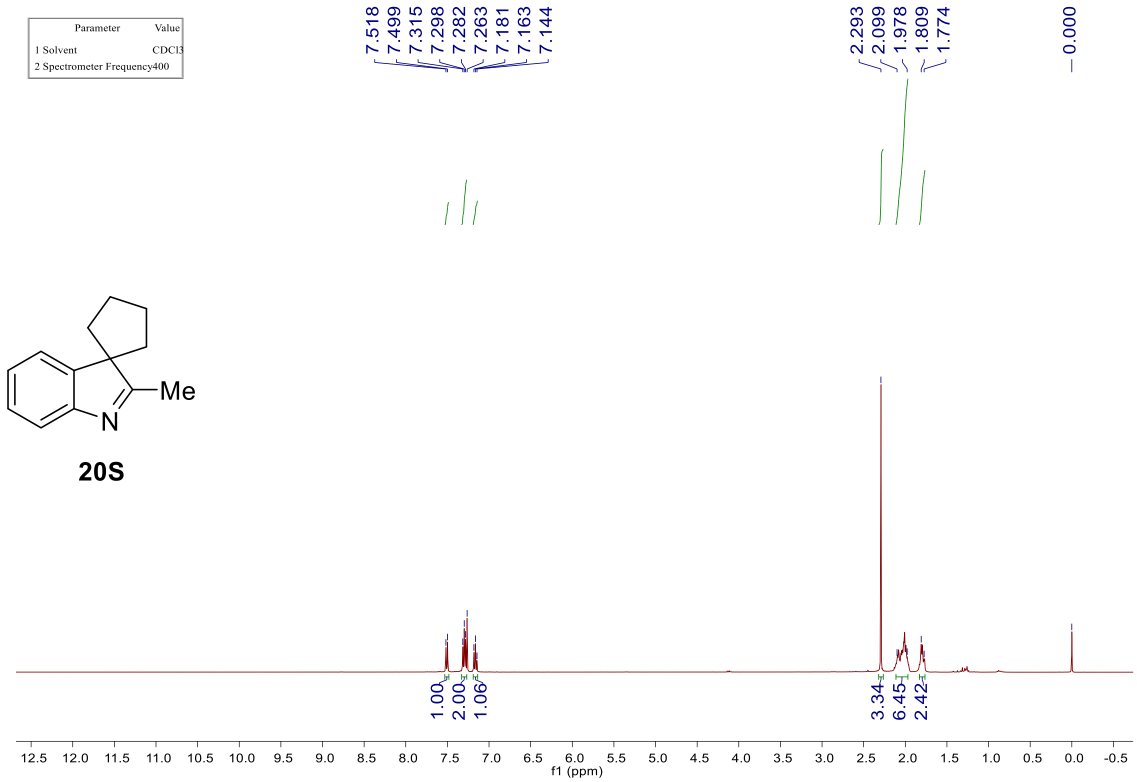

Supplementary Figure 39. ^{1}H NMR spectra of compound **21S** (400 MHz, $CDCl_3$)

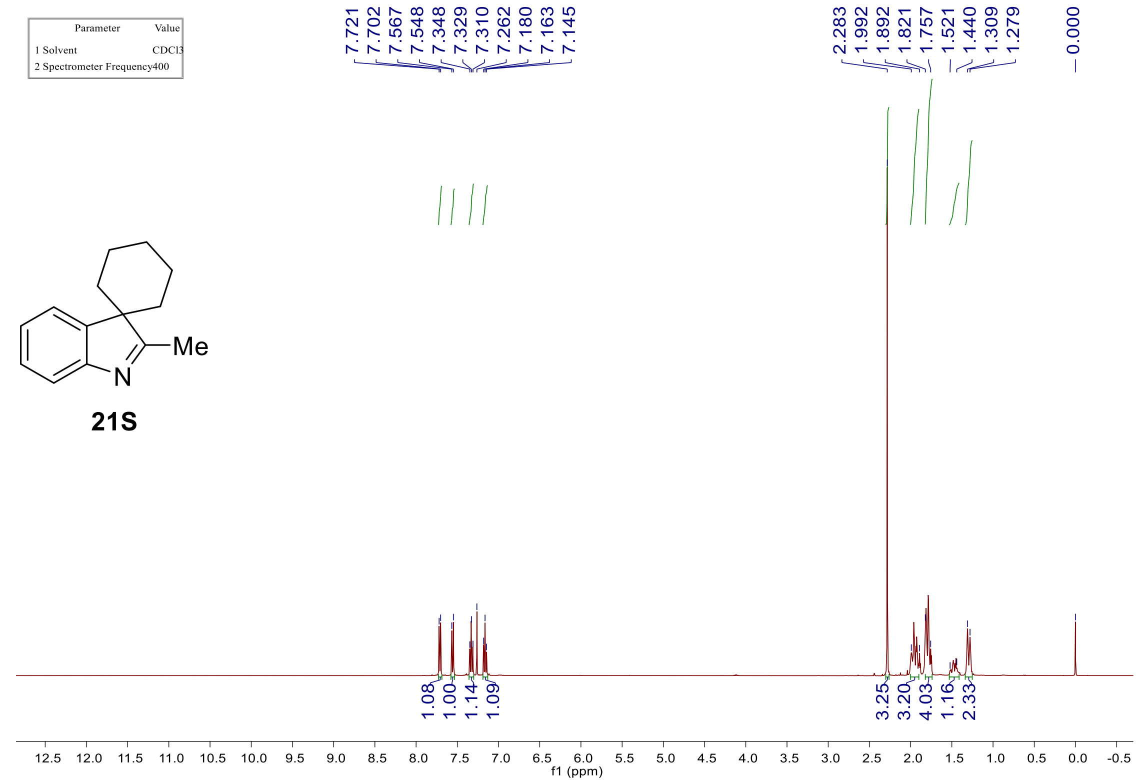

Supplementary Figure 40. ^{1}H NMR spectra of compound **22S** (400 MHz, $CDCl_3$)

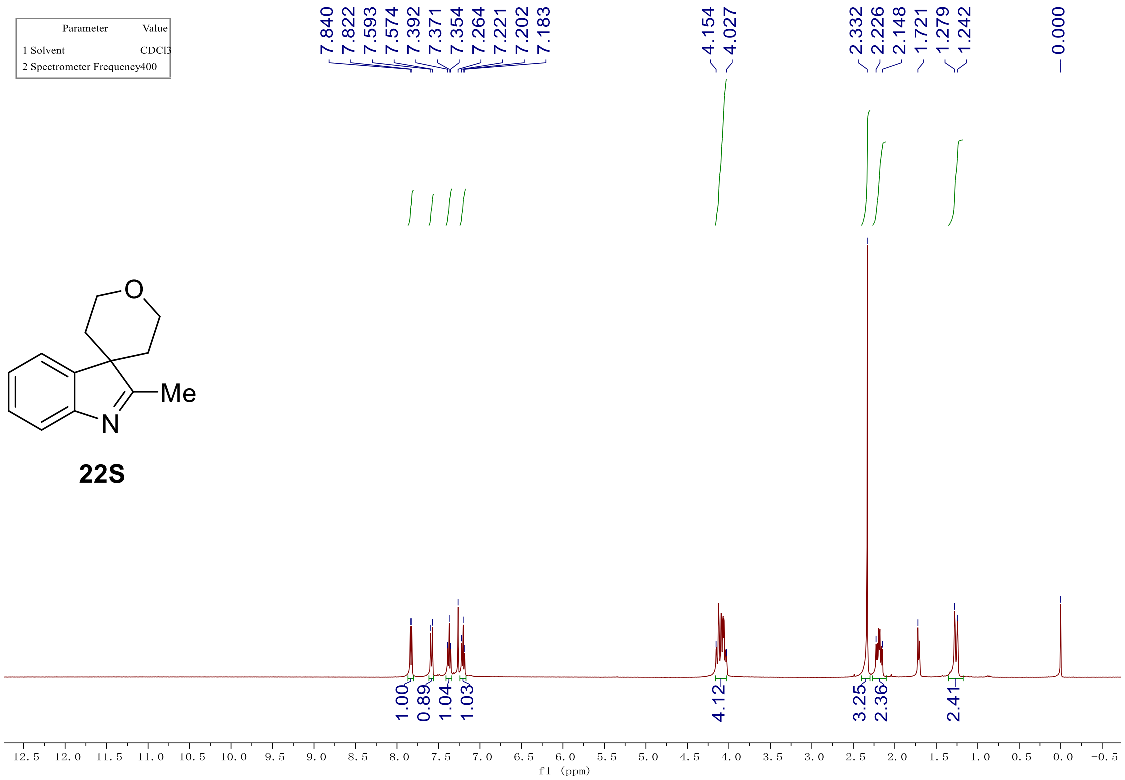

Supplementary Figure 41. ^{1}H NMR spectra of compound **23S** (400 MHz, $CDCl_3$)

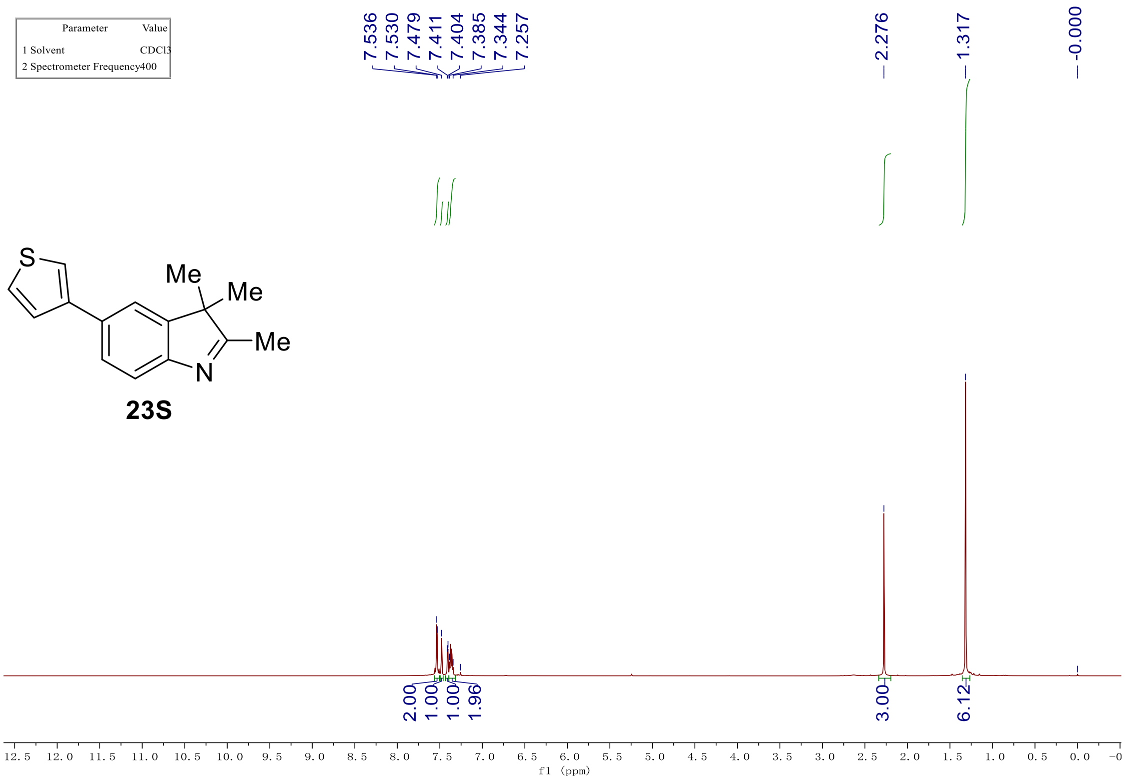

Supplementary Figure 42. ^{13}C NMR spectra of compound **23S** (101 MHz, $CDCl_3$)

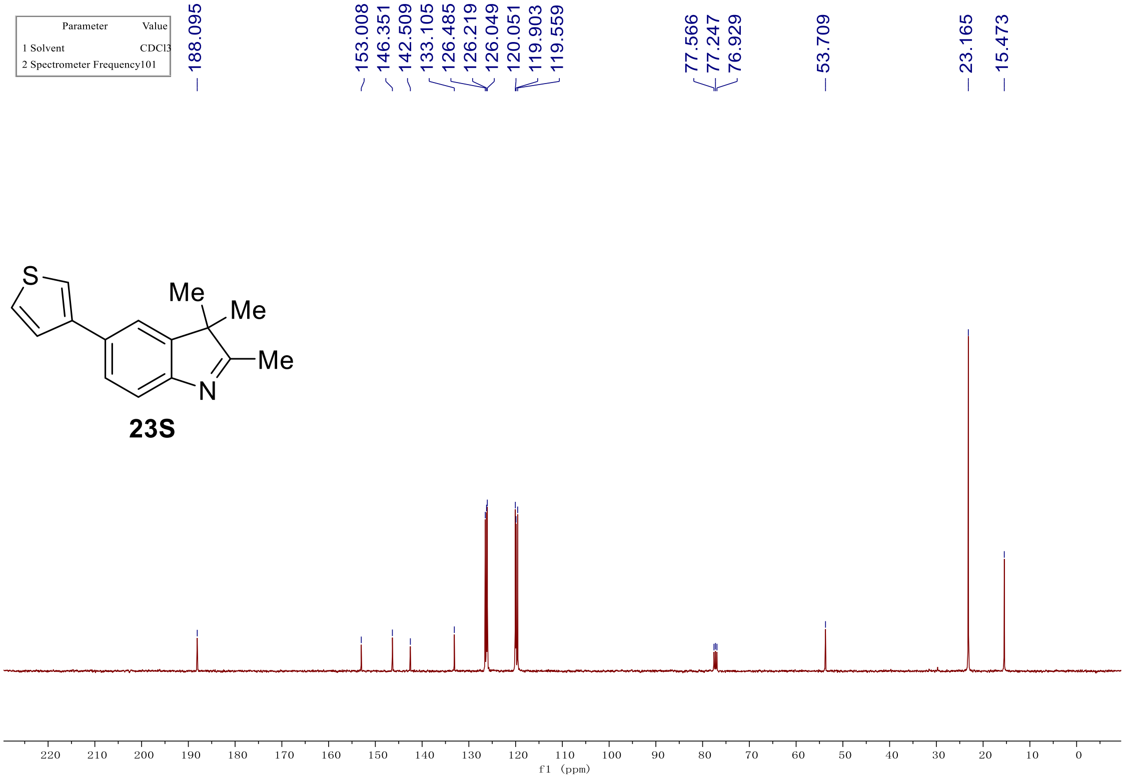

Supplementary Figure 43. ^{1}H NMR spectra of compound **24S** (400 MHz, $CDCl_3$)

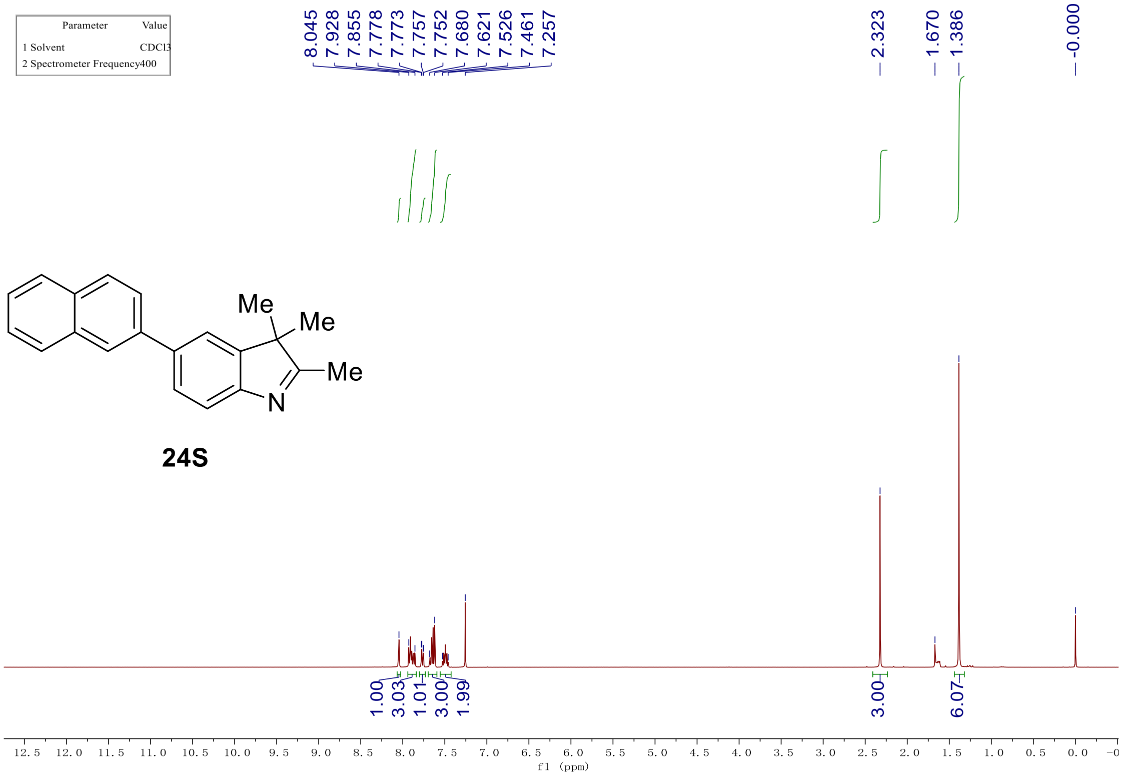

Supplementary Figure 44. ^{13}C NMR spectra of compound **24S** (101 MHz, $CDCl_3$)

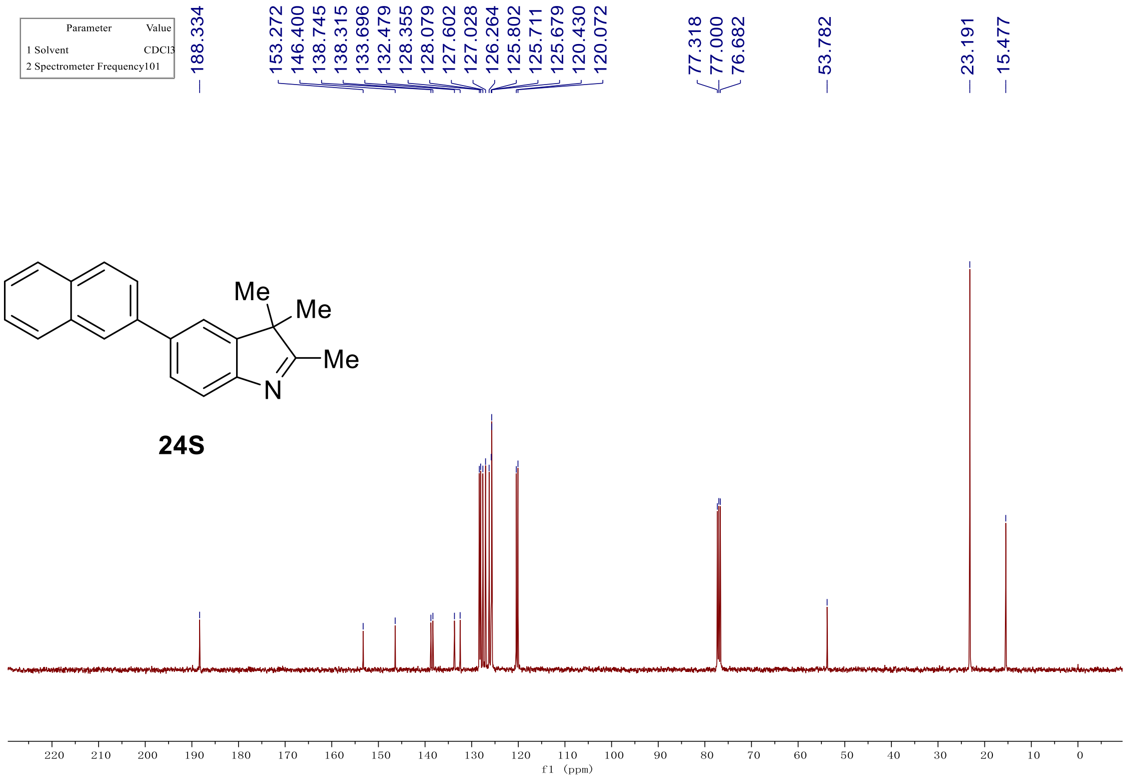

Supplementary Figure 45. ^{1}H NMR spectra of compound **43S** (400 MHz, $CDCl_3$)

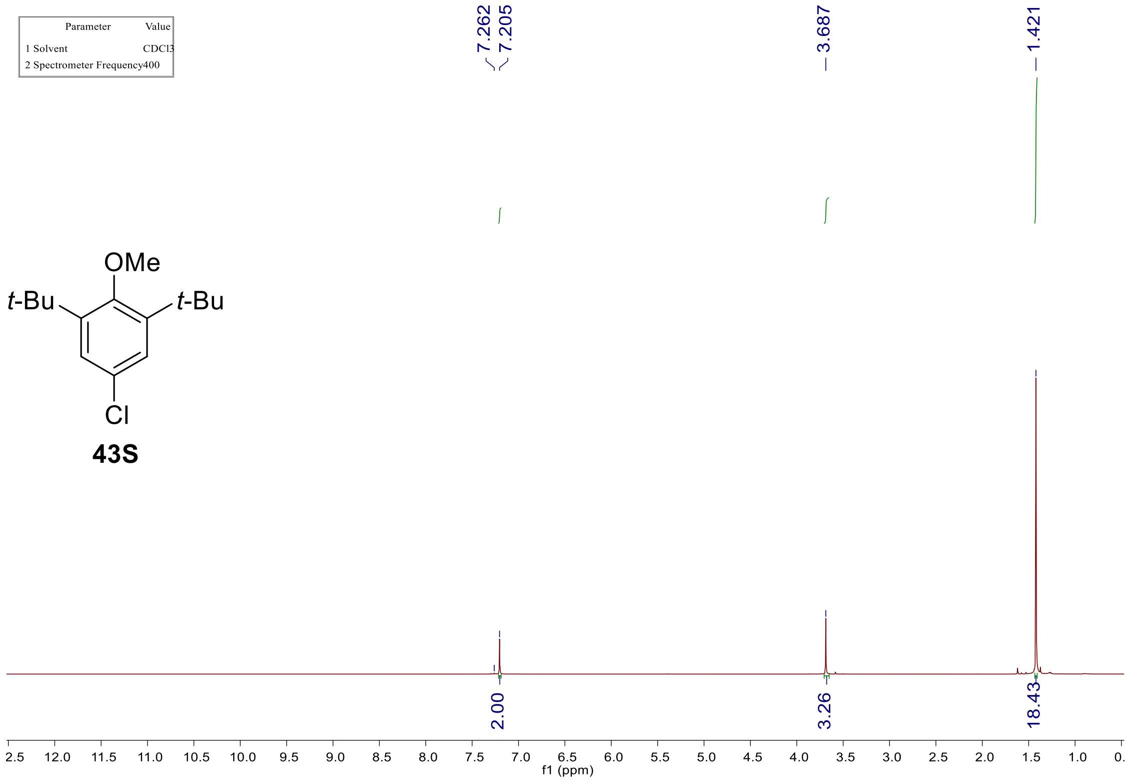

Supplementary Figure 46. ^{1}H NMR spectra of compound **6** (400 MHz, $CDCl_3$)

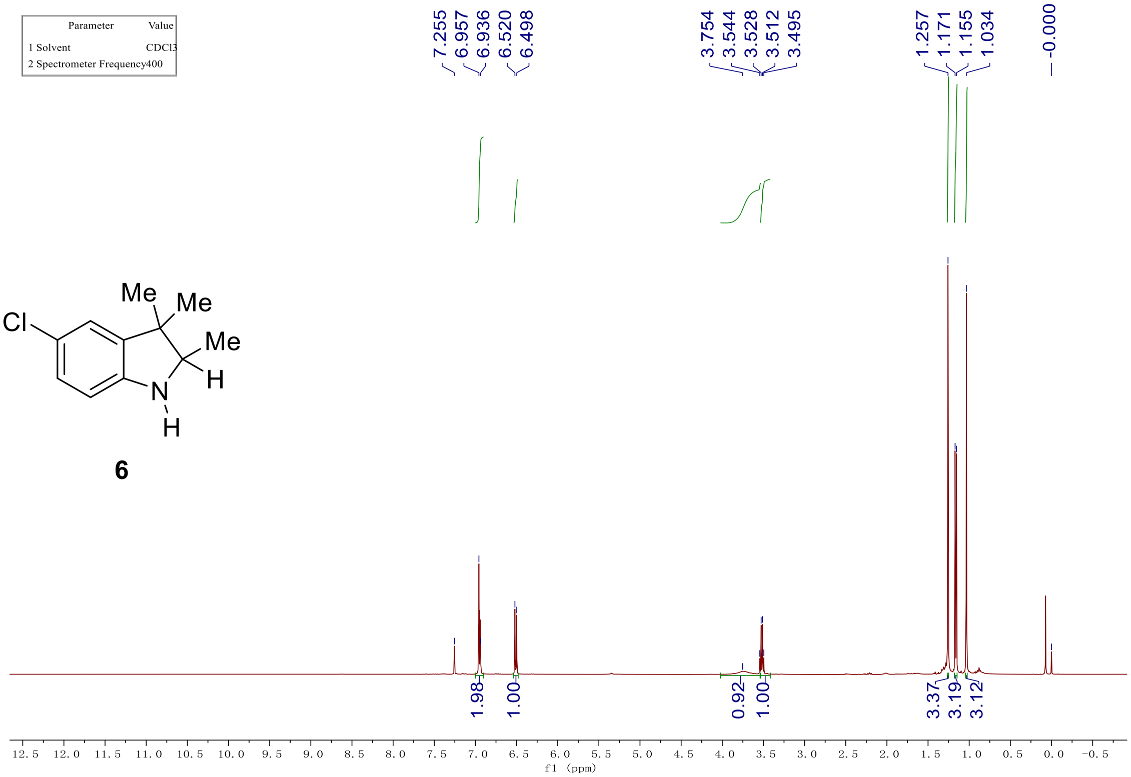

Supplementary Figure 47. ^{1}H NMR spectra of compound **7** (400 MHz, $CDCl_3$)

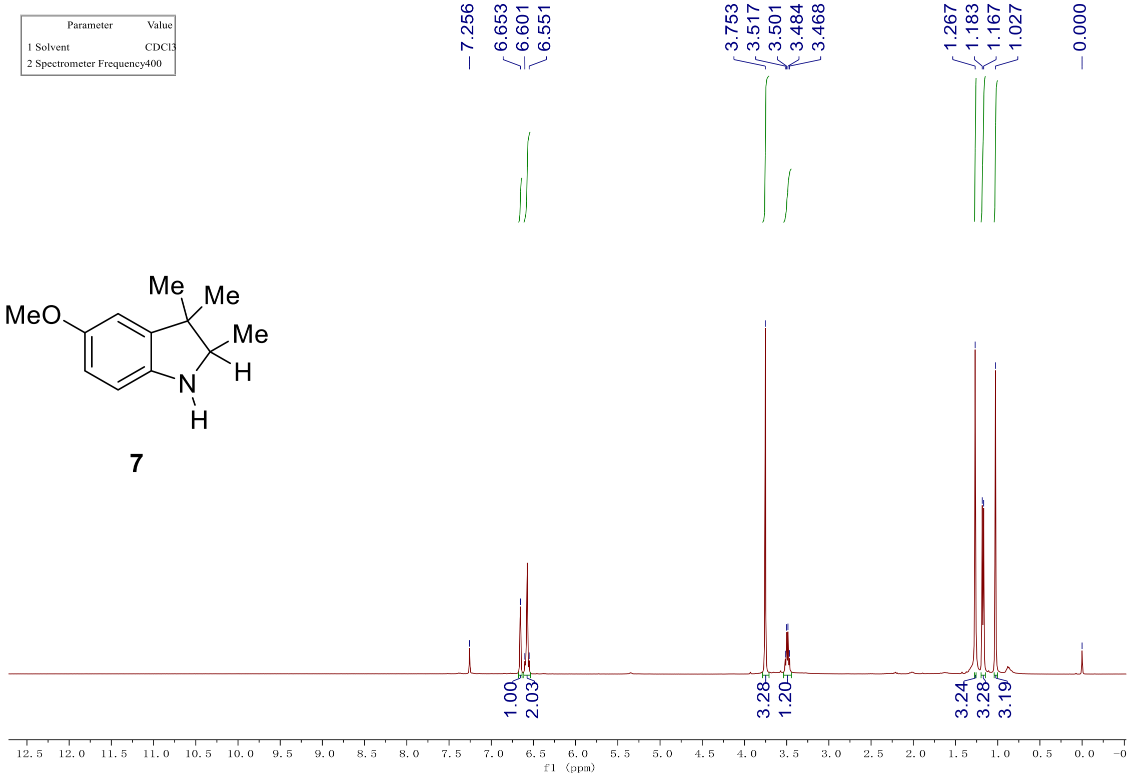

Supplementary Figure 48. ^{1}H NMR spectra of compound **8** (400 MHz, $CDCl_3$)

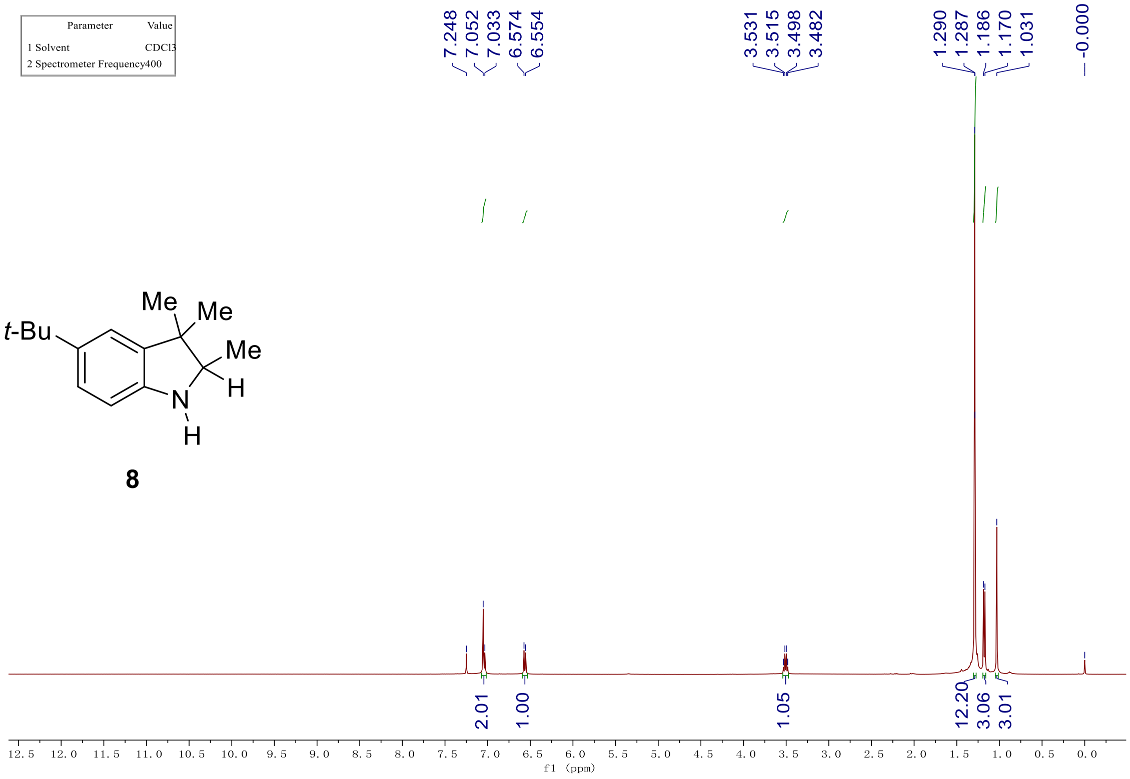

Supplementary Figure 49. ^{13}C NMR spectra of compound **8** (101 MHz, $CDCl_3$)

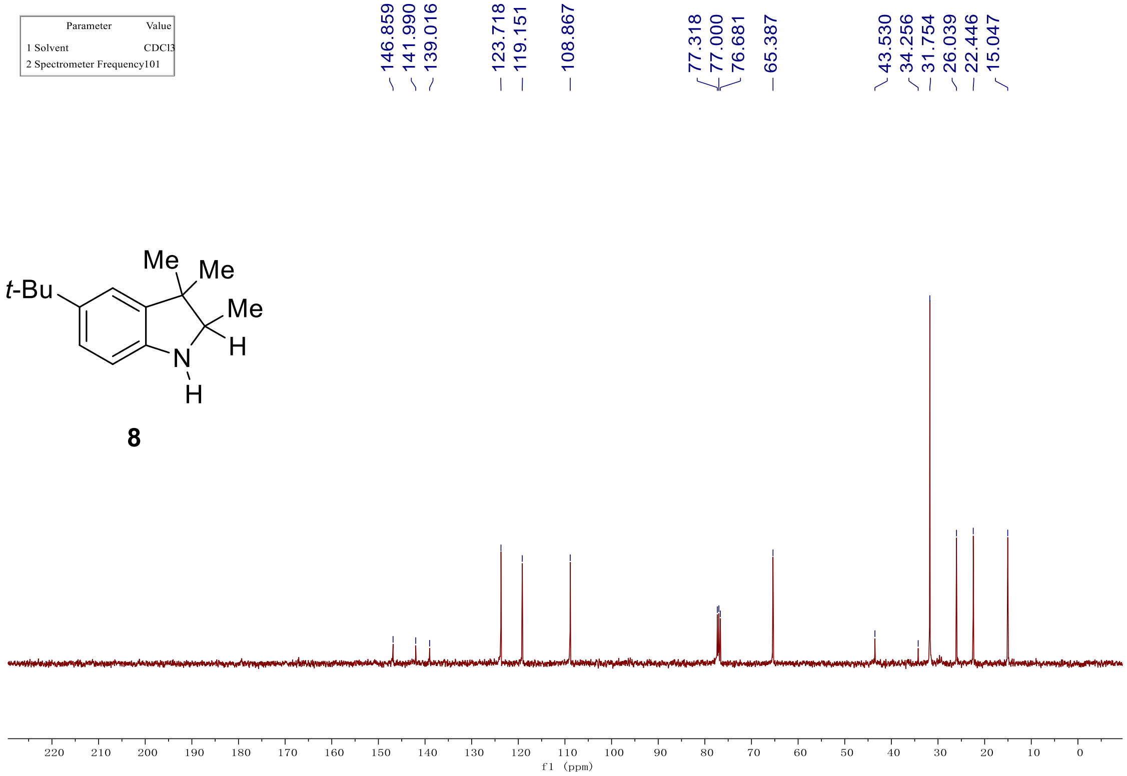

Supplementary Figure 50. ^{1}H NMR spectra of compound **9** (400 MHz, $CDCl_3$)

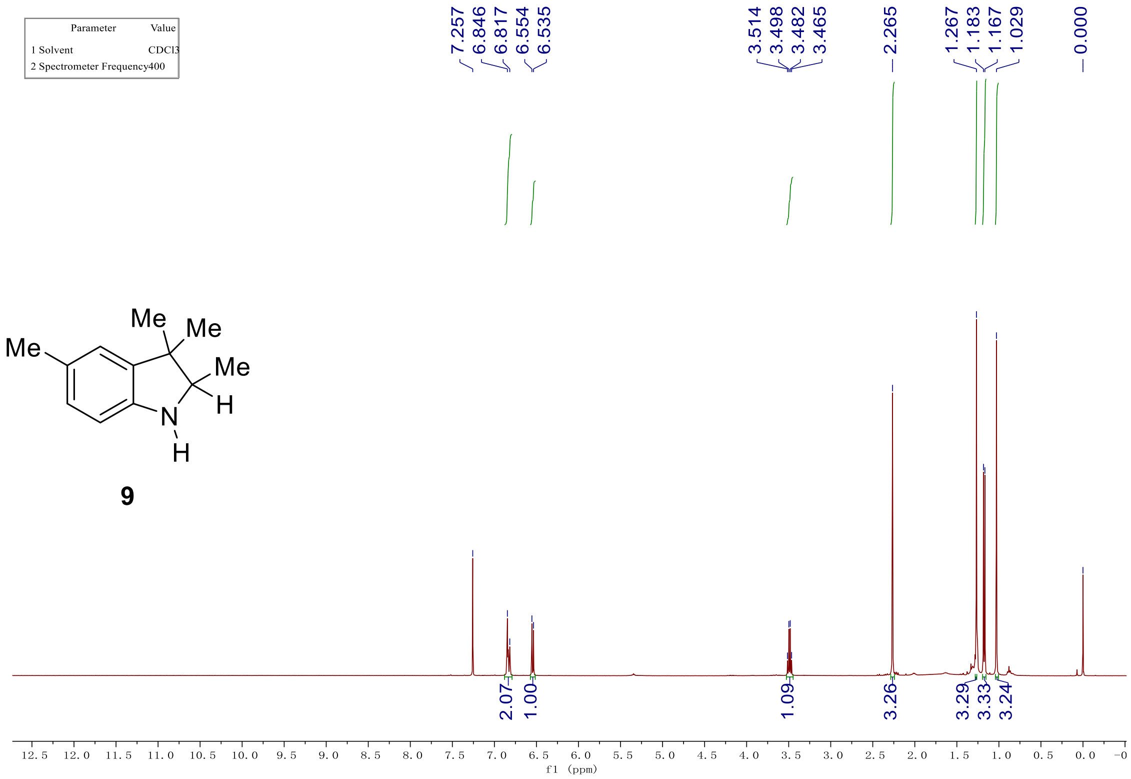

Supplementary Figure 51. ^{1}H NMR spectra of compound **10** (400 MHz, $CDCl_3$)

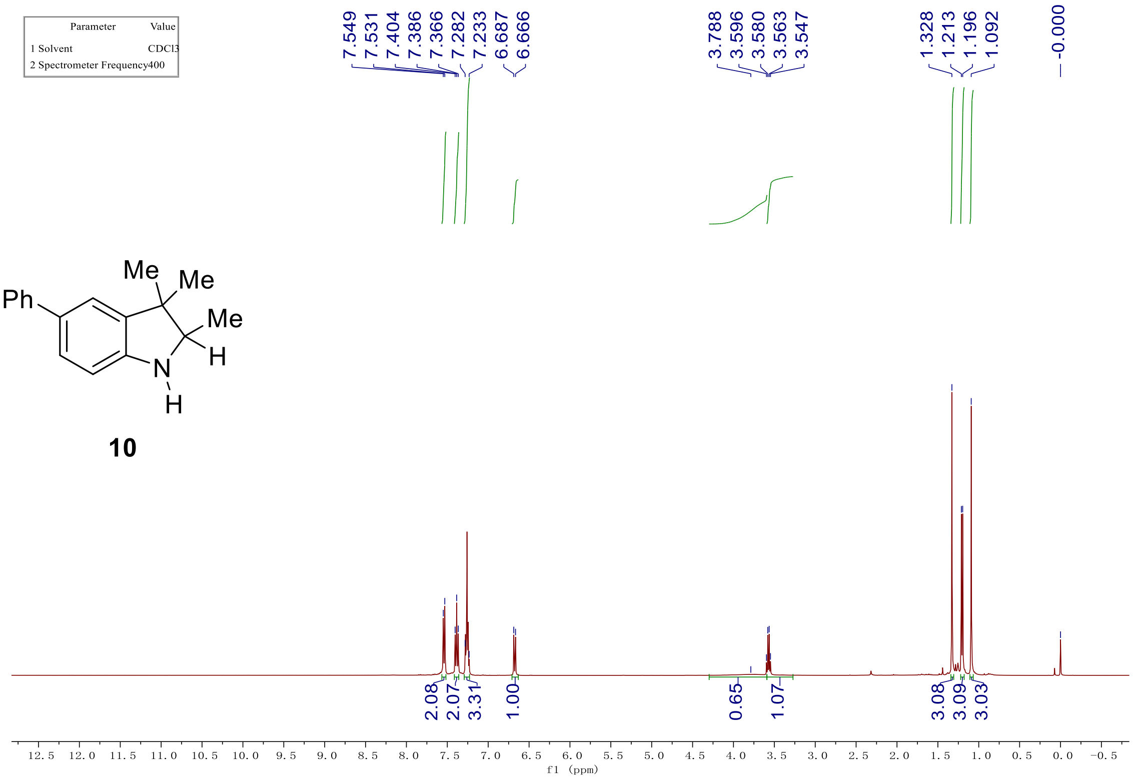

Supplementary Figure 52. ^{13}C NMR spectra of compound **10** (101 MHz, $CDCl_3$)

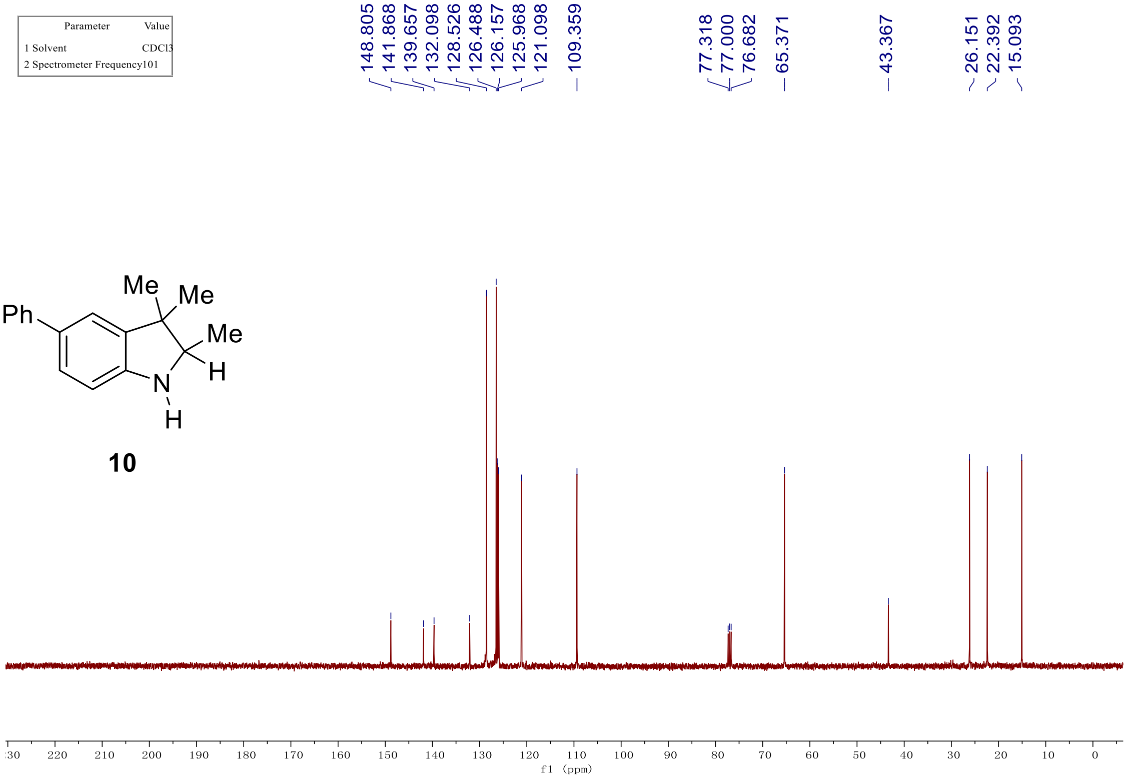

Supplementary Figure 53. ^{1}H NMR spectra of compound **11** (400 MHz, $CDCl_3$)

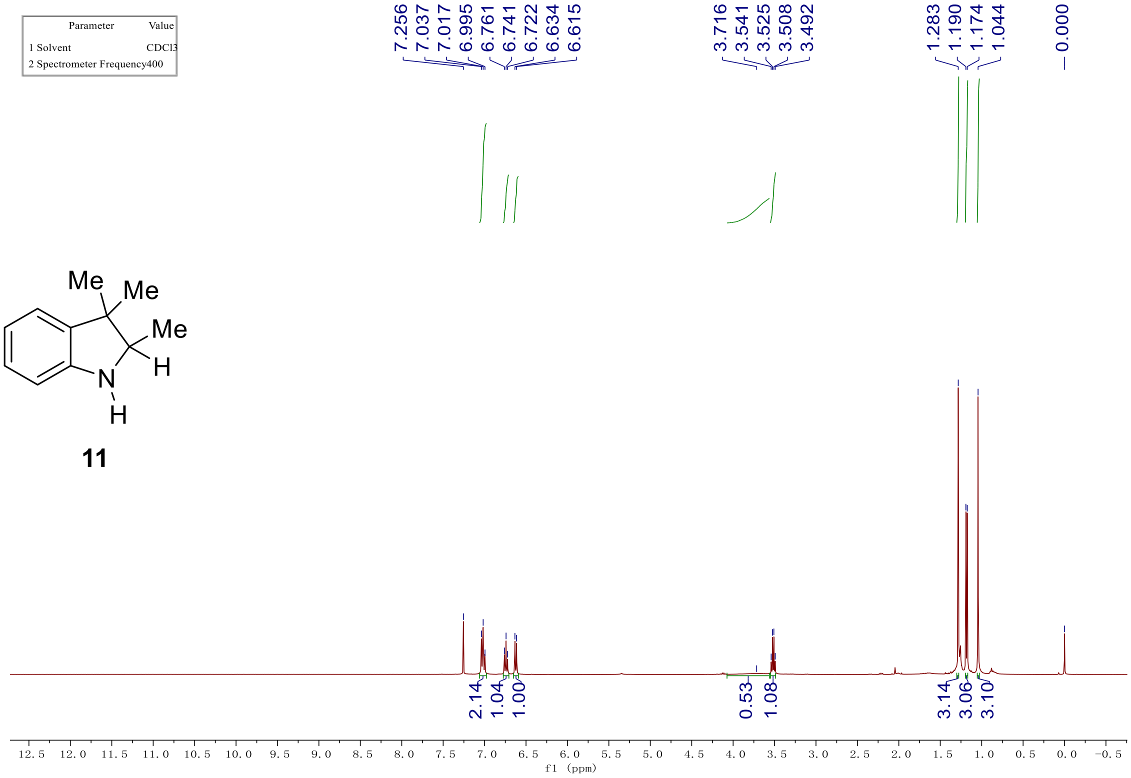

Supplementary Figure 54. ^{1}H NMR spectra of compound **12** (400 MHz, $CDCl_3$)

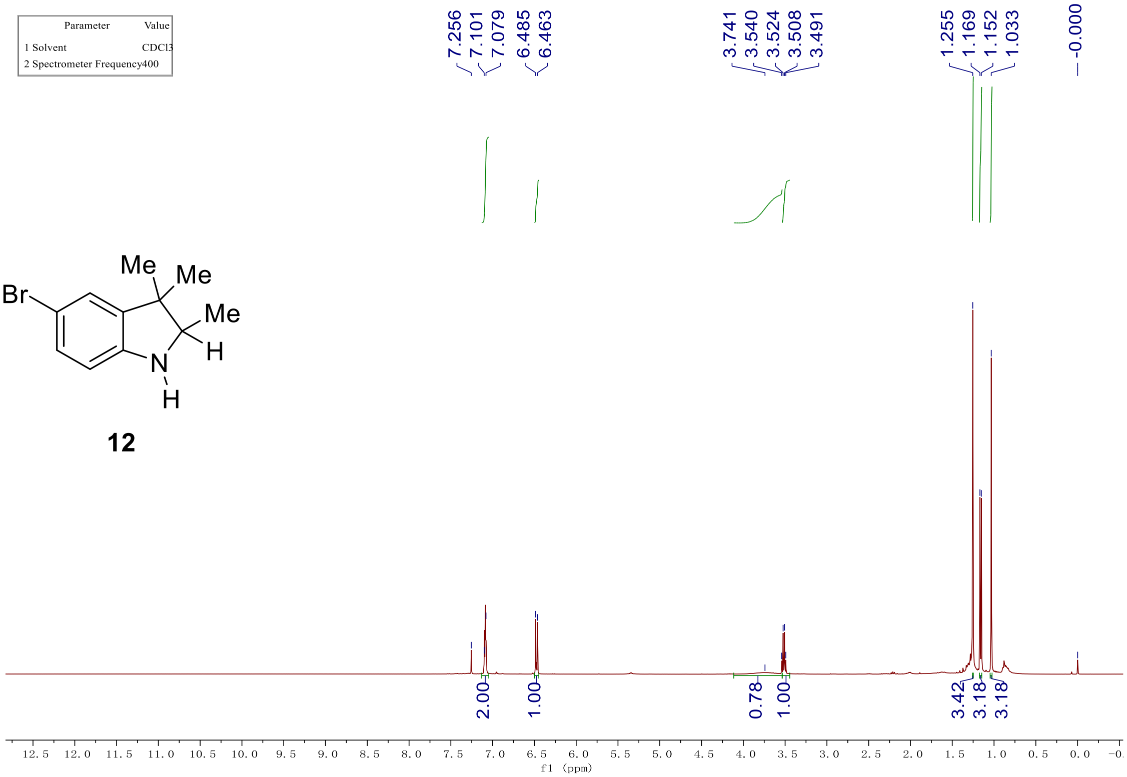

Supplementary Figure 55. ^{1}H NMR spectra of compound **13** (400 MHz, $CDCl_3$)

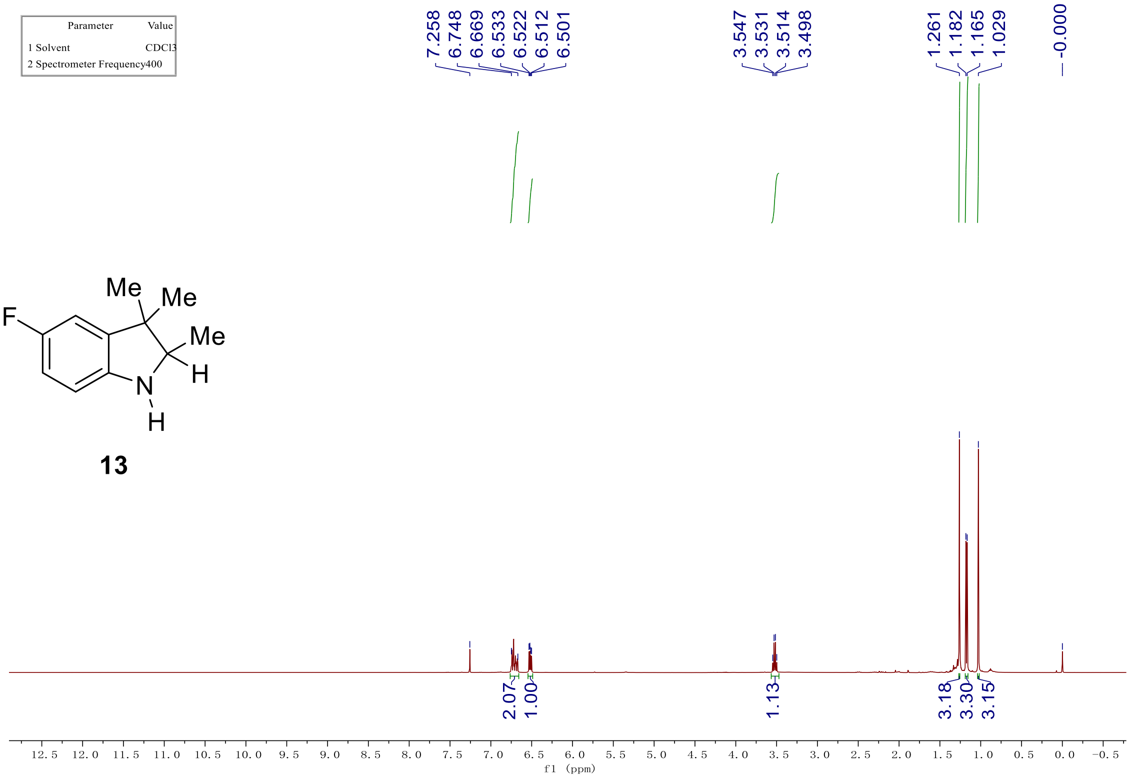

Supplementary Figure 56. ^{1}H NMR spectra of compound **14** (400 MHz, $CDCl_3$)

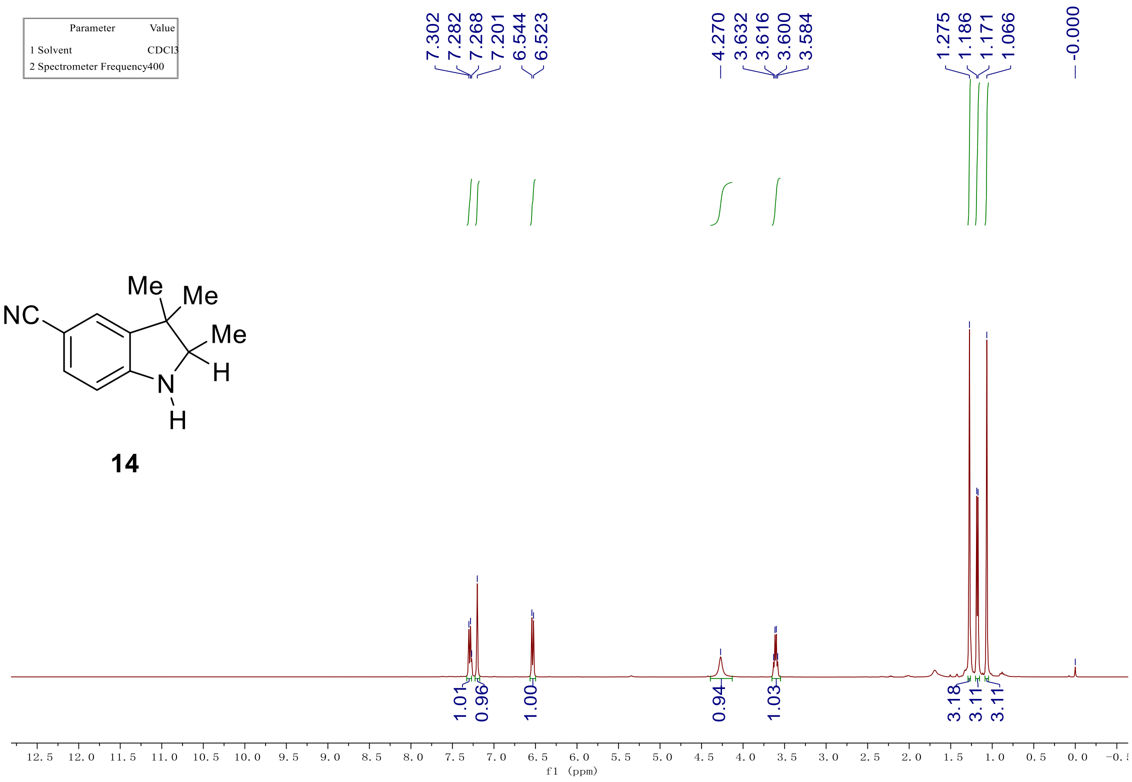

Supplementary Figure 57. ^{13}C NMR spectra of compound **14** (101 MHz, $CDCl_3$)

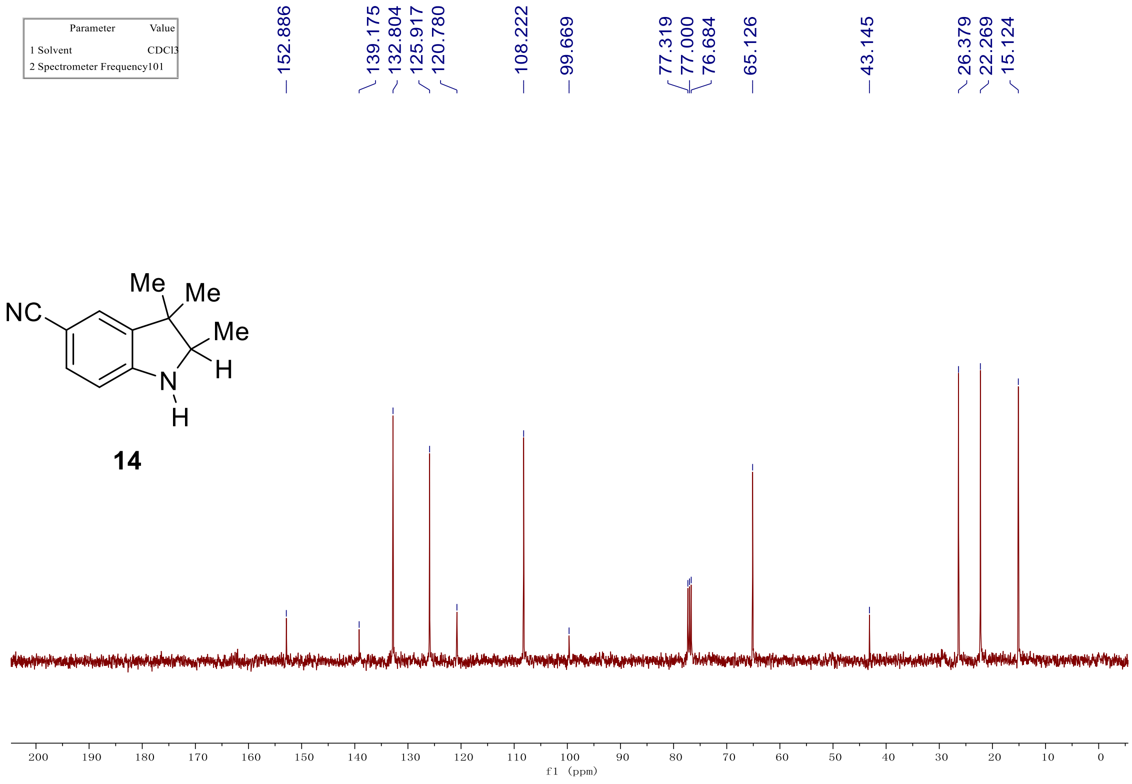

Supplementary Figure 58. ^{1}H NMR spectra of compound **15** (400 MHz, $CDCl_3$)

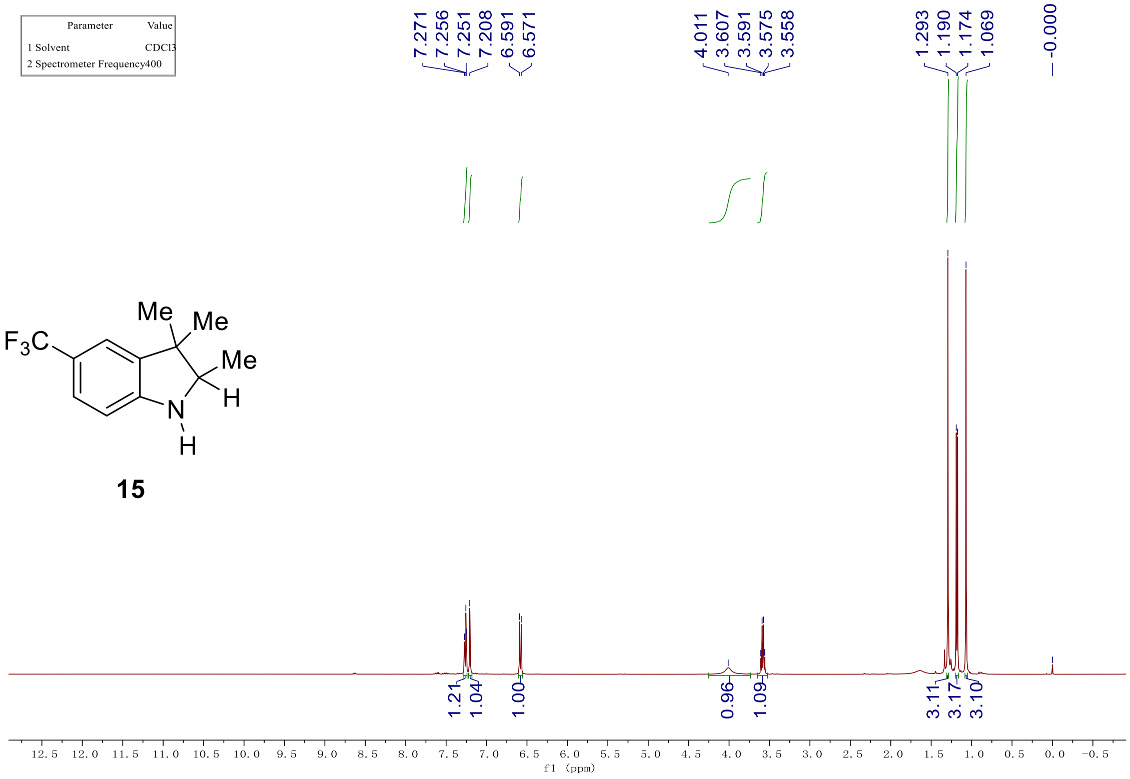

Supplementary Figure 59. ^{1}H NMR spectra of compound **16** (400 MHz, $CDCl_3$)

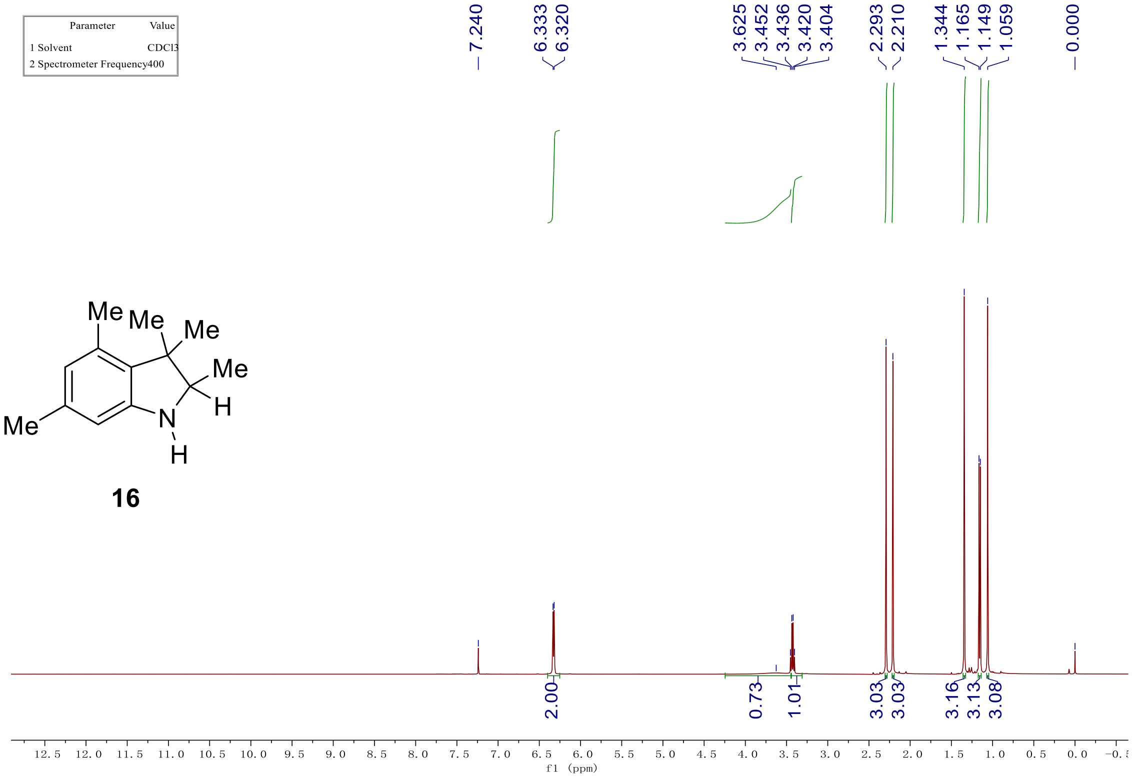

Supplementary Figure 60. ^{13}C NMR spectra of compound **16** (101 MHz, $CDCl_3$)

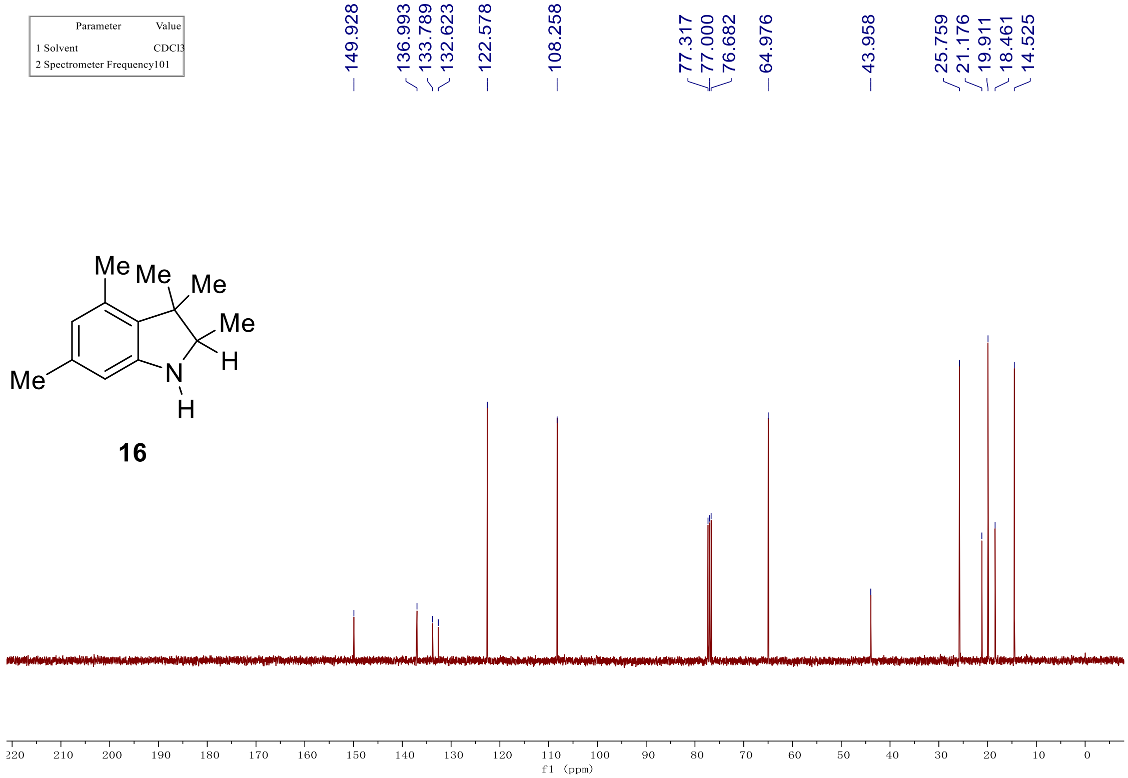

Supplementary Figure 61. ^{1}H NMR spectra of compound **17** (400 MHz, $CDCl_3$)

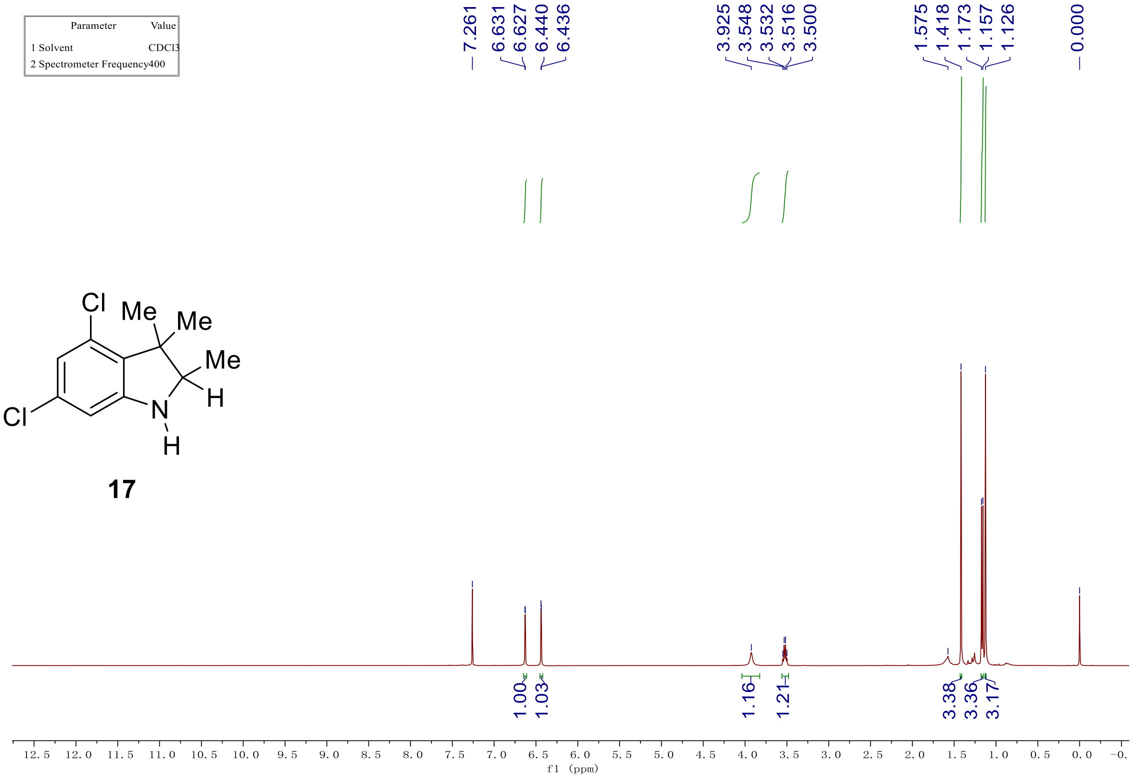

Supplementary Figure 62. ^{13}C NMR spectra of compound **17** (101 MHz, $CDCl_3$)

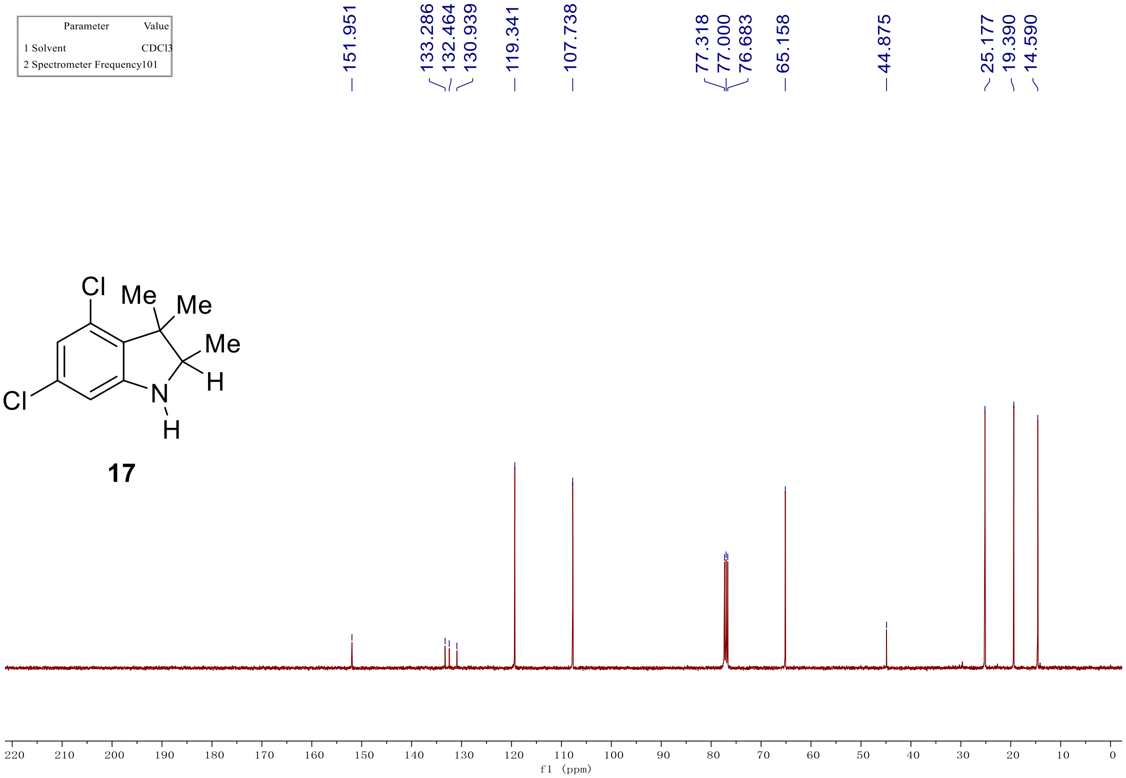

Supplementary Figure 63. ^{1}H NMR spectra of compound **18** (400 MHz, $CDCl_3$)

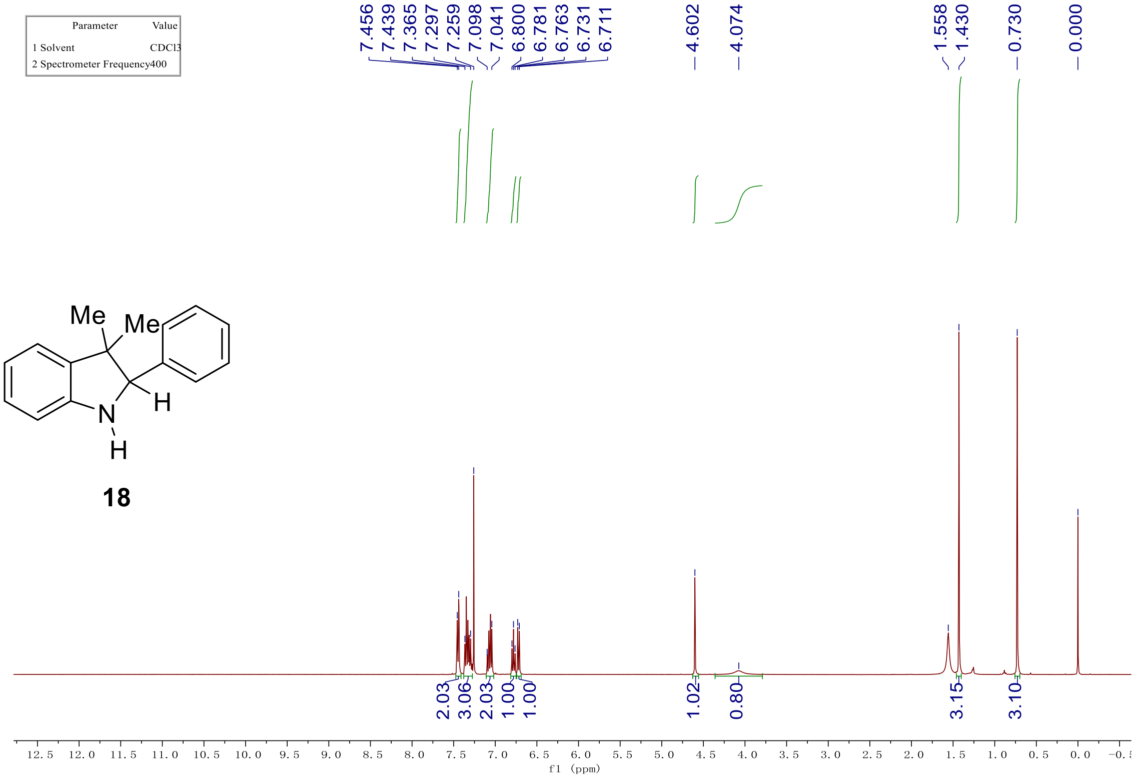

Supplementary Figure 64. ^{1}H NMR spectra of compound **19** (400 MHz, $CDCl_3$)

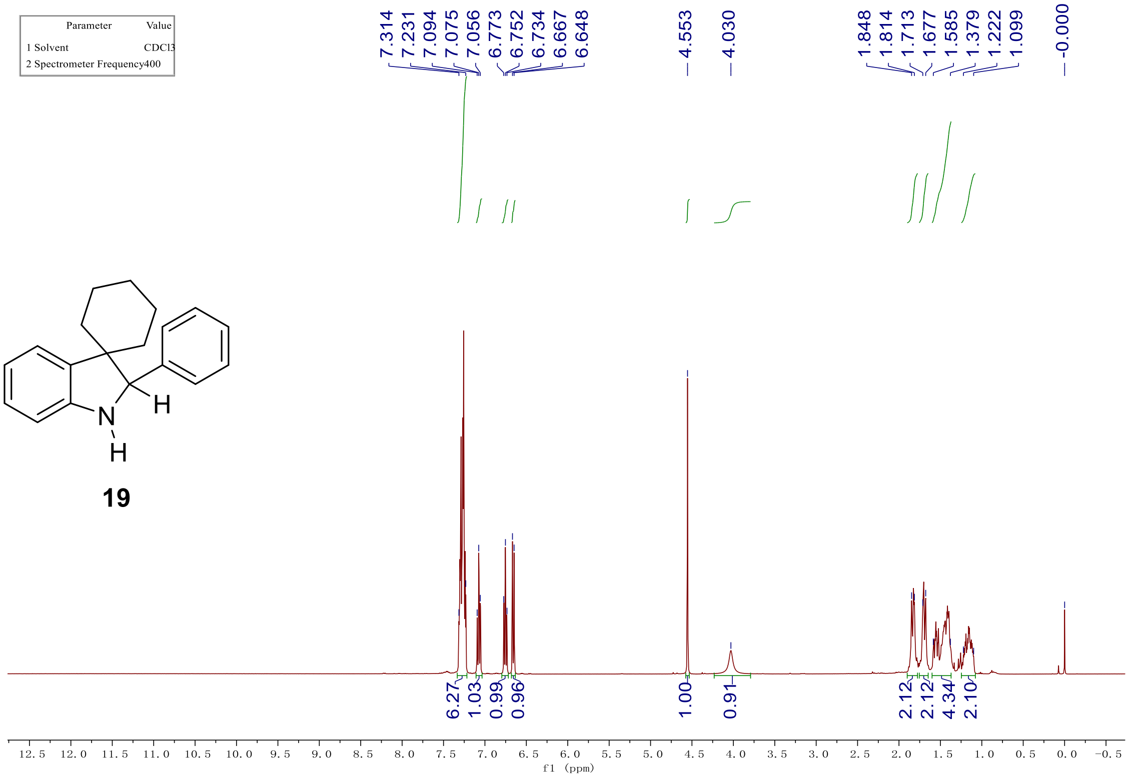

Supplementary Figure 65. ^{1}H NMR spectra of compound **20** (400 MHz, $CDCl_3$)

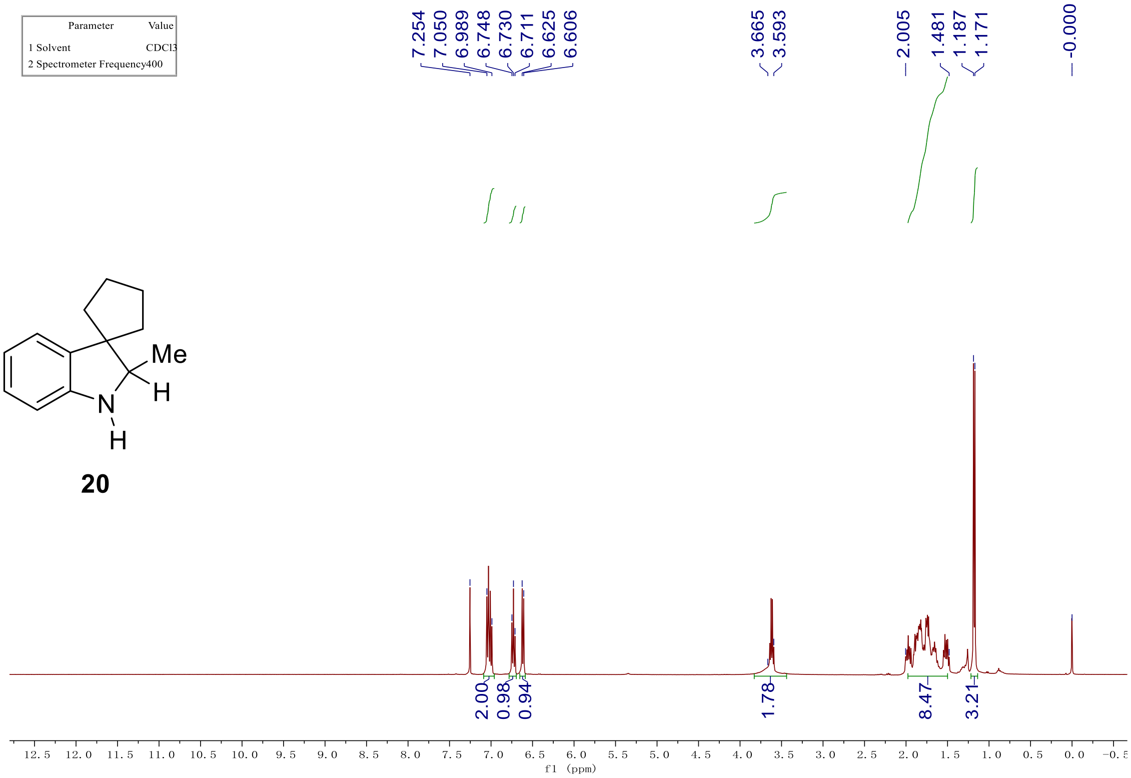

Supplementary Figure 66. ^{1}H NMR spectra of compound **21** (400 MHz, $CDCl_3$)

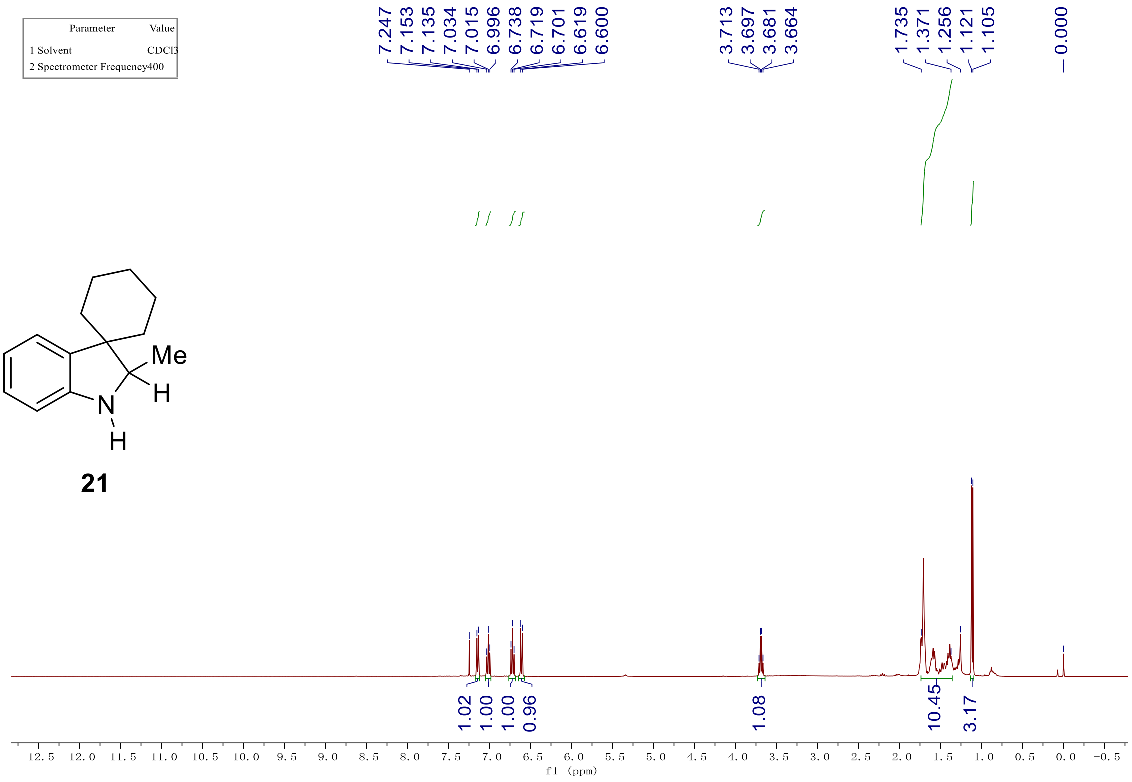

Supplementary Figure 67. ^{1}H NMR spectra of compound **22** (400 MHz, $CDCl_3$)

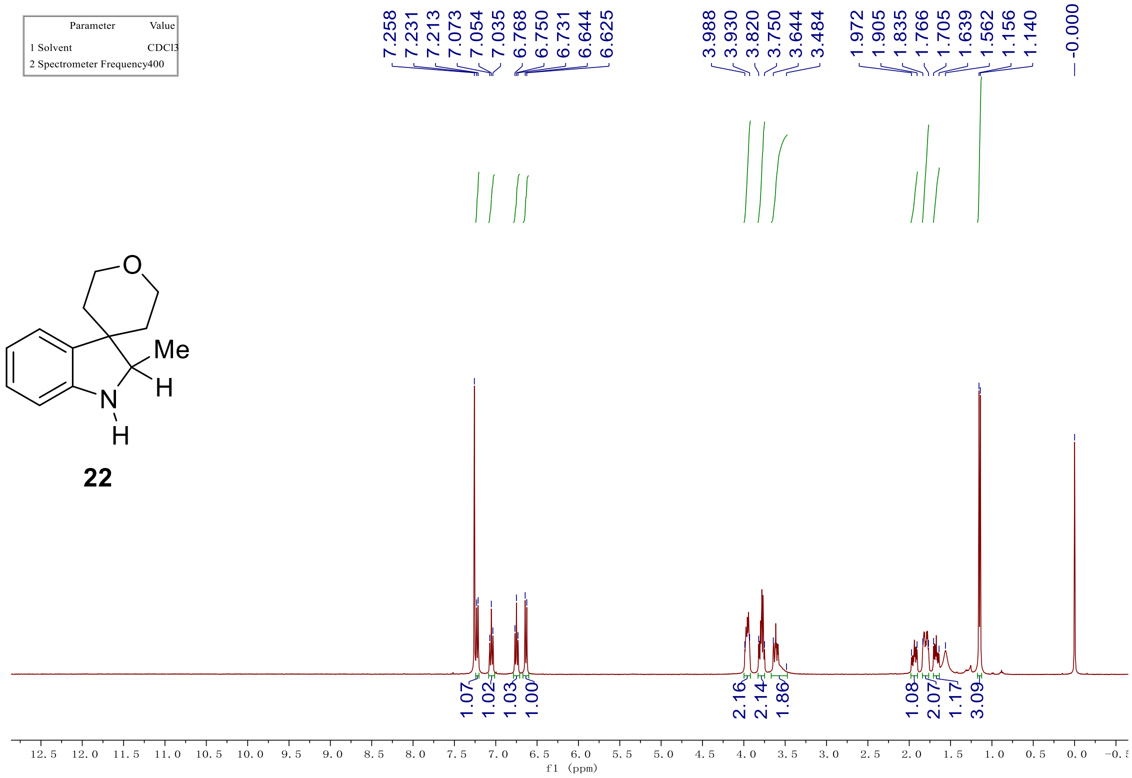

Supplementary Figure 68. ^{1}H NMR spectra of compound **23** (400 MHz, $CDCl_3$)

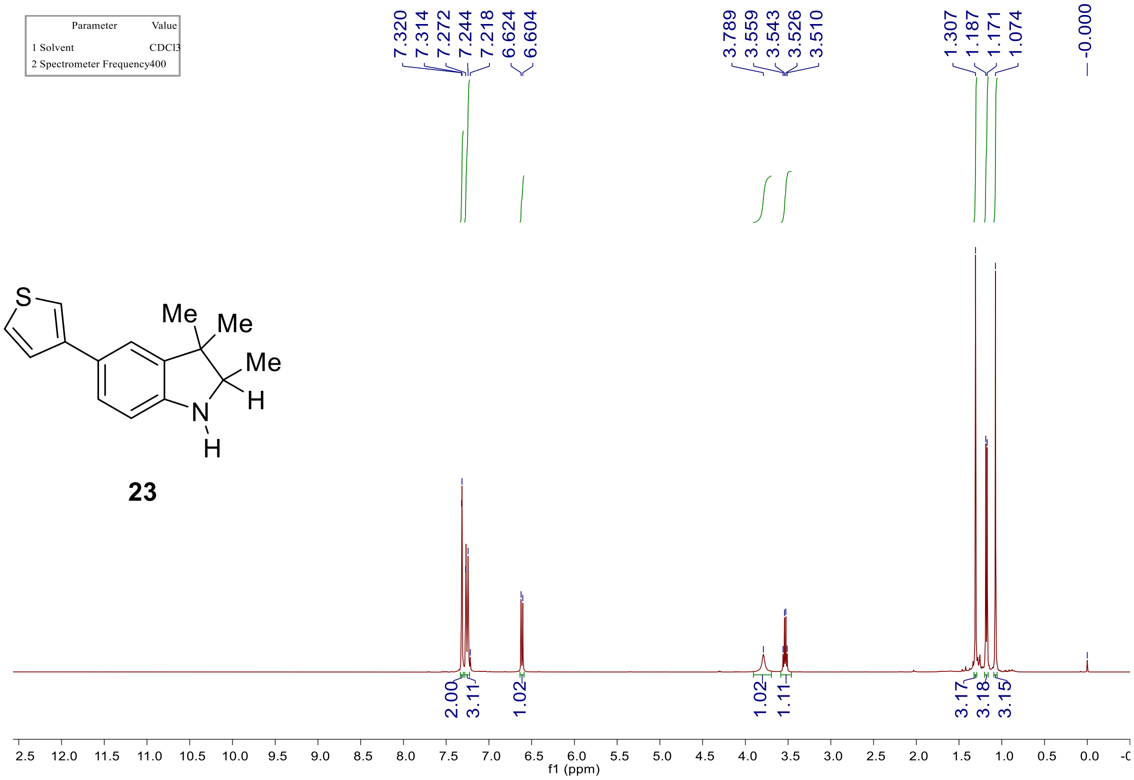

Supplementary Figure 69. ^{13}C NMR spectra of compound **23** (101 MHz, $CDCl_3$)

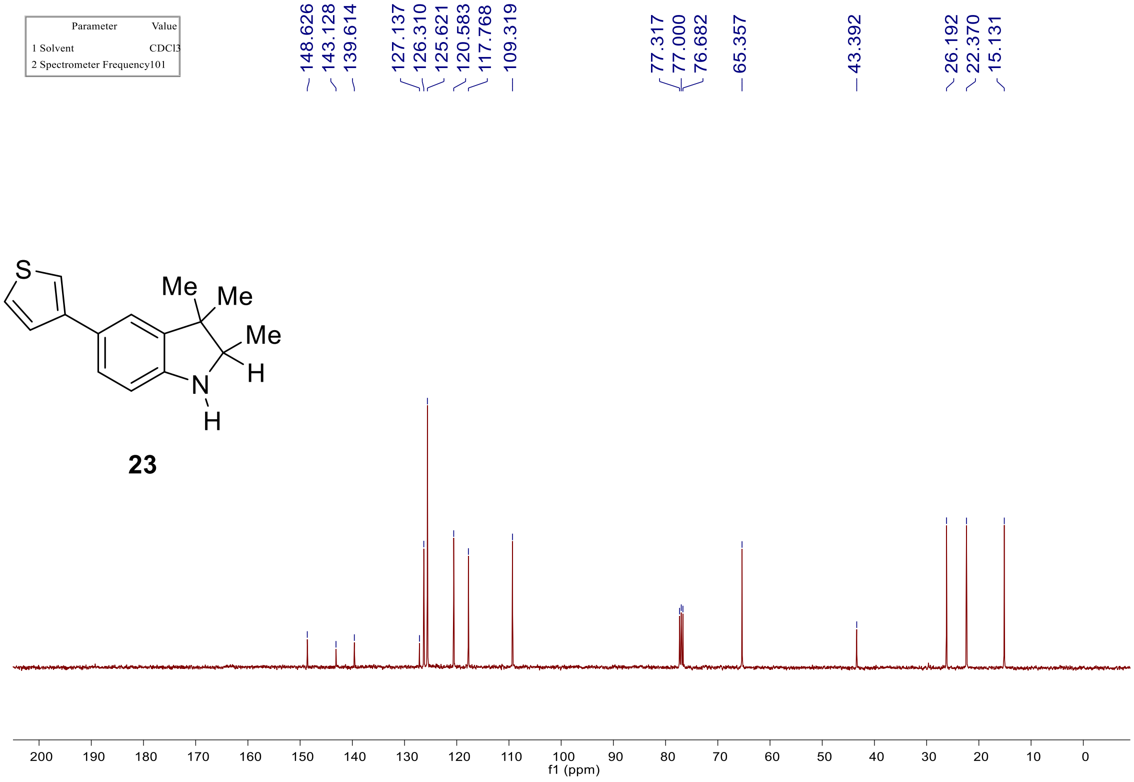

Supplementary Figure 70. ^{1}H NMR spectra of compound **24** (400 MHz, $CDCl_3$)

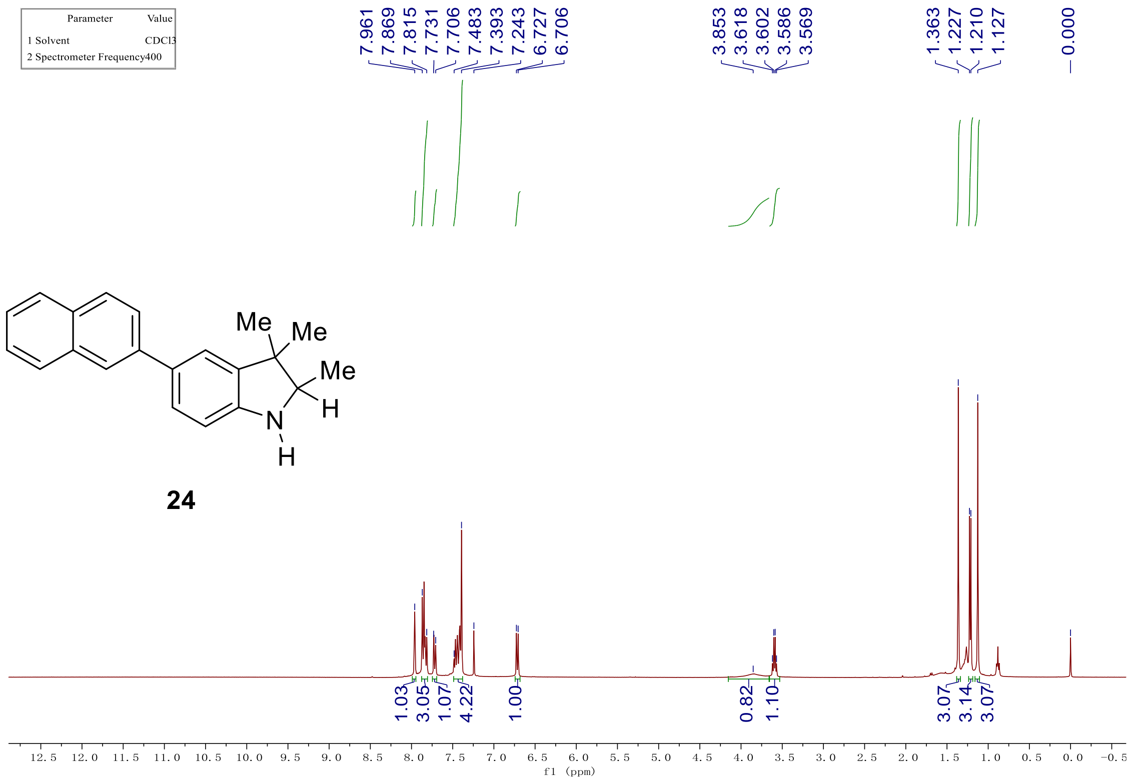

Supplementary Figure 71. ^{13}C NMR spectra of compound **24** (101 MHz, $CDCl_3$)

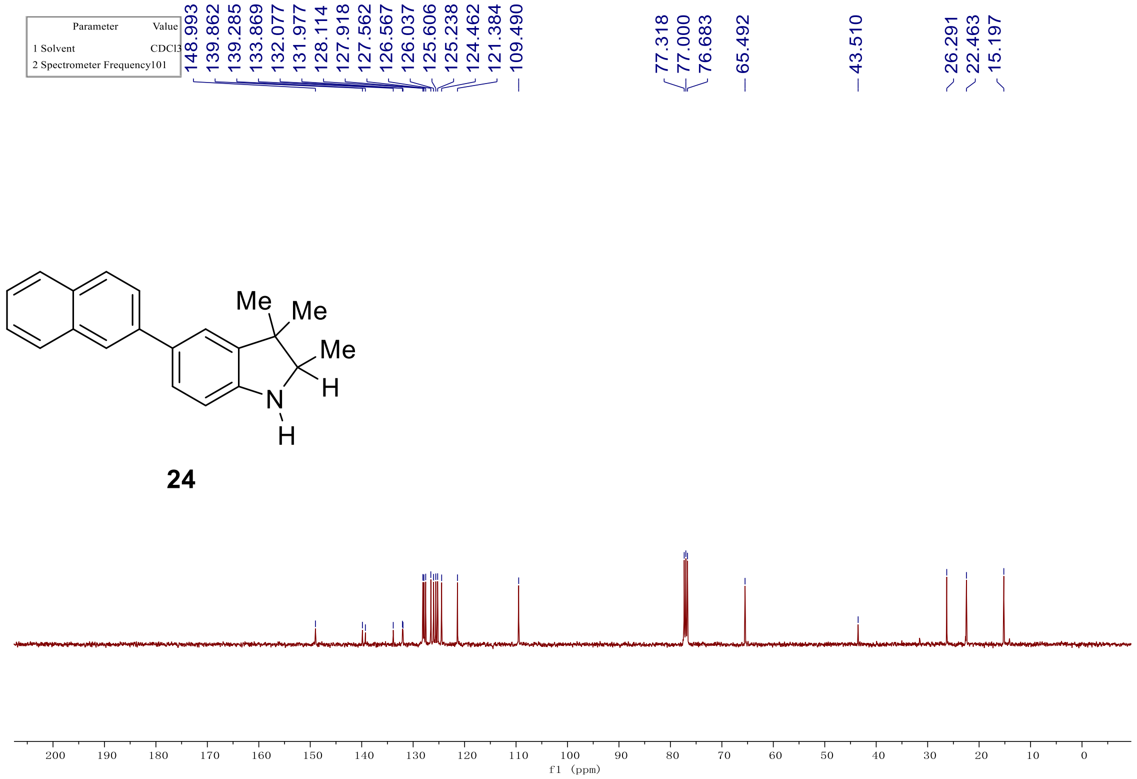

Supplementary Figure 72. ^{1}H NMR spectra of compound **25** (400 MHz, $CDCl_3$)

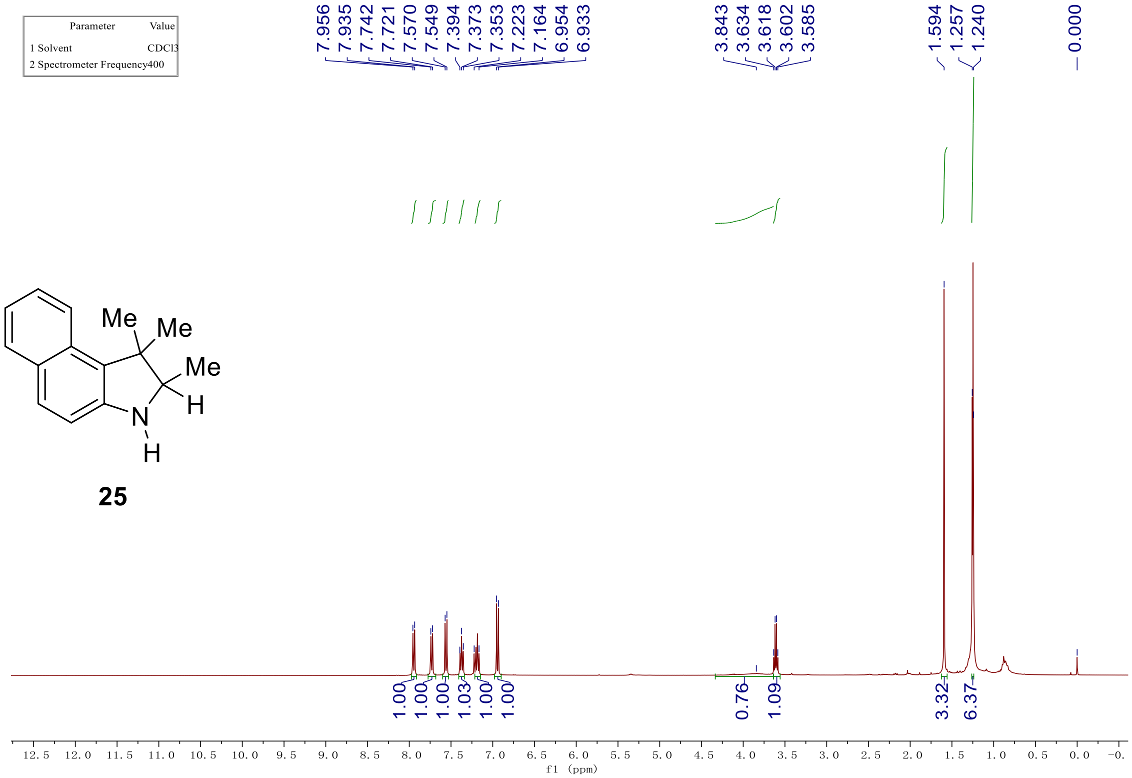

Supplementary Figure 73. ^{1}H NMR spectra of compound **26** (400 MHz, $CDCl_3$)

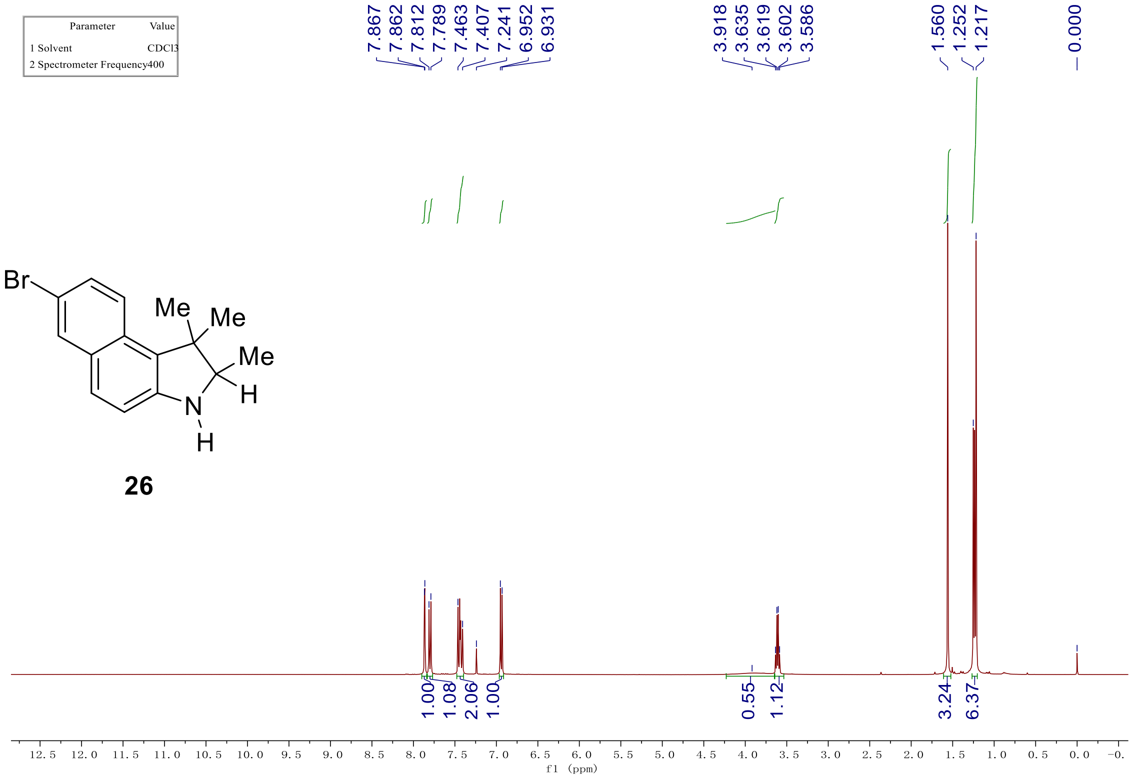

Supplementary Figure 74. ^{13}C NMR spectra of compound **26** (101 MHz, $CDCl_3$)

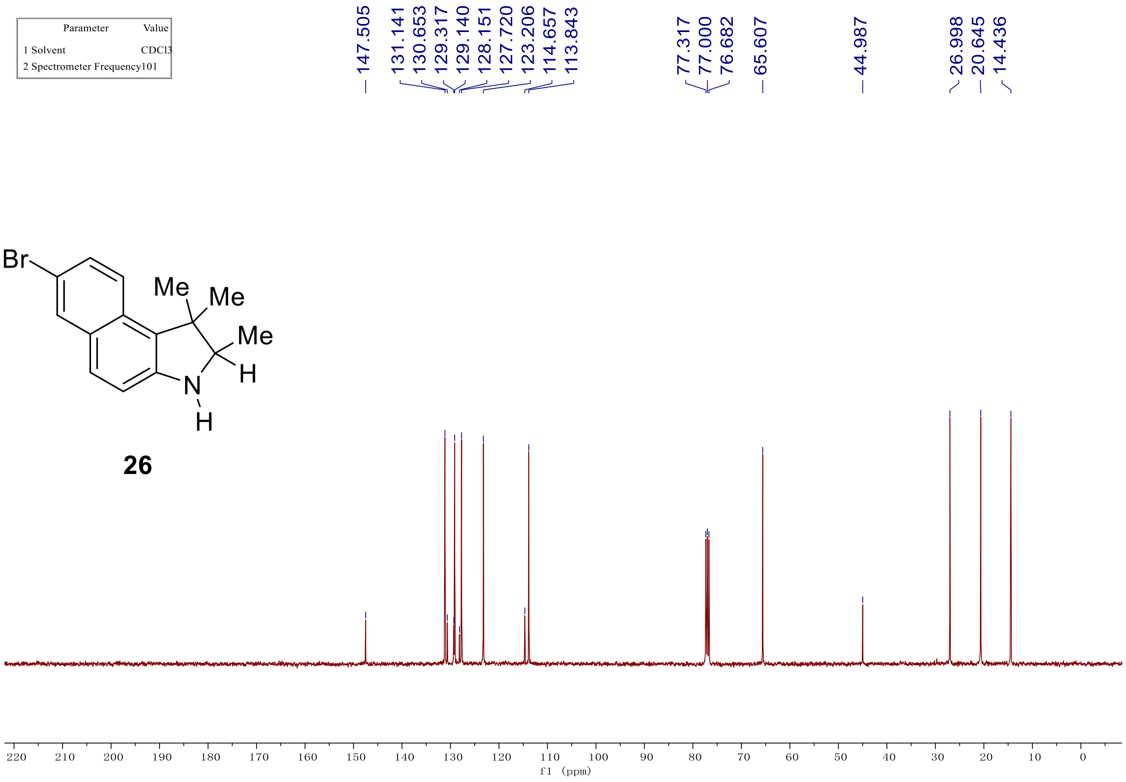

Supplementary Figure 75. ^{1}H NMR spectra of compound **27** (400 MHz, $CDCl_3$)

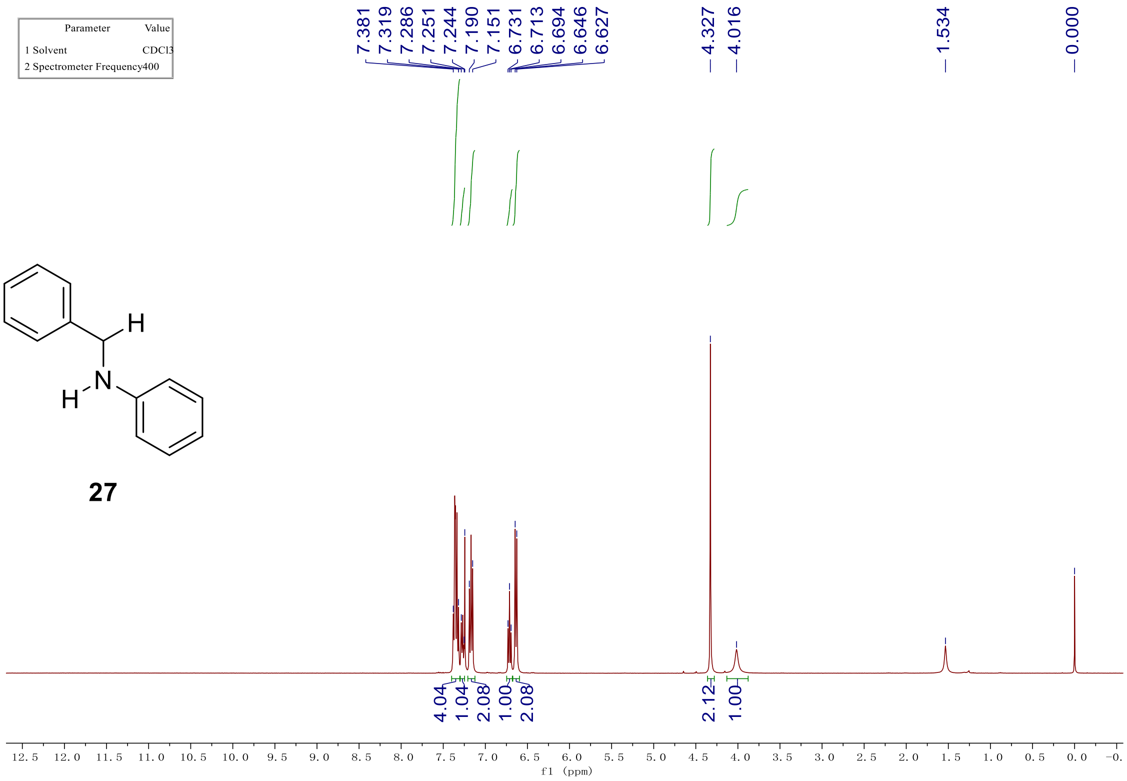

Supplementary Figure 76. ^{1}H NMR spectra of compound **28** (400 MHz, $CDCl_3$)

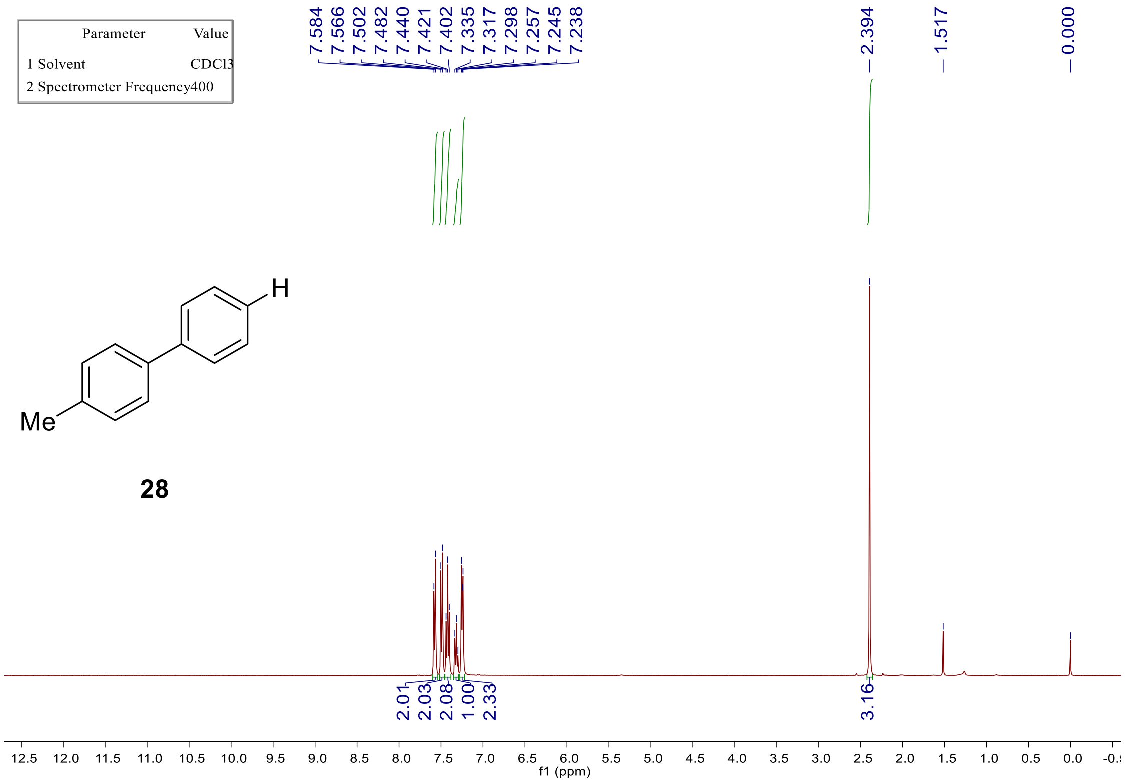

Supplementary Figure 77. ^{1}H NMR spectra of compound **29** (400 MHz, $CDCl_3$)

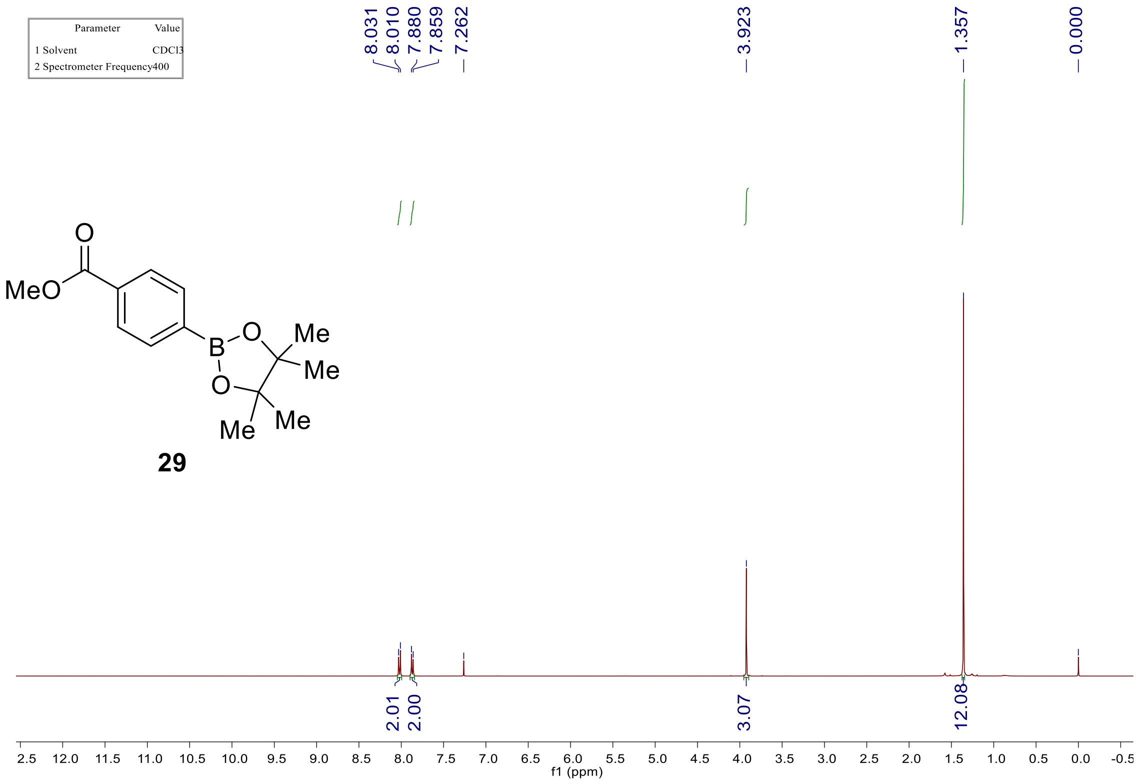

Supplementary Figure 78. ^{1}H NMR spectra of compound **30** (400 MHz, $CDCl_3$)

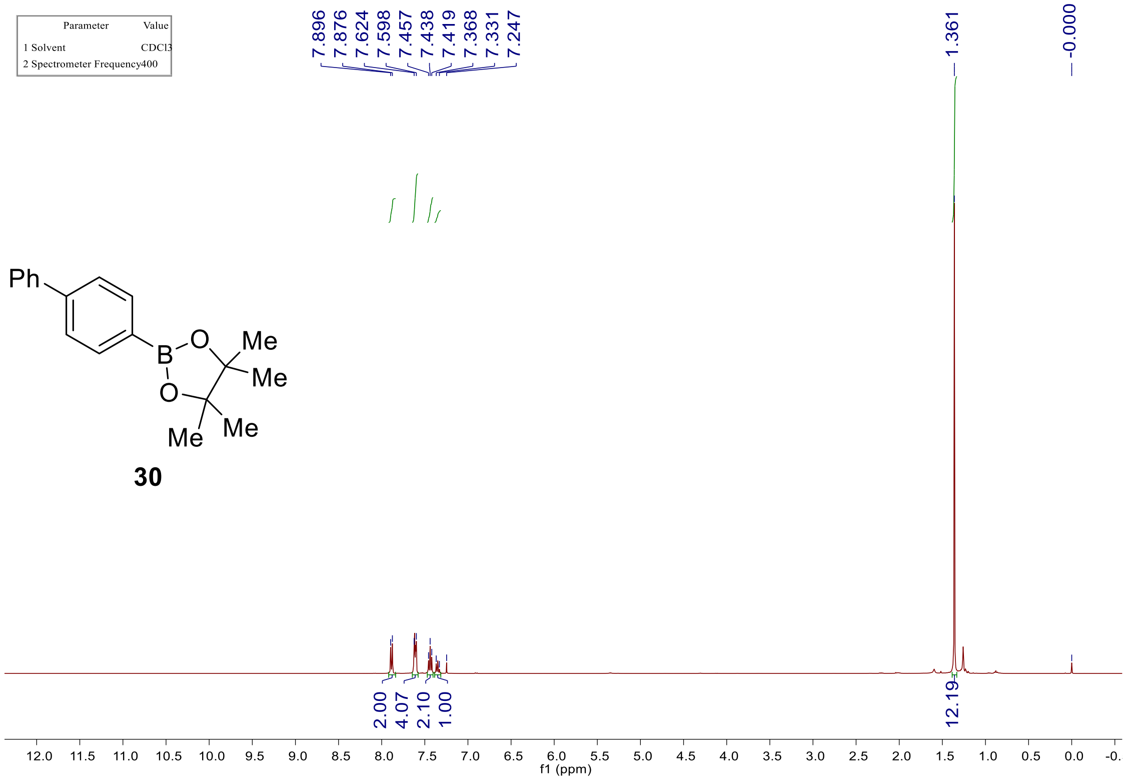

Supplementary Figure 79. ^{1}H NMR spectra of compound **31** (400 MHz, $CDCl_3$)

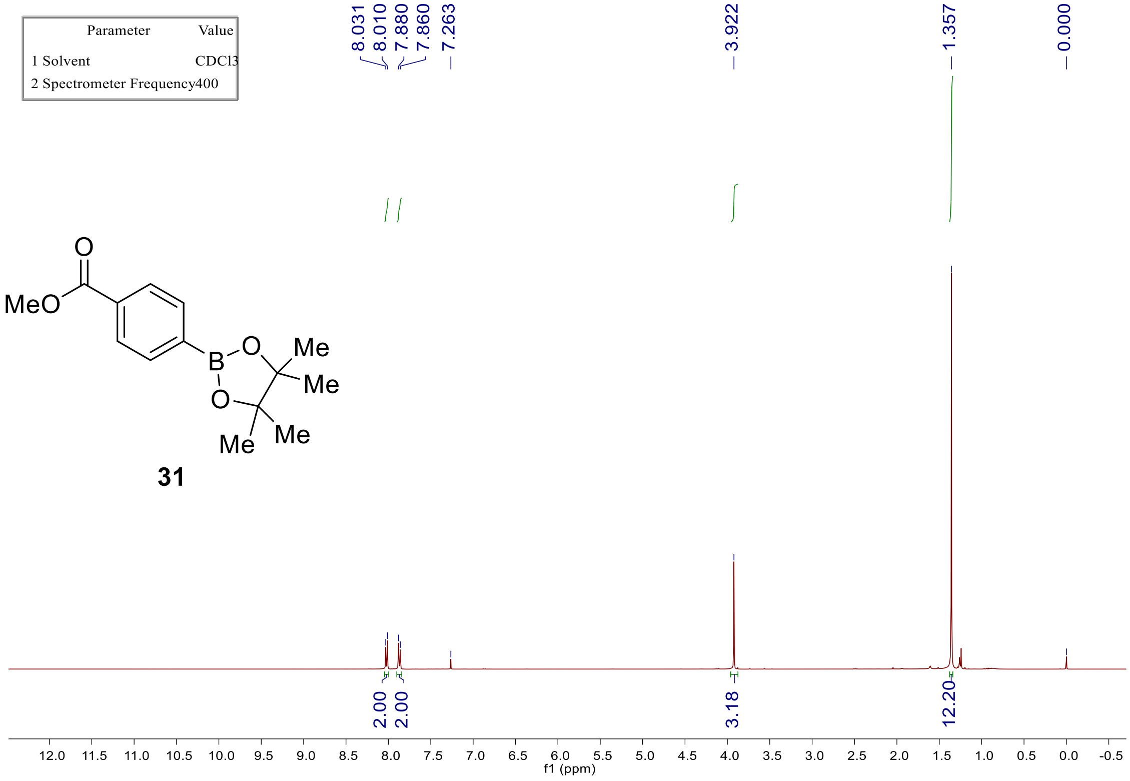

Supplementary Figure 80. ^{1}H NMR spectra of compound **32** (400 MHz, $CDCl_3$)

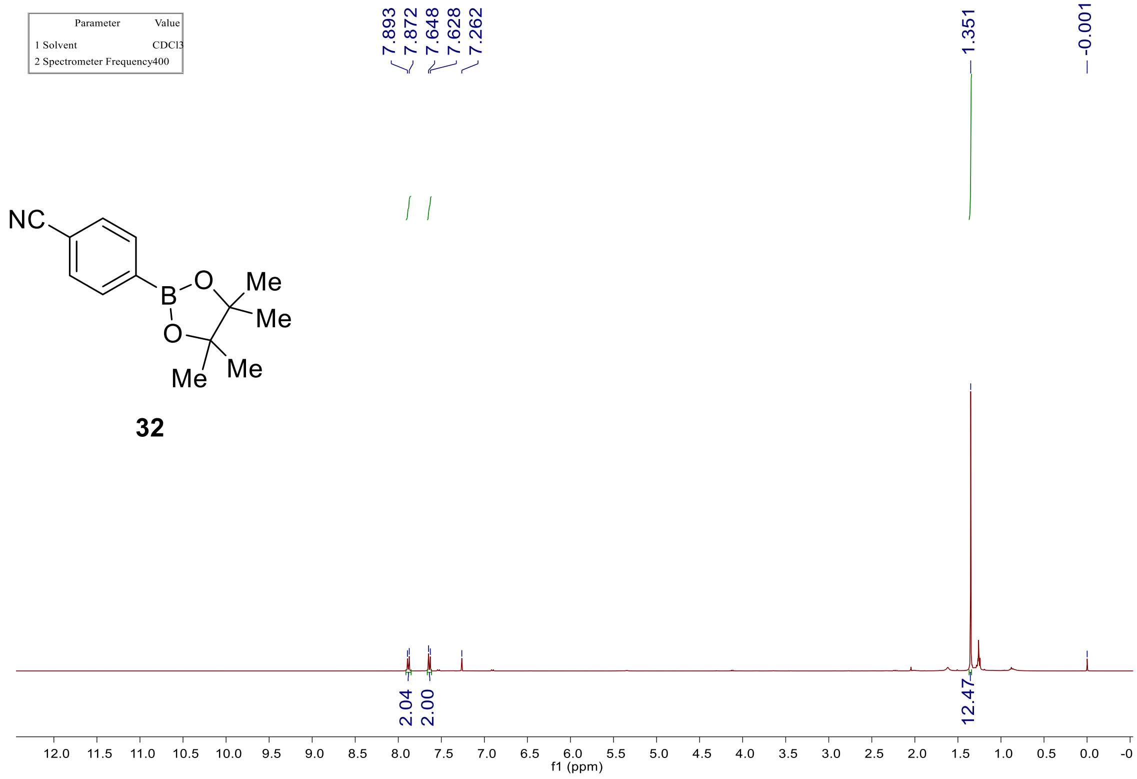

Supplementary Figure 81. ^{1}H NMR spectra of compound **33** (400 MHz, $CDCl_3$)

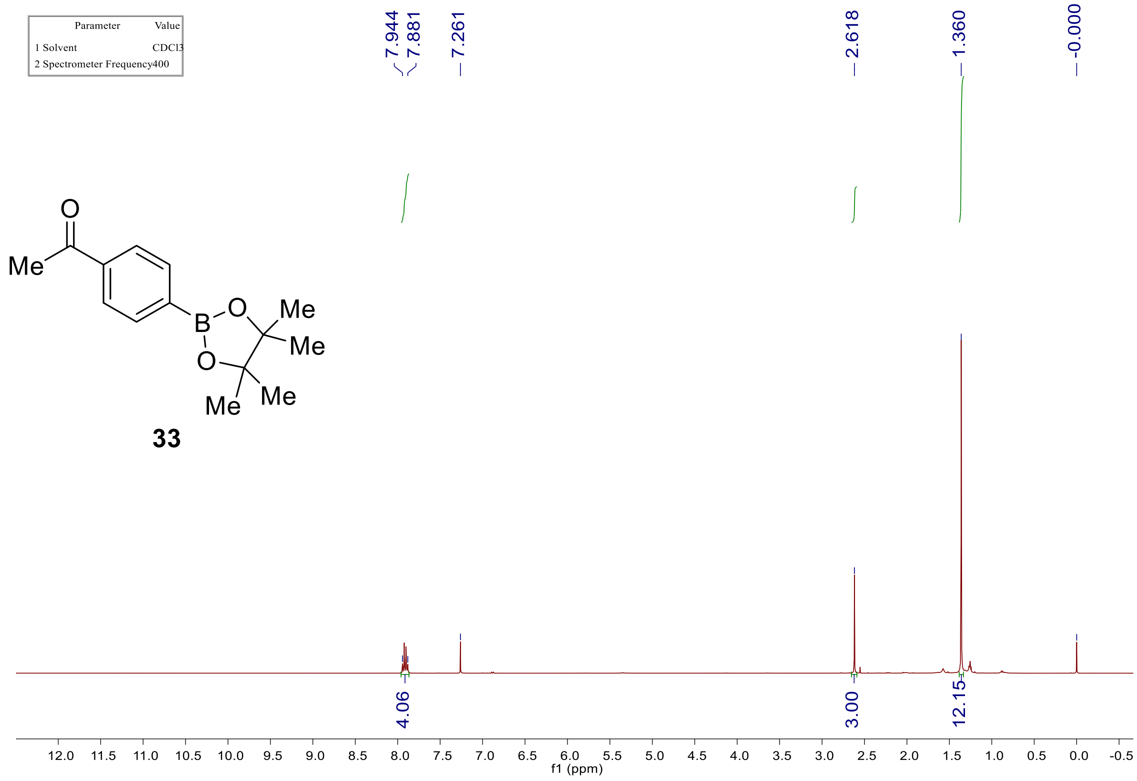

Supplementary Figure 82. ^{1}H NMR spectra of compound **34** (400 MHz, $CDCl_3$)

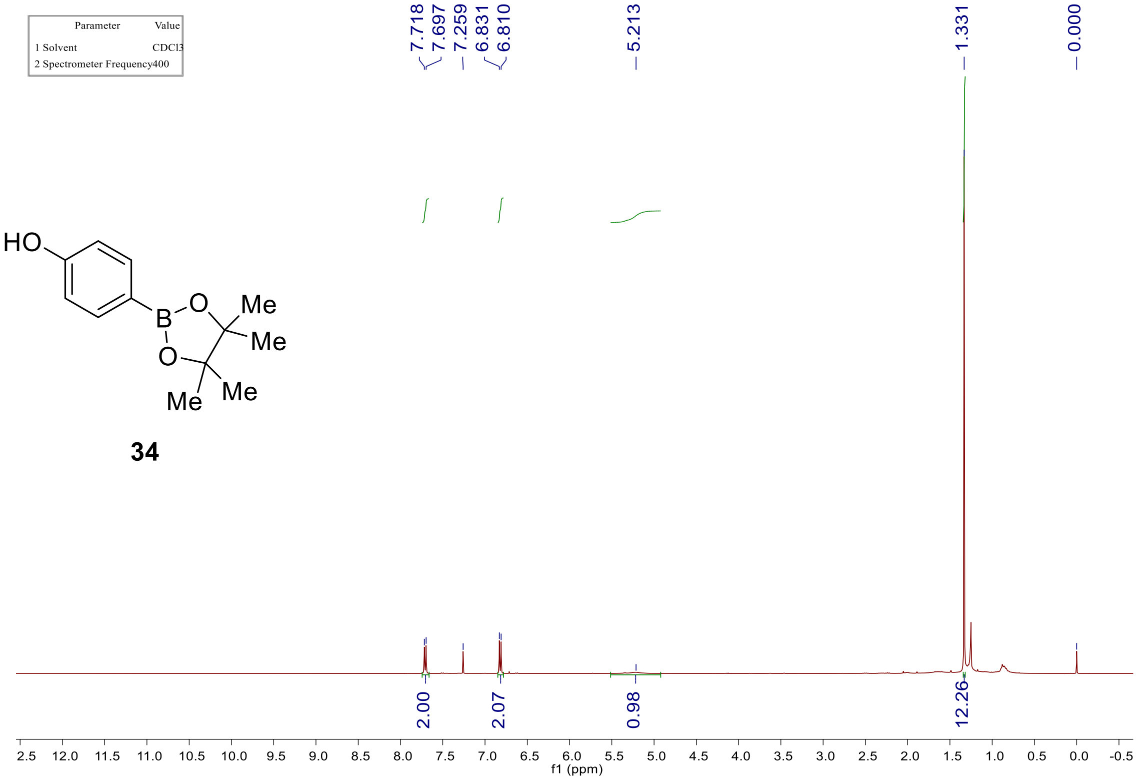

Supplementary Figure 83. ^{1}H NMR spectra of compound **35** (400 MHz, $CDCl_3$)

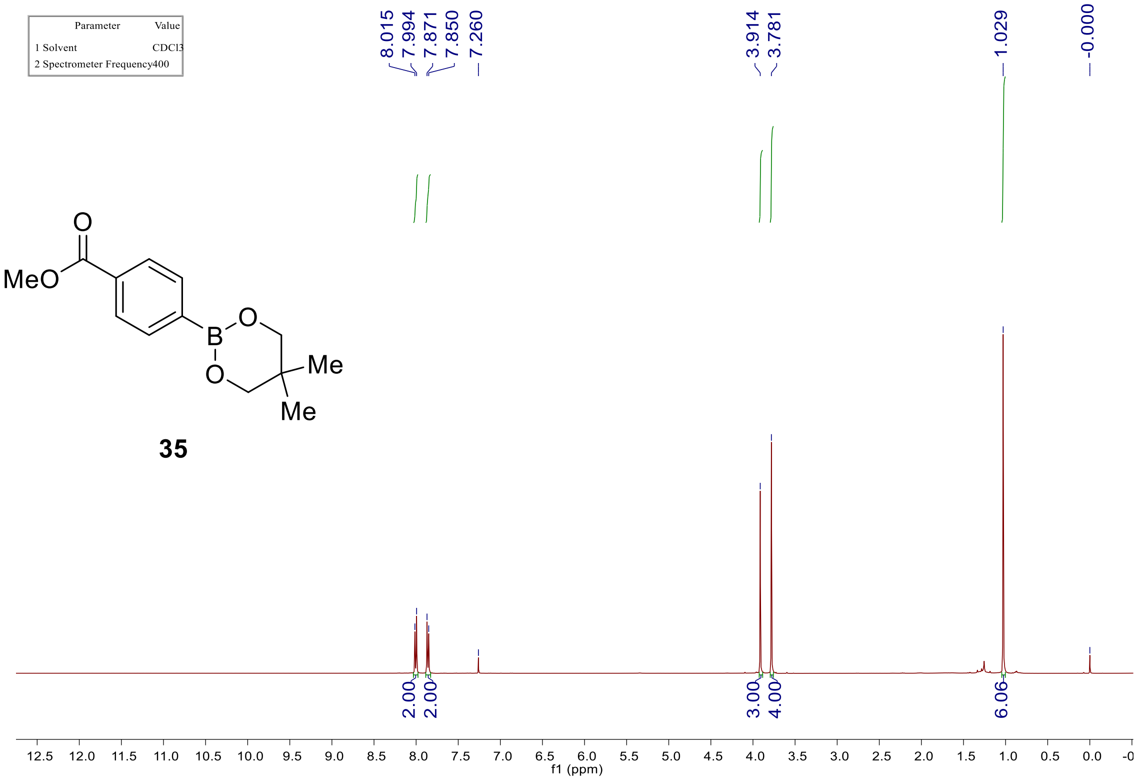

Supplementary Figure 84. ^{1}H NMR spectra of compound **36** (400 MHz, $CDCl_3$)

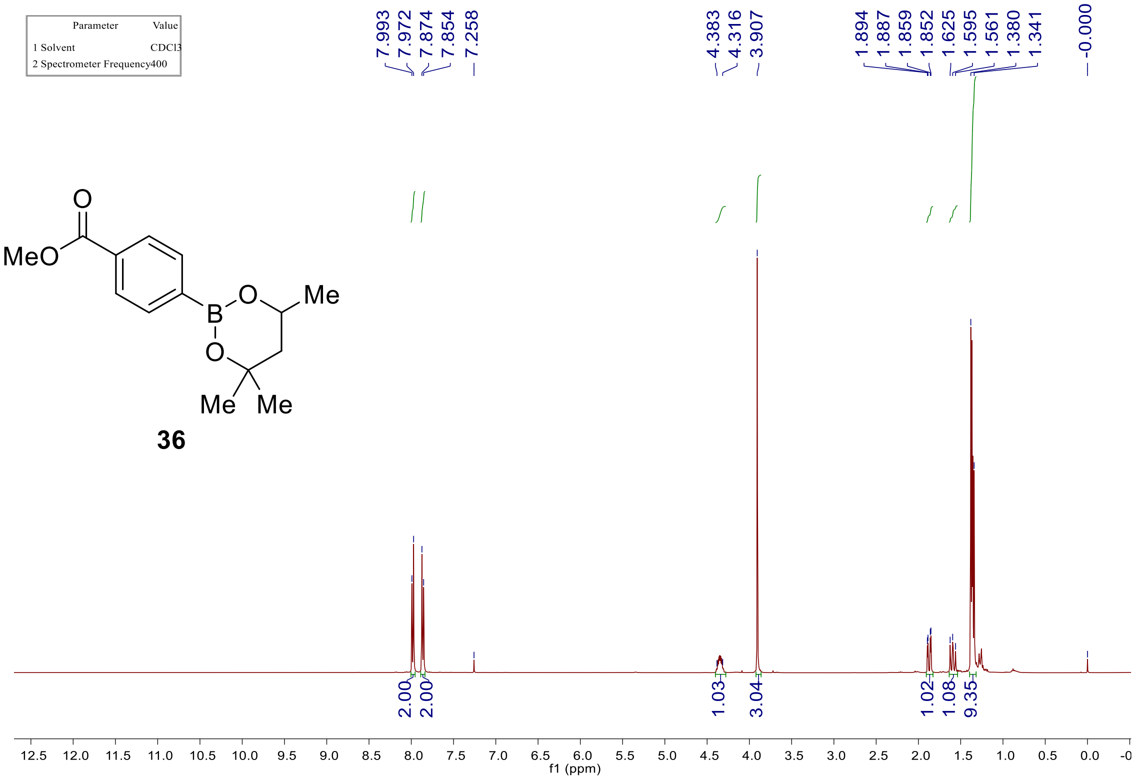

Supplementary Figure 85. ^{1}H NMR spectra of compound **37** (400 MHz, $CDCl_3$)

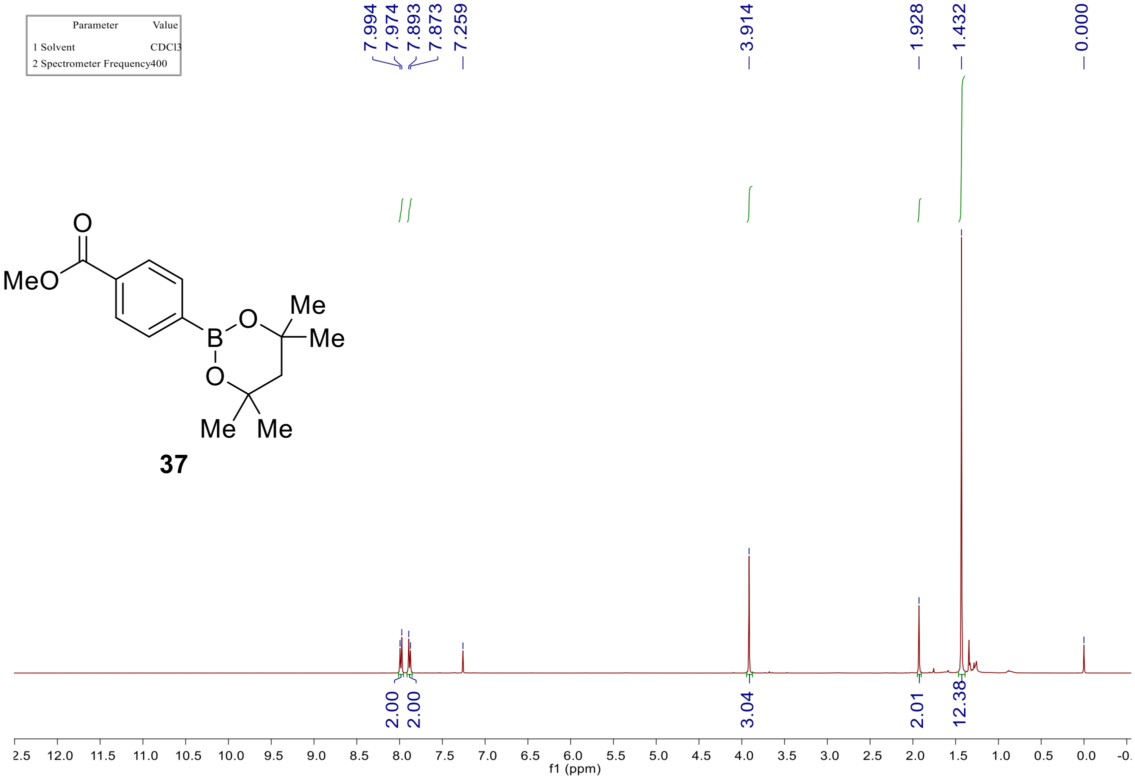

Supplementary Figure 86. ^{13}C NMR spectra of compound **37** (101 MHz, $CDCl_3$)

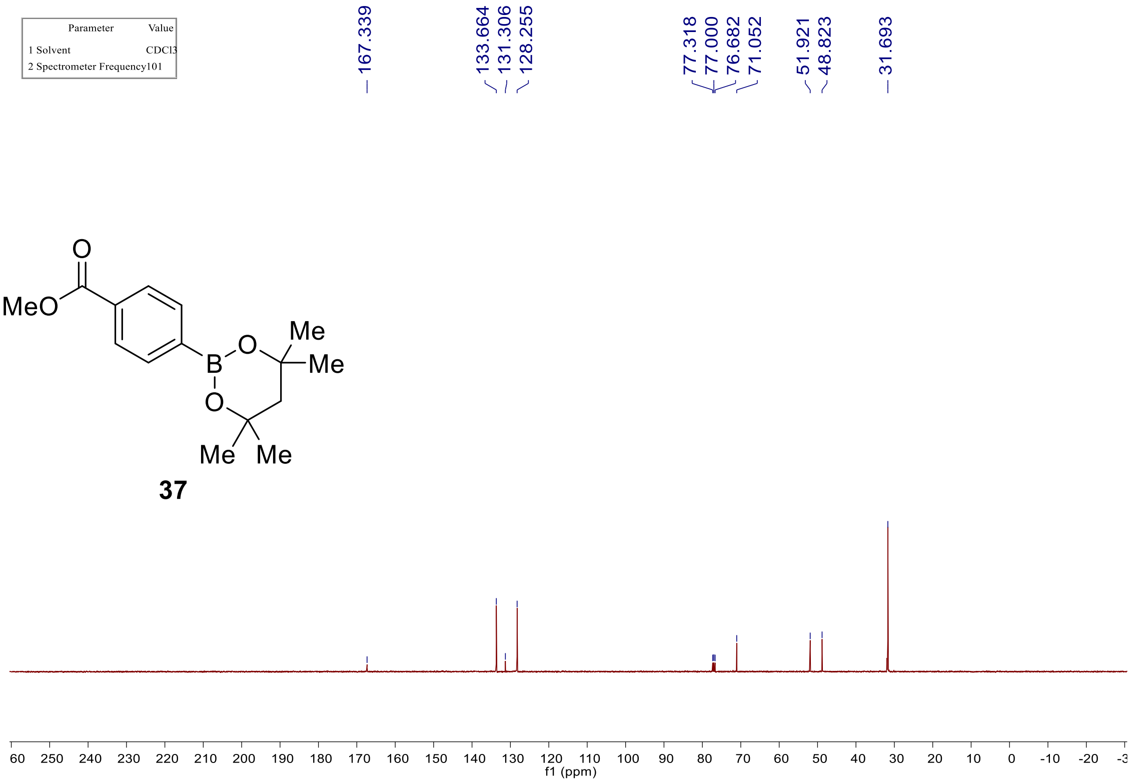

Supplementary Figure 87. ^{11}B NMR spectra of compound **37** (128 MHz, $CDCl_3$)

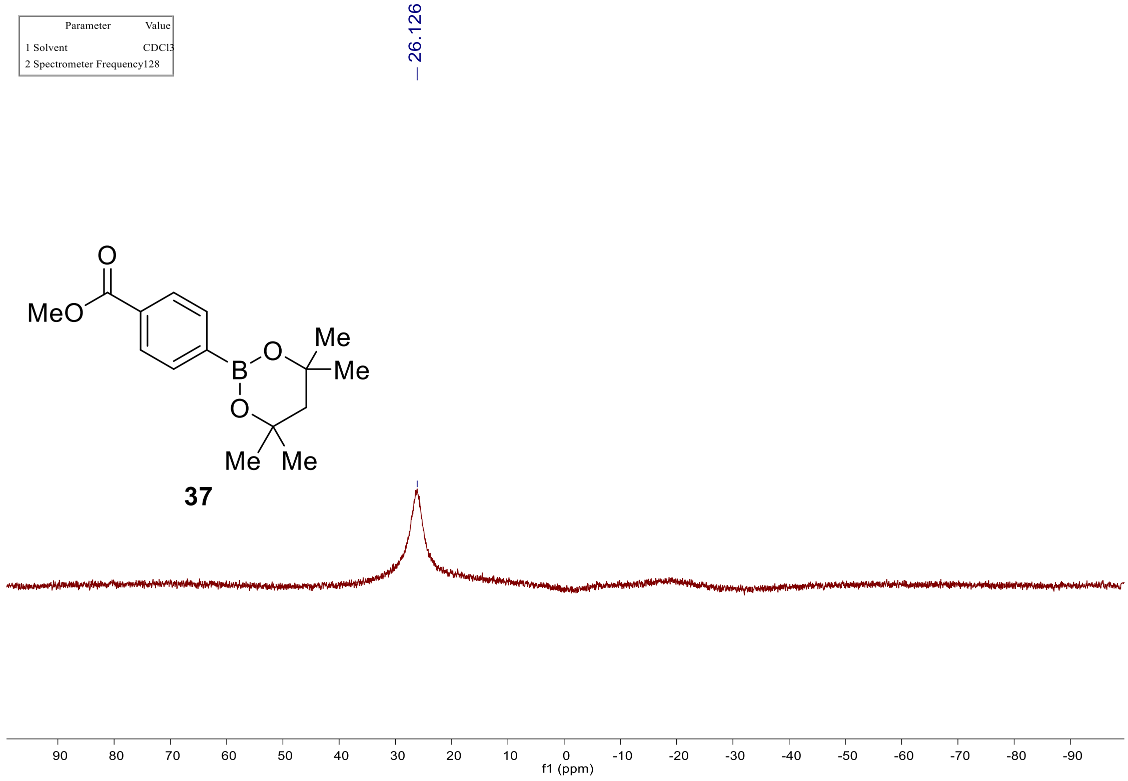

Supplementary Figure 88. ^{1}H NMR spectra of compound **38** (400 MHz, $CDCl_3$)

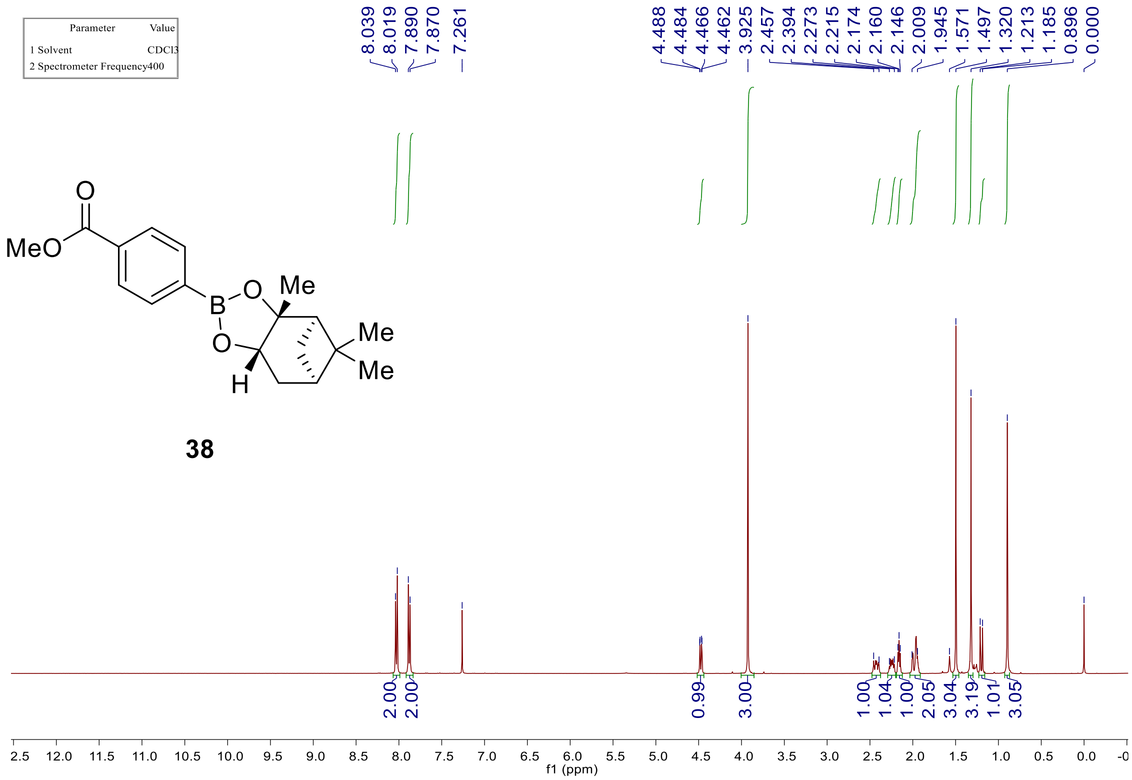

Supplementary Figure 89. ^{13}C NMR spectra of compound **38** (101 MHz, $CDCl_3$)

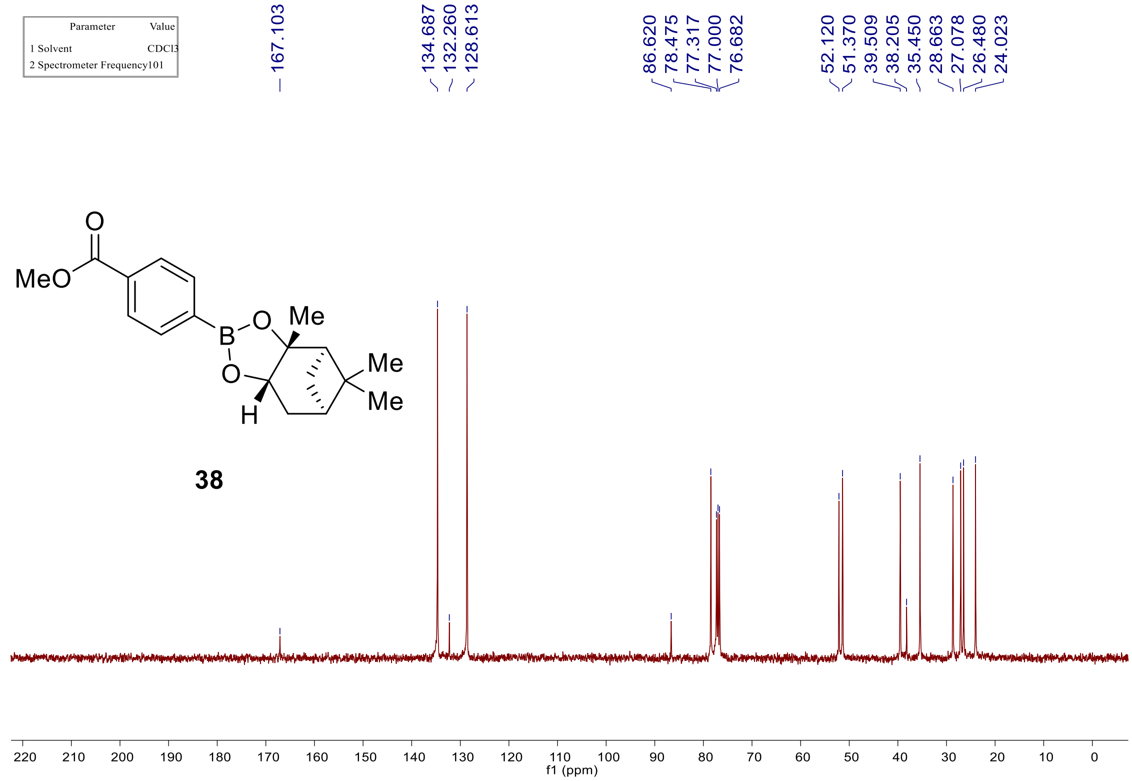

Supplementary Figure 90. ^{11}B NMR spectra of compound **38** (128 MHz, $CDCl_3$)

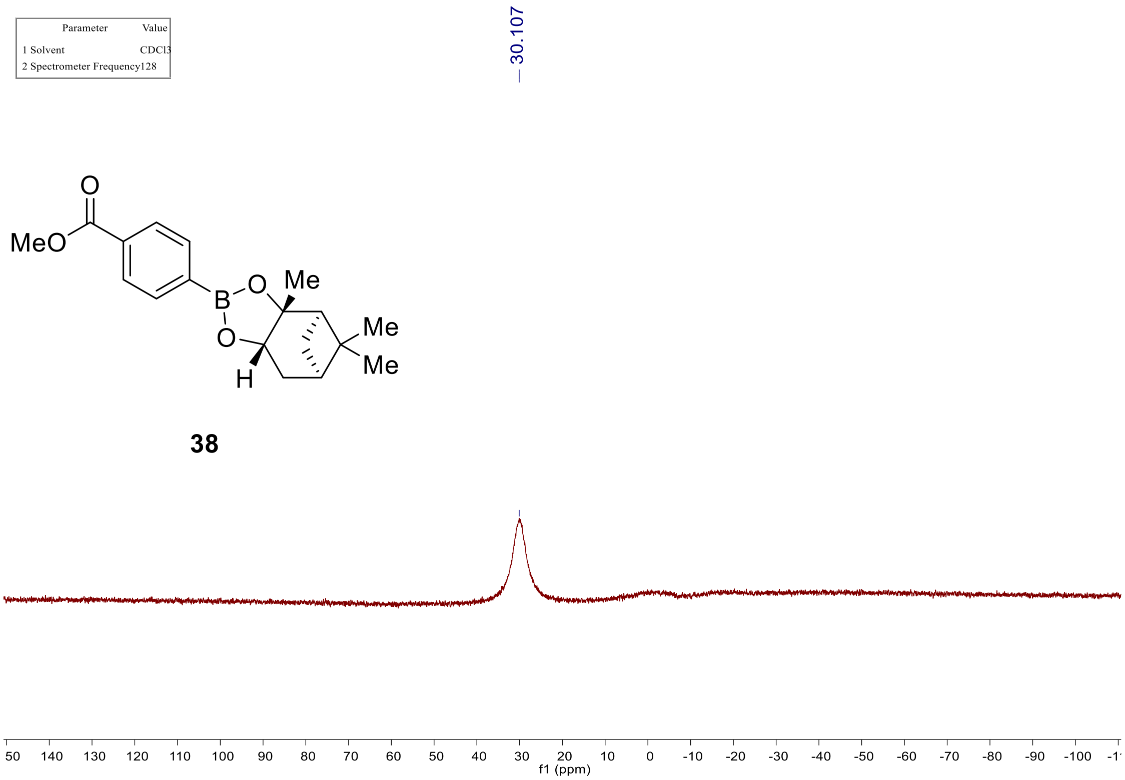

Supplementary Figure 91. ^{1}H NMR spectra of compound **39** (400 MHz, $CDCl_3$)

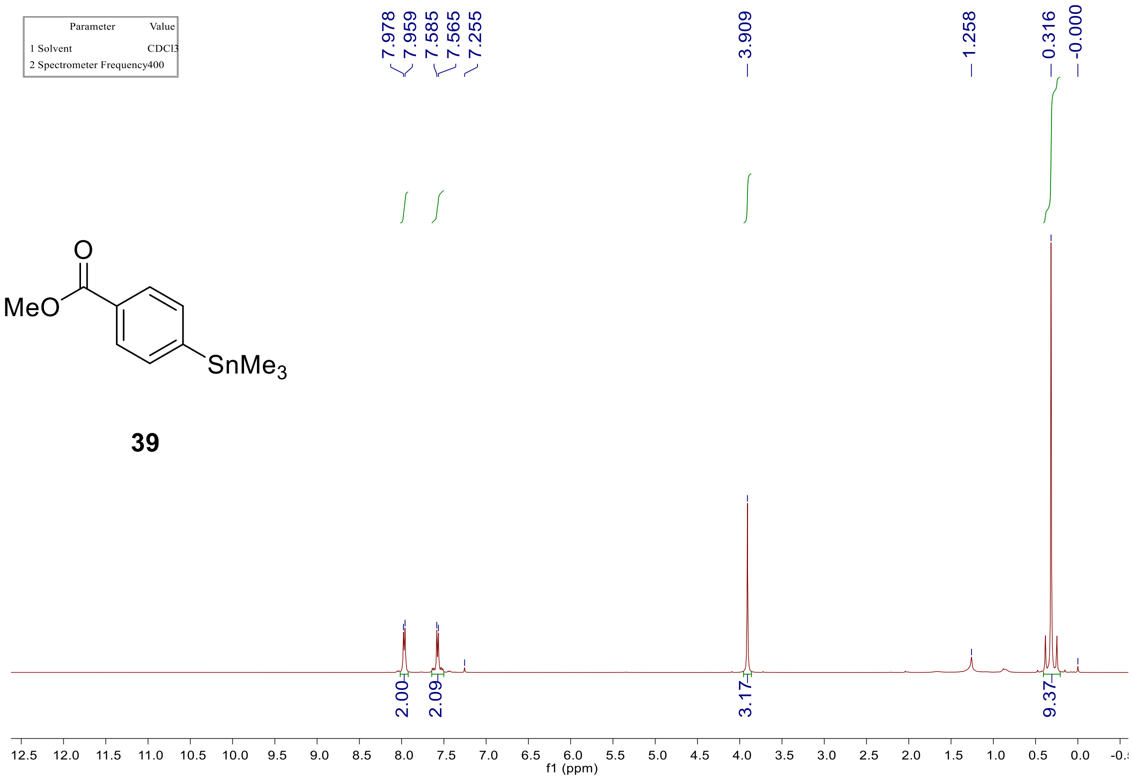

Supplementary Figure 92. ^{1}H NMR spectra of compound **40** (400 MHz, $CDCl_3$)

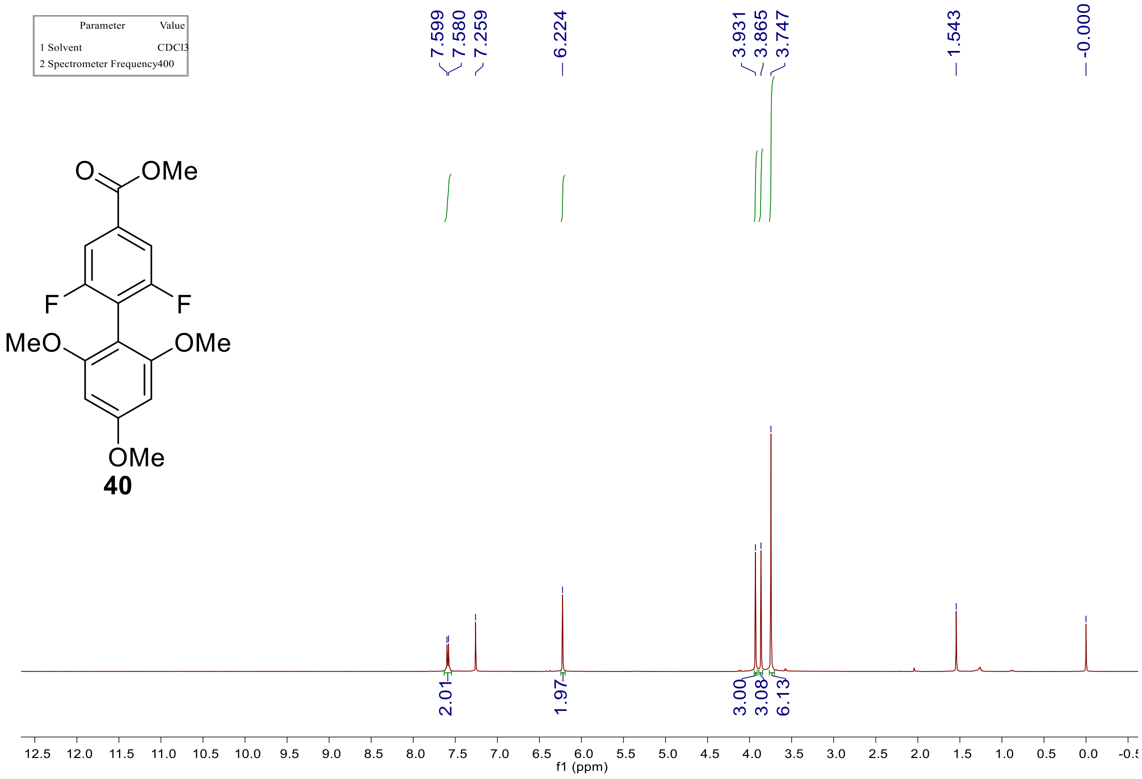

Supplementary Figure 93. ^{1}H NMR spectra of compound **41** (400 MHz, $CDCl_3$)

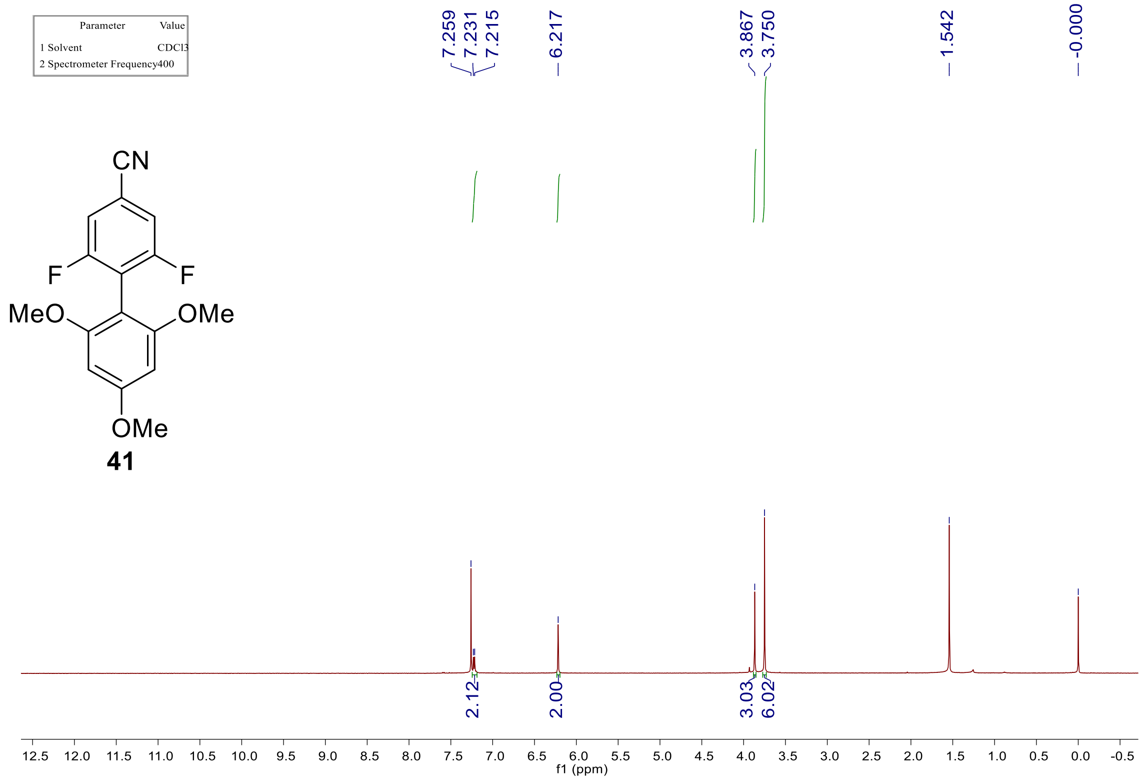

Supplementary Figure 94. ^{1}H NMR spectra of compound **42** (400 MHz, $CDCl_3$)

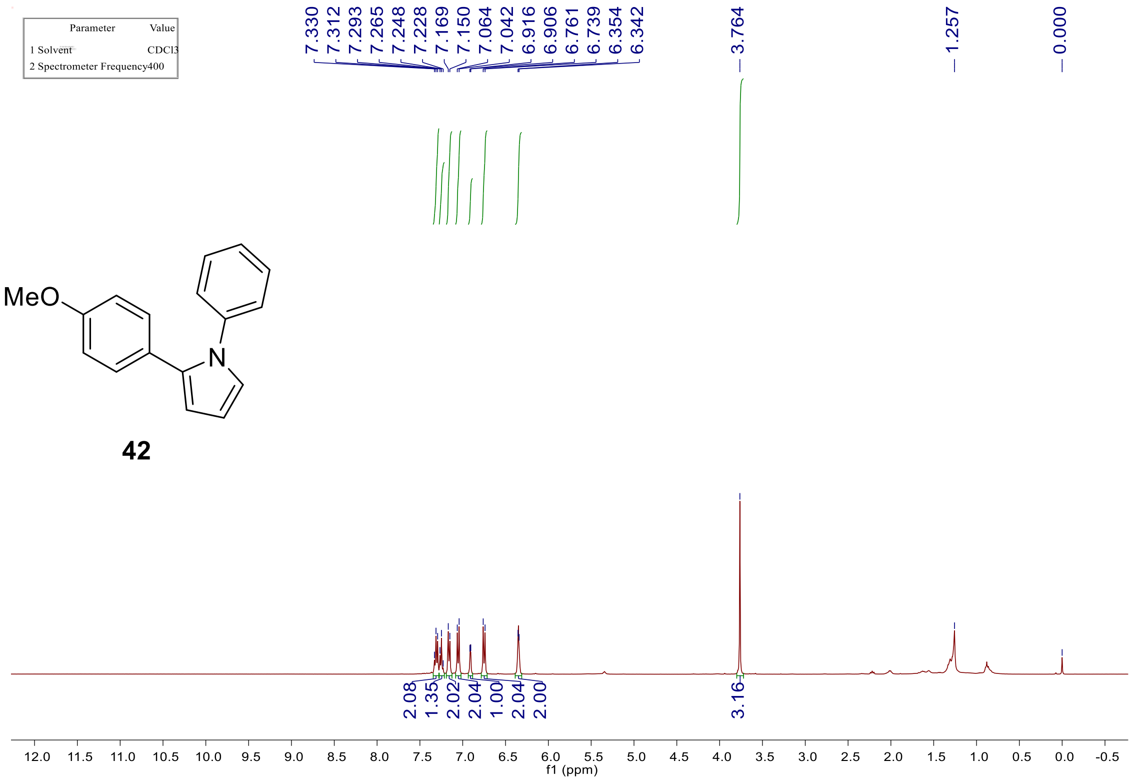

Supplementary Figure 95. ^{1}H NMR spectra of compound **43** (400 MHz, $CDCl_3$)

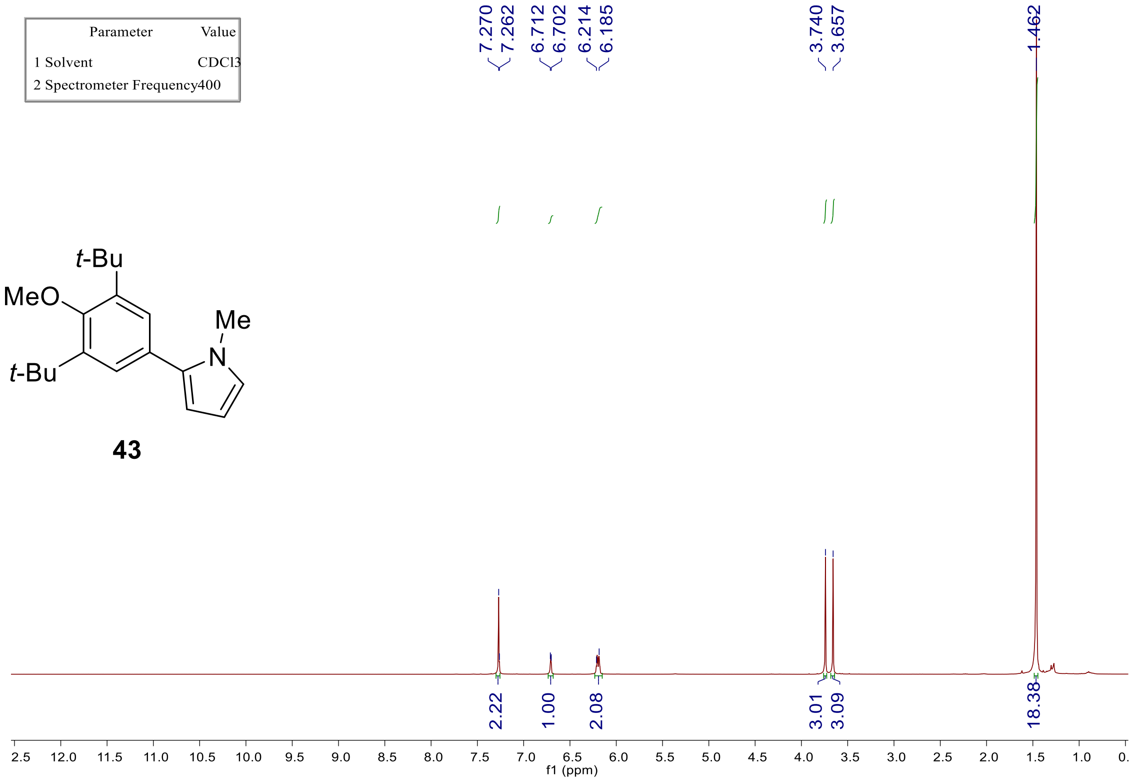

Supplementary Figure 96. ^{1}H NMR spectra of compound **44** (400 MHz, $CDCl_3$)

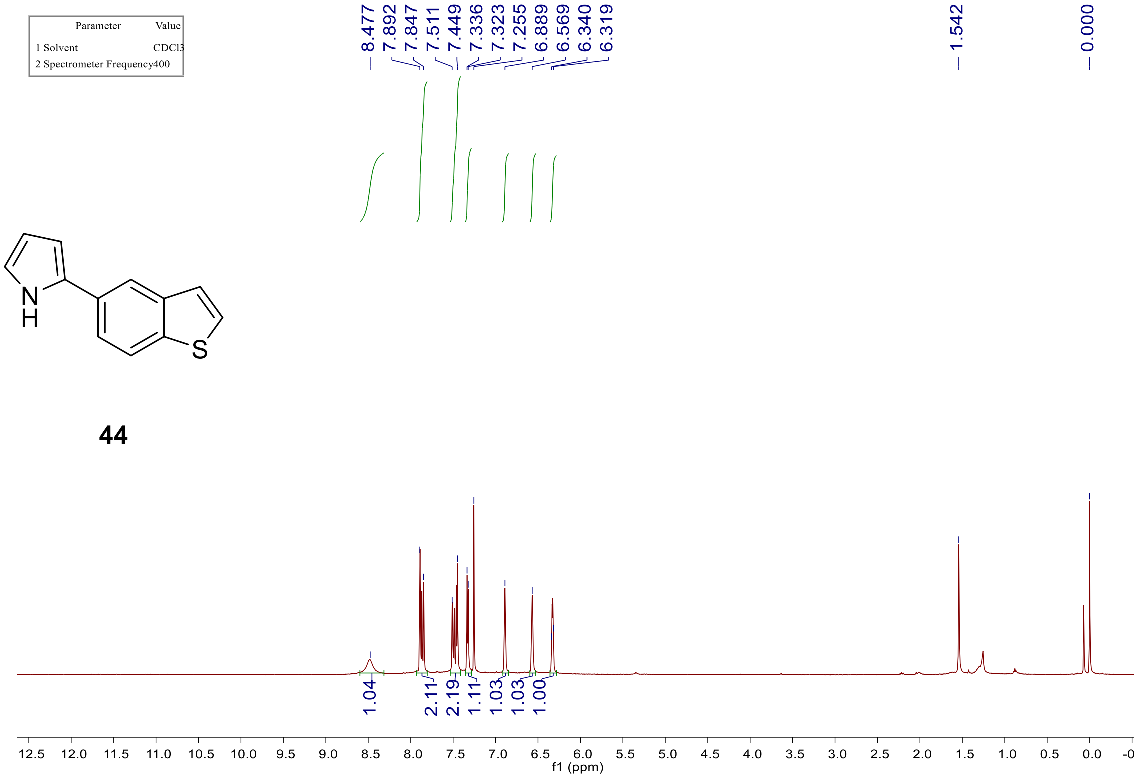

Supplementary Figure 97. ^{13}C NMR spectra of compound **44** (101 MHz, $CDCl_3$)

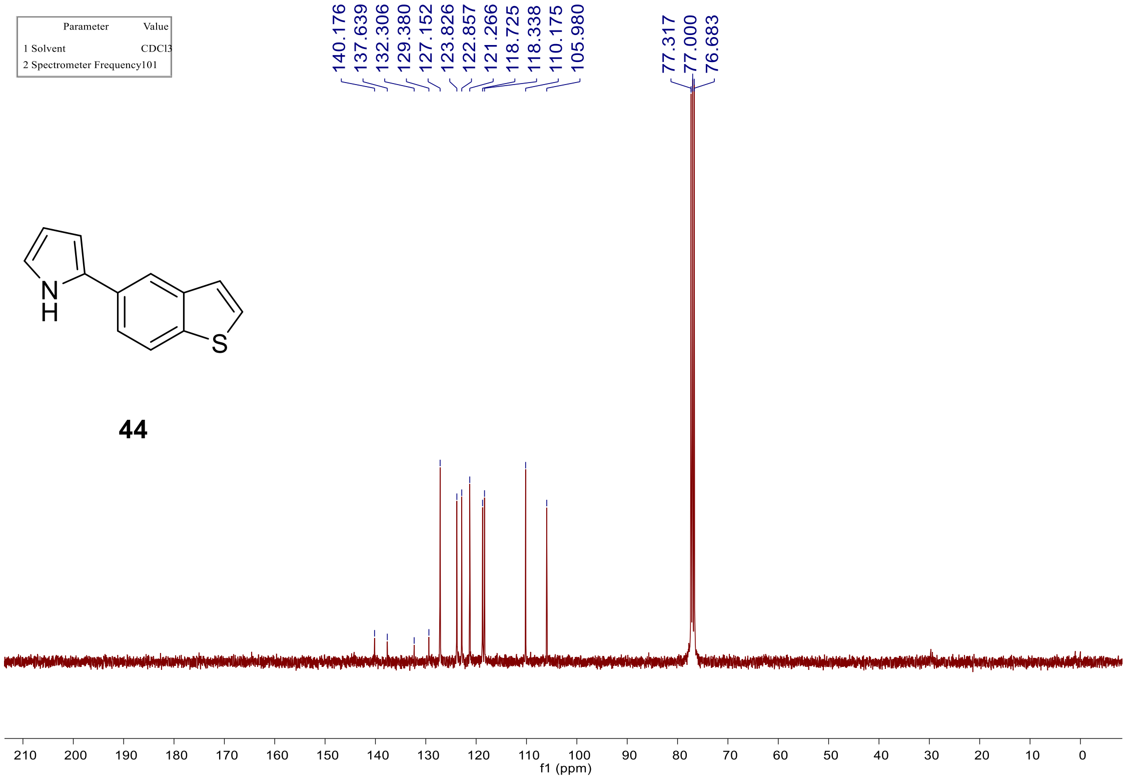

Supplementary Figure 98. ^{1}H NMR spectra of compound **45** (400 MHz, $CDCl_3$)

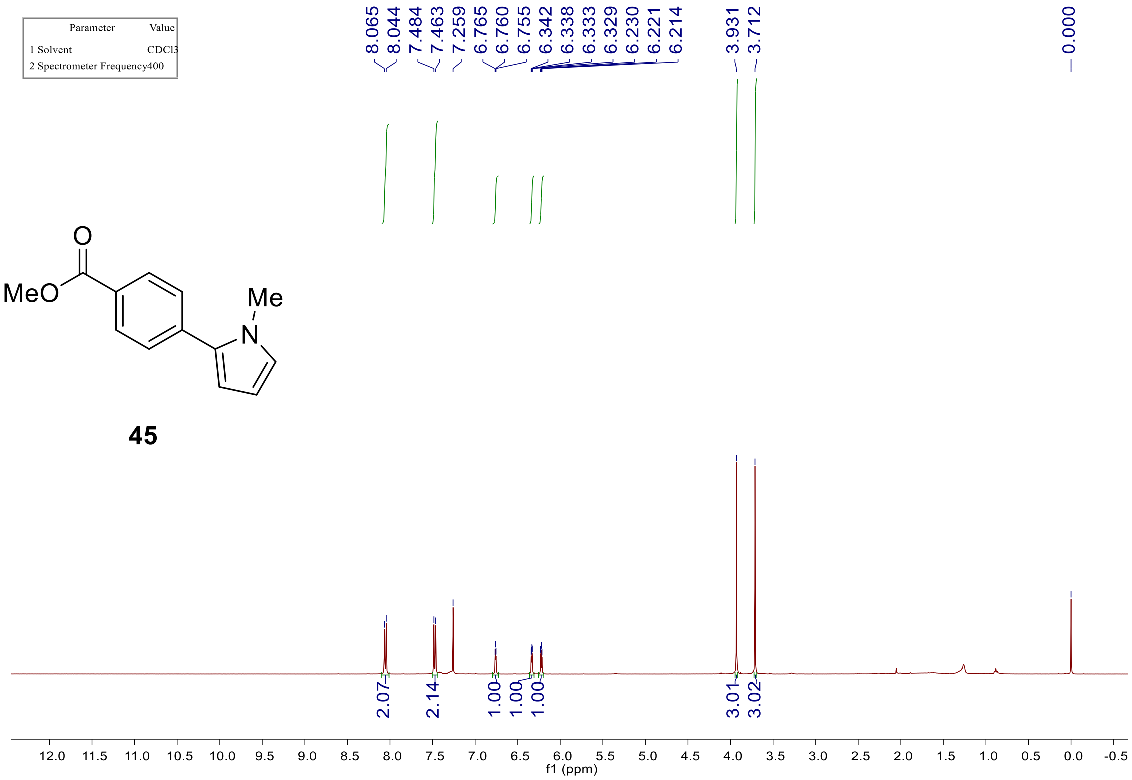

Supplementary Figure 99. ^{1}H NMR spectra of compound **46** (400 MHz, $CDCl_3$)

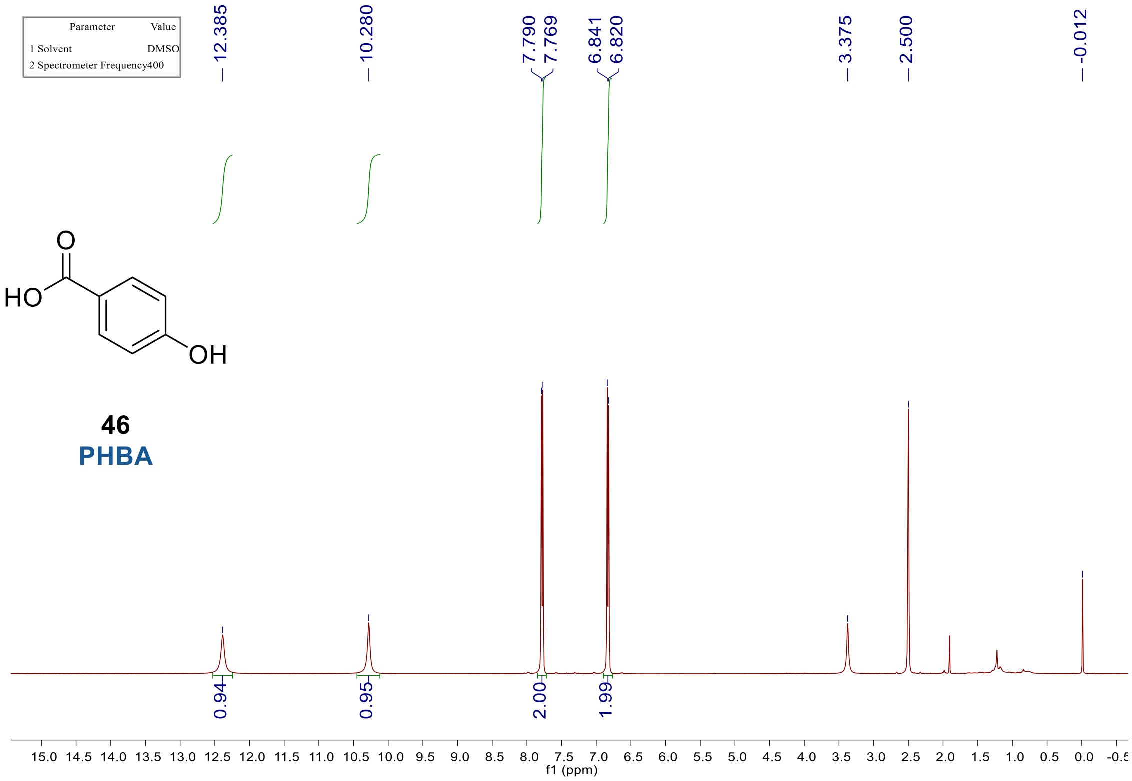

Supplementary Figure 100. ^{1}H NMR spectra of compound **47** (400 MHz, $CDCl_3$)

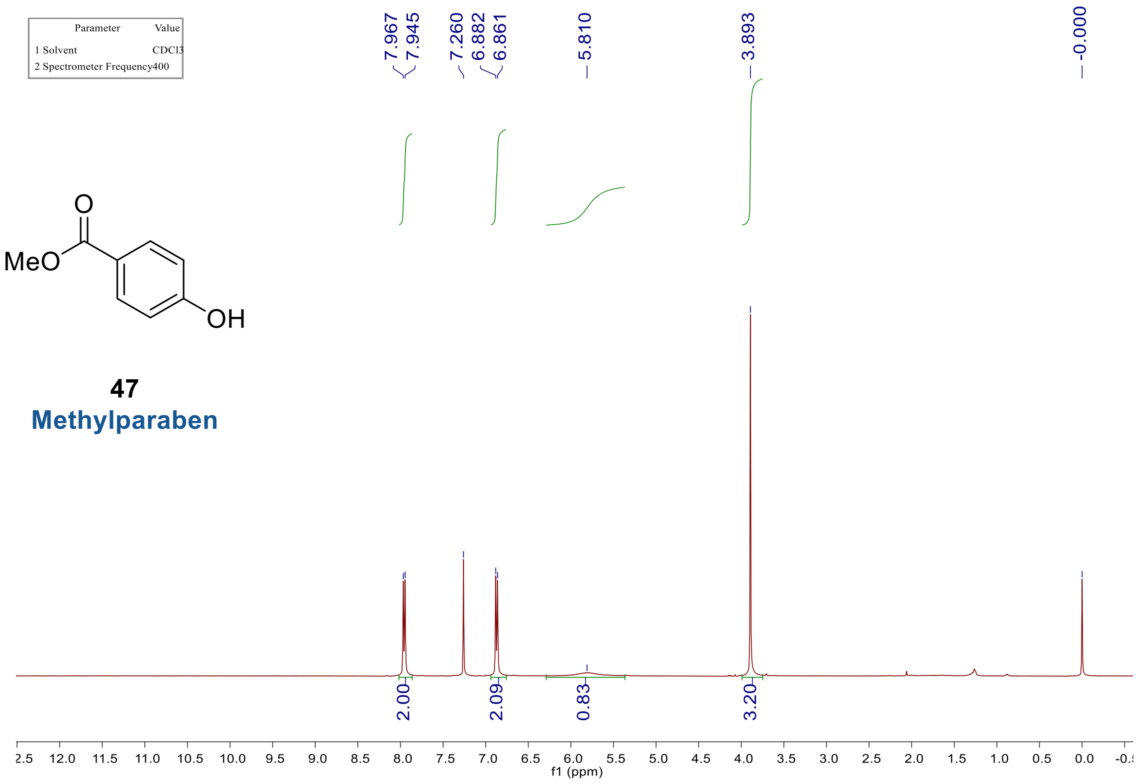

Supplementary Figure 101. ^{1}H NMR spectra of compound **48** (400 MHz, $CDCl_3$)

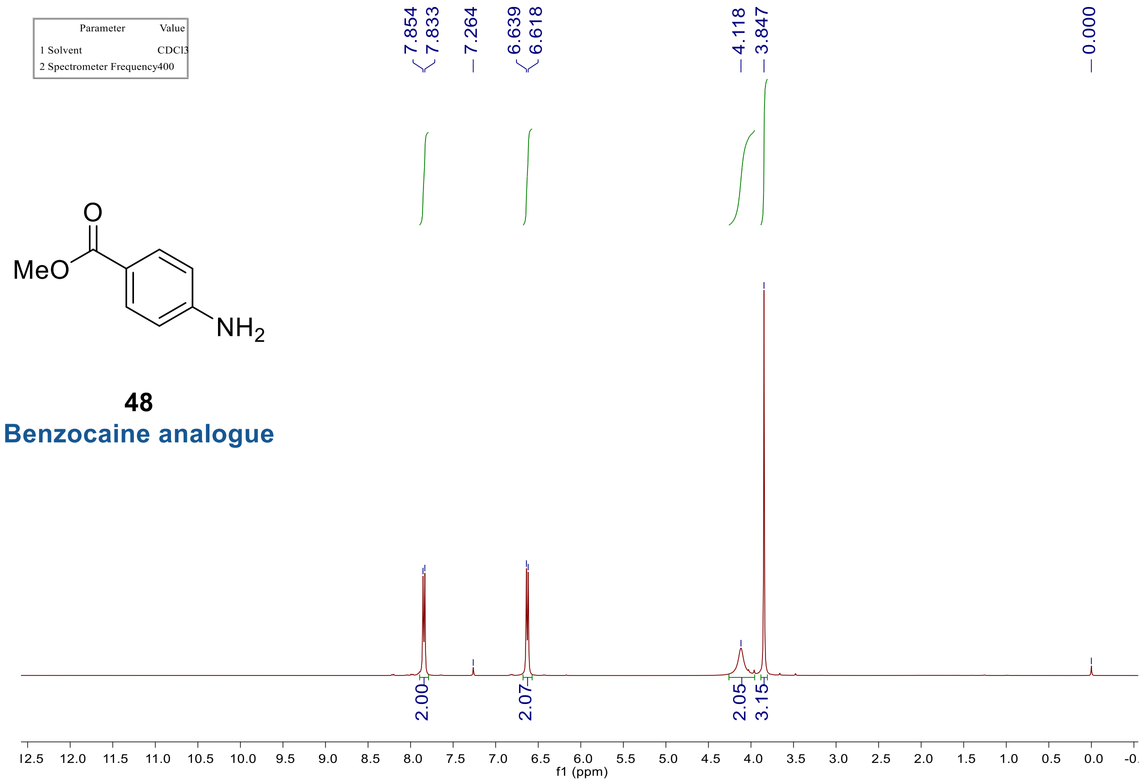

Supplementary Figure 102. ^{1}H NMR spectra of compound **49** (400 MHz, $CDCl_3$)

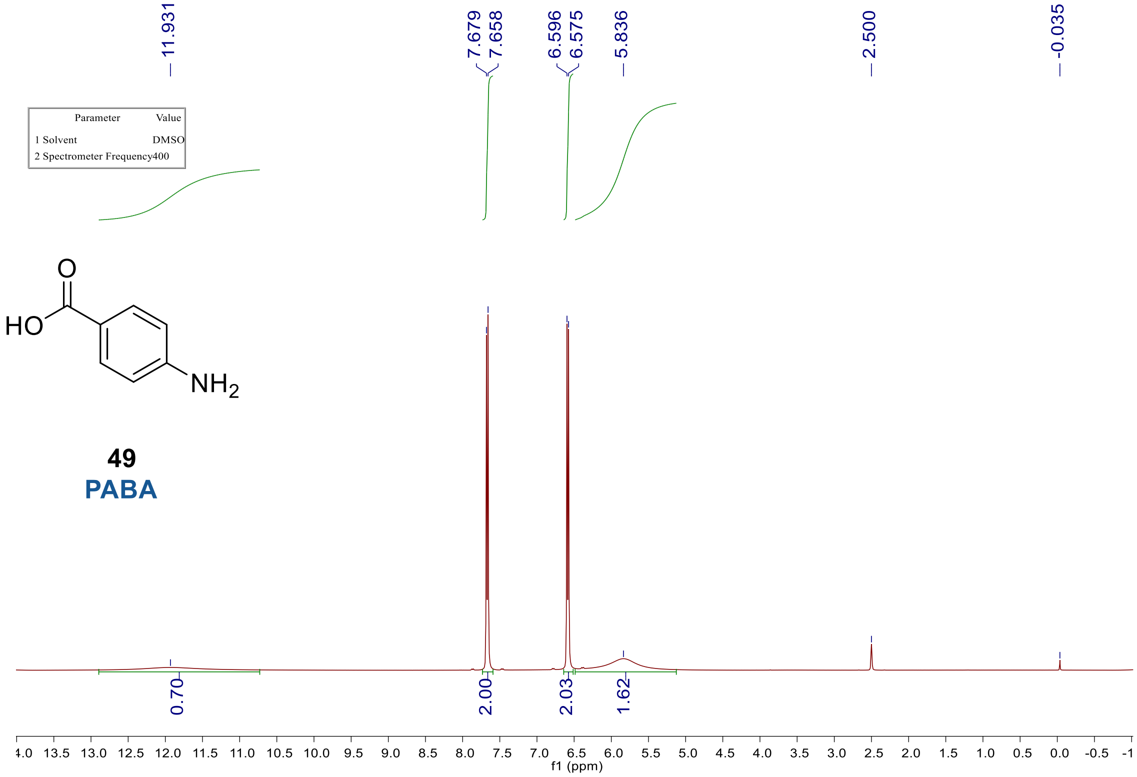

Supplementary Figure 103. ^{1}H NMR spectra of compound **50** (400 MHz, $CDCl_3$)

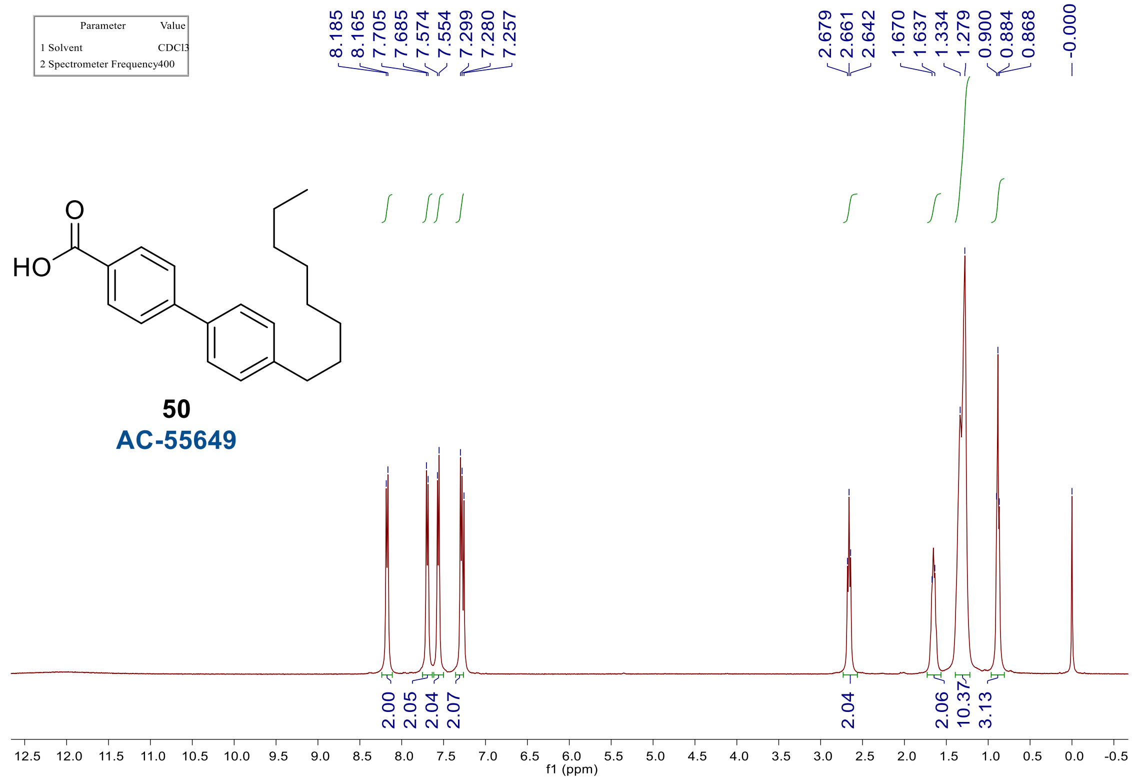

Supplementary Figure 104. ^{1}H NMR spectra of compound **51** (400 MHz, $CDCl_3$)

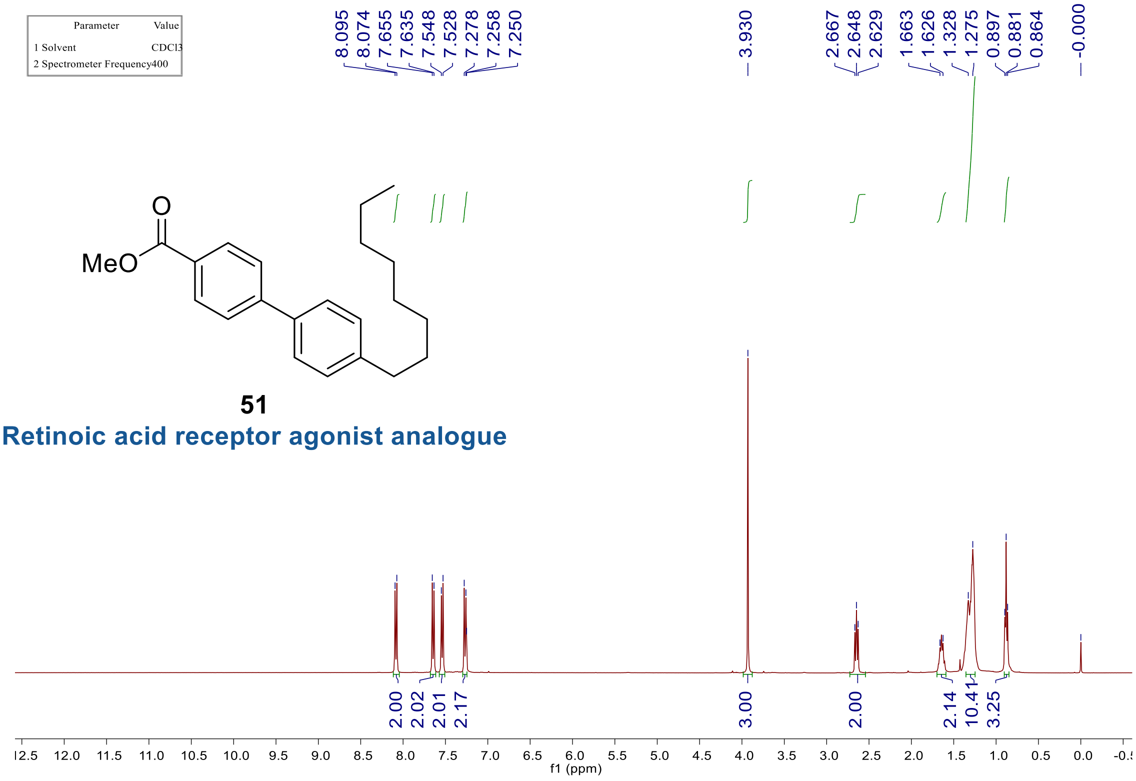

Supplementary Figure 105. ^{13}C NMR spectra of compound **51** (101 MHz, $CDCl_3$)

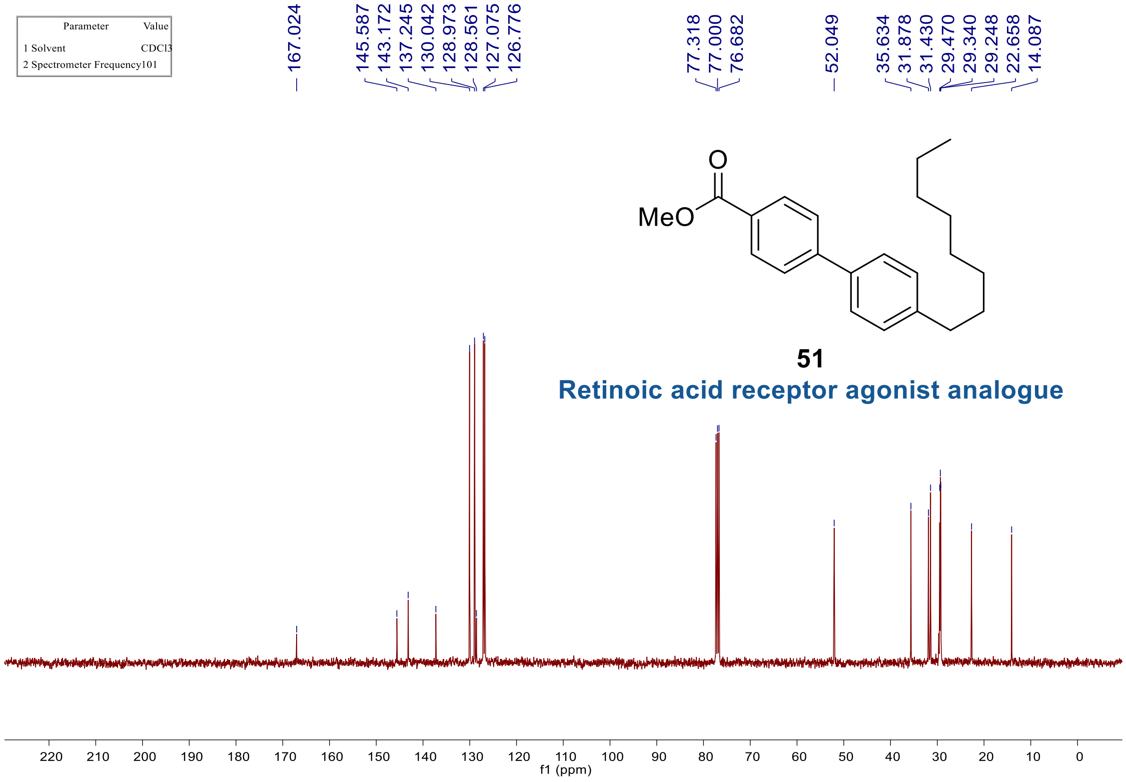

Supplementary Figure 106. ^{1}H NMR spectra of compound **52** (400 MHz, $CDCl_3$)

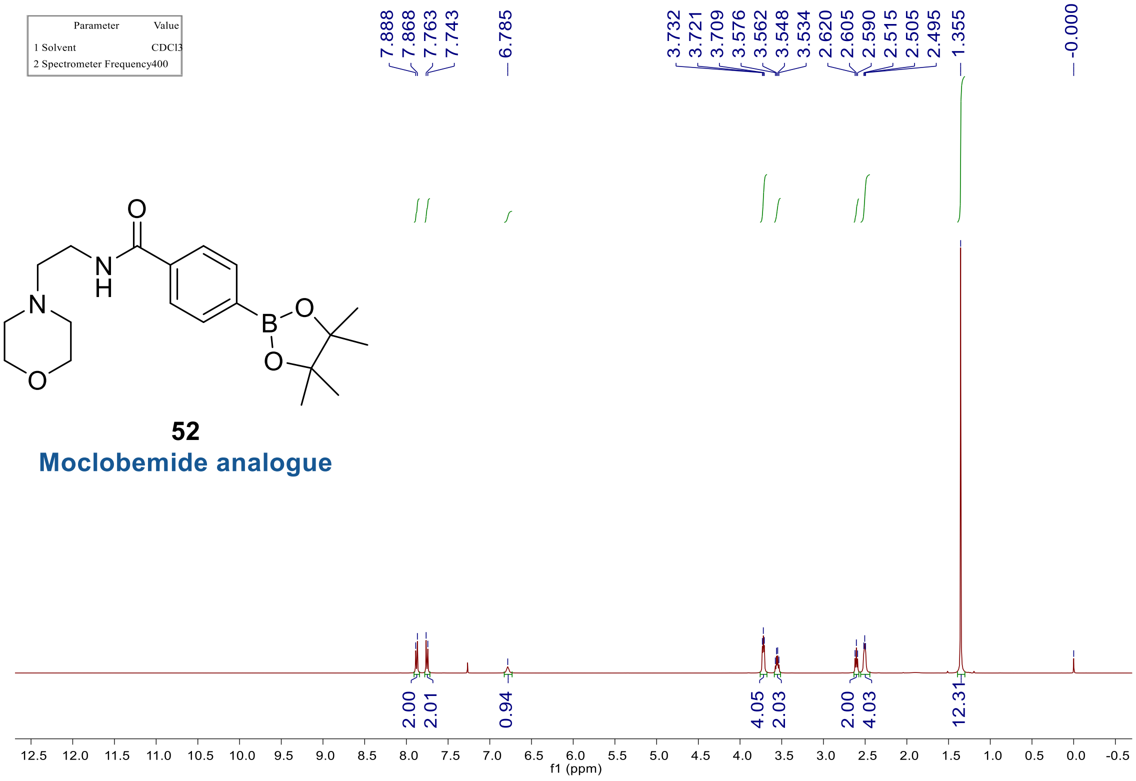

Supplementary Figure 107. ^{13}C NMR spectra of compound **52** (101 MHz, $CDCl_3$)

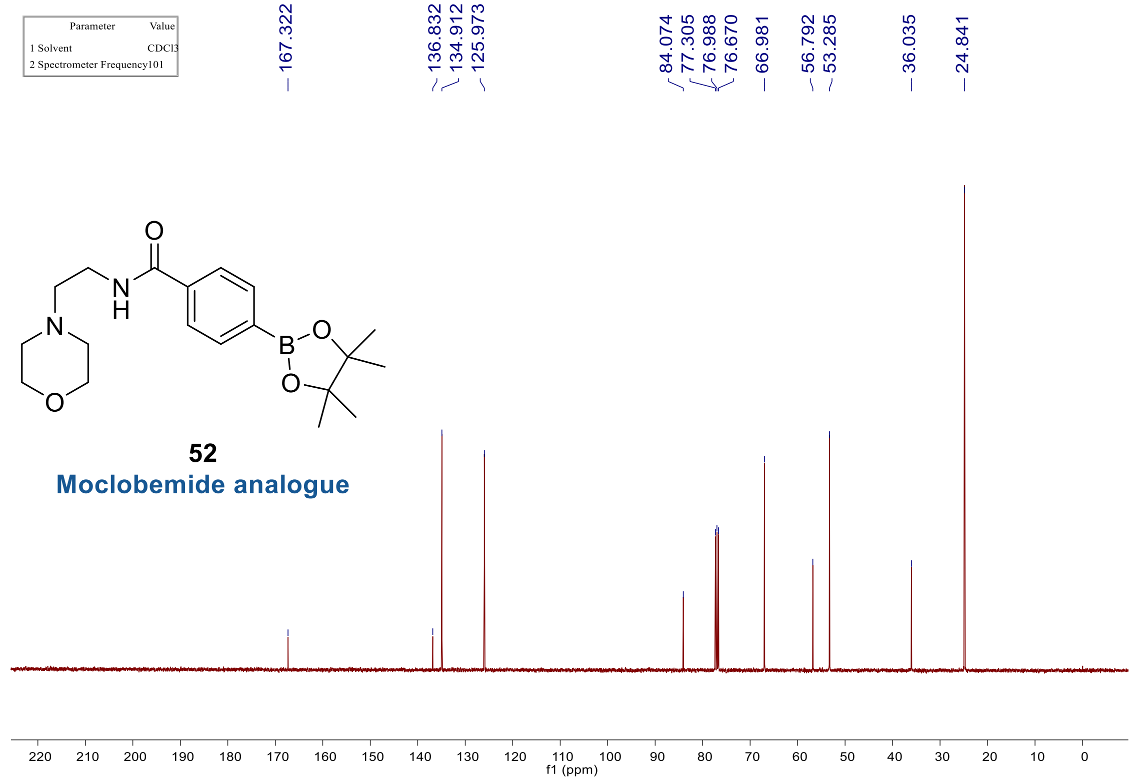

Supplementary Figure 108. ^{11}B NMR spectra of compound **52** (128 MHz, $CDCl_3$)

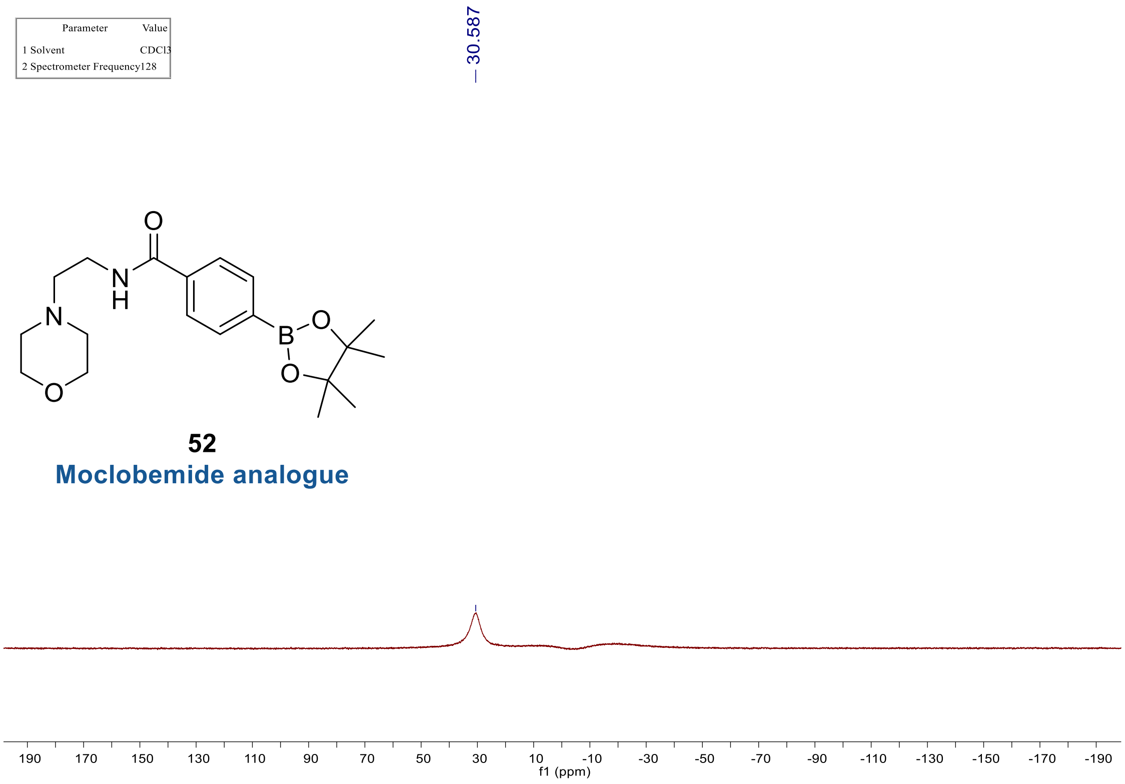

Supplementary Figure 109. ^{1}H NMR spectra of compound **53** (400 MHz, $CDCl_3$)

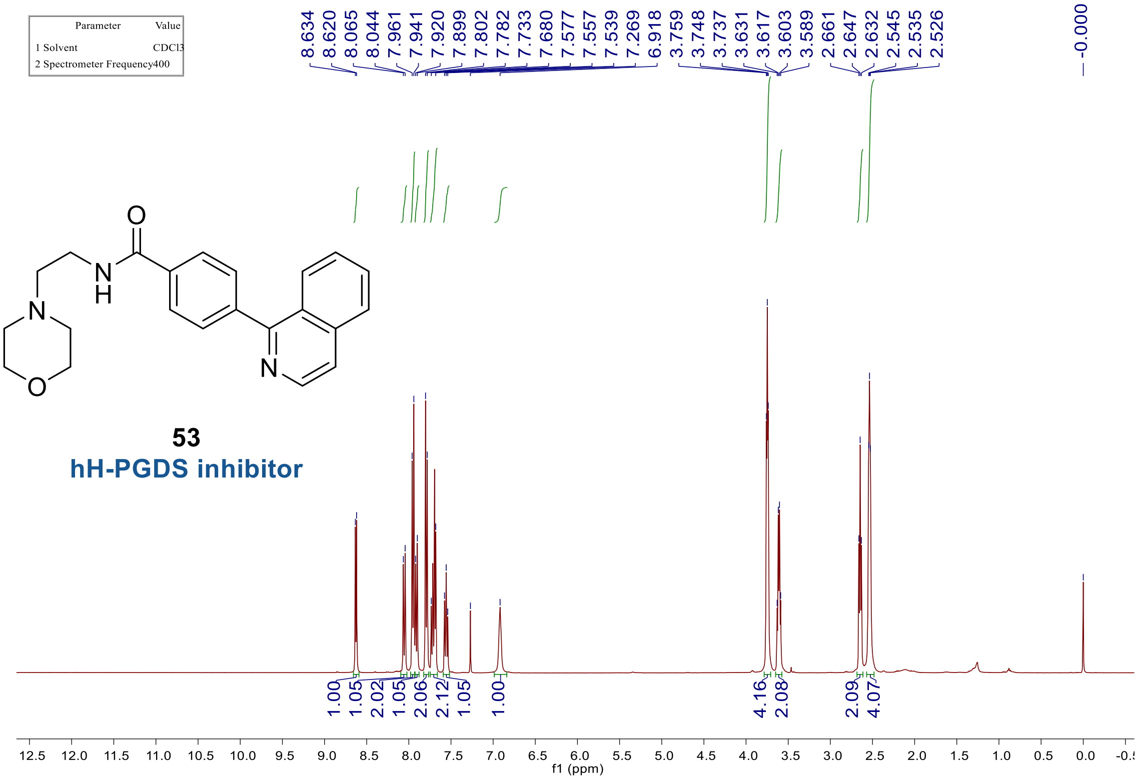

Supplementary Figure 110. ^{13}C NMR spectra of compound **53** (101 MHz, $CDCl_3$)

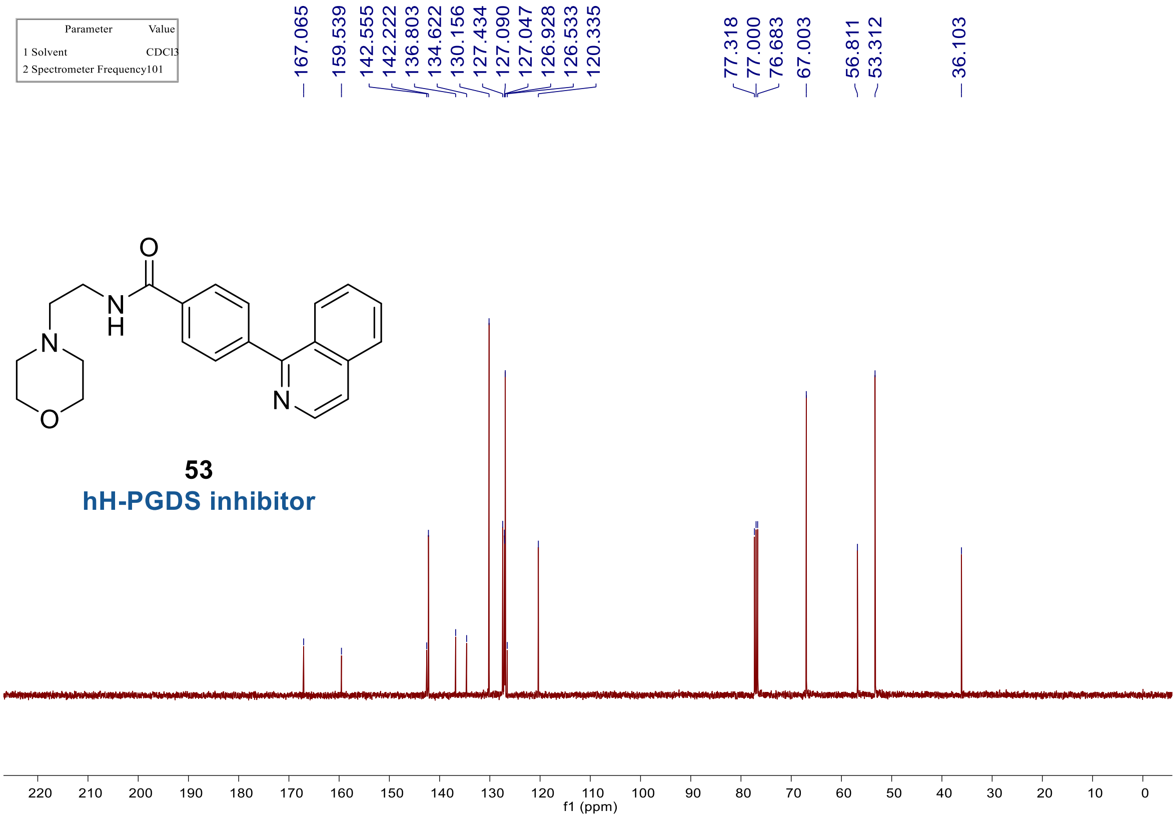

Supplementary references

1. Ha, S. *et al.* Alkyne-Alkene [2 + 2] cycloaddition based on visible light photocatalysis. *Nat Commun.*, **11**, 2509 (2020).
2. MacKenzie, I. A. *et al.* Discovery and characterization of an acridine radical photoreductant. *Nature* **580**, 76-80 (2020).
3. Romero, N. A. & Nicewicz, D. A. Organic Photoredox Catalysis. *Chem. Rev.* **116**, 10075-10166 (2016).
4. Cowper, N. G. W., Chernowsky, C. P., Williams, O. P. & Wickens, Z. K. Potent Reductants via Electron-Primed Photoredox Catalysis: Unlocking Aryl Chlorides for Radical Coupling. *J. Am. Chem. Soc.* **142**, 2093-2099 (2020).
5. Li, H. & Wenger, O. S. Photophysics of Perylene Diimide Dianions and Their Application in Photoredox Catalysis. *Angew. Chem. Int. Ed.* **61**, e202110491 (2022).
6. Kim, H., Kim, H., Lambert, T. H. & Lin, S. Reductive Electrophotocatalysis: Merging Electricity and Light To Achieve Extreme Reduction Potentials. *J. Am. Chem. Soc.* **142**, 2087-2092 (2020).
7. Chernowsky, C. P., Chmiel, A. F. & Wickens, Z. K. Electrochemical Activation of Diverse Conventional Photoredox Catalysts Induces Potent Photoreductant Activity. *Angew. Chem. Int. Ed.* **60**, 21418-21425 (2021).
8. Gaussian 16 v. Revision A.03 (Gaussian, Inc., Wallingford CT, 2016).
9. Yanai, T., Tew, D. P. & Handy, N. C. A new hybrid exchange–correlation functional using the Coulomb-attenuating method (CAM-B3LYP). *Chem. Phys. Lett.* **393**, 51-57 (2004).
10. Grimme, S., Ehrlich, S. & Goerigk, L. Effect of the damping function in dispersion corrected density functional theory. *J. Comput. Chem.* **32**, 1456-1465 (2011).
11. Weigend, F. & Ahlrichs, R. Balanced basis sets of split valence, triple zeta valence and quadruple zeta valence quality for H to Rn: Design and assessment of accuracy. *Phys. Chem. Chem. Phys.* **7**, 3297-3305 (2005).
12. Marenich, A. V., Cramer, C. J. & Truhlar, D. G. Universal Solvation Model Based on Solute Electron Density and on a Continuum Model of the Solvent Defined by the Bulk Dielectric Constant and Atomic Surface Tensions. *J. Phys. Chem. B* **113**, 6378-6396 (2009).
13. The PyMOL Molecular Graphics System (Delano Scientific, 2002).
14. Zimmermann, T. & Hennig, L. Ring transformations of heterocyclic compounds. XXII.

Pyrido[1,2-*a*]indolium salts from 2-methyl-3*H*-indoles by pyrylium mediated three carbon annelation. *J. Heterocycl. Chem.* **39**, 263-269 (2002).

15. Liu, C., Wang, M., Xu, Y., Li, Y. & Liu, Q. Manganese-Catalyzed Asymmetric Hydrogenation of 3*H*-Indoles. *Angew. Chem. Int. Ed.* **61**, e202202814 (2022).
16. Shivashimpi, G. M. *et al.* Novel unsymmetrical squaraine dye bearing cyanoacrylic acid anchoring group and its photosensitization behavior. *Tetrahedron Lett.* **53**, 5437-5440 (2012).
17. Wagaw, S., Yang, B. H. & Buchwald, S. L. A Palladium-Catalyzed Method for the Preparation of Indoles via the Fischer Indole Synthesis. *J. Am. Chem. Soc.* **121**, 10251-10263 (1999).
18. Rashidi, A., Baradarani, M. M. & Joule, J. A. The Synthesis of 6-Substituted Pyrido[2,3-*d*]pyrimidine-2,4(1*H*,3*H*)-diones Using Aminomethylene Malondialdehydes and 6-Aminouracils. *J. Heterocycl. Chem.* **51**, 1068-1072 (2014).
19. Punzi, A. *et al.* Croconaines as molecular materials for organic electronics: synthesis, solid state structure and use in transistor devices. *J. Mater. Chem. C* **4**, 3138-3142 (2016).
20. Pavlovska, T. *et al.* Tuning Deazaflavins Towards Highly Potent Reducing Photocatalysts Guided by Mechanistic Understanding – Enhancement of the Key Step by the Internal Heavy Atom Effect. *Chem. Eur. J.* **28**, e202200768 (2022).
21. Li, H., Luan, Z.-J., Zheng, G.-W. & Xu, J.-H. Efficient Synthesis of Chiral Indolines using an Imine Reductase from Paenibacillus lactis. *Adv. Synth. Catal.* **357**, 1692-1696 (2015).
22. Gao, C., Xuan, Q. & Song, Q. Cu-Catalyzed Chemoselective Reduction of *N*-Heteroaromatics with $NH_3 \cdot BH_3$ in Aqueous Solution. *Chin. J. Chem .* **39**, 2504-2508 (2021).
23. Lackner, A. D., Samant, A. V. & Toste, F. D. Single-Operation Deracemization of 3*H*-Indolines and Tetrahydroquinolines Enabled by Phase Separation. *J. Am. Chem. Soc.* **135**, 14090-14093 (2013).
24. Liddon, J. T. R., Rossi-Ashton, J. A., Taylor, R. J. K. & Unsworth, W. P. Dearomatizing Spiroannulation Reagents: Direct Access to Spirocycles from Indoles and Dihalides. *Org. Lett.* **20**, 3349-3353 (2018).
25. Chatterjee, A. & König, B. Birch-Type Photoreduction of Arenes and Heteroarenes by Sensitized Electron Transfer. *Angew. Chem. Int. Ed.* **58**, 14289-14294 (2019).
26. Hoshimoto, Y., Kinoshita, T., Hazra, S., Ohashi, M. & Ogoshi, S. Main-Group-Catalyzed Reductive Alkylation of Multiply Substituted Amines with Aldehydes Using H_2. *J. Am. Chem. Soc.* **140**, 7292-7300 (2018).

27. Jiang, M., Yang, H. & Fu, H. Visible-Light Photoredox Borylation of Aryl Halides and Subsequent Aerobic Oxidative Hydroxylation. *Org. Lett.* **18**, 5248-5251 (2016).
28. Hatakeyama, T., Hashimoto, S., Ishizuka, K. & Nakamura, M. Highly Selective Biaryl Cross-Coupling Reactions between Aryl Halides and Aryl Grignard Reagents: A New Catalyst Combination of *N*-Heterocyclic Carbenes and Iron, Cobalt, and Nickel Fluorides. *J. Am. Chem. Soc.* **131**, 11949-11963 (2009).
29. Jin, S. *et al.* Visible Light-Induced Borylation of C–O, C–N, and C–X Bonds. *J. Am. Chem. Soc.* **142**, 1603-1613 (2020).
30. Tian, Y.-M. *et al.* Visible-Light-Induced Ni-Catalyzed Radical Borylation of Chloroarenes. *J. Am. Chem. Soc.* **142**, 18231-18242 (2020).
31. Ochiai, H., Uetake, Y., Niwa, T. & Hosoya, T. Rhodium-Catalyzed Decarbonylative Borylation of Aromatic Thioesters for Facile Diversification of Aromatic Carboxylic Acids. *Angew. Chem. Int. Ed.* **56**, 2482-2486 (2017).
32. Ludwig, J. R., Simmons, E. M., Wisniewski, S. R. & Chirik, P. J. Cobalt-Catalyzed C(sp^2)–C(sp^3) Suzuki–Miyaura Cross Coupling. *Org. Lett.* **23**, 625-630 (2021).
33. Demory, E., Blandin, V., Einhorn, J. & Chavant, P. Y. Noncryogenic Preparation of Functionalized Arylboronic Esters through a Magnesium−Iodine Exchange with in Situ Quench. *Org. Process Res. Dev.* **15**, 710-716 (2011).
34. Chen, K., He, P., Zhang, S. & Li, P. Synthesis of aryl trimethylstannanes from aryl halides: an efficient photochemical method. *Chem. Commun.* **52**, 9125-9128 (2016).
35. Chen, Y.-J. *et al.* Transition-Metal-Free, Site-Selective C–F Arylation of Polyfluoroarenes via Electrophotocatalysis. *J. Am. Chem. Soc.* **144**, 17261-17268 (2022).
36. Wang, J., Tang, M., Gu, W., Huang, S. & Xie, L.-G. Synthesis of Pyrrole via Formal Cycloaddition of Allyl Ketone and Amine under Metal-Free Conditions. *J. Org. Chem.* **87**, 12482-12490 (2022).
37. Li, H. *et al.* Polysulfide Anions as Visible Light Photoredox Catalysts for Aryl Cross-Couplings. *J. Am. Chem. Soc.* **143**, 481-487 (2021).
38. Furukawa, T., Tobisu, M. & Chatani, N. C-H functionalization at sterically congested positions by the platinum-catalyzed borylation of arenes. *J. Am. Chem. Soc.* **137**, 12211-12214 (2015).
39. Zhu, C., Wang, R. & Falck, J. R. Mild and Rapid Hydroxylation of Aryl/Heteroaryl Boronic

Acids and Boronate Esters with *N*-Oxides. *Org. Lett.* **14**, 3494-3497 (2012).

40. Wang, Z.-Q. *et al.* Highly active bidentate *N*-heterocyclic carbene/ruthenium complexes performing dehydrogenative coupling of alcohols and hydroxides in open air. *Chem. Commun.* **55**, 8591-8594 (2019).
41. Li, Z. *et al.* Palladium Nanoparticles Supported on Nitrogen-Functionalized Active Carbon: A Stable and Highly Efficient Catalyst for the Selective Hydrogenation of Nitroarenes. *ChemCatChem* **6**, 1333-1339 (2014).
42. Prathap, K. J., Wu, Q., Olsson, R. T. & Dinér, P. Catalytic Reductions and Tandem Reactions of Nitro Compounds Using in Situ Prepared Nickel Boride Catalyst in Nanocellulose Solution. *Org. Lett.* **19**, 4746-4749 (2017).
43. Pedersen, P. J. *et al.* Liposomal Formulation of Retinoids Designed for Enzyme Triggered Release. *J. Med. Chem.* **53**, 3782-3792 (2010).
44. Li, G., Ji, C.-L., Hong, X. & Szostak, M. Highly Chemoselective, Transition-Metal-Free Transamidation of Unactivated Amides and Direct Amidation of Alkyl Esters by N–C/O–C Cleavage. *J. Am. Chem. Soc.* **141**, 11161-11172 (2019).
