# Supplementary material for: Electrophotocatalytic hydrogenation of imines and reductive functionalization of aryl halides
Source: Nat Commun. 2024 Jan 22;15:655. doi: 10.1038/s41467-024-45015-6 (PMC10803379; doi:10.1038/s41467-024-45015-6)
Supplement: Supplementary file 3 — Description of Additional Supplementary Files [file 41467_2024_45015_MOESM3_ESM.pdf]

File Name: Supplementary Data 1

Description: The coordinates of optimized structures
